# Supplementary material for: Effects of Maternal High-Fructose Diet on Long Non-Coding RNAs and Anxiety-like Behaviors in Offspring
Source: Int J Mol Sci. 2023 Feb 24;24(5):4460. doi: 10.3390/ijms24054460 (PMC10003385; doi:10.3390/ijms24054460)
Supplement: Supplementary file 1 [file ijms-24-04460-s001.zip › Table S1.pdf]

Table S1: Primer's sequence of significant DElncRNAs in Con/F13% and Con/F40%.

Gene name	Sequence (5′-3′)	Sequence (3′-5′)
**Con vs F13%**		
ONT.13539	GAACCAATCTGGAGACGGGATATGC	TCTGCTTTGTGGAATGGTGTGGATC
ONT.119	AGAGGAGGAGGAGGACGGAGAG	TGCTTCAGCCTGAGTGTTGGATTC
ONT.13715	CCTGTGCGATGTCCTCCTTGTTG	CCCTTTCCTGGCTGCTTCTTCTC
ONT.11765	GTGGTGACCTCGGAGTTTCGTG	GCAGTCCATGCTCTTAACCTCTGAG
**Con vs F40%**		
ONT.11295	CTGCTTTTCCAGAGGTCCTGAGTTC	AGACCAGAAGAGGGCATCAGATCC
ONT.1222	GAAACGGCATCTGGGAAGGTCTG	TTCATCAACACTGGAGAGCAAGCC
ONT.5939	AGGACCCTCAGGAGATGAACAGATG	TCACAGGAGCCTTTGGGAAACAATG
ONT.252	TCTACCTCCTGTTTGCGTGATGTTC	CAGGGCACGGAAGAGTCTTTAATGG
