# Supplementary material for: Effects of Maternal High-Fructose Diet on Long Non-Coding RNAs and Anxiety-like Behaviors in Offspring
Source: Int J Mol Sci. 2023 Feb 24;24(5):4460. doi: 10.3390/ijms24054460 (PMC10003385; doi:10.3390/ijms24054460)
Supplement: Supplementary file 1 [file ijms-24-04460-s001.zip › Table S2.pdf]

Table S2: The target genes were identified by cis method.

#lncRNA	Genes	Symbol
ONT.4436.1	ONT.4437;ENSRNOG00000057703;ENSRNOG00000007849;ENSRNOG00000059061	ONT.4437;Cabp7;Zmat5;Uqcr10
ONT.2319.1	ENSRNOG00000000251;ENSRNOG00000011182;ENSRNOG00000011459;ENSRNOG00000012083;ENSRNOG00000011541;ENSRNOG000	St6galnac1;Aanat;Rhbdf2;St6galnac2;Cygb;AC123144.1
ONT.9569.1	ONT.9571;ENSRNOG00000009742	ONT.9571;Rad52
ONT.13035.2	ENSRNOG00000048394;ENSRNOG00000025937;ENSRNOG00000033624;ENSRNOG00000033025;ENSRNOG00000028864;ONT.13036	Zfp560;Zfp26;Zfp426;Olr1192;Olr1193;ONT.13036
ONT.10464.1	ONT.10934;ENSRNOG0000000957	ONT.10934;Ubiad1
ONT.8030.2	ENSRNOG00000000818;ENSRNOG00000032596;ENSRNOG00000059268;ENSRNOG00000000799;ENSRNOG00000000787;ENSRNOG00000000812;ONT.8031;ENSRNOG00000000816;ENSRNOG00000000804;ENSRNOG00000030157;ENSRNOG00000045924;ENSRNOG00000000809;ENSRNOG00000000798	Nrm;RT1-T24-1;Ppp1r10;Abcf1;AABR07044364.1;RGD1302996;ONT.8031;Ppp1r18;Mrps18b;Dhx16;RT1-T24-3;Atat1;Gnl1
ONT.9189.1	ENSRNOG00000009207;ENSRNOG00000008354;ONT.8661	Spata2;Slc9a8;ONT.8661
ONT.2180.1	ENSRNOG00000036894;ENSRNOG00000053285;ENSRNOG00000011475;ONT.2182;ENSRNOG00000048187;ENSRNOG00000052730;ENSRNOG00000012705;ONT.2179;O	Cisd3;Mllt6;Srcin1;ONT.2182;Epop;Psmb3;Pcgf2;ONT.2179;ONT.2772
ONT.115.1	ENSRNOG00000042826;ENSRNOG00000043341;ENSRNOG00000058436;ENSRNOG00000042347	Zfp52;Zfp51;LOC108348215;AABR07001896.1
ONT.6840.1	ENSRNOG00000015695;ENSRNOG00000015335;ENSRNOG00000015150;ONT.6612;ENSRNOG00000015397;ONT.6841	Sult5a1;Rpl13;Spg7;ONT.6612;Cpne7;ONT.6841
ONT.10838.2	ENSRNOG00000048288;ENSRNOG00000046922;ENSRNOG00000055344;ENSRNOG00000055426;ONT.10387;ONT.10388;ENSRNOG0000	Taf12;Phactr4;Trnau1ap;Rab42;ONT.10387;ONT.10388;Rcc1
ONT.10794.2	ENSRNOG00000017426;ENSRNOG00000037368;ENSRNOG00000017166;ENSRNOG00000026592	Rragc;AABR07049862.1;Mycbp;Rhbdl2
ONT.9965.1	ONT.9567;ENSRNOG00000008168;ENSRNOG0000007508;ENSRNOG00000007990	ONT.9567;Wnt5b;Lrtm2;Adipor2
ONT.7094.2	ENSRNOG00000032715	AABR07010705.1

ONT.5245.2	ENSRNOG00000016186;ENSRNOG00000060701;ONT.5460;ENSRNOG00000052038;ENSRNOG000000	Zfp709;AC130232.2;ONT.5460;Haus8;Zfp617
ONT.2526.1	ENSRNOG00000002682;ENSRNOG00000054400;ENSRNOG00000053179;ENSRNOG00000002713;ENSRNOG00000026844;ENSRNOG000	Zfp692;Sh3bp5l;LOC691277;Zfp672;Lypd8;RGD1308564
ONT.671.1	ENSRNOG00000021098;ONT.670;ENSRNOG00000021108	Rasgrp2;ONT.670;Slc22a12
ONT.8883.1	ENSRNOG00000001524;ENSRNOG00000001519;ENSRNOG00000061587;ENSRNOG00000048003;ENSRNOG00000001520	Hat1;Dlx2;Metap1d;AABR07052498.1;Dlx1
ONT.4577.1	ENSRNOG00000057975	AABR07014882.1
ONT.6324.5	ENSRNOG00000019276	Dele1
ONT.6686.2	ONT.6684;ENSRNOG00000014124;ONT.6685;ENSRNOG0000001420	ONT.6684;Nod2;ONT.6685;Snx20
ONT.6847.1	ENSRNOG00000053560	Rhou
ONT.175.1	ENSRNOG00000057645;ENSRNOG00000013847;ONT.1024;ENSRNOG00000013767;ENSRNOG00000026891;ONT.177;ENSRNOG00000038088;ENSRNOG00000014132;ENSRNOG00000013395;ENSRNOG00000038087;ENSRNOG00000014252	Micb;Nova2;ONT.1024;Ccdc61;AC093995.1;ONT.177;Nanos2;Mypop;Pglyrp1;AC110846.1;Irf2bp1;ONT.176
ONT.1569.1	ENSRNOG00000021202;ENSRNOG00000032042	Rtn3;RGD1560108
ONT.13331.1	ENSRNOG00000021660;ENSRNOG00000021548;ENSRNOG00000021602;ENSRNOG00000054063;ENSRNOG00000031420;ENSRNOG00000015858;ENSRNOG00000016093;ENSRNOG00000016150;ENSRNOG00000021899;ENSRNOG00000016512;ENSRNOG0000021528;ENSRNOG00000052634;ENSRNOG000	Nprl2;Rassf1;Zmynd10;Naa80;Hyal2;Hyal1;Hyal3;Ifrd2;Tmem115;Sema3b;Tusc2;Lsmem2;Cyb561d2
ONT.9136.2	ENSRNOG00000019330;ENSRNOG00000049497;ONT.9135;ENSRNOG00000046772	Procr;Eif6;ONT.9135;Fam83c
ONT.131.1	ENSRNOG00000022129;ENSRNOG00000015456;ENSRNOG0000004	Galp;Zfp787;Zscan5b
ONT.13441.20	ENSRNOG00000047003	AABR07066693.1
ONT.11668.1	ONT.11669;ENSRNOG00000004351;ENSRNOG00000003020;ONT.11321;ENSRNOG00000011716;ENSRNOG00000004339	ONT.11669;Slc25a29;Slc25a47;ONT.11321;Degs2;Yy1
ONT.107.1	ENSRNOG00000001490;ENSRNOG00000001488;ENSRNOG00000001489;ENSRNOG00000021493	Pdcd2;Psmb1;Tbp;Prdm9

ONT.7165.1	ENSRNOG00000020797;ENSRNOG00000020791;ONT.7630;ONT.7167;ENSRNOG00000020778;ENSRNOG00000020744	She;Ube2q1;ONT.7630;ONT.7167;Chrnb2;Adar
ONT.86.1	ENSRNOG00000018007;ENSRNOG00000018012;ONT.84;ENSRNOG00000017889	Gtf2h5;Tulp4;ONT.84;Serac1
ONT.9480.7	ONT.9481;ONT.9877;ENSRNOG00000017380;ONT.9876	ONT.9481;ONT.9877;RGD1306746;ONT.9876
ONT.6359.4	ENSRNOG00000018082;ENSRNOG00000018414	Slc26a2;Csf1r
ONT.5880.9	ENSRNOG00000033625;ENSRNOG00000023688	AABR07027015.1;Drd1
ONT.7138.4	ENSRNOG00000028765;ENSRNOG00000019201;ENSRNOG00000022101;ENSRNOG00000018681;ENSRNOG00000018798;ENSRNOG00000018870;ENSRNOG00000027894;	AABR07072748.1;Naxe;Crabp2;Nes;Bcan;Hapln2;Iqgap3;ONT.7602
ONT.10116.1	ONT.10115;ENSRNOG0000000690	ONT.10115;Pou3f2
ONT.7708.12	ENSRNOG00000060988	Kcnc4
ONT.11606.1	ONT.11268;ENSRNOG00000011111;ENSRNOG00000011026	ONT.11268;Cipc;Irf2bpl
ONT.2511.1	ENSRNOG00000008144	Irf1
ONT.10830.1	ENSRNOG00000024788;ENSRNOG00000013179;ENSRNOG00000012879;ENSRNOG00000012989	RGD1562036;Tinagl1;Fabp3;Serinc2
ONT.8027.1	ENSRNOG00000000787;ENSRNOG00000000795;ENSRNOG00000000777;ENSRNOG00000042905;ENSRNOG00000045924;ONT.7819	AABR07044364.1;RT1-N3;RT1-S3;RT1-T24-4;RT1-T24-3;ONT.7819
ONT.11657.6	ENSRNOG00000004206;ENSRNOG00000031889	Glrx5;AABR07065438.1
ONT.7924.4	ENSRNOG00000052415;ONT.8115;ENSRNOG00000038300;ENSRNOG00000028356;ONT.7923;ENSRNOG00000028344;ENSRNOG00000028394;ENSRNOG00000028386;ENSRNOG00000028243;ENSRNOG00000049771;ENSRNOG00000028302	Gstt2;ONT.8115;Gstt4;LOC103694872;ONT.7923;Mmp11;Vpreb3;LOC103694873;LOC103694875;Gstt1;LOC103694876
ONT.8113.3	ENSRNOG00000028441;ENSRNOG00000028302;ENSRNOG00000028243;ONT.8112;ENSRNOG00000028386;ENSRNOG00000006589;ENSRNOG00000028394;ENSRNOG00000028344;ONT.7923;ENSRNOG00000028356;ENSRNOG0000002997	LOC100909869;LOC103694876;LOC103694875;ONT.8112;LOC103694873;LOC103694877;Vpreb3;Mmp11;ONT.7923;LOC103694872;LOC103694884;ONT.8115
ONT.12720.18	ONT.13160	ONT.13160
ONT.13276.1	ENSRNOG00000010699;ENSRNOG00000043223	Trim43a;Zfp949
ONT.8660.3	ENSRNOG00000008081	Ddx27

ONT.12775.1	ENSRNOG00000014846	Anp32a
ONT.4848.1	ENSRNOG00000022597	Cenpj
ONT.3914.1	ENSRNOG00000005229;ENSRNOG00000057851;ENSRNOG0000000	Sec16b;LOC680254;Tp53i3
ONT.3459.18	ENSRNOG00000022171;ONT.3714;ENSRNOG00000000979	Dhx37;ONT.3714;Bri3bp
ONT.11141.3	ONT.11508;ENSRNOG00000047891;ENSRNOG00000031317	ONT.11508;Foxg1;AABR07064224.1
ONT.9375.4	ONT.9373;ONT.9372;ENSRNOG00000027445;ENSRNOG00000006814;ONT.9777;ONT.9778	ONT.9373;ONT.9372;AABR07060293.1;Klrg2;ONT.9777;ONT.9778
ONT.5739.1	ENSRNOG00000053920;ENSRNOG00000056708;ENSRNOG00000051823;ENSRNOG00000053635	Olr1658;Nkapl;Zkscan4;Zkscan8
ONT.8195.2	ENSRNOG00000050735;ONT.8020;ONT.8194	Cd99;ONT.8020;ONT.8194
ONT.8863.1	ENSRNOG00000006623;ENSRNOG00000006241	Cd302;March7
ONT.12072.1	ENSRNOG00000059545;ONT.12071;ENSRNOG00000053675;ENSRNOG00000061607;ENSRNOG00000021438;ENSRNOG00000054385	AC114446.1;ONT.12071;Dhh;Lmbr1l;Tuba1c;Rhebl1
ONT.12642.4	ONT.12643;ONT.13073	ONT.12643;ONT.13073
ONT.12663.12	ONT.13095	ONT.13095
ONT.583.1	ENSRNOG00000016935;ONT.582;ENSRNOG00000017243;ENSRNOG00000016940	Mapk1ip1;ONT.582;Bnip3;Ppp2r2d
ONT.4275.2	ENSRNOG00000002158	Ibsp
ONT.7773.1	ENSRNOG00000048258;ENSRNOG00000042985	Cisd2;Slc9b1
ONT.3648.2	ONT.3647;ONT.3649	ONT.3647;ONT.3649
ONT.11805.1	ENSRNOG00000004294	Ascl1
ONT.1789.3	ENSRNOG00000004814;ENSRNOG00000004461;ENSRNOG00000004333;ENSRNOG00000029279;ENSRNOG00000029223;ENSRNOG00000004122;ENSRNOG00000042258;ONT.1790;ENSRNOG00000033690;ENSRNOG00000047969;ENSRNOG00000003721;ENSRNOG0000004	Elob;NEWGENE_6497122;Flywch1;Prss33;Prss32;Kremen2;RGD1561157;ONT.1790;Prss41;Prss21;Paqr4;Csap1
ONT.10048.1	ENSRNOG00000001811;ONT.9641;ENSRNOG00000001817	Fgfr1op2;ONT.9641;Tm7sf3
ONT.5671.2	ENSRNOG00000027311;ENSRNOG00000061064	Nutm2f;AABR07027128.1
ONT.1285.1	ENSRNOG00000018322	Picalm
ONT.5130.3	ONT.5129;ENSRNOG00000001049;ONT.4921;ENSRNOG00000001052;ENSRNOG00000050996	ONT.5129;Tpt1;ONT.4921;Slc25a30;Kctd4

ONT.13620.1	ENSRNOG00000019810;ENSRNOG00000019772;ENSRNOG00000019946;ENSRNOG00000019985;ENSRNOG00000020038	Des;Dnpep;Gmppa;Asic4;Chpf
ONT.1374.1	ENSRNOG00000036743;ENSRNOG00000036742;ENSRNOG0000001	AABR07005632.1;Uqcrc2;Pdzd9
ONT.5808.2	ONT.5809;ENSRNOG00000033330	ONT.5809;Skida1
ONT.968.1	ENSRNOG00000012692	Riok2
ONT.7708.14	ENSRNOG00000060988	Kcnc4
ONT.6464.12	ONT.6465;ENSRNOG00000016245	ONT.6465;Neto2
ONT.5585.4	ENSRNOG00000048769;ENSRNOG00000012878;ENSRNOG00000012757;ENSRNOG00000012841	Nek5;Atp7b;Nek3;Alg11
ONT.5987.4	ENSRNOG00000049410;ONT.5766	Chrm3;ONT.5766
ONT.8054.4	ENSRNOG00000050647;ENSRNOG00000000840;ENSRNOG00000000836;ENSRNOG00000000837;ONT.8056;ENSRNOG00000000839;ENSRNOG00000031607;ENSRNOG00000047966;ENSRNOG00000048725;ENSRNOG00000000838;ENSRNO	LOC108348108;Atp6v1g2;Ltb;LOC103694380;ONT.8056;Nfkbil1;RT1-CE3;Hspa1l;Lsm2;Lta;RT1-CE5
ONT.14125.1	ENSRNOG00000025730;ENSRNOG00000011513;ENSRNOG00000037709;ENSRNOG00000011494;ENSRNOG00000011661;ENSRNOG00000037711;ENSRNOG00000037707	Armcx3;Gla;Armcx1;Rpl36a;Hnrnph2;LOC501618;Armcx6
ONT.5939.1	ONT.5723	ONT.5723
ONT.12463.1	ENSRNOG00000005332;ENSRNOG00000024128;ENSRNOG00000005577;ONT.12015;ENSRNOG00000004471;ENSRNOG00000005572;ENSRNOG00000050500;ENSRNOG00000024177;ONT.12461;ENSRNOG00000024170;ENSRNOG0000000	Csdc2;Aco2;Desi1;ONT.12015;Polr3h;RGD1306782;Tob2;AC096601.1;ONT.12461;Phf5a;Pmm1
ONT.11555.1	ENSRNOG00000021628;ONT.11202;ENSRNOG00000051449	Wdr89;ONT.11202;AABR07064867.1
ONT.4609.1	ENSRNOG00000002192	Rell1
ONT.8025.1	ENSRNOG00000033136;ENSRNOG00000000783;ENSRNOG00000000775;ENSRNOG00000060412;ENSRNOG00000021518;ONT.7812;ENSRNOG00000000779;ENSRNOG00000000781;ENSRNOG00000022107;ENSRNOG00000000780;ENSRNOG00000040052;ENSRNOG0000002	RT1-M5;Trim40;Mog;RT1-M4;Trim31;ONT.7812;Znrd1;Rnf39;RT1-M6-1;Ppp1r11;RT1-M6-2;Zfp57
ONT.3923.3	ENSRNOG00000026907;ENSRNOG00000038789;ENSRNOG00000002783;ONT.3921;ENSRNOG000000	Zbtb37;Cenpl;Serpinc1;ONT.3921;Dars2
ONT.9411.6	ENSRNOG00000011175;ENSRNOG00000011814;ONT.9808;ONT.980	Hnrnpa2b1;Cbx3;ONT.9808;ONT.9809

ONT.6043.2	ENSRNOG00000031197;ENSRNOG00000024066;ONT.6042;ENSRNOG00000024071	F8;Fundc2;ONT.6042;Mtcp1
ONT.4682.1	ENSRNOG00000004515;ENSRNOG00000004940;ENSRNOG00000042041;ENSRNOG00000004672;ENSRNOG00000004640	Pes1;Rnf215;Gal3st1;Sec14l2;Mtfp1
ONT.10073.1	ENSRNOG00000006804;ENSRNOG00000052668	Ppp1r42;Tcf24
ONT.11985.1	ENSRNOG00000012090;ENSRNOG00000011305;ENSRNOG00000011507;ENSRNOG00000011170;ENSRNOG00000011020;ENSRNOG00000011214;ONT.12433;ENSRNOG00000012215;ENSRNOG0000002621	Slc16a8;Sox10;Pick1;RGD1359634;Eif3l;Polr2f;ONT.12433;Baiap2l2;Micall1
ONT.11910.1	ONT.11911	ONT.11911
ONT.4463.3	ONT.4464	ONT.4464
ONT.14308.1	ONT.14309;ENSRNOG00000002662;ENSRNOG00000002660	ONT.14309;Pbdc1;Magee1
ONT.4186.4	ENSRNOG00000007705;ENSRNOG00000032378;ENSRNOG00000006930;ENSRNOG00000046996;ENSRNOG00000007604;ENSRNOG000	Kcnj10;Atp1a4;Casq1;Pea15;Igsf8;Kcnj9
ONT.10814.1	ENSRNOG00000043364;ENSRNOG00000005935	Zfp362;A3galt2
ONT.7962.17	ENSRNOG00000000572;ONT.8148	Chst3;ONT.8148
ONT.10811.1	ENSRNOG00000011463;ENSRNOG00000027595	Psmb2;Tfap2e
ONT.4463.11	ONT.4464	ONT.4464
ONT.4580.1	ENSRNOG00000002106	Usp46
ONT.6709.1	ENSRNOG00000005619;ONT.6708;ONT.6707;ENSRNOG00000005911;ONT.6481;ENSRNOG00000005115;ENSRNOG00000006049;ENSRNOG00000039252;ENSRNOG00000005257;ENSRNOG00000005747;ENSRNOG00000006551;ENSRNOG00000052637;ENSRNOG00000005729;ENSRNOG00000029134	Misp3;ONT.6708;ONT.6707;Rln3;ONT.6481;Asf1b;Rfx1;Gm10644;Prkaca;Il27ra;Dcaf15;Samd1;Palm3;Adgrl1
ONT.4789.1	ENSRNOG00000009481	Ddhd1
ONT.3610.1	ENSRNOG00000001006	Nptx2
ONT.7803.1	ONT.7351	ONT.7351
ONT.2323.1	ONT.2324;ENSRNOG00000002722;ONT.2326	ONT.2324;Sec14l1;ONT.2326
ONT.8007.5	ENSRNOG00000000314	Sec63
ONT.12895.1	ONT.13307;ENSRNOG00000009434;ENSRNOG00000009198	ONT.13307;RGD1310507;Rab6b
ONT.5880.3	ENSRNOG00000023688;ENSRNOG00000033625	Drd1;AABR07027015.1

ONT.5932.1	ENSRNOG00000017820;ONT.5931;ENSRNOG00000049124	Nqo2;ONT.5931;AABR07027466.1
ONT.12154.1	ENSRNOG00000032202;ENSRNOG00000019822;ENSRNOG00000019891;ENSRNOG00000019924;ENSRNOG00000020005;ONT.12153;ENSRNOG00000045524;ENSRNOG00000019857;ENSRNOG000000297	Creb3l3;Gadd45b;Sgta;Thop1;Map2k2;ONT.12153;Slc39a3;Gng7;Diras1
ONT.14337.2	ONT.14142;ENSRNOG00000049849;ENSRNOG00000037661;ENSRNOG00000045618;ENSRNOG00000048011;ONT.14141	ONT.14142;Fam199x;Tmsb15b2;AABR07040686.1;AABR07040695.1;ONT.14141
ONT.671.2	ONT.670;ENSRNOG00000021098;ENSRNOG00000021108	ONT.670;Rasgrp2;Slc22a12
ONT.8505.1	ENSRNOG00000004763;ONT.9049	Sirpa;ONT.9049
ONT.7773.2	ENSRNOG00000048258;ENSRNOG00000013741;ENSRNOG0000004	Cisd2;Ube2d3;Slc9b1
ONT.11215.1	ONT.11563	ONT.11563
ONT.4823.2	ENSRNOG00000011592;ENSRNOG00000010947;ENSRNOG00000039297;ENSRNOG00000012046;ENSRNOG00000039284;ENSRNOG00000011646;ENSRNOG00000009713;ENSRNOG00000010296	Lrp10;Mmp14;Mrpl52;Prmt5;Haus4;Rem2;Oxa1l;Slc7a7
ONT.11552.1	ENSRNOG00000009296	Snapc1
ONT.87.1	ENSRNOG00000018207;ENSRNOG00000018158;ENSRNOG0000001	Dynlt1;Tmem181;Tulp4
ONT.8755.1	ENSRNOG00000056675;ENSRNOG00000056471;ENSRNOG00000019466;ENSRNOG00000019388	LOC100911507;AABR07051251.1;Agpat2;Egfl7
ONT.7708.7	ENSRNOG00000060988	Kcnc4
ONT.8932.2	ENSRNOG00000013594;ENSRNOG00000011208;ENSRNOG00000013172;ENSRNOG00000011981;ENSRNOG00000012568;ENSRNOG00000011414;ENSRNOG00000012307	Acp2;Rapsn;Nr1h3;Slc39a13;Madd;Psmc3;Mybpc3
ONT.3459.16	ENSRNOG00000022171;ENSRNOG00000000979;ONT.3714	Dhx37;Bri3bp;ONT.3714
ONT.10125.4	ENSRNOG00000007434;ENSRNOG00000007490	Ube2j1;Gabrr2
ONT.1534.4	ENSRNOG00000056038;ONT.1535;ENSRNOG00000024239;ENSRNOG00000023668;ONT.1536;ENSRNOG00000020881;ENSRNOG00000020813;ENSRNOG00000023318;ENSRNOG00000030616;ENSRNOG00	Ehbp1l1;ONT.1535;Fam89b;Scyl1;ONT.1536;Frmd8;Ltbp3;Tigd3;Slc25a45;Sssca1
ONT.11189.1	ENSRNOG00000004499;ENSRNOG00000004751	Gpr135;Jkamp
ONT.6918.2	ENSRNOG00000025274;ONT.6917;ENSRNOG00000025285;ONT.739	Hexb;ONT.6917;Gfm2;ONT.7394

ONT.8444.1	ENSRNOG00000007906;ONT.8446;ONT.8998;ENSRNOG00000042446;ENSRNOG00000058337	Bub1b;ONT.8446;ONT.8998;Ankrd63;Plcb2
ONT.3648.3	ONT.3649;ONT.3647;ENSRNOG00000060153	ONT.3649;ONT.3647;Spry3
ONT.13631.5	ONT.13912	ONT.13912
ONT.5396.1	ONT.5397	ONT.5397
ONT.13374.1	ENSRNOG00000008981	Pdcd6ip
ONT.3459.14	ENSRNOG00000000979;ONT.3714;ENSRNOG00000022171	Bri3bp;ONT.3714;Dhx37
ONT.5176.1	ENSRNOG00000010989	Ipo5
ONT.10587.1	ENSRNOG00000007434;ONT.10586;ENSRNOG00000007490	Ube2j1;ONT.10586;Gabrr2
ONT.7708.8	ENSRNOG00000060988	Kcnc4
ONT.5788.1	ONT.6012	ONT.6012
ONT.4476.3	ENSRNOG00000042277	AABR07016578.1
ONT.2412.2	ENSRNOG00000053437;ENSRNOG00000008843;ENSRNOG00000009536;ENSRNOG00000003195;ENSRNOG00000042352;ENSRNOG00000009667;ENSRNOG00000009611;ENSRNOG00000042086;ENSRNOG00000009224;ENSRNOG00000008703;ENSRNOG00000003131	AC103090.1;Eci1;Pgp;Caskin1;Dnase1l2;Mlst8;Bricd5;Rab26;E4f1;Rnps1;Traf7
ONT.11725.3	ENSRNOG00000043289;ENSRNOG00000021900	LOC102551539;AABR07055834.1
ONT.8932.1	ENSRNOG00000013594;ENSRNOG00000011208;ENSRNOG00000011981;ENSRNOG00000013172;ENSRNOG00000012568;ENSRNOG00000011414;ENSRNOG00000012307	Acp2;Rapsn;Slc39a13;Nr1h3;Madd;Psmc3;Mybpc3
ONT.6832.1	ENSRNOG00000014581;ENSRNOG00000056786;ENSRNOG00000013777;ENSRNOG00000013970;ENSRNOG00000014461;ONT.6834;ONT.6608;ENSRNOG00000051531;ENSRNOG00000029558;ENSRNOG00	Trappc2l;Piezo1;Rnf166;Cdt1;Galns;ONT.6834;ONT.6608;Ctu2;Pabpn1l;Aprt
ONT.12890.1	ENSRNOG00000023021;ONT.13303;ONT.12889	Msl2;ONT.13303;ONT.12889
ONT.7962.15	ONT.8148;ENSRNOG00000000572	ONT.8148;Chst3
ONT.4291.12	ENSRNOG00000002262;ENSRNOG00000002292	Enoph1;Hnrnpd
ONT.11765.1	ENSRNOG00000014625;ENSRNOG00000016193;ONT.11763;ONT.12173;ENSRNOG00000015434;ENSRNOG00000014287;ENSRNOG00000013604;ENSRNOG00000013987;ENSRNOG00000015999;ENSRNOG00000013545;ENSRNOG00000016203;ENSRNOG00000024349	Atp5f1d;RGD1562114;ONT.11763;ONT.12173;Midn;Stk11;Gpx4;Sbno2;Cirbp;Polr2e;Efna2;Cbarp

ONT.3459.13	ENSRNOG00000022171;ONT.3714;ENSRNOG00000000979	Dhx37;ONT.3714;Bri3bp
ONT.14102.25	ENSRNOG00000061703;ONT.1430	AABR07039303.7;ONT.14301
ONT.6413.6	ENSRNOG00000051666;ENSRNOG00000050034;ENSRNOG0000006	AABR07042609.1;LOC501297;AABR07042607.1
ONT.3306.1	ENSRNOG00000001885;ENSRNOG00000042711;ONT.3304;ENSRNOG00000001884;ENSRNOG00000021891;ONT.3126;ENSRNOG00000030920;ENSRNOG00000001886	Trmt2a;AABR07072264.1;ONT.3304;Ranbp1;Zdhhc8;ONT.3126;Rtn4r;Dgcr8
ONT.6766.2	ENSRNOG00000020049;ONT.6542;ENSRNOG00000019943;ENSRNOG00000000258;ENSRNOG00000019859;ENSRNOG00000023177	Slc7a6os;ONT.6542;Slc7a6;Prmt7;Pla2g15;Esrp2
ONT.6676.2	ENSRNOG00000014761;ONT.6677	Rasd2;ONT.6677
ONT.14304.1	ONT.14108;ENSRNOG0000000292	ONT.14108;Zcchc13
ONT.7962.19	ONT.8149;ENSRNOG00000000572;ONT.8148	ONT.8149;Chst3;ONT.8148
ONT.7140.10	ENSRNOG00000019412;ENSRNOG00000019090;ENSRNOG00000031778;ENSRNOG00000037552;ONT	Rhbg;Cct3;Mef2d;Tsacc;ONT.7139
ONT.8028.5	ENSRNOG00000029386;ENSRNOG00000000795;ENSRNOG00000058490;ENSRNOG00000045924;ENSRNOG00000000777;ENSRNOG00000000798;ONT.7819;ENSRNOG00000000787;ENSRNOG00000025806;ONT.8031;ENSRNOG00000032596;ENSRNOG00000029001	RT1-N2;RT1-N3;AABR07044362.3;RT1-T24-3;RT1-S3;Gnl1;ONT.7819;AABR07044364.1;Prr3;ONT.8031;RT1-T24-1;RT1-S2
ONT.4403.1	ENSRNOG00000012534;ENSRNOG00000012147	Mfsd10;Nop14
ONT.5110.2	ENSRNOG00000015495;ENSRNOG00000016321	Slc25a37;Entpd4
ONT.5347.1	ENSRNOG00000046342	LOC100359503
ONT.10794.1	ENSRNOG00000026592;ENSRNOG00000017426	Rhbdl2;Rragc
ONT.5880.5	ENSRNOG00000033625;ENSRNOG00000023688	AABR07027015.1;Drd1
ONT.13539.1	ENSRNOG00000003991;ENSRNOG00000004001;ENSRNOG0000004	Ankar;Osgepl1;Ormdl1
ONT.4264.1	ONT.4265;ENSRNOG00000049895;ENSRNOG00000023937;ENSRNOG00000048308;ENSRNOG00000000062;ONT.4501;ENSRNOG000000	ONT.4265;Pigg;Slc49a3;Dr1;Pcgf3;ONT.4501;Atp5me
ONT.10284.1	ENSRNOG00000037620;ENSRNOG00000010381;ENSRNOG00000010162;ENSRNOG00000037618;ENSRNOG00000010091	Mob3c;Mknk1;Tex38;Kncn;Efcab14
ONT.13148.11	ONT.13147;ENSRNOG00000008595;ENSRNOG00000025037	ONT.13147;Ttc12;Ankk1

ONT.7752.1	ENSRNOG00000013409;ENSRNOG00000025025	Gclm;Dnttip2
ONT.3947.2	ENSRNOG00000002926;ENSRNOG00000002941;ENSRNOG0000003	Uap1;Uhmk1;Sh2d1b2
ONT.11697.2	ENSRNOG00000004968	Ncapg2
ONT.12663.14	ONT.13095	ONT.13095
ONT.2774.2	ENSRNOG00000012705;ONT.2190;ONT.2185;ONT.2189;ENSRNOG00000052730;ENSRNOG00000004107;ONT.2182;ENSRNOG00000053285;ENSRNOG00000036894;ENSRNOG00000013030;ONT.2188;ENSRNOG00000004132;ENSRNOG00000036888;ONT.2772;ONT.2187	Pcgf2;ONT.2190;ONT.2185;ONT.2189;Psmb3;Rpl23;ONT.2182;Mllt6;Cisd3;Pip4k2b;ONT.2188;Lasp1;LOC691189;ONT.2772;ONT.2187
ONT.2784.1	ENSRNOG00000006450;ENSRNOG00000046057;ENSRNOG00000042044;ENSRNOG00000060511;ONT.2783;ENSRNOG00000028430;ENSRNOG00000028404;ENSRNOG00000046143;ENSRNOG00000007227	Erbb2;Pnmt;Stard3;AABR07030443.2;ONT.2783;LOC257650;Ppp1r1b;Pgap3;Mien1
ONT.571.1	ENSRNOG00000017137;ONT.570;ENSRNOG00000017222	Eef1akmt2;ONT.570;Abraxas2
ONT.7140.4	ONT.7139;ENSRNOG00000037552;ENSRNOG00000031778;ENSRNOG00000019090;ENSRNOG0000001	ONT.7139;Tsacc;Mef2d;Cct3;Rhbg
ONT.2100.2	ENSRNOG00000057092;ENSRNOG00000009709	Slfn4;AC118772.1
ONT.6686.1	ENSRNOG00000014124;ONT.6684;ENSRNOG00000014202;ONT.668	Nod2;ONT.6684;Snx20;ONT.6685
ONT.13698.1	ENSRNOG00000019295	Rab12
ONT.4740.2	ENSRNOG00000003901;ENSRNOG00000003712	Cfap36;Ppp4r3b
ONT.7094.3	ENSRNOG00000032715	AABR07010705.1
ONT.3369.1	ENSRNOG00000001083;ENSRNOG00000051699;ENSRNOG00000001079;ENSRNOG00000030927	Kdelr2;Zdhhc4;Daglb;Grid2ip
ONT.6043.1	ENSRNOG00000024066;ONT.6042;ENSRNOG00000024071;ENSRNOG00000031197	Fundc2;ONT.6042;Mtcp1;F8
ONT.6840.2	ENSRNOG00000015397;ONT.6841;ENSRNOG00000015150;ONT.6612;ENSRNOG00000015335;ENSRNOG00000015695	Cpne7;ONT.6841;Spg7;ONT.6612;Rpl13;Sult5a1
ONT.6513.1	ENSRNOG00000012167;ENSRNOG00000033487	Pou4f2;AABR07043626.1
ONT.1072.1	ONT.227;ENSRNOG00000018408;ONT.1073;ENSRNOG00000037690;ENSRNOG00000024410;ENSRNOG00000018390;ENSRNOG0000002	ONT.227;RGD1307554;ONT.1073;Sertad3;Blvrb;Pld3;Sertad1

ONT.7454.1	ENSRNOG00000018662;ENSRNOG00000018570;ENSRNOG00000023126;ENSRNOG00000018759	Amacr;C1qtnf3;Rxfp3;Slc45a2
ONT.1456.1	ENSRNOG00000017538	Lrrc27
ONT.9151.1	ENSRNOG00000016636;ENSRNOG00000051490;ENSRNOG00000027988;ONT.9150;ENSRNOG00000016734;ONT.8631	Lpin3;Plcg1;Zhx3;ONT.9150;Emilin3;ONT.8631
ONT.267.1	ENSRNOG00000020781;ENSRNOG00000050015;ENSRNOG00000050560;ENSRNOG00000048407;ENSRNOG00000049708;ENSRNOG00000024880;ENSRNOG00000045747	Tbcb;Cox7a1;Polr2i;LOC100912070;NEWGENE_1306714;Ovol3;Capns1
ONT.8051.2	ENSRNOG00000047966;ENSRNOG00000048725;ENSRNOG00000000723;ONT.8056;ENSRNOG000000	Hspa1l;Lsm2;RT1-CE5;ONT.8056;RT1-CE3
ONT.10995.7	ONT.11374;ENSRNOG00000049335;ENSRNOG00000005279;ENSRNOG00000048315	ONT.11374;Sult6b1;Ndufaf7;Eif2ak2
ONT.13103.1	ONT.13102;ONT.13101	ONT.13102;ONT.13101
ONT.5717.1	ENSRNOG00000017040;ENSRNOG00000016581;ENSRNOG0000001	Wrnip1;Serpinb1a;Serpinb6b
ONT.5912.2	ONT.5694;ENSRNOG00000014019	ONT.5694;Tbc1d7
ONT.8113.2	ENSRNOG00000028356;ENSRNOG00000028344;ONT.7923;ENSRNOG00000029979;ONT.8115;ENSRNOG00000028302;ONT.8112;ENSRNOG00000028243;ENSRNOG00000028441;ENSRNOG00000028386;ENSRNOG00000028394;ENSRNO	LOC103694872;Mmp11;ONT.7923;LOC103694884;ONT.8115;LOC103694876;ONT.8112;LOC103694875;LOC100909869;LOC103694873;Vpreb3;LOC103694877
ONT.12989.24	ENSRNOG00000026759;ENSRNOG00000018645;ENSRNOG0000001	Ccr8;Rpsa;Slc25a38
ONT.7704.1	ENSRNOG00000018236;ENSRNOG00000018186;ENSRNOG00000018201;ENSRNOG00000050416;ENSRNOG00000018285	Cym;Lamtor5;Prok1;Kcna10;Kcna2
ONT.3309.2	ENSRNOG00000001880;ENSRNOG00000001839;ENSRNOG00000000281;ENSRNOG00000030920	Dgcr6;Vpreb2;Prodh1;Rtn4r
ONT.13435.6	ENSRNOG00000053989;ENSRNOG00000050748;ENSRNOG00000045998;ENSRNOG00000049142;ENSRNOG00000047860;ENSRNOG00000045999;ENSRNOG00000050646;ENSRNOG00000047046;ENSRNO	AABR07066529.2;Dpp9;Sema6b;Hdgfl2;Plin5;Tnfaip8l1;Fem1a;Plin4;Lrg1
ONT.5636.1	ENSRNOG00000012189;ENSRNOG00000012216;ENSRNOG0000001	Lect2;Tgfbi;Fbxl21

ONT.1534.3	ENSRNOG00000020881;ONT.1536;ONT.1535;ENSRNOG00000056038;ENSRNOG00000024239;ENSRNOG00000023668;ENSRNOG00000030616;ENSRNOG00000012736;ENSRNOG00000023318;ENSRNOG00	Frmd8;ONT.1536;ONT.1535;Ehbp1l1;Fam89b;Scyl1;Slc25a45;Sssca1;Tigd3;Ltbp3
ONT.11295.1	ONT.11294;ENSRNOG00000052549;ONT.11293	ONT.11294;Chga;ONT.11293
ONT.645.1	ENSRNOG00000020455;ENSRNOG00000020551;ENSRNOG00000020587;ENSRNOG00000020460;ENSRNOG00000027564;ONT.1525;ENSRNOG00000020552;ENSRNOG00000020475;ENSRNOG00000056208;ENSRNOG00000020617;ENSRNOG00000020567;ENSRNOG00000020527;ENSRNOG00000027096;ENS	Cst6;Bles03;Efemp2;Banf1;Tsga10ip;ONT.1525;Fosl1;Sart1;Catsper1;Mus81;Fibp;Drap1;Ctsw;Eif1ad
ONT.2677.1	ONT.2676	ONT.2676
ONT.890.4	ENSRNOG00000047821	Smlr1
ONT.6442.1	ENSRNOG00000019004;ENSRNOG00000019020;ONT.6673;ONT.644	Mt4;Bbs2;ONT.6673;ONT.6443
ONT.2975.1	ENSRNOG00000060979	Hspa13
ONT.1947.1	ONT.1948;ENSRNOG00000003086;ENSRNOG00000047907;ONT.1945;ENSRNOG00000045683;ENSRNOG00000002462;ENSRNOG00000055994;ENSRNOG00000046598;ENSRNOG00000027286;ENSRNOG00	ONT.1948;Cenpv;LOC100912585;ONT.1945;LOC102553715;LOC108348055;AABR07029741.1;AABR07029742.2;Lrrc75a;Trpv2
ONT.5603.4	ENSRNOG00000025222;ENSRNOG00000019504;ENSRNOG00000062013;ONT.5602;ENSRNOG00000061995;ENSRNOG00000019649	Pcid2;AABR07026536.1;Adprhl1;ONT.5602;Grtp1;Cul4a
ONT.2260.2	ENSRNOG00000007433;ONT.2261	Cyb561;ONT.2261
ONT.13416.13	ONT.13417	ONT.13417
ONT.3156.3	ENSRNOG00000001554	RGD1563888
ONT.4891.1	ENSRNOG00000047258;ENSRNOG00000047637;ENSRNOG0000004	Prss55;Prss51;LOC683422
ONT.5245.1	ENSRNOG00000060701;ENSRNOG00000016186;ONT.5460;ENSRNOG00000052038;ENSRNOG000000	AC130232.2;Zfp709;ONT.5460;Haus8;Zfp617
ONT.2769.2	ENSRNOG00000055533;ENSRNOG00000022771;ONT.2179	AABR07030435.1;Arhgap23;ONT.2179
ONT.1655.1	ENSRNOG00000056069;ENSRNOG00000016595	Kif11;Hhex
ONT.969.1	ENSRNOG00000061533	AABR07001926.3

ONT.4707.1	ENSRNOG00000060825;ONT.4453;ONT.4704;ONT.4706;ENSRNOG00000061438;ENSRNOG00000056150;ONT.4454;ENSRNOG00000059140;ENSRNOG00000052477;ENSRNOG00000055684;ENSRNOG0000	Ccm2;ONT.4453;ONT.4704;ONT.4706;LOC100360491;Purb;ONT.4454;Myo1g;Tbrg4;Wap;Nacad
ONT.4337.10	ENSRNOG00000002205;ONT.4582	Ociad1;ONT.4582
ONT.1655.2	ENSRNOG00000016595;ENSRNOG00000056069	Hhex;Kif11
ONT.6324.2	ENSRNOG00000019276	Dele1
ONT.6043.8	ENSRNOG00000031197;ENSRNOG00000056435;ENSRNOG00000024071;ONT.6042;ENSRNOG000000	F8;Cmc4;Mtcp1;ONT.6042;Fundc2
ONT.14248.9	ENSRNOG00000048516	Fam156b
ONT.1534.5	ENSRNOG00000023668;ENSRNOG00000024239;ONT.1535;ENSRNOG00000056038;ONT.1536;ENSRNOG00000020881;ENSRNOG00000020813;ENSRNOG00000023318;ENSRNOG00000030616;ENSRNOG	Scyl1;Fam89b;ONT.1535;Ehbp1l1;ONT.1536;Frmd8;Ltbp3;Tigd3;Slc25a45;Sssca1
ONT.9480.2	ONT.9481;ONT.9876;ENSRNOG00000017380;ONT.9877	ONT.9481;ONT.9876;RGD1306746;ONT.9877
ONT.6102.1	ENSRNOG00000022009;ENSRNOG00000061695;ENSRNOG00000019875;ONT.6104;ENSRNOG00000019976;ENSRNOG00000039596;ENSRNOG00000019934	Mzb1;Slc23a1;Matr3;ONT.6104;Spata24;Prob1;Paip2
ONT.3090.4	ENSRNOG00000001727;ENSRNOG00000001728	Lsg1;Fam43a
ONT.6216.1	ENSRNOG00000015806	AABR07032457.1
ONT.3648.5	ONT.3649;ONT.3647;ENSRNOG00000060153	ONT.3649;ONT.3647;Spry3
ONT.232.1	ENSRNOG00000053366;ENSRNOG00000018936;ENSRNOG00000054618;ENSRNOG00000018994;ENSRNOG00000054568	AABR07002784.1;Zfp780b-ps1;AABR07002783.1;Psmc4;AABR07002782.1
ONT.2150.1	ENSRNOG00000002474;ONT.2149;ONT.2148	Tom1l1;ONT.2149;ONT.2148
ONT.6464.11	ONT.6465;ENSRNOG00000016245	ONT.6465;Neto2
ONT.10000.3	ENSRNOG00000052486;ONT.9999	Kcna6;ONT.9999
ONT.1947.3	ENSRNOG00000055994;ENSRNOG00000046598;ENSRNOG00000003104;ENSRNOG00000027286;ENSRNOG00000047907;ONT.1948;ENSRNOG00000003086;ENSRNOG00000045683;ENSRNOG00000002462	AABR07029741.1;AABR07029742.2;Trpv2;Lrrc75a;LOC100912585;ONT.1948;Cenpv;LOC102553715;LOC108348055;ONT.1945
ONT.8834.4	ENSRNOG00000030143;ONT.8832	AABR07051796.1;ONT.8832
ONT.4463.7	ONT.4464	ONT.4464

ONT.4185.4	ENSRNOG00000032378;ENSRNOG00000006930;ENSRNOG00000057116;ENSRNOG00000007290	Atp1a4;Casq1;LOC102548286;Atp1a2
ONT.13494.7	ENSRNOG00000012718	Zfp451
ONT.5046.1	ENSRNOG00000019162;ENSRNOG00000018536;ENSRNOG00000019438;ENSRNOG00000018239;ENSRNOG00000018528;ENSRNOG00000019041;ENSRNOG00000019246;ENSRNOG00000018825;ONT.5047;ENSRNOG00000019019	Emc9;Pck2;Rnf31;Dhrs4;Nrl;Psme1;Psme2;Dcaf11;ONT.5047;Fitm1
ONT.11020.1	ONT.11396	ONT.11396
ONT.1151.2	ONT.285;ENSRNOG00000019318;ENSRNOG00000037298;ENSRNOG00000019627;ENSRNOG00000019532;ENSRNOG00000031739;ENSRNOG00000019442;ONT.1149;ENSRNOG00000019424;ONT.1148;ENSRNOG00000019418	ONT.285;Syt3;RGD1309036;Mybpc2;Emc10;Fam71e1;Josd2;ONT.1149;Aspdh;ONT.1148;Lrrc4b
ONT.6464.10	ONT.6465;ENSRNOG00000016245	ONT.6465;Neto2
ONT.1797.1	ENSRNOG00000008843;ENSRNOG00000009536;ENSRNOG00000003195;ENSRNOG00000042352;ENSRNOG00000009667;ENSRNOG00000009611;ENSRNOG00000009224;ENSRNOG00000010771;ENSRNO	Eci1;Pgp;Caskin1;Dnase1l2;Mlst8;Bricd5;E4f1;Pkd1;Traf7
ONT.7477.1	ENSRNOG00000011963	Tas2r119
ONT.6370.1	ENSRNOG00000061507;ENSRNOG00000017181;ONT.6193	AABR07032328.1;Malt1;ONT.6193
ONT.14308.3	ENSRNOG00000002662;ENSRNOG00000002660;ONT.14309	Pbdc1;Magee1;ONT.14309
ONT.686.4	ENSRNOG00000018487;ENSRNOG00000019419;ENSRNOG00000042878;ENSRNOG00000019461;ENSRNOG00000019069;ENSRNOG00000018847;ENSRNOG00000019464;ENSRNOG00000019266;ENSRNOG00000018385;ENSRNOG00000029315;ENSRNOG00000018215;ENSRNOG00000019341;ENSRNOG00000048966;ENSRNOG00000019439	Slc3a2;Taf6l;Wdr74;Zbtb3;Nxf1;Stx5;Ttc9c;Tmem223;Chrm1;AC099294.1;Slc22a6;Tmem179b;1700092M07Rik;Polr2g
ONT.12463.3	ENSRNOG00000024170;ONT.12461;ENSRNOG00000024177;ENSRNOG00000005358;ENSRNOG00000005572;ENSRNOG00000050500;ENSRNOG00000004471;ONT.12015;ENSRNOG00000005332;ENSRNOG00000024128;ENSRNOG000000055	Phf5a;ONT.12461;AC096601.1;Pml;RGD1306782;Tob2;Polr3h;ONT.12015;Csdc2;Aco2;Desi1

ONT.10878.1	ENSRNOG00000011910;ENSRNOG00000049975;ENSRNOG00000011794;ENSRNOG00000049714	Hnrnpr;Zfp46;Tcea3;Asap3
ONT.2520.3	ENSRNOG00000013118;ONT.2519;ONT.1894	Atox1;ONT.2519;ONT.1894
ONT.9353.2	ENSRNOG00000027055;ONT.9759;ONT.9352	LOC689042;ONT.9759;ONT.9352
ONT.897.1	ENSRNOG00000016371;ENSRNOG00000016411	Slc18b1;Rps12
ONT.4785.14	ENSRNOG00000007320;ENSRNOG00000007817;ENSRNOG0000000	Fam3d;Kctd6;Acox2
ONT.1151.1	ONT.1149;ENSRNOG00000019418;ONT.1148;ENSRNOG00000019424;ENSRNOG00000019318;ONT.285;ENSRNOG00000019442;ENSRNOG00000031739;ENSRNOG00000019532;ENSRNOG00000037298;ENSRNOG00000019627	ONT.1149;Lrrc4b;ONT.1148;Aspdh;Syt3;ONT.285;Josd2;Fam71e1;Emc10;RGD1309036;Mybpc2
ONT.3450.1	ENSRNOG00000000922;ENSRNOG00000000925;ONT.3453;ENSRNOG00000000920;ENSRNOG000000	Sumf2;Psph;ONT.3453;Phkg1;Cct6a
ONT.7094.1	ENSRNOG00000032715	AABR07010705.1
ONT.3179.2	ONT.2998;ONT.3178;ONT.3177	ONT.2998;ONT.3178;ONT.3177
ONT.8775.2	ENSRNOG00000046655;ENSRNOG00000050262;ENSRNOG00000057545;ENSRNOG00000026636;ENSRNOG00000043189;ONT.8774;ENSRNOG00000026604;ENSRNOG00000026691;ENSRNOG00000014369;ENSRNOG00000014584;ONT.824	AABR07051395.1;LOC102547011;AC114363.1;Urm1;Trub2;ONT.8774;Cercam;Coq4;Slc27a4;Odf2;ONT.8242
ONT.5878.1	ENSRNOG00000000104	Thoc3
ONT.1327.1	ENSRNOG00000017387;ENSRNOG00000030818;ENSRNOG00000017408;ENSRNOG00000048852;ENSRNOG00000025819;ENSRNOG00000017914;ENSRNOG00000049480	Olr201;RGD1561034;Fam160a2;Olr200;Olr202;Cavin3;LOC686660
ONT.7708.13	ENSRNOG00000060988	Kcnc4
ONT.10082.5	ENSRNOG00000008871;ENSRNOG00000008567;ENSRNOG00000012088;ONT.10081;ENSRNOG00000	Sdr16c5;Mos;LOC100364265;ONT.10081;Plag1
ONT.5820.1	ENSRNOG00000033076;ENSRNOG00000005017;ENSRNOG0000001	Thnsl1;AABR07028839.1;Enkur
ONT.1753.1	ONT.2368;ENSRNOG00000048555	ONT.2368;AC103514.1
ONT.10133.1	ENSRNOG00000006384;ENSRNOG00000024539;ENSRNOG0000000	Ddx58;Ndufb6;Topors
ONT.1571.1	ONT.1570;ENSRNOG00000018487;ENSRNOG00000018385;ENSRNOG00000018086	ONT.1570;Slc3a2;Chrm1;Slc22a8

ONT.7140.9	ONT.7139;ENSRNOG00000031778;ENSRNOG00000037552;ENSRNOG00000019412;ENSRNOG0000001	ONT.7139;Mef2d;Tsacc;Rhbg;Cct3
ONT.3459.17	ONT.3714;ENSRNOG00000000979;ENSRNOG00000022171	ONT.3714;Bri3bp;Dhx37
ONT.11235.1	ONT.11583;ENSRNOG00000006399;ENSRNOG00000006976;ENSRNOG00000047115;ONT.11234	ONT.11583;Synj2bp;Med6;Cox16;ONT.11234
ONT.8282.1	ENSRNOG00000032700;ENSRNOG00000017017	Zbtb34;Zbtb43
ONT.12558.1	ENSRNOG00000019550;ENSRNOG00000046069;ENSRNOG00000032395;ENSRNOG00000019653	Slc11a2;Higd1c;Tfcp2;Csrnp2
ONT.7936.1	ENSRNOG00000049063	AABR07044759.1
ONT.8372.13	ONT.8373;ONT.8916;ENSRNOG00000058982	ONT.8373;ONT.8916;AABR07052729.1
ONT.10405.1	ONT.10406;ENSRNOG00000022288;ENSRNOG00000016543;ENSRNOG00000016810;ENSRNOG00000016536;ENSRNOG00000054142	ONT.10406;Pafah2;Trim63;Stmn1;Pdik1l;Slc30a2
ONT.10830.2	ENSRNOG00000024788;ENSRNOG00000012879;ENSRNOG00000013179;ENSRNOG00000012989	RGD1562036;Fabp3;Tinagl1;Serinc2
ONT.3459.11	ENSRNOG00000000979;ONT.3714;ENSRNOG00000022171	Bri3bp;ONT.3714;Dhx37
ONT.14128.8	ENSRNOG00000003191;ENSRNOG00000037658;ENSRNOG00000003187;ENSRNOG00000043306;ENSRNOG00000053780	Bhlhb9;Gprasp2;Armcx5;AABR07040617.1;LOC100361139
ONT.10045.1	ENSRNOG00000009338;ENSRNOG00000015848;ONT.10044	Kras;Etfrf1;ONT.10044
ONT.583.4	ENSRNOG00000016935;ONT.582;ENSRNOG00000017243;ENSRNOG00000016940	Mapk1ip1;ONT.582;Bnip3;Ppp2r2d
ONT.2170.1	ONT.2172;ENSRNOG00000009225;ENSRNOG00000008830;ONT.2171;ENSRNOG00000008642	ONT.2172;Copz2;Nfe2l1;ONT.2171;Snx11
ONT.3659.2	ENSRNOG00000001412;ENSRNOG00000001410;ENSRNOG00000001408;ENSRNOG00000001409;ENSRNOG00000025310	Epo;Gigyf1;Actl6b;Gnb2;Pop7
ONT.2768.1	ENSRNOG00000059008;ENSRNOG00000029245;ENSRNOG00000023095;ONT.2178;ENSRNOG000000	Socs7;Gpr179;Npepps;ONT.2178;Mrpl45
ONT.6654.8	ENSRNOG00000012271;ENSRNOG00000012447;ONT.6430;ENSRNOG00000011863	Cnot1;Setd6;ONT.6430;Gins3
ONT.13217.11	ENSRNOG00000059963;ENSRNOG00000037352;ENSRNOG0000005	AABR07070416.3;RGD1562747;AABR07070416.2

ONT.686.3	ENSRNOG00000019464;ENSRNOG00000019419;ENSRNOG00000019461;ENSRNOG00000019069;ENSRNOG00000042878;ENSRNOG00000018487;ENSRNOG00000018847;ENSRNOG00000019439;ENSRNOG00000029315;ENSRNOG00000018215;ENSRNOG00000019341;ENSRNOG00000048966;ENSRNOG00000018385;ENSRNOG00000019266	Ttc9c;Taf6l;Zbtb3;Nxf1;Wdr74;Slc3a2;Stx5;Polr2g;AC099294.1;Slc22a6;Tmem179b;1700092M07Rik;Chrm1;Tmem223
ONT.14320.1	ENSRNOG00000045666;ENSRNOG00000002789	AABR07039651.1;AABR07039648.1
ONT.2323.2	ONT.2326;ENSRNOG00000002722;ONT.2324	ONT.2326;Sec14l1;ONT.2324
ONT.12564.1	ENSRNOG00000007756;ENSRNOG00000007607;ENSRNOG00000028713;ENSRNOG00000036872	Atg101;Nr4a1;Acvrl1;AC119007.1
ONT.4323.1	ENSRNOG00000057494	AABR07014739.1
ONT.11057.1	ENSRNOG00000008812;ENSRNOG00000008605;ENSRNOG00000008612;ONT.11056	Tmem214;Ost4;Agbl5;ONT.11056
ONT.12663.10	ONT.13095	ONT.13095
ONT.955.8	ENSRNOG00000024172;ENSRNOG00000024149;ENSRNOG00000012964;ENSRNOG00000012415	T2;Prr18;Ccr6;Mpc1
ONT.3610.2	ENSRNOG00000001006	Nptx2
ONT.6102.2	ENSRNOG00000061695;ENSRNOG00000022009;ENSRNOG00000019976;ONT.6104;ENSRNOG00000019875;ENSRNOG00000019934;ENSRNOG00000039596	Slc23a1;Mzb1;Spata24;ONT.6104;Matr3;Paip2;Prob1
ONT.12548.3	ENSRNOG00000059545;ONT.12071;ENSRNOG00000053675;ENSRNOG00000061607;ENSRNOG000000	AC114446.1;ONT.12071;Dhh;Lmbr1l;Rhebl1
ONT.7138.3	ENSRNOG00000018870;ENSRNOG00000018798;ENSRNOG00000022101;ENSRNOG00000018681;ENSRNOG00000019201;ENSRNOG00000028765;ONT.7602;ENSRNOG00	Hapln2;Bcan;Crabp2;Nes;Naxe;AABR07072748.1;ONT.7602;Iqgap3
ONT.11928.1	ENSRNOG00000055178;ENSRNOG00000053535;ONT.11929;ENSRNOG00000004744	AABR07058124.4;AABR07058124.3;ONT.11929;Fam84b
ONT.8054.1	ONT.8056;ENSRNOG00000000839;ENSRNOG00000031607;ENSRNOG00000000840;ENSRNOG00000000836;ENSRNOG00000000837;ENSRNOG00000050647;ENSRNOG00000000723;ENSRNOG00000000838;ENSRNOG00000048725;ENSRNO	ONT.8056;Nfkbil1;RT1-CE3;Atp6v1g2;Ltb;LOC103694380;LOC108348108;RT1-CE5;Lta;Lsm2;Hspa1l

ONT.12564.3	ENSRNOG00000036872;ENSRNOG00000028713;ENSRNOG00000007756;ENSRNOG00000007607;ENSRNOG00000006934	AC119007.1;Acvrl1;Atg101;Nr4a1;Acvr1b
ONT.9561.1	ONT.9562;ENSRNOG00000023065;ENSRNOG00000014562;ONT.9563;ENSRNOG00000014578	ONT.9562;Zfp637;Hnrnpf;ONT.9563;Fxyd4
ONT.8621.1	ENSRNOG00000015359;ENSRNOG00000058375;ENSRNOG00000023989;ENSRNOG00000015393;ONT	Arhgap40;LOC100911217;Actr5;Slc32a1;ONT.8623
ONT.1405.1	ENSRNOG00000020010;ENSRNOG00000036713;ENSRNOG00000019960;ENSRNOG00000019920;ENSRNOG00000029061;ENSRNOG00000027213;ENSRNOG00000052802;ENSRNOG00000027098;ENSRNOG00000024144;ENSRNOG00000019914;ONT.527;ENSRNOG0000001	Kctd13;RGD1563217;Ino80e;Doc2a;Hirip3;Asphd1;Aldoa;Sez6l2;Cdipt;Fam57b;ONT.527;Taok2
ONT.1374.2	ENSRNOG00000036742;ENSRNOG00000036743;ENSRNOG0000001	Uqcrc2;AABR07005632.1;Pdzd9
ONT.5130.4	ENSRNOG00000050996;ONT.5129;ENSRNOG00000001049;ONT.492	Kctd4;ONT.5129;Tpt1;ONT.4921
ONT.8054.6	ENSRNOG00000000837;ENSRNOG00000000840;ENSRNOG00000000836;ENSRNOG00000000839;ONT.8056;ENSRNOG00000031607;ENSRNOG00000050647;ENSRNOG00000000838;ENSRNOG00000000723;ENSRNOG00000047966;ENSRNO	LOC103694380;Atp6v1g2;Ltb;Nfkbil1;ONT.8056;RT1-CE3;LOC108348108;Lta;RT1-CE5;Hspa1l;Lsm2
ONT.8140.1	ENSRNOG00000051063	AABR07044940.1
ONT.1003.1	ENSRNOG00000053828;ENSRNOG00000058906;ENSRNOG00000038600;ENSRNOG00000018250;ENSRNOG00000038607;ENSRNOG00000027942;ENSRNOG00000028041	Ppp1r12c;Ptprh;Dnaaf3;Tnni3;Tmem86b;Eps8l1;Tnnt1
ONT.7140.8	ENSRNOG00000031778;ENSRNOG00000037552;ENSRNOG00000019412;ENSRNOG00000019090;ONT	Mef2d;Tsacc;Rhbg;Cct3;ONT.7139
ONT.1978.2	ENSRNOG00000008480;ENSRNOG00000009823;ENSRNOG00000037627;ENSRNOG00000045742;ENSRNOG00000008270;ENSRNOG00000021540;ONT.1977;ENSRNOG00000009870;ENSRNOG00000037613	Kcnab3;Naa38;Trappc1;Cyb5d1;Cntrob;RGD1563441;ONT.1977;Tmem88;Kdm6b
ONT.4275.1	ENSRNOG00000002158	Ibsp
ONT.76.1	ONT.932	ONT.932
ONT.8282.2	ENSRNOG00000017017;ENSRNOG00000032700	Zbtb43;Zbtb34
ONT.7047.1	ONT.7049	ONT.7049

ONT.6043.6	ENSRNOG00000024066;ENSRNOG00000024071;ONT.6042;ENSRNOG00000056435;ENSRNOG000000	Fundc2;Mtcp1;ONT.6042;Cmc4;F8
ONT.13207.1	ENSRNOG00000025359	Spesp1
ONT.6043.7	ENSRNOG00000056435;ENSRNOG00000031197;ENSRNOG00000024066;ONT.6042;ENSRNOG000000	Cmc4;F8;Fundc2;ONT.6042;Mtcp1
ONT.1151.3	ENSRNOG00000019424;ONT.1148;ENSRNOG00000019418;ONT.1149;ENSRNOG00000019532;ENSRNOG00000037298;ENSRNOG00000019627;ENSRNOG00000019442;ENSRNOG00000031739;ONT.285;ENSRNOG00000019318	Aspdh;ONT.1148;Lrrc4b;ONT.1149;Emc10;RGD1309036;Mybpc2;Josd2;Fam71e1;ONT.285;Syt3
ONT.9714.1	ENSRNOG00000010905;ENSRNOG00000010822	Dlx5;AABR07059679.1
ONT.5968.1	ONT.5757;ENSRNOG00000017466	ONT.5757;Kif5b
ONT.7708.16	ENSRNOG00000060988	Kcnc4
ONT.3156.2	ENSRNOG00000001554	RGD1563888
ONT.22.1	ENSRNOG00000053347;ENSRNOG00000055142	Ccdc28a;Ect2l
ONT.11657.1	ENSRNOG00000031889;ENSRNOG00000004206	AABR07065438.1;Glrx5
ONT.11725.1	ENSRNOG00000021900;ENSRNOG00000043289	AABR07055834.1;LOC102551539
ONT.5742.1	ONT.5955;ONT.5959;ONT.5956;ENSRNOG00000057176;ENSRNOG00000055917;ONT.5958;ENSRNOG00000061218	ONT.5955;ONT.5959;ONT.5956;Olr1660;Trim27;ONT.5958;Olr1662
ONT.12564.2	ENSRNOG00000036872;ENSRNOG00000028713;ENSRNOG00000007756;ENSRNOG00000007607;ENSRNOG00000007346	AC119007.1;Acvrl1;Atg101;Nr4a1;Grasp
ONT.10134.1	ENSRNOG00000024539;ENSRNOG00000006384	Ndufb6;Ddx58
ONT.4079.2	ENSRNOG00000027702;ENSRNOG00000004424;ENSRNOG00000004193;ENSRNOG00000003973;ENSRNOG00000027593;ONT.3858;ENSRNOG00000039568;ENSRNOG00000004143;ENSRNOG00000003594	LOC289035;RGD1563962;Klhl12;Cyb5r1;Ndufv3;ONT.3858;Mgat4e;Adipor1;Tmem183a
ONT.13217.2	ENSRNOG00000050915;ENSRNOG00000037352;ENSRNOG0000005	AABR07070416.2;RGD1562747;AABR07070416.3
ONT.5585.1	ENSRNOG00000012878;ENSRNOG00000012108;ENSRNOG0000001	Atp7b;Thsd1;Alg11
ONT.4871.1	ENSRNOG00000021843;ONT.5087	AABR07072463.1;ONT.5087

ONT.14125.2	ENSRNOG00000025730;ENSRNOG00000011513;ENSRNOG00000037709;ENSRNOG00000011661;ENSRNOG00000011494;ENSRNOG00000037711;ENSRNOG00000037707	Armcx3;Gla;Armcx1;Hnrnph2;Rpl36a;LOC501618;Armcx6
ONT.5742.2	ONT.5958;ENSRNOG00000061218;ENSRNOG00000055917;ENSRNOG00000057176;ONT.5956;ONT.5959;ONT.5955	ONT.5958;Olr1662;Trim27;Olr1660;ONT.5956;ONT.5959;ONT.5955
ONT.7902.1	ENSRNOG00000001203;ENSRNOG00000001194;ENSRNOG0000000	Rrp1;Rrp1b;Cstb
ONT.4965.1	ENSRNOG00000048428;ENSRNOG00000013112;ENSRNOG0000001	LOC108348179;Dusp13;Samd8
ONT.11200.1	ONT.11201	ONT.11201
ONT.947.1	ENSRNOG00000014582;ENSRNOG00000023140;ENSRNOG00000014965;ENSRNOG00000019048;ENSRNOG00000013975;ENSRNOG00000019091;ENSRNOG00000014160	Mrpl18;Pnldc1;Mas1l;Sod2;Acat2l1;Wtap;Tcp1
ONT.1718.1	ENSRNOG00000016926;ENSRNOG00000016948;ONT.844;ONT.845	Plekhs1;Nhlrc2;ONT.844;ONT.845
ONT.3648.1	ONT.3649;ONT.3647;ENSRNOG00000060153	ONT.3649;ONT.3647;Spry3
ONT.9.1	ONT.10;ONT.864;ENSRNOG00000023549;ONT.865;ENSRNOG00000031859	ONT.10;ONT.864;Samd5;ONT.865;RGD1560633
ONT.4938.2	ENSRNOG00000024798;ENSRNOG00000024813	Bora;Mzt1
ONT.6326.1	ONT.6137;ENSRNOG00000013562;ENSRNOG00000047213;ONT.613	ONT.6137;Ndfip1;Gnpda1;ONT.6138
ONT.11141.4	ONT.11508;ENSRNOG00000047891;ENSRNOG00000031317	ONT.11508;Foxg1;AABR07064224.1
ONT.11073.1	ENSRNOG00000006046;ENSRNOG00000006062;ONT.11456	Gdf7;Hs1bp3;ONT.11456
ONT.7708.19	ENSRNOG00000060988	Kcnc4
ONT.671.5	ENSRNOG00000021108;ONT.670	Slc22a12;ONT.670
ONT.5939.2	ONT.5723	ONT.5723
ONT.4194.2	ENSRNOG00000046165;ENSRNOG00000009177;ENSRNOG0000004	Mptx1;Fcer1a;LOC681470
ONT.1883.17	ENSRNOG00000007624;ENSRNOG00000033065;ENSRNOG00000007652;ENSRNOG00000007462	Il4;Rad50;Il13;Sept8
ONT.6262.1	ONT.6263;ENSRNOG00000029370;ONT.6052;ENSRNOG0000001371	ONT.6263;Abhd3;ONT.6052;Snrpd1
ONT.13828.1	ONT.13525;ENSRNOG0000003890	ONT.13525;Pou3f3

ONT.13731.1	ENSRNOG00000021159;ENSRNOG00000048411;ENSRNOG00000045999;ENSRNOG00000050646;ENSRNOG00000050748;ENSRNOG00000053989;ENSRNOG00000048834;ENSRNOG00000046883;ENSRNO	LOC100362987;Uhrf1;Tnfaip8l1;Fem1a;Dpp9;AABR07066529.2;Plin3;Mydgf;Arrdc5
ONT.11564.1	ONT.11565;ENSRNOG00000033423;ONT.11566	ONT.11565;Ccdc196;ONT.11566
ONT.8054.2	ENSRNOG00000048725;ENSRNOG00000047966;ENSRNOG00000000723;ENSRNOG00000000838;ENSRNOG00000050647;ONT.8056;ENSRNOG00000031607;ENSRNOG00000000839;ENSRNOG00000000837;ENSRNOG00000000840;ENSRNO	Lsm2;Hspa1l;RT1-CE5;Lta;LOC108348108;ONT.8056;RT1-CE3;Nfkbil1;LOC103694380;Atp6v1g2;Ltb
ONT.3648.4	ONT.3647;ONT.3649	ONT.3647;ONT.3649
ONT.2420.2	ENSRNOG00000012213;ENSRNOG00000014641;ENSRNOG00000012620;ENSRNOG00000013370;ENSRNOG00000014179;ENSRNOG00000025117;ENSRNOG00000014672;ENSRNOG00000002997;ENSRNOG00000012390;ENSRNOG00000055314;ENSRNOG00000012434;ENSRNOG00000058984;ENSRNOG00000013429;ENSRNOG00000014568	Nthl1;Rpl3l;Syngr3;Gfer;Rps2;Noxo1;Hs3st6;Slc9a3r2;Npw;Msrb1;Zfp598;AC115181.1;Tbl3;Ndufb10
ONT.4079.3	ENSRNOG00000004193;ENSRNOG00000004424;ENSRNOG00000027702;ENSRNOG00000003594;ENSRNOG00000004143;ONT.3858;ENSRNOG00000039568;ENSRNOG00000003973;ENSRNOG00000027593	Klhl12;RGD1563962;LOC289035;Tmem183a;Adipor1;ONT.3858;Mgat4e;Cyb5r1;Ndufv3
ONT.13016.4	ENSRNOG00000005731;ENSRNOG00000010584;ONT.13017	Birc3;Tmem123;ONT.13017
ONT.5545.1	ENSRNOG00000011424	Cldn23
ONT.11332.1	ENSRNOG00000007184;ENSRNOG00000007219;ENSRNOG00000032348;ENSRNOG00000059714	RGD1305298;LOC103692716;LOC103690996;Hsp90aa1
ONT.13514.1	ONT.13515;ENSRNOG00000048571;ENSRNOG00000047233;ENSRNOG00000018593	ONT.13515;AABR07067422.1;LOC100910070;Txndc9
ONT.198.2	ENSRNOG00000024376;ONT.197;ENSRNOG00000029336;ONT.1045;ENSRNOG00000029022	Zfp111;ONT.197;Zfp180;ONT.1045;Zfp112
ONT.7594.2	ONT.7593	ONT.7593
ONT.7507.1	ONT.7509;ENSRNOG00000010263;ONT.7027;ONT.7028	ONT.7509;Cldn11;ONT.7027;ONT.7028
ONT.11457.1	ENSRNOG00000006119;ENSRNOG00000021403;ONT.11456	Slc7a15;Rhob;ONT.11456

ONT.4194.10	ENSRNOG00000009177;ENSRNOG00000049477;ENSRNOG0000004	Fcer1a;LOC681470;Mptx1
ONT.7708.3	ENSRNOG00000060988	Kcnc4
ONT.1757.1	ONT.2371	ONT.2371
ONT.11657.4	ENSRNOG00000031889;ENSRNOG00000004206	AABR07065438.1;Glrx5
ONT.3659.1	ENSRNOG00000001412;ENSRNOG00000025310;ENSRNOG00000001408;ENSRNOG00000001409;ENSRNOG00000001410	Epo;Pop7;Actl6b;Gnb2;Gigyf1
ONT.9480.1	ONT.9481;ENSRNOG00000017380;ONT.9877;ONT.9876	ONT.9481;RGD1306746;ONT.9877;ONT.9876
ONT.1569.2	ENSRNOG00000032042;ENSRNOG00000021202	RGD1560108;Rtn3
ONT.7708.11	ENSRNOG00000060988	Kcnc4
ONT.4394.1	ENSRNOG00000056162;ONT.4644;ENSRNOG00000061779;ENSRNOG00000055319;ENSRNOG00000054224;ONT.4645	AC111885.1;ONT.4644;Man2b2;Mrfap1;Bloc1s4;ONT.4645
ONT.607.1	ENSRNOG00000020044;ENSRNOG00000029394	Mob2;Dusp8
ONT.9253.1	ENSRNOG00000011667;ENSRNOG00000008017;ONT.9666;ENSRNOG00000008557;ENSRNOG00000023449;ENSRNOG00000013383;ENSRNOG00000009348;ENSRNOG00000008380;ENSRNOG00000012764	Fastk;Cdk5;ONT.9666;Abcb8;Atg9b;Tmub1;Nos3;Asic3;Agap3
ONT.1424.1	ONT.550;ENSRNOG00000019485;ENSRNOG00000018962;ENSRNOG00000018986;ENSRNOG00000050828;ENSRNOG00000026115;ENSRNOG00000018916;ENSRNOG00000019302;ENSRNOG00000030907;ENSRNOG00000026040;ENSRNOG00000039730;ENSRNOG00000055028;ENSRNOG00000019080;ENS	ONT.550;Bckdk;Ctf1;Fbxl19;Vkorc1;Zfp646;Bcl7c;Stx4;Ctf2;Prss53;Orai3;Setd1a;Hsd3b7;Zfp668
ONT.3006.35	ENSRNOG00000001681;ONT.3185	Vps26c;ONT.3185
ONT.12501.1	ENSRNOG00000006984;ENSRNOG00000055530;ENSRNOG00000031233;ENSRNOG00000031915;ENSRNOG00000031093;ENSRNOG00000007133;ENSRNOG00000060120;ENSRNOG00000006028	Mapk11;Panx2;Mapk12;Hdac10;Mov10l1;Plxnb2;Selenoo;Tubgcp6
ONT.7708.4	ENSRNOG00000060988	Kcnc4
ONT.4171.1	ENSRNOG00000034221;ENSRNOG00000003163;ENSRNOG00000024382;ENSRNOG00000003171;ONT	Cfap126;Sdhc;Fcgr3a;Mpz;ONT.4170
ONT.9465.1	ENSRNOG00000011617;ENSRNOG00000012227;ENSRNOG0000002	Dguok;Stambp;Actg2

ONT.5585.6	ENSRNOG00000012757;ENSRNOG00000012841;ENSRNOG00000048769;ENSRNOG00000012878	Nek3;Alg11;Nek5;Atp7b
ONT.6324.3	ENSRNOG00000019276	Dele1
ONT.4343.15	ONT.4344	ONT.4344
ONT.6582.2	ENSRNOG00000012030;ENSRNOG00000012238	AABR07043897.1;Clec3a
ONT.4007.2	ENSRNOG00000022144	AABR07072356.1
ONT.4436.2	ENSRNOG00000007849;ENSRNOG00000057703;ONT.4438;ENSRNOG00000059061	Zmat5;Cabp7;ONT.4438;Uqcr10
ONT.13641.1	ENSRNOG00000017053;ENSRNOG00000033747;ENSRNOG0000001	Fbxo36;Sp110;Csprs
ONT.9997.2	ENSRNOG00000019556;ONT.9599	Cd9;ONT.9599
ONT.9569.2	ONT.9571;ENSRNOG00000009742	ONT.9571;Rad52
ONT.9758.1	ONT.9351	ONT.9351
ONT.2393.1	ENSRNOG00000003648;ENSRNOG00000003654;ENSRNOG00000003542;ENSRNOG00000021872;ENSRNOG00000003401;ENSRNOG00000003497;ONT.1786;ENSRNOG00000050905;ENSRNOG00000003457;ENSRNOG00000021782;ENSRNOG00000003455;ENSRNOG00000056896;ENSRNOG00000003546	Cldn6;Cldn9;AABR07029198.1;Zfp213;AABR07029195.1;Thoc6;ONT.1786;Olr1382;Bicdl2;Zscan10;Zfp13;AABR07029198.2;Tnfrsf12a
ONT.9480.5	ONT.9877;ENSRNOG00000017380;ONT.9876;ONT.9481	ONT.9877;RGD1306746;ONT.9876;ONT.9481
ONT.14333.8	ENSRNOG00000028585;ENSRNOG00000034198;ENSRNOG00000028822;ENSRNOG00000037645	Tceal8;Tceal9;Bex3;Tceal7
ONT.11657.5	ENSRNOG00000031889;ENSRNOG00000004206	AABR07065438.1;Glrx5
ONT.4442.1	ENSRNOG00000008786;ENSRNOG00000008951;ENSRNOG00000009437;ENSRNOG00000033575;ENSRNOG00000027032;ENSRNOG00000009040;ONT.4690	Ap1b1;Rasl10a;Ewsr1;Emid1;Rhbdd3;Gas2l1;ONT.4690
ONT.4405.4	ENSRNOG00000013039;ENSRNOG00000012147	Add1;Nop14
ONT.2879.3	ENSRNOG00000003827;ONT.2880;ENSRNOG00000003969	Wipi1;ONT.2880;Fam20a
ONT.2328.1	ONT.2329;ENSRNOG00000022395;ENSRNOG00000053469;ENSRNOG00000046086	ONT.2329;Tnrc6c;AABR07030861.1;Tmc8
ONT.5681.1	ENSRNOG00000016099	Id4
ONT.5969.2	ONT.5970	ONT.5970
ONT.3156.4	ENSRNOG00000001554	RGD1563888
ONT.8082.1	ENSRNOG00000000499	Tcp11

ONT.6043.5	ENSRNOG00000024066;ONT.6042;ENSRNOG00000024071;ENSRNOG00000056435;ENSRNOG0000003	Fundc2;ONT.6042;Mtcp1;Cmc4;F8
ONT.7755.1	ENSRNOG00000015353	Prss12
ONT.5179.1	ENSRNOG00000012628;ENSRNOG00000025094	Gpr18;Gpr183
ONT.12038.1	ENSRNOG00000015750	Wnt7b
ONT.12463.2	ENSRNOG00000005572;ENSRNOG00000050500;ONT.12461;ENSRNOG00000024177;ENSRNOG00000024170;ENSRNOG00000005358;ONT.12015;ENSRNOG00000004471;ENSRNOG00000024128;ENSRNOG00000005332;ENSRNOG000000055	RGD1306782;Tob2;ONT.12461;AC096601.1;Phf5a;Pmm1;ONT.12015;Polr3h;Aco2;Csdc2;Desi1
ONT.8660.2	ENSRNOG00000008081	Ddx27
ONT.8763.1	ENSRNOG00000005900;ENSRNOG00000027742;ENSRNOG00000006142;ENSRNOG00000006641	Slc2a6;Adamtsl2;Mymk;Dbh
ONT.3827.2	ENSRNOG00000003952	AABR07020879.1
ONT.546.1	ENSRNOG00000016683;ENSRNOG00000019585;ENSRNOG00000019080;ENSRNOG00000055028;ENSRNOG00000039730;ENSRNOG00000019302;ENSRNOG00000026040;ENSRNOG00000018986;ENSRNOG00000050828;ENSRNOG00000026115;ONT.550;ENSRNOG0000001	Zfp668;Kat8;Hsd3b7;Setd1a;Orai3;Stx4;Prss53;Fbxl19;Vkorc1;Zfp646;ONT.550;Bckdk
ONT.11153.1	ENSRNOG00000004509;ENSRNOG00000004292;ENSRNOG00000005249;ONT.11515	Eapp;Sptssa;Snx6;ONT.11515
ONT.4980.1	ENSRNOG00000006143	Ngly1
ONT.11871.1	ENSRNOG00000047631;ENSRNOG00000047598;ENSRNOG00000050419;ENSRNOG00000052955;ENSRNOG00000046245;ENSRNOG00000033161;ENSRNOG00000025584;ENSRNOG00000047895;ENSRNOG00000046214;ENSRNOG00000048843;ONT.12295;ENSRNOG00000025602;ENSRNOG00000025592;ONT.11873;ENSRNOG00000025570	Eef1akmt3;Ctdsp2;Avil;AC114111.1;AABR07057423.1;RGD1565117;Agap2;Mettl1;Cyp27b1;Tsfm;ONT.12295;Cdk4;Tspan31;ONT.11873;Os9
ONT.14090.1	ENSRNOG00000033316;ENSRNOG00000003848;ENSRNOG00000003746;ONT.14293;ENSRNOG00000003812;ENSRNOG00000003707;ENSRNOG00000003954	Foxo4;Med12;Gjb1;ONT.14293;Nlgn3;Zmym3;Il2rg
ONT.8821.1	ENSRNOG00000048914;ENSRNOG00000018809;ENSRNOG00000018832;ENSRNOG00000018874	Traf1;Psmd5;RGD1564854;Phf19

ONT.375.1	ONT.376;ENSRNOG00000036981;ENSRNOG00000019278;ENSRNOG00000028113;ENSRNOG0000001	ONT.376;LOC100360933;Fsd2;Whamm;Ap3b2
ONT.86.2	ONT.84;ENSRNOG00000018007;ENSRNOG00000018012;ENSRNOG00000017889	ONT.84;Gtf2h5;Tulp4;Serac1
ONT.11141.5	ONT.11508;ENSRNOG00000047891;ENSRNOG00000031317	ONT.11508;Foxg1;AABR07064224.1
ONT.10116.3	ONT.10115;ENSRNOG0000000690	ONT.10115;Pou3f2
ONT.5511.1	ENSRNOG00000024411;ONT.5300	Cbr4;ONT.5300
ONT.7450.1	ENSRNOG00000016163	Slc1a3
ONT.9465.4	ENSRNOG00000011617;ENSRNOG00000029401;ENSRNOG0000001	Dguok;Actg2;Stambp
ONT.6370.2	ENSRNOG00000017181;ONT.6193;ENSRNOG00000061507	Malt1;ONT.6193;AABR07032328.1
ONT.361.2	ENSRNOG00000032293;ENSRNOG00000015526;ENSRNOG0000003	Polg;Rhcg;LOC691427
ONT.5130.1	ENSRNOG00000050996;ENSRNOG00000001052;ONT.4921;ENSRNOG00000001049;ONT.5129	Kctd4;Slc25a30;ONT.4921;Tpt1;ONT.5129
ONT.10073.2	ENSRNOG00000052668;ENSRNOG00000006804	Tcf24;Ppp1r42
ONT.4255.3	ENSRNOG00000005565;ENSRNOG00000006019;ENSRNOG0000000	Traf3ip3;G0s2;Lamb3
ONT.4258.1	ONT.4025;ENSRNOG00000007917;ENSRNOG00000045558	ONT.4025;Cd46;Cd34
ONT.8755.2	ENSRNOG00000019388;ENSRNOG00000056471;ENSRNOG00000056675;ENSRNOG00000019466	Egfl7;AABR07051251.1;LOC100911507;Agpat2
ONT.4264.2	ENSRNOG00000000062;ENSRNOG00000000064;ONT.4501;ENSRNOG00000049895;ONT.4265;ENSRNOG00000023937;ENSRNOG0000	Pcgf3;Atp5me;ONT.4501;Pigg;ONT.4265;Slc49a3;Dr1
ONT.14107.1	ENSRNOG00000037911;ONT.14108;ENSRNOG00000002925	LOC680227;ONT.14108;Tsx
ONT.5511.2	ONT.5300;ENSRNOG00000024411	ONT.5300;Cbr4
ONT.11985.2	ENSRNOG00000012090;ENSRNOG00000011305;ENSRNOG00000011170;ENSRNOG00000011507;ENSRNOG00000012215;ENSRNOG00000011020;ENSRNOG00000011214;ONT.12433;ENSRNOG0000002621	Slc16a8;Sox10;RGD1359634;Pick1;Baiap2l2;Eif3l;Polr2f;ONT.12433;Micall1
ONT.7340.2	ENSRNOG00000046468;ENSRNOG00000022218	Ptgfr;Ifi44
ONT.9784.2	ENSRNOG00000010005;ENSRNOG00000026616;ONT.9786;ONT.978	Adck2;Ndufb2;ONT.9786;ONT.9785
ONT.7708.15	ENSRNOG00000060988	Kcnc4
ONT.7362.2	ENSRNOG00000012770;ONT.7361;ENSRNOG00000026937;ENSRNOG00000012810	Spata9;ONT.7361;Arsk;Rfesd

ONT.11287.2	ENSRNOG00000004442	Dglucy
ONT.4101.1	ENSRNOG00000002525	Ptgs2
ONT.9480.6	ONT.9481;ONT.9876;ENSRNOG00000017380;ONT.9877	ONT.9481;ONT.9876;RGD1306746;ONT.9877
ONT.8859.1	ENSRNOG00000005600	Nr4a2
ONT.12548.14	ENSRNOG00000021438;ENSRNOG00000053675;ENSRNOG00000061607;ENSRNOG00000054385;ENSRNOG00000059545;ONT.12071;ENSRNOG00000053468	Tuba1c;Dhh;Lmbr1l;Rhebl1;AC114446.1;ONT.12071;Tuba1b
ONT.13035.1	ENSRNOG00000025937;ENSRNOG00000048394;ONT.13036;ENSRNOG00000028864;ENSRNOG00000033025;ENSRNOG00000033624	Zfp26;Zfp560;ONT.13036;Olr1193;Olr1192;Zfp426
ONT.5880.10	ENSRNOG00000033625;ENSRNOG00000023688	AABR07027015.1;Drd1
ONT.9411.9	ENSRNOG00000011175;ENSRNOG00000011814;ONT.9809;ONT.980	Hnrnpa2b1;Cbx3;ONT.9809;ONT.9808
ONT.13210.1	ENSRNOG00000038202;ONT.12776;ONT.13211;ENSRNOG00000007	Calml4;ONT.12776;ONT.13211;Fem1b
ONT.1738.1	ENSRNOG00000012034;ENSRNOG00000051496	Ces2i;AABR07007134.1
ONT.7140.2	ENSRNOG00000019090;ENSRNOG00000019412;ENSRNOG00000037552;ENSRNOG00000031778;ONT	Cct3;Rhbg;Tsacc;Mef2d;ONT.7139
ONT.8911.1	ONT.8365	ONT.8365
ONT.11928.2	ENSRNOG00000004744;ENSRNOG00000055178;ENSRNOG00000053535;ONT.11929	Fam84b;AABR07058124.4;AABR07058124.3;ONT.11929
ONT.1222.2	ENSRNOG00000014030	Synm
ONT.5982.1	ENSRNOG00000018698;ONT.5762;ONT.5981	Wac;ONT.5762;ONT.5981
ONT.9789.1	ENSRNOG00000012100;ENSRNOG00000030413;ONT.9788;ENSRNOG00000011854;ENSRNOG000000	Ssbp1;Tas2r137;ONT.9788;RGD1563986;Tas2r108
ONT.10073.3	ENSRNOG00000006804;ENSRNOG00000052668	Ppp1r42;Tcf24
ONT.2293.3	ENSRNOG00000028946;ENSRNOG00000030224	Cdc42ep4;AABR07030729.1
ONT.6092.2	ENSRNOG00000026050	Epb41l4a
ONT.9581.2	ENSRNOG00000014918;ENSRNOG00000015191;ENSRNOG0000005	Klrg1;Phc1;AC127013.1
ONT.3429.1	ENSRNOG00000001449;ONT.3676;ENSRNOG00000022483;ENSRNOG00000001450	Pom121;ONT.3676;Trim50;Nsun5
ONT.5669.1	ENSRNOG00000014130;ENSRNOG00000013773	Cks2;Secisbp2
ONT.4198.1	ONT.3973;ONT.4199	ONT.3973;ONT.4199
ONT.5783.1	ENSRNOG00000062048;ENSRNOG00000023587;ENSRNOG0000002	AC141220.4;Dhtkd1;Sec61a2

ONT.13528.1	ENSRNOG00000016446;ONT.1382	Gpr45;ONT.13829
ONT.10320.1	ENSRNOG00000014259;ENSRNOG00000013492;ENSRNOG00000014274;ONT.10322	Mycl;Cap1;Trit1;ONT.10322
ONT.4194.5	ENSRNOG00000049477;ENSRNOG00000009177;ENSRNOG0000004	LOC681470;Fcer1a;Mptx1
ONT.5146.1	ONT.4935;ONT.4934	ONT.4935;ONT.4934
ONT.4548.2	ENSRNOG00000002273;ENSRNOG00000052061;ONT.4302	Naaa;Ppef2;ONT.4302
ONT.4479.2	ENSRNOG00000009642;ENSRNOG00000009881	Cct4;Fam161a
ONT.4476.4	ENSRNOG00000005464;ENSRNOG00000042277	Lgalsl;AABR07016578.1
ONT.4479.4	ENSRNOG00000009881;ENSRNOG00000009642	Fam161a;Cct4
ONT.13715.1	ENSRNOG00000031939	AABR07066416.1
ONT.10838.1	ENSRNOG00000046922;ENSRNOG00000055344;ENSRNOG00000055426;ENSRNOG00000048288;ONT.10388;ENSRNOG00000050106;ON	Phactr4;Trnau1ap;Rab42;Taf12;ONT.10388;Rcc1;ONT.10387
ONT.5671.3	ENSRNOG00000027311;ENSRNOG00000061064	Nutm2f;AABR07027128.1
ONT.9346.3	ONT.9348;ENSRNOG00000048088;ONT.9349	ONT.9348;Mest;ONT.9349
ONT.953.1	ENSRNOG00000024149;ENSRNOG00000012415;ENSRNOG00000024172;ENSRNOG00000024140;ENSRNOG00000012229	Prr18;Mpc1;T2;Sft2d1;Tbxt
ONT.120.1	ENSRNOG00000056884;ENSRNOG00000060745;ENSRNOG00000053124;ENSRNOG00000055798;ENSRNOG00000056257	Olr386;Nlrp12;Olr11;LOC103689958;Cacng7
ONT.8084.1	ENSRNOG00000000501;ENSRNOG00000000502;ENSRNOG0000000	Zfp523;Def6;Tcp11
ONT.8028.3	ENSRNOG00000030157;ENSRNOG00000000804;ENSRNOG00000000798;ENSRNOG00000000809;ENSRNOG00000058490;ENSRNOG00000029386;ENSRNOG00000059268;ENSRNOG00000029001;ONT.8031;ENSRNOG00000025806;ENSRNOG00000000812;ENSRNOG0000000	Dhx16;Mrps18b;Gnl1;Atat1;AABR07044362.3;RT1-N2;Ppp1r10;RT1-S2;ONT.8031;Prr3;RGD1302996;Abcf1
ONT.12548.25	ENSRNOG00000053468;ONT.12071;ENSRNOG00000059545;ENSRNOG00000054385;ENSRNOG00000021438;ENSRNOG00000053675;ENSRNOG00000061607	Tuba1b;ONT.12071;AC114446.1;Rhebl1;Tuba1c;Dhh;Lmbr1l

ONT.1066.7	ENSRNOG00000001501;ENSRNOG00000020848;ENSRNOG00000001499;ENSRNOG00000032805;ENSRNOG00000037715;ENSRNOG00000020947;ENSRNOG00000028891;ENSRNOG00000013945	Snrpa;Coq8b;Mia;Cyp2f4;RGD1560854;Egln2;Cyp2t1;Itpkc
ONT.4289.1	ENSRNOG00000002258	Tmem150c
ONT.6891.1	ENSRNOG00000016580	Rps23
ONT.1424.11	ENSRNOG00000019302;ENSRNOG00000030907;ENSRNOG00000026040;ENSRNOG00000018986;ENSRNOG00000018916;ENSRNOG00000050828;ENSRNOG00000026115;ONT.550;ENSRNOG00000019485;ENSRNOG00000018962;ENSRNOG00000016683;ENSRNOG00000019080;ENSRNOG00000055028;ENS	Stx4;Ctf2;Prss53;Fbxl19;Bcl7c;Vkorc1;Zfp646;ONT.550;Bckdk;Ctf1;Zfp668;Hsd3b7;Setd1a;Orai3
ONT.1569.3	ENSRNOG00000032042;ENSRNOG00000021202	RGD1560108;Rtn3
ONT.11457.2	ENSRNOG00000006119;ENSRNOG00000021403;ONT.11456	Slc7a15;Rhob;ONT.11456
ONT.12048.6	ENSRNOG00000031233;ENSRNOG00000031915;ENSRNOG00000060120;ENSRNOG00000029095;ENSRNOG00000006028;ENSRNOG00000006984;ENSRNOG00000031093	Mapk12;Hdac10;Selenoo;Trabd;Tubgcp6;Mapk11;Mov10l1
ONT.13674.1	ENSRNOG00000032545;ENSRNOG00000030424;ENSRNOG00000037402;ENSRNOG00000047997;ENSRNOG00000046219;ENSRNOG00000016546;ENSRNOG00000037404;ENSRNOG00000046386;ENSRNO	Olr1351;Olr1350;Cops9;Olr1347;Olr1346;Olr1349;Olr1352;Olfr12;Otos
ONT.7977.1	ONT.7976;ENSRNOG00000043099;ENSRNOG00000000395;ENSRNOG00000030683;ENSRNOG0000000	ONT.7976;Ddx21;Kif1bp;Vps26a;Supv3l1
ONT.8618.8	ENSRNOG00000014532;ONT.9147;ENSRNOG00000052654	Lbp;ONT.9147;AABR07054460.2
ONT.13666.9	ONT.13667;ENSRNOG00000024878;ENSRNOG00000019926	ONT.13667;Rbm44;Ramp1
ONT.3459.9	ENSRNOG00000022171;ONT.3714;ENSRNOG00000000979	Dhx37;ONT.3714;Bri3bp
ONT.12663.11	ONT.13095	ONT.13095
ONT.1569.4	ENSRNOG00000021202;ENSRNOG00000032042	Rtn3;RGD1560108
ONT.3739.1	ENSRNOG00000001336;ENSRNOG00000048050;ENSRNOG0000004	Orai1;Tmem120b;Rhof
ONT.11141.2	ONT.11508;ENSRNOG00000047891;ENSRNOG00000031317	ONT.11508;Foxg1;AABR07064224.1

ONT.14333.9	ENSRNOG00000037645;ENSRNOG00000028822;ENSRNOG00000034198;ENSRNOG00000028585	Tceal7;Bex3;Tceal9;Tceal8
ONT.9997.1	ONT.9599;ENSRNOG00000019556	ONT.9599;Cd9
ONT.11282.1	ENSRNOG00000037891	AABR07065353.1
ONT.9226.1	ENSRNOG00000015558;ENSRNOG00000015202;ENSRNOG00000036719;ENSRNOG00000014592;ENSRNOG00000050277;ENSRNOG000	Zfp512b;Dnajc5;Samd10;Zbtb46;Uckl1;Abhd16b
ONT.9465.2	ENSRNOG00000011617;ENSRNOG00000012227;ENSRNOG0000002	Dguok;Stambp;Actg2
ONT.6043.3	ENSRNOG00000031197;ONT.6042;ENSRNOG00000024071;ENSRNOG00000024066	F8;ONT.6042;Mtcp1;Fundc2
ONT.1978.1	ENSRNOG00000008480;ENSRNOG00000009823;ENSRNOG00000037627;ENSRNOG00000045742;ENSRNOG00000008270;ENSRNOG00000021540;ONT.1977;ENSRNOG00000009870;ENSRNOG00000037613	Kcnab3;Naa38;Trappc1;Cyb5d1;Cntrob;RGD1563441;ONT.1977;Tmem88;Kdm6b
ONT.11657.2	ENSRNOG00000004206;ENSRNOG00000031889	Glrx5;AABR07065438.1
ONT.8028.2	ENSRNOG00000000787;ENSRNOG00000025806;ONT.8031;ENSRNOG00000032596;ENSRNOG00000029001;ENSRNOG00000029386;ENSRNOG00000058490;ENSRNOG00000045924;ENSRNOG00000000798	AABR07044364.1;Prr3;ONT.8031;RT1-T24-1;RT1-S2;RT1-N2;AABR07044362.3;RT1-T24-3;Gnl1
ONT.11595.3	ENSRNOG00000011376;ONT.11254;ENSRNOG00000033206;ENSRNOG00000011164;ENSRNOG000000	Bbof1;ONT.11254;Entpd5;Coq6;Lin52
ONT.6043.9	ENSRNOG00000031197;ENSRNOG00000056435;ENSRNOG00000024071;ONT.6042;ENSRNOG000000	F8;Cmc4;Mtcp1;ONT.6042;Fundc2
ONT.258.1	ENSRNOG00000024625;ENSRNOG00000055525;ENSRNOG00000020851;ENSRNOG00000020864;ENSRNOG00000025111;ONT.1109;ENSRNOG00000024497;ENSRNOG00000020929;ENSRNOG00000020849;ENSRNOG00000020957;ENSRNOG00000042465;ENSRNOG00000020941;ENSRNOG00000024331;ENSRNOG00000020845;ENSRNOG00000020873;ENSRNOG00000020922;	Proser3;Kmt2b;Aplp1;Kirrel2;Nfkbid;ONT.1109;U2af1l4;Lin37;Hcst;Igflr1;Zbtb32;Psenen;Upk1a;Tyrobp;Nphs1;Hspb6;Cox6b1
ONT.96.1	ENSRNOG00000055981	AABR07001516.1

ONT.1857.3	ENSRNOG00000048858;ENSRNOG00000031208;ENSRNOG00000048793;ENSRNOG00000046377;ENSRNOG00000046838;ENSRNOG00000046425;ENSRNOG00000050601;ENSRNOG00000048210	Olr1387;Mgat1;LOC684471;Btnl9;Olr1386;Olr1388;Olr1389;Tpcr12
ONT.243.1	ENSRNOG00000045545;ENSRNOG00000032034;ENSRNOG00000019338;ENSRNOG00000037638;ENSRNOG00000019310;ENSRNOG000	Eid2b;Supt5h;Dll3;Timm50;Eid2;Selenov
ONT.9613.1	ENSRNOG00000006628;ONT.9614;ENSRNOG00000007126;ENSRNOG00000007115	Dusp16;ONT.9614;Gpr19;Crebl2
ONT.10343.1	ENSRNOG00000009131	Zc3h12a
ONT.14333.7	ENSRNOG00000037645;ENSRNOG00000028822;ENSRNOG00000034198;ENSRNOG00000028585	Tceal7;Bex3;Tceal9;Tceal8
ONT.6326.2	ONT.6137;ENSRNOG00000013562;ONT.6138;ENSRNOG0000004721	ONT.6137;Ndfip1;ONT.6138;Gnpda1
ONT.5075.1	ENSRNOG00000011471	Fgf9
ONT.12663.13	ONT.13095	ONT.13095
ONT.4823.3	ENSRNOG00000009713;ENSRNOG00000010296;ENSRNOG00000039297;ENSRNOG00000012046;ENSRNOG00000039284;ENSRNOG00000011646;ENSRNOG00000011592;ENSRNOG00000010947	Oxa1l;Slc7a7;Mrpl52;Prmt5;Haus4;Rem2;Lrp10;Mmp14
ONT.11779.1	ENSRNOG00000042262;ENSRNOG00000047090	Olr1086;Olr1085
ONT.7876.1	ENSRNOG00000025864	LOC108348120
ONT.2100.1	ENSRNOG00000009709;ENSRNOG00000057092	AC118772.1;Slfn4
ONT.14402.1	ONT.14403	ONT.14403
ONT.686.2	ENSRNOG00000019464;ENSRNOG00000019419;ENSRNOG00000019461;ENSRNOG00000019069;ENSRNOG00000042878;ENSRNOG00000018487;ENSRNOG00000018847;ENSRNOG00000019439;ENSRNOG00000048966;ENSRNOG00000029315;ENSRNOG00000019341;ENSRNOG00000018215;ENSRNOG00000018385;ENSRNOG00000019266	Ttc9c;Taf6l;Zbtb3;Nxf1;Wdr74;Slc3a2;Stx5;Polr2g;1700092M07Rik;AC099294.1;Tmem179b;Slc22a6;Chrm1;Tmem223
ONT.8054.5	ENSRNOG00000000838;ENSRNOG00000000723;ENSRNOG00000047966;ENSRNOG00000048725;ENSRNOG00000000840;ENSRNOG00000000836;ENSRNOG00000000837;ENSRNOG00000031607;ENSRNOG00000000839;ONT.8056;ENSRN	Lta;RT1-CE5;Hspa1l;Lsm2;Atp6v1g2;Ltb;LOC103694380;RT1-CE3;Nfkbil1;ONT.8056;LOC108348108

ONT.3201.1	ONT.3202;ENSRNOG00000055281	ONT.3202;Dcbld2
ONT.13823.1	ENSRNOG00000013946;ONT.13824;ENSRNOG00000023220;ONT.13	Rnf149;ONT.13824;Cnot11;ONT.13825
ONT.4707.2	ENSRNOG00000055684;ENSRNOG00000052477;ENSRNOG00000053875;ONT.4704;ONT.4706;ONT.4453;ENSRNOG00000060825;ONT.4454;ENSRNOG00000059140;ENSRNOG00000056150;ENSRNOG0000	Wap;Tbrg4;Nacad;ONT.4704;ONT.4706;ONT.4453;Ccm2;ONT.4454;Myo1g;Purb;LOC100360491
ONT.6062.1	ENSRNOG00000045844;ENSRNOG00000016887;ENSRNOG0000003	Impact;Hrh4;LOC108349606
ONT.5146.2	ONT.4935;ONT.4934	ONT.4935;ONT.4934
ONT.4463.15	ONT.4464	ONT.4464
ONT.6105.1	ONT.6104;ENSRNOG00000019875;ONT.6308;ENSRNOG00000022009;ENSRNOG00000019995;ENSRNOG00000039596;ONT.6309;ENSRNOG00000019976;ENSRNOG00000061695;ONT.6106	ONT.6104;Matr3;ONT.6308;Mzb1;Dnajc18;Prob1;ONT.6309;Spata24;Slc23a1;ONT.6106
ONT.9465.3	ENSRNOG00000029401;ENSRNOG00000012227;ENSRNOG0000001	Actg2;Stambp;Dguok
ONT.12663.15	ONT.13095	ONT.13095
ONT.6413.1	ENSRNOG00000050034;ENSRNOG00000026262;ENSRNOG00000011330;ENSRNOG00000011635	LOC501297;AABR07042571.1;Ces2a;Ces2e
ONT.1787.3	ENSRNOG00000021782;ENSRNOG00000003457;ENSRNOG00000003455;ENSRNOG00000028231;ENSRNOG00000056896;ENSRNOG00000003546;ENSRNOG00000003648;ENSRNOG00000003657;ENSRNOG00000003654;ENSRNOG00000003721;ENSRNOG00000003497;ENSRNOG00000042258;ENSRNOG000	Zscan10;Bicdl2;Zfp13;AABR07029202.1;AABR07029198.2;Tnfrsf12a;Cldn6;Pkmyt1;Cldn9;Paqr4;Thoc6;RGD1561157;Kremen2
ONT.2925.9	ENSRNOG00000000249;ENSRNOG00000042915;ENSRNOG00000000251;ENSRNOG00000000250;ENSRNOG00000000247	Mettl23;Mxra7;St6galnac1;Jmjd6;Mfsd11
ONT.12989.22	ENSRNOG00000018552;ENSRNOG00000018645;ENSRNOG0000002	Slc25a38;Rpsa;Ccr8
ONT.4476.1	ENSRNOG00000042277	AABR07016578.1
ONT.10116.2	ENSRNOG00000006908;ONT.1011	Pou3f2;ONT.10115
ONT.8660.1	ENSRNOG00000008081	Ddx27
ONT.102.1	ENSRNOG00000058564	AABR07001592.2
ONT.10690.4	ONT.10689	ONT.10689
ONT.10930.8	ENSRNOG00000008619;ENSRNOG00000008911;ENSRNOG00000031641;ONT.10931;ENSRNOG00000009409;ONT.10460;ENSRNOG00000009217;ENSRNOG00000009134	Agtrap;Draxin;LOC108351058;ONT.10931;Fbxo2;ONT.10460;Fbxo6;Mad2l2

ONT.11266.1	ENSRNOG00000010567;ENSRNOG00000010457	Angel1;Vash1
ONT.8134.1	ONT.8133	ONT.8133
ONT.9480.4	ONT.9481;ONT.9877;ENSRNOG00000017380;ONT.9876	ONT.9481;ONT.9877;RGD1306746;ONT.9876
ONT.2740.1	ENSRNOG00000002948;ENSRNOG00000002831;ENSRNOG00000002835;ENSRNOG00000045669	Abcc3;Wfikkn2;Luc7l3;AABR07030335.1
ONT.6699.1	ENSRNOG00000003461	Zfp330
ONT.5407.1	ENSRNOG00000011636;ENSRNOG00000059442;ENSRNOG0000001	Dennd6a;Pde12;Arf4
ONT.11545.1	ONT.11543;ONT.11182;ENSRNOG00000007851;ENSRNOG00000007504;ONT.11544;ENSRNOG00000026239;ONT.11542;ONT.11181	ONT.11543;ONT.11182;Psma3l;Actr10;ONT.11544;Arid4a;ONT.11542;ONT.11181
ONT.2286.1	ENSRNOG00000004713	Kcnj16
ONT.3923.2	ONT.3921;ENSRNOG00000026907;ENSRNOG00000038789;ENSRNOG00000002783;ENSRNOG0000000	ONT.3921;Zbtb37;Cenpl;Serpinc1;Dars2
ONT.6092.1	ENSRNOG00000026050	Epb41l4a
ONT.8884.1	ONT.8885;ENSRNOG00000061587;ENSRNOG00000001519;ONT.888	ONT.8885;Metap1d;Dlx2;ONT.8886
ONT.5585.3	ENSRNOG00000012841;ENSRNOG00000012757;ENSRNOG00000012878;ENSRNOG00000048769	Alg11;Nek3;Atp7b;Nek5
ONT.7708.10	ENSRNOG00000060988	Kcnc4
ONT.3179.1	ONT.2998;ONT.3178;ONT.3177	ONT.2998;ONT.3178;ONT.3177
ONT.12663.16	ONT.13095	ONT.13095
ONT.12747.1	ENSRNOG00000030654;ENSRNOG00000038477;ENSRNOG00000018671;ENSRNOG00000018689;ENSRNOG00000032254;ENSRNOG000	Man2c1;AABR07070252.1;Commd4;RGD1305464;Sin3a;Neil1
ONT.5110.1	ENSRNOG00000016321;ENSRNOG00000015495	Entpd4;Slc25a37
ONT.7962.16	ENSRNOG00000000572;ONT.8148	Chst3;ONT.8148
ONT.10342.1	ENSRNOG00000009131	Zc3h12a
ONT.10855.3	ENSRNOG00000014665;ENSRNOG00000060320;ONT.10401	Dhdds;Lin28a;ONT.10401
ONT.13801.1	ENSRNOG00000042646;ENSRNOG00000014001;ENSRNOG00000023858;ENSRNOG00000059580	Cfc1;Prss40;Prss39;AABR07067349.1
ONT.4740.1	ENSRNOG00000003901;ENSRNOG00000003712	Cfap36;Ppp4r3b
ONT.263.1	ENSRNOG00000021015;ENSRNOG00000024000;ENSRNOG00000021023;ENSRNOG00000037467;ENSRNOG00000021006;ENSRNOG00000021009;ENSRNOG00000055934;ENSRNOG00000024009;ENSRNOG00000021021;ENSRNOG0000002	Sbsn;Cd22;Mag;Ffar3;Tmem147;Gapdhs;Dmkn;Ffar1;Ffar2;Atp4a

ONT.8406.1	ONT.8947;ENSRNOG00000000008	ONT.8947;Alx4
ONT.12882.1	ENSRNOG00000060723;ENSRNOG00000047984;ENSRNOG0000001	AABR07071244.1;Mrps22;Rbp2
ONT.8887.1	ENSRNOG00000028543	AABR07052523.1
ONT.12293.1	ENSRNOG00000045629;ENSRNOG00000047598;ENSRNOG0000005	Atp23;Ctdsp2;Avil
ONT.10715.10	ENSRNOG00000047792;ENSRNOG00000032944	AABR07049320.2;AABR07049320.1
ONT.4577.2	ENSRNOG00000057975	AABR07014882.1
ONT.9353.1	ENSRNOG00000027055;ONT.9352;ONT.9759	LOC689042;ONT.9352;ONT.9759
ONT.3728.1	ENSRNOG00000001081;ENSRNOG00000001080	Ogfod2;Arl6ip4
ONT.14187.1	ENSRNOG00000055562	Dkc1
ONT.1650.1	ENSRNOG00000018494;ENSRNOG00000056753	Ppp1r3c;Hectd2
ONT.8894.6	ENSRNOG00000018286	Chrna1
ONT.13065.1	ENSRNOG00000026979;ENSRNOG00000029456	AABR07069466.1;Rp9
ONT.2194.1	ENSRNOG00000046143;ENSRNOG00000046057;ENSRNOG00000028430;ENSRNOG00000028404;ENSRNOG00000060511;ENSRNOG000	Pgap3;Pnmt;LOC257650;Ppp1r1b;AABR07030443.2;Stard3
ONT.11057.2	ENSRNOG00000008612;ENSRNOG00000008605;ENSRNOG00000008812;ONT.11056	Agbl5;Ost4;Tmem214;ONT.11056
ONT.5130.2	ONT.4921;ENSRNOG00000001052;ONT.5129;ENSRNOG00000001049;ENSRNOG00000050996	ONT.4921;Slc25a30;ONT.5129;Tpt1;Kctd4
ONT.3179.4	ONT.3177;ONT.3178;ONT.2998	ONT.3177;ONT.3178;ONT.2998
ONT.6737.1	ENSRNOG00000055892	AABR07043395.1
ONT.10453.1	ENSRNOG00000014961;ENSRNOG00000030596	Pdpn;LOC100359951
ONT.9226.2	ENSRNOG00000015558;ENSRNOG00000015202;ENSRNOG00000014592;ENSRNOG00000036719;ENSRNOG00000015067;ENSRNOG000	Zfp512b;Dnajc5;Zbtb46;Samd10;Abhd16b;Uckl1
ONT.1306.1	ENSRNOG00000017118;ENSRNOG00000016906;ENSRNOG00000017079;ENSRNOG00000018411	P4ha3;Lipt2;Pgm2l1;Pold3
ONT.4343.14	ONT.4344	ONT.4344
ONT.8054.7	ENSRNOG00000048725;ENSRNOG00000047966;ENSRNOG00000000838;ENSRNOG00000000723;ENSRNOG00000050647;ENSRNOG00000031607;ENSRNOG00000000839;ONT.8056;ENSRNOG00000000837;ENSRNOG00000000836;ENSRNO	Lsm2;Hspa1l;Lta;RT1-CE5;LOC108348108;RT1-CE3;Nfkbil1;ONT.8056;LOC103694380;Ltb;Atp6v1g2

ONT.4823.1	ENSRNOG00000010296;ENSRNOG00000009713;ENSRNOG00000012046;ENSRNOG00000039284;ENSRNOG00000011646;ENSRNOG00000039297;ENSRNOG00000011592;ENSRNOG00000010947	Slc7a7;Oxa1l;Prmt5;Haus4;Rem2;Mrpl52;Lrp10;Mmp14
ONT.1858.2	ENSRNOG00000046377;ENSRNOG00000048858;ENSRNOG00000050601;ENSRNOG00000046425;ENSRNOG00000051756	Btnl9;Olr1387;Olr1389;Olr1388;Zfp62
ONT.11741.6	ENSRNOG00000032202;ENSRNOG00000019822;ONT.12153;ENSRNOG00000019924;ENSRNOG00000020005;ENSRNOG00000029738;ENSRNOG00000019857;ONT.12150;ENSRNOG00000019891	Creb3l3;Gadd45b;ONT.12153;Thop1;Map2k2;Diras1;Gng7;ONT.12150;Sgta
ONT.2879.2	ONT.2880;ENSRNOG00000003827;ENSRNOG00000003969	ONT.2880;Wipi1;Fam20a
ONT.11779.2	ENSRNOG00000042262;ENSRNOG00000047090	Olr1086;Olr1085
ONT.686.5	ENSRNOG00000019439;ENSRNOG00000029315;ENSRNOG00000048966;ENSRNOG00000019341;ENSRNOG00000018215;ENSRNOG00000018385;ENSRNOG00000019266;ENSRNOG00000019464;ENSRNOG00000018847;ENSRNOG00000019419;ENSRNOG00000019461;ENSRNOG00000042878;ENSRNOG00000019069;ENSRNOG00000018487	Polr2g;AC099294.1;1700092M07Rik;Tmem179b;Slc22a6;Chrm1;Tmem223;Ttc9c;Stx5;Taf6l;Zbtb3;Wdr74;Nxf1;Slc3a2
ONT.13276.2	ENSRNOG00000010699;ENSRNOG00000043223	Trim43a;Zfp949
ONT.11287.1	ENSRNOG00000004442	Dglucy
ONT.7708.6	ENSRNOG00000060988	Kcnc4
ONT.5579.1	ENSRNOG00000019150;ENSRNOG00000019267	Polb;Dkk4
ONT.3526.2	ENSRNOG00000001160;ONT.3525;ENSRNOG00000001156;ENSRNOG00000001153;ENSRNOG0000000	NEWGENE_1586233;ONT.3525;Msi1;Pla2g1b;Sirt4
ONT.5997.1	ENSRNOG00000017165;ENSRNOG00000029844	Akr1e2;Akr1c2
ONT.12879.1	ENSRNOG00000012386	Zbtb38
ONT.4436.3	ONT.4437;ENSRNOG00000057703;ENSRNOG00000007849;ENSRNOG00000059061	ONT.4437;Cabp7;Zmat5;Uqcr10
ONT.9480.3	ONT.9876;ONT.9877;ENSRNOG00000017380;ONT.9481	ONT.9876;ONT.9877;RGD1306746;ONT.9481
ONT.12453.1	ENSRNOG00000032777;ENSRNOG00000018316;ENSRNOG0000001	AABR07058519.1;Grap2;Fam83f

ONT.5953.1	ENSRNOG00000053518;ENSRNOG00000031902;ENSRNOG00000052959;ENSRNOG00000051823;ENSRNOG00000056708;ENSRNOG000	Zscan26;AABR07027819.1;Zscan12;Zkscan4;Nkapl;Zkscan3
ONT.12325.2	ENSRNOG00000029443	LOC108352650
ONT.7708.2	ENSRNOG00000060988	Kcnc4
ONT.8054.3	ENSRNOG00000000838;ENSRNOG00000000723;ENSRNOG00000047966;ENSRNOG00000048725;ENSRNOG00000000840;ENSRNOG00000000836;ENSRNOG00000000837;ENSRNOG00000000839;ENSRNOG00000031607;ONT.8056;ENSRN	Lta;RT1-CE5;Hspa1l;Lsm2;Atp6v1g2;Ltb;LOC103694380;Nfkbil1;RT1-CE3;ONT.8056;LOC108348108
ONT.1724.2	ENSRNOG00000017982;ENSRNOG00000025953;ENSRNOG0000001	Pnliprp2;LOC681006;Pnliprp1
ONT.11459.2	ONT.11456;ENSRNOG00000006119;ENSRNOG00000021403	ONT.11456;Slc7a15;Rhob
ONT.8030.1	ENSRNOG00000059268;ENSRNOG00000000818;ENSRNOG00000032596;ENSRNOG00000000812;ENSRNOG00000000816;ONT.8031;ENSRNOG00000000799;ENSRNOG00000000787;ENSRNOG00000030157;ENSRNOG00000000804;ENSRNOG00000045924;ENSRNOG00000000809;ENSRNOG00000000798	Ppp1r10;Nrm;RT1-T24-1;RGD1302996;Ppp1r18;ONT.8031;Abcf1;AABR07044364.1;Dhx16;Mrps18b;RT1-T24-3;Atat1;Gnl1
ONT.6356.1	ENSRNOG00000026060;ENSRNOG00000018735;ENSRNOG0000002	Arsi;Cd74;Tcof1
ONT.11697.1	ENSRNOG00000004968	Ncapg2
ONT.4504.1	ENSRNOG00000023533	Fam69a
ONT.4476.2	ENSRNOG00000042277	AABR07016578.1
ONT.7594.1	ONT.7593	ONT.7593
ONT.12830.1	ENSRNOG00000052499;ENSRNOG00000052787;ONT.12828;ENSRNOG00000059622;ENSRNOG00000056654;ENSRNOG00000054460;ENSRNOG00000053428;ONT.12826;ENSRNOG00000052869	Rab27a;Rsl24d1;ONT.12828;Pigb;Dnaaf4;Khdc3;Ccpg1;ONT.12826;Ccpg1os
ONT.14133.2	ENSRNOG00000034198;ENSRNOG00000028822;ENSRNOG00000058183;ENSRNOG00000037645	Tceal9;Bex3;AABR07040624.1;Tceal7
ONT.3640.2	ENSRNOG00000001312	Pdgfa
ONT.3459.12	ENSRNOG00000022171;ONT.3714;ENSRNOG00000000979	Dhx37;ONT.3714;Bri3bp
ONT.794.1	ENSRNOG00000016356;ENSRNOG00000016302;ENSRNOG0000001	Got1;Cnnm1;Nkx2-3
ONT.3718.13	ENSRNOG00000021691	Ccdc92

ONT.6757.1	ENSRNOG00000024452;ENSRNOG00000024364;ENSRNOG00000039001;ENSRNOG00000017867;ENSRNOG00000017746;ENSRNOG000	Carmil2;Enkd1;Agrp;RGD1561415;Pard6a;Acd
ONT.2879.1	ONT.2880;ENSRNOG00000003827;ENSRNOG00000003969	ONT.2880;Wipi1;Fam20a
ONT.14308.2	ENSRNOG00000002660;ENSRNOG00000002662;ONT.14309	Magee1;Pbdc1;ONT.14309
ONT.10426.6	ENSRNOG00000014445;ENSRNOG00000014909	Hp1bp3;Sh2d5
ONT.1778.1	ENSRNOG00000046980;ENSRNOG00000005227;ENSRNOG00000005269;ENSRNOG00000027368	LOC108348151;Tfap4;Srl;LOC100910875
ONT.9116.1	ENSRNOG00000061603;ONT.8586;ENSRNOG00000010361;ONT.858	Asxl1;ONT.8586;Kif3b;ONT.8585
ONT.1791.1	ENSRNOG00000007436;ENSRNOG00000007483;ONT.2405;ENSRNOG00000006970;ENSRNOG00000006542;ENSRNOG00000052204;ONT.2408;ENSRNOG00000006460	Tedc2;Ccnf;ONT.2405;Ntn3;Atp6v0c;Tbc1d24;ONT.2408;Amdhd2
ONT.3718.14	ENSRNOG00000021691	Ccdc92
ONT.3526.1	ENSRNOG00000001160;ONT.3525;ENSRNOG00000001156;ENSRNOG00000001153;ENSRNOG0000000	NEWGENE_1586233;ONT.3525;Msi1;Pla2g1b;Sirt4
ONT.12045.1	ONT.12044;ENSRNOG00000004591;ENSRNOG00000029698;ENSRNOG00000004538;ENSRNOG000000	ONT.12044;Alg12;Pim3;Brd1;Creld2
ONT.5969.1	ONT.5970	ONT.5970
ONT.13848.1	ENSRNOG00000032947;ONT.13850;ONT.13849;ONT.13551	AABR07067749.1;ONT.13850;ONT.13849;ONT.13551
ONT.7823.6	ENSRNOG00000000816;ENSRNOG00000000812;ENSRNOG00000025806;ENSRNOG00000000787;ENSRNOG00000059268;ENSRNOG00000032596;ENSRNOG00000000818;ENSRNOG00000000798;ENSRNOG00000000809;ENSRNOG00000045924;ENSRNOG00000030157;ENSRNOG00000000804;ENSRNOG000	Ppp1r18;RGD1302996;Prr3;AABR07044364.1;Ppp1r10;RT1-T24-1;Nrm;Gnl1;Atat1;RT1-T24-3;Dhx16;Mrps18b;Mdc1
ONT.5585.5	ENSRNOG00000012841;ENSRNOG00000012757;ENSRNOG00000012878;ENSRNOG00000048769	Alg11;Nek3;Atp7b;Nek5
ONT.361.1	ENSRNOG00000032293;ENSRNOG00000031802;ENSRNOG0000001	Polg;LOC691427;Rhcg
ONT.4463.4	ONT.4464	ONT.4464
ONT.5662.1	ENSRNOG00000000104	Thoc3
ONT.2293.2	ENSRNOG00000028946;ENSRNOG00000030224	Cdc42ep4;AABR07030729.1
ONT.11725.2	ENSRNOG00000021900;ENSRNOG00000043289	AABR07055834.1;LOC102551539

ONT.14133.1	ENSRNOG00000046280;ENSRNOG00000058183;ENSRNOG0000002	Tceal5;AABR07040624.1;Tceal8
ONT.3459.15	ONT.3714;ENSRNOG00000000979;ENSRNOG00000022171	ONT.3714;Bri3bp;Dhx37
ONT.994.5	ENSRNOG00000016608;ENSRNOG00000038625;ENSRNOG00000038622;ENSRNOG00000016274;ONT.992;ENSRNOG00000015753;ENSRNOG00000016565;ENSRNOG00000042414;ENSRNOG00000042927;ENSRNOG00000016279;ENSRNOG00000015743;ENSRNOG00000032913;ENSRNOG00000016687;ENSRNOG00000015914;ENSRNOG000	Zfp579;Sbk2;Zfp628;Zfp580;ONT.992;Epn1;Zfp524;Nat14;Sbk3;Zfp865;Rfpl4a;Rasl2-9;Ssc5d;U2af2;Fiz1
ONT.9452.1	ENSRNOG00000005587	Suclg1
ONT.686.1	ENSRNOG00000019439;ENSRNOG00000019341;ENSRNOG00000029315;ENSRNOG00000018215;ENSRNOG00000048966;ENSRNOG00000019266;ENSRNOG00000018385;ENSRNOG00000019464;ENSRNOG00000019419;ENSRNOG00000019461;ENSRNOG00000019069;ENSRNOG00000042878;ENSRNOG00000018487;ENSRNOG00000018847	Polr2g;Tmem179b;AC099294.1;Slc22a6;1700092M07Rik;Tmem223;Chrm1;Ttc9c;Taf6l;Zbtb3;Nxf1;Wdr74;Slc3a2;Stx5
ONT.10221.1	ENSRNOG00000049025;ENSRNOG00000007663	Rps6;LOC100911372
ONT.9067.2	ENSRNOG00000038259;ENSRNOG00000025295;ENSRNOG00000025286;ENSRNOG00000021249;ONT	AABR07053749.1;Mavs;Pank2;Ap5s1;ONT.8520
ONT.5880.6	ENSRNOG00000023688;ENSRNOG00000033625	Drd1;AABR07027015.1
ONT.7755.3	ENSRNOG00000015353	Prss12
ONT.1752.1	ENSRNOG00000002568;ENSRNOG00000002564;ENSRNOG00000021902;ENSRNOG00000002539;ENSRNOG00000002566;ENSRNOG000	Socs1;Prm3;Rmi2;Prm2;Tnp2;Prm1
ONT.7047.2	ONT.7049;ENSRNOG00000012199	ONT.7049;Sox2
ONT.14337.3	ONT.14141;ENSRNOG00000048011;ENSRNOG00000049849;ONT.14142;ENSRNOG00000037661;ENSRNOG00000045618	ONT.14141;AABR07040695.1;Fam199x;ONT.14142;Tmsb15b2;AABR07040686.1
ONT.13100.1	ENSRNOG00000050630	AABR07070043.1
ONT.3201.2	ENSRNOG00000055281;ONT.3202	Dcbld2;ONT.3202
ONT.583.2	ONT.582;ENSRNOG00000016935;ENSRNOG00000016940;ENSRNOG00000017243	ONT.582;Mapk1ip1;Ppp2r2d;Bnip3
ONT.10082.3	ENSRNOG00000012088;ONT.10081;ENSRNOG00000008846;ENSRNOG00000008567;ENSRNOG000000	LOC100364265;ONT.10081;Plag1;Mos;Sdr16c5

ONT.8761.1	ENSRNOG00000047737;ENSRNOG00000005900;ENSRNOG00000005167;ENSRNOG00000006142;ENSRNOG00000027742;ENSRNOG00000027911;ENSRNOG00000027867;ENSRNOG00000060005	Rpl7a;Slc2a6;Surf2;Mymk;Adamtsl2;Stkld1;Rexo4;Surf4
ONT.10883.1	ENSRNOG00000012807;ENSRNOG00000012804;ENSRNOG00000013036;ENSRNOG00000012749	C1qa;C1qc;Epha8;C1qb
ONT.8660.4	ENSRNOG00000008081	Ddx27
ONT.6840.3	ENSRNOG00000015150;ONT.6612;ENSRNOG00000015335;ENSRNOG00000015695;ENSRNOG00000015397;ONT.6841	Spg7;ONT.6612;Rpl13;Sult5a1;Cpne7;ONT.6841
ONT.6324.4	ENSRNOG00000019276	Dele1
ONT.7708.9	ENSRNOG00000060988	Kcnc4
ONT.4079.1	ENSRNOG00000004193;ENSRNOG00000027702;ENSRNOG00000004424;ENSRNOG00000004143;ENSRNOG00000003594;ENSRNOG00000027593;ENSRNOG00000003973;ENSRNOG00000039568;ONT.3858	Klhl12;LOC289035;RGD1563962;Adipor1;Tmem183a;Ndufv3;Cyb5r1;Mgat4e;ONT.3858
ONT.11141.1	ENSRNOG00000031317;ENSRNOG00000047891;ONT.11508	AABR07064224.1;Foxg1;ONT.11508
ONT.119.1	ENSRNOG00000057848;ENSRNOG00000055581;ONT.973;ENSRNOG00000055716;ONT.974;ONT.972;ENSRNOG00000057852	Cacng8;Tarm1;ONT.973;Oscar;ONT.974;ONT.972;Cacng6
ONT.252.2	ENSRNOG00000055519;ONT.1097	AABR07002868.1;ONT.1097
ONT.13148.15	ONT.13147;ENSRNOG00000025037;ENSRNOG00000008595	ONT.13147;Ankk1;Ttc12
ONT.3923.1	ENSRNOG00000002813;ONT.3921;ENSRNOG00000026907;ENSRNOG00000038789;ENSRNOG0000000	Dars2;ONT.3921;Zbtb37;Cenpl;Serpinc1
ONT.1576.1	ENSRNOG00000048966;ONT.693;ENSRNOG00000026005;ENSRNOG00000026302;ENSRNOG00000019069;ENSRNOG00000052393;ONT.1579;ENSRNOG00000019341;ENSRNOG00000029315;ENSRNOG00000019266;ENSRNOG00000019570;ENSRNOG00000019666;ENSRNOG00000019439;ENSRNOG00000019507;ENSRNOG00000019419;ENSRNOG00000019461;ENSRNOG00000042878;ENSRNOG00000018487;ENSRNOG00000045561;ENSRNOG00000018847;ENSRNOG0000001	1700092M07Rik;ONT.693;Ints5;Lrrn4cl;Nxf1;Bscl2;ONT.1579;Tmem179b;AC099294.1;Tmem223;Gng3;Ubxn1;Polr2g;Hnrnpul2;Taf6l;Zbtb3;Wdr74;Slc3a2;Uqcc3;Stx5;RGD1597339
ONT.3640.3	ENSRNOG00000001312	Pdgfa
ONT.3718.12	ENSRNOG00000021691	Ccdc92

ONT.11657.3	ENSRNOG00000031889;ENSRNOG00000004206	AABR07065438.1;Glrx5
ONT.13844.1	ONT.13549	ONT.13549
ONT.10795.2	ENSRNOG00000058812	AABR07049886.3
ONT.821.7	ENSRNOG00000020035;ENSRNOG00000020081	Cyp17a1;As3mt
ONT.12754.1	ONT.13185;ENSRNOG00000018816;ENSRNOG00000038459;ENSRNOG00000019136;ENSRNOG00000018898;ENSRNOG00000019374;ONT.13186;ONT.12749;ENSRNOG00000016173;ENSRNOG00000054468;ENSRNOG00000019500	ONT.13185;Cox5a;Ulk3;Scamp2;Mpi;Csk;ONT.13186;ONT.12749;Cyp1a2;Fam219b;Cyp1a1
ONT.6102.3	ENSRNOG00000039596;ENSRNOG00000019934;ENSRNOG00000019976;ONT.6104;ENSRNOG00000019875;ENSRNOG00000061695;ENSRNOG00000022009	Prob1;Paip2;Spata24;ONT.6104;Matr3;Slc23a1;Mzb1
ONT.7708.18	ENSRNOG00000060988	Kcnc4
ONT.5871.1	ENSRNOG00000018122;ONT.5653;ONT.5655;ENSRNOG0000001803	Tspan17;ONT.5653;ONT.5655;Sncb
ONT.671.3	ENSRNOG00000021108;ENSRNOG00000021098;ONT.670	Slc22a12;Rasgrp2;ONT.670
ONT.12038.2	ENSRNOG00000015750	Wnt7b
ONT.4436.4	ENSRNOG00000059061;ONT.4438;ENSRNOG00000057703;ENSRNOG00000007849	Uqcr10;ONT.4438;Cabp7;Zmat5
ONT.7140.6	ENSRNOG00000019090;ENSRNOG00000019412;ENSRNOG00000037552;ENSRNOG00000031778;ONT	Cct3;Rhbg;Tsacc;Mef2d;ONT.7139
ONT.3179.3	ONT.3178;ONT.2998;ONT.3177	ONT.3178;ONT.2998;ONT.3177
ONT.7752.2	ENSRNOG00000025025;ENSRNOG00000013409	Dnttip2;Gclm
ONT.6836.1	ENSRNOG00000015077	Acsf3
ONT.13666.8	ONT.13667;ENSRNOG00000019926;ENSRNOG00000024878	ONT.13667;Ramp1;Rbm44
ONT.9411.8	ENSRNOG00000011814;ENSRNOG00000011175;ONT.9808;ONT.980	Cbx3;Hnrnpa2b1;ONT.9808;ONT.9809
ONT.12147.1	ENSRNOG00000020005;ENSRNOG00000020407;ONT.11738;ENSRNOG00000020416;ONT.12150;ENSRNOG00000020161;ENSRNOG00000042897;ENSRNOG00000020230;ENSRNOG00000020383;ENSRNOG00000020464;ENSRNOG00000020431;ENSRNOG00000032202;ENSRNOG00000020466;ENSRNOG0000	Map2k2;Atcay;ONT.11738;Zfr2;ONT.12150;Zbtb7a;Nmrk2;Pias4;Dapk3;Mrpl54;Matk;Creb3l3;Apba3;Eef2;ONT.12148
ONT.6043.4	ENSRNOG00000024066;ONT.6042;ENSRNOG00000024071;ENSRNOG00000031197	Fundc2;ONT.6042;Mtcp1;F8

ONT.583.3	ENSRNOG00000016940;ENSRNOG00000017243;ONT.582;ENSRNOG00000016935	Ppp2r2d;Bnip3;ONT.582;Mapk1ip1
ONT.7708.17	ENSRNOG00000060988	Kcnc4
ONT.11624.1	ENSRNOG00000004060;ONT.11626;ONT.11286;ENSRNOG00000003951;ENSRNOG00000004004	Calm2;ONT.11626;ONT.11286;Psmc1;Nrde2
ONT.11545.2	ONT.11181;ONT.11542;ENSRNOG00000026239;ONT.11544;ENSRNOG00000007504;ENSRNOG00000007851;ONT.11182;ONT.11543	ONT.11181;ONT.11542;Arid4a;ONT.11544;Actr10;Psma3l;ONT.11182;ONT.11543
ONT.6092.3	ENSRNOG00000026050	Epb41l4a
ONT.821.6	ENSRNOG00000020081;ENSRNOG00000020035	As3mt;Cyp17a1
ONT.9672.1	ENSRNOG00000022182;ONT.9674;ONT.9673;ONT.9257;ENSRNOG00000043114	Rint1;ONT.9674;ONT.9673;ONT.9257;Tomm7
ONT.7406.1	ENSRNOG00000017945;ENSRNOG00000018067;ENSRNOG0000003	Serf1;Smn1;Naip2
ONT.7140.3	ONT.7139;ENSRNOG00000019090;ENSRNOG00000019412;ENSRNOG00000031778;ENSRNOG0000003	ONT.7139;Cct3;Rhbg;Mef2d;Tsacc
ONT.7708.5	ENSRNOG00000060988	Kcnc4
ONT.14337.1	ENSRNOG00000049849;ONT.14142;ENSRNOG00000045618;ENSRNOG00000037661;ENSRNOG00000048011;ONT.14141	Fam199x;ONT.14142;AABR07040686.1;Tmsb15b2;AABR07040695.1;ONT.14141
ONT.8660.5	ENSRNOG00000008081	Ddx27
ONT.5579.2	ENSRNOG00000019150;ENSRNOG00000019267	Polb;Dkk4
ONT.3070.1	ENSRNOG00000002187	Ropn1
ONT.2672.1	ENSRNOG00000012557	Lgals5
ONT.4848.2	ENSRNOG00000022597	Cenpj
ONT.11674.1	ONT.11675	ONT.11675
