# Supplementary material for: Effects of Maternal High-Fructose Diet on Long Non-Coding RNAs and Anxiety-like Behaviors in Offspring
Source: Int J Mol Sci. 2023 Feb 24;24(5):4460. doi: 10.3390/ijms24054460 (PMC10003385; doi:10.3390/ijms24054460)
Supplement: Supplementary file 1 [file ijms-24-04460-s001.zip › Table S3.pdf]

Table S3: The target genes were identified by trans method.

#LncRNA_	Target_mRNA_ID	Symbol
ONT.4405.	ENSRNOG00000001441	Tmem120a
ONT.6013.	ENSRNOG00000021365;ENSRNOG00000016767;ENSRNOG00000005378;ENSRNOG00000049104;ENSRNOG00000001781;ENSRNOG00000020630;ENSRNOG00000013713;ENSRNOG00000017285;ENSRNOG00000054751;ENSRNOG00000059640;ENSRNOG00000014801;ENSRNOG00000013521;ENSRNOG00000051592;ENSRNOG00000001757;ENSRNOG00000021702;ENSRNOG00000003132;ENSRNOG00000018781;ENSRNOG00000024960;ENSRNOG00000003865;ENSRNOG00000050828;ENSRNOG00000014214;ENSRNOG00000032561;ENSRNOG00000021056;ENSRNOG00000005618;ENSRNOG00000019840;ENSRNOG00000001527;ENSRNOG00000011962;ENSRNOG00000030572;	Ybey;Ggps1;Gna15;LOC100911256;Lmln;Il9r;LOC499240;Tab1;Lmbrd2;Ccl28;Exog;Dhfr;AABR07044925.1;Tm4sf19;Mcmdc2;Mip;Map1s;Smco1;Tmigd1;Vkorc1;Rpl27a;Slc52a2;Kcnj14;Fmc1;Mdp1;Cd80;Gin1;Smc5;Pou2f3
ONT.3309.	ONT.7464;ONT.10546	ONT.7464;ONT.10546
ONT.13331	ENSRNOG00000015835	Cacna2d2
ONT.8834.	ENSRNOG00000051719;ENSRNOG00000059604;ENSRNOG00000015971;ENSRNOG00000001346;ENSRNOG00000001156;ENSRNOG00000024149;ENSRNOG00000010174;ENSRNOG00000026705;ENSRNOG00000024808;ENSRNOG00000051929;ENSRNOG00000050437;ENSRNOG00000001720;ONT.4323;ONT.13217;ENSRNOG00000050108;ENSRNOG00000019459;ENSRNOG00000005067;ENSRNOG00000012409;ENSRNOG00000007683;ENSRNOG00000028187;ENSRNOG00000001588;ENSRNOG00000001580;ENSRNOG00000046313;ENSRNOG00000012167;ENSRNOG000000142	AABR07065498.1;AABR07012274.1;Slc12a2;Cops6;Msi1;Prr18;Enpp4;Dgki;Stk39;Phox2b;Bod1l1;Hes1;ONT.4323;ONT.13217;LOC100911319;Oaz1;Zfp36l2;Exosc3;Prdm13;Sp9;Hoxd13;Hoxd9;Basp1;Pou4f2;Zfp503;Kcnma1
ONT.7337.	ENSRNOG00000018232	Srf
ONT.3648.	ONT.4491;ONT.2600	ONT.4491;ONT.2600
ONT.12548	ONT.12072;ENSRNOG00000009610;ENSRNOG00000004150;ENSRNOG00000054080;ENSRNOG00000007240;ENSRNOG00000013767	ONT.12072;AABR07072759.1;Slc9a7;Cgnl1;Rrs1;Ccdc61
ONT.8755.	ENSRNOG00000004532	Fam69b

ONT.1066.?	ENSRNOG00000013059;ENSRNOG00000014230;ENSRNOG00000017133;ENSRNOG00000012434;ENSRNOG00000011756;ENSRNOG00000007281;ENSRNOG00000009314	Aste1;Map1a;LOC306766;Zfp598;Phf3;Flnc;AABR07012307.1
ONT.4436.	ONT.4576;ONT.10546	ONT.4576;ONT.10546
ONT.821.6	ONT.4795;ONT.4013;ONT.2091;ONT.7427;ONT.5818;ONT.5410;ONT.84;ONT.6246;ONT.5032;ONT.1090;ONT.11899	ONT.4795;ONT.4013;ONT.2091;ONT.7427;ONT.5818;ONT.5410;ONT.84;ONT.6246;ONT.5032;ONT.1090;ONT.11899
ONT.6840.:	ONT.12901	ONT.12901
ONT.3718.	ONT.4323	ONT.4323
ONT.1410?	ENSRNOG00000028206;ENSRNOG00000001424;ONT.9048;ENSRNOG00000032776;ENSRNOG00000058785;ENSRNOG00000022326;ENSRNOG00000050158;ENSRNOG00000023668;ENSRNOG00000019681;ENSRNOG00000019199	Pheta1;Cux1;ONT.9048;Srp54a;LOC103692170;Ccdc142;Cracr2b;Scyl1;Pold1;LOC100911692
ONT.4079.:	ENSRNOG00000050828;ENSRNOG00000014801;ENSRNOG00000051592;ENSRNOG00000054751;ENSRNOG00000003132;ENSRNOG00000049104;ENSRNOG00000016214;ENSRNOG00000030572;ENSRNOG00000005618;ENSRNOG00000021365;ENSRNOG00000020630;ENSRNOG00000012734;ENSRNOG00000014214;ENSRNOG00000006467;ENSRNOG00000016767;ENSRNOG00000021056;ENSRNOG00000013713;ENSRNOG00000032561;ENSRNOG00000024960;ENSRNOG00000001781;ENSRNOG00000011879;ENSRNOG00000001757;ENSRNO	Vkorc1;Exog;AABR07044925.1;Lmbrd2;Mip;LOC100911256;Agl;Smc5;Fmc1;Ybey;Il9r;Dcun1d1;Rpl27a;Eif2b2;Ggps1;Kcnj14;LOC499240;Slc52a2;Smco1;Lmln;Nfat5;Tm4sf19;Ptgr2;Heatr3
ONT.1858.:	ENSRNOG00000030714;ENSRNOG00000023812;ENSRNOG0000003	Bsn;Raver2;Defb25
ONT.13711	ENSRNOG00000028523;ENSRNOG00000004686	Tctn1;Spop
ONT.7708.:	ONT.2611	ONT.2611

ONT.1272(	ENSRNOG00000012924;ENSRNOG00000001412;ENSRNOG00000017254;ONT.7504;ENSRNOG00000004744;ENSRNOG00000007147;ENSRNOG00000006441;ENSRNOG00000002956;ENSRNOG00000050158;ENSRNOG00000019199;ONT.4015;ONT.1601;ONT.12137;ONT.12071;ENSRNOG00000053932;ENSRNOG00000009118;ENSRNOG00000014879;ENSRNOG00000013062;ENSRNOG00000024728;ENSRNOG00000012255;ENSRNOG00000037162;ENSRNOG00000002074;ENSRNOG00000004278;ENSRNOG0000003	Nemp2;Epo;Nsun2;ONT.7504;Fam84b;Cyp46a1;Zbtb25;Stim2;Cracr2b;LOC100911692;ONT.4015;ONT.1601;ONT.12137;ONT.12071;Ddx6;Pou2f3;Ttc7a;Cyp24a1;Arhgap22;Ktn1;Slbp;Rest;Dlx3;Tbc1d12
ONT.607.1	ONT.1474;ENSRNOG00000060973;ENSRNOG00000039726	ONT.1474;AABR07013843.1;LOC307727
ONT.14337	ONT.11043;ONT.7479;ONT.7055;ONT.6598;ONT.615;ONT.4421	ONT.11043;ONT.7479;ONT.7055;ONT.6598;ONT.615;ONT.4421
ONT.1129:	ENSRNOG00000024960;ENSRNOG00000050828;ENSRNOG00000014801;ENSRNOG00000038166;ENSRNOG00000051592;ONT.264;ONT.1525;ENSRNOG00000031896;ENSRNOG00000023346;ENSRNOG00000057245;ENSRNOG00000052415;ENSRNOG00000025324;ENSRNOG00000019232;ENSRNOG00000016767;ENSRNOG00000039528;ENSRNOG00000029938;ENSRNOG00000020630;ENSRNOG00000010839;ENSRNOG00000024266;ENSRNOG00000020777;ENSRNOG00000018476;ENSRNOG00000053675;ENS	Smco1;Vkorc1;Exog;Ptgr2;AABR07044925.1;ONT.264;ONT.1525;Stt3a;Cplane2;Rbbp8nl;Gstt2;Spire1;RGD1311345;Ggps1;Fam32a;Pik3c2b;Il9r;Krt222;Haus5;Zfp382;LOC100911225;Dhh;Nfat5
ONT.1166(	ONT.14318;ONT.4070;ONT.12913;ONT.1965;ONT.6772;ONT.4805;ONT.13045;ONT.12867;ONT.8309;ONT.7427;ONT.7359;ONT.6926;ONT.6872;ONT.6871	ONT.14318;ONT.4070;ONT.12913;ONT.1965;ONT.6772;ONT.4805;ONT.13045;ONT.12867;ONT.8309;ONT.7427;ONT.7359;ONT.6926;ONT.6872;ONT.6871
ONT.3648.	ENSRNOG00000047234	AABR07057530.1
ONT.9411.:	ENSRNOG00000024266;ONT.3512;ENSRNOG00000007310;ENSRNOG00000057245;ENSRNOG00000061102;ENSRNOG00000008336;ENSRNOG00000014909	Haus5;ONT.3512;Klrb1a;Rbbp8nl;Taf9b;Tnfrsf11b;Sh2d5
ONT.1797.	ENSRNOG00000001525	Igsf11
ONT.2975.	ENSRNOG00000013004;ENSRNOG00000015008;ENSRNOG00000053337;ENSRNOG00000019657;ONT	Akr1d1;Gcm2;Ly49s6;Mk1;ONT.3524

ONT.1285.	ENSRNOG00000005639;ENSRNOG00000007443	Ar;Jag1
ONT.7752.:	ONT.4346;ENSRNOG00000011619;ENSRNOG00000011927;ENSRNOG00000014647;ENSRNOG0000003	ONT.4346;Myo9a;Sdc3;Cbfb;Atp8a1
ONT.7977.	ENSRNOG00000026226	Hook1
ONT.7708.	ONT.2611	ONT.2611
ONT.8054.:	ONT.197	ONT.197
ONT.5671.:	ONT.8149	ONT.8149
ONT.994.5	ONT.3501;ENSRNOG00000005990;ENSRNOG00000010540;ONT.472	ONT.3501;Wdsub1;Mrpl45;ONT.4722
ONT.2768.	ONT.6729;ENSRNOG00000001811;ONT.7462;ENSRNOG00000028814;ONT.733;ONT.8101;ONT.11092;ONT.9523;ENSRNOG00000021555;ONT.5609;ONT.11182;ONT.9917;ONT.7395;ONT.7341;ONT.6314;ONT.519;ONT.1801;ENSRNOG00000011321;ENSRNOG00000039859;ENSRNOG00000005749;ENSRNOG00000014265;ONT.9955;ONT.7065;ONT.69;ONT.6218;ONT.40;ONT.3501;ONT.1731;ONT.12667;ONT.12	ONT.6729;Fgfr1op2;ONT.7462;Oasl2;ONT.733;ONT.8101;ONT.11092;ONT.9523;Mis18a;ONT.5609;ONT.11182;ONT.9917;ONT.7395;ONT.7341;ONT.6314;ONT.519;ONT.1801;Rftn1;Dnmt1;Foxred2;Tnfrsf19;ONT.9955;ONT.7065;ONT.69;ONT.6218;ONT.40;ONT.3501;ONT.1731;ONT.12667;ONT.12384
ONT.6043.:	ENSRNOG00000052113	Ppp1r9b
ONT.6513.	ENSRNOG00000062231;ENSRNOG00000020384;ONT.8290;ENSRNOG00000019677;ENSRNOG00000008683;ENSRNOG00000002149;ENSRNOG00000039216;ENSRNOG00000027061;ENSRNOG00000057848	AABR07037451.2;Fam13b;ONT.8290;Arid3b;Alk;Nkx6-1;RGD1562319;Nxpe5l1;Cacng8
ONT.13207	ENSRNOG00000012791	Ajuba
ONT.546.1	ENSRNOG00000019193;ONT.5989;ENSRNOG00000011101	Stx1b;ONT.5989;Twist1
ONT.12253	ENSRNOG00000019482;ONT.7748;ENSRNOG00000031135;ENSRNOG00000004831;ENSRNOG00000016708;ENSRNOG00000003936;ENSRNOG00000014928;ENSRNOG00000010918;ENSRNOG00000005371;ENSRNOG00000017489;ENSRNOG00000058940;ENSRNOG0000000	Gnao1;ONT.7748;Smarcc2;Arid2;Necab3;Pwwp2a;Apba1;Cebpa;Klhl29;Gse1;Rab11fip1;Hmx1
ONT.1857.:	ENSRNOG00000007810	Gdf6
ONT.3718.	ENSRNOG00000007445;ENSRNOG00000021380;ONT.4323;ENSRNOG00000049575	Asph;Fads6;ONT.4323;Atn1

ONT.3179.4	ENSRNOG00000014876;ENSRNOG00000019825;ENSRNOG00000007649;ENSRNOG00000011954;ENSRNOG00000008415;ENSRNOG00000007779;ENSRNOG00000016891;ENSRNOG00000002659	Lpin2;Zdhhc24;Dnm2;Kiss1r;Nab2;Kank4;Uimc1;Ciita
ONT.3585.	ENSRNOG00000006759;ENSRNOG00000010960;ONT.10509;ENSRNOG00000057620	Ankrd24;Ankh;ONT.10509;Slc6a8
ONT.3141.:	ENSRNOG00000025011	Chd8
ONT.1954.:	ENSRNOG00000029012	Shisa6
ONT.2740.	ENSRNOG00000013713;ENSRNOG00000006467;ENSRNOG00000021365;ENSRNOG00000054751;ENSRNOG00000014801;ENSRNOG00000003132;ENSRNOG00000049104;ENSRNOG00000005618;ENSRNOG00000003865;ENSRNOG00000005378;ENSRNOG00000016767;ENSRNOG00000014214;ENSRNOG00000051592;ENSRNOG00000021056;ENSRNOG00000020630;ENSRNOG00000016214;ENSRNOG00000019840;ENSRNOG00000013521;ENSRNOG00000012734;ENSRNOG00000059997;ENSRNOG00000050828;ENSRNOG00000032561;ENSRNOG00000024960;ENSRNOG00000030572;ENSRNOG00000038166;ENSRNOG00000011962;ENSRNOG00000059640;ENSRNOG00000015459;	LOC499240;Eif2b2;Ybey;Lmbrd2;Exog;Mip;LOC100911256;Fmc1;Tmigd1;Gna15;Ggps1;Rpl27a;AABR07044925.1;Kcnj14;Il9r;Agl;Mdp1;Dhfr;Dcun1d1;Brip1;Vkorc1;Slc52a2;Smco1;Smc5;Ptgr2;Gin1;Ccl28;Heatr3;Pnoc;Lmln
ONT.11332	ENSRNOG00000040314	AABR07034637.1
ONT.1954.	ENSRNOG00000029012	Shisa6
ONT.3585.:	ENSRNOG00000010888;ENSRNOG00000049123;ENSRNOG00000006759;ENSRNOG00000048430;ENSRNOG00000017208;ENSRNOG00000004208;ENSRNOG00000048408;ENSRNOG00000001608;ENSRNO	Ankrd33b;AABR07066510.1;Ankrd24;Myo18b;Cspg4;Crim1;Olr869;Nxpe3;Scnn1g
ONT.8054.:	ENSRNOG00000016684;ONT.197	Wnk2;ONT.197
ONT.11985	ONT.4491	ONT.4491
ONT.5636.	ONT.1149	ONT.1149
ONT.6043.:	ENSRNOG00000052113	Ppp1r9b
ONT.1978.	ENSRNOG00000052486;ONT.3077;ENSRNOG00000050485	Kcna6;ONT.3077;Gas1
ONT.8030.:	ENSRNOG00000025806	Prr3

ONT.13416	ENSRNOG00000005157;ENSRNOG00000003269;ENSRNOG00000016815;ONT.9207;ONT.8148;ENSRNOG00000053805;ENSRNOG00000019894;ENSRNOG00000009592;ENSRNOG00000014874;ENSRNOG00000001640;ENSRNOG00000001528;ENSRNOG00000052273	Rbbp7;Atp6v0e1;Tmem135;ONT.9207;ONT.8148;Akain1;Izumo1r;Cyb5r3;Zfyve28;Tomm70;Gap43;RGD1305713
ONT.1176	ENSRNOG00000003132;ENSRNOG00000050828;ENSRNOG00000014801;ENSRNOG00000051592;ENSRNOG00000030572;ENSRNOG00000049104;ENSRNOG00000016214;ENSRNOG00000021365;ENSRNOG00000013713;ENSRNOG00000006467;ENSRNOG00000005618;ENSRNOG00000054751;ENSRNOG00000012734;ENSRNOG00000005378;ENSRNOG00000016767;ENSRNOG00000014231;ENSRNOG00000003865;ENSRNOG00000021056;ENSRNOG00000020630;ENSRNOG00000059997;ENSRNOG00000015459;ENSRNOG00000038166;ENSRNOG00000014214;ENSRNOG00000059640;ENSRNOG00000022502;ENSRNOG00000020658;ENSRNOG00000011879;ENSRNOG00000000503;	Mip;Vkorc1;Exog;AABR07044925.1;Smc5;LOC100911256;Agl;Ybey;LOC499240;Eif2b2;Fmc1;Lmbrd2;Dcun1d1;Gna15;Ggps1;Pnoc;Tmigd1;Kcnj14;Il9r;Brip1;Heatr3;Ptgr2;Rpl27a;Ccl28;Nsrp1;Aarsd1;Nfat5;Ppard;Mdp1;Lmln
ONT.1791.	ENSRNOG00000008781;ONT.3501;ENSRNOG00000005990;ENSRNOG00000010540;ONT.11021;ENSRNOG00000008635	Erg28;ONT.3501;Wdsub1;Mrpl45;ONT.11021;Mks1
ONT.6043.	ENSRNOG00000055371;ENSRNOG00000052113	Sptbn4;Ppp1r9b
ONT.5570.	ENSRNOG00000010258	Vhl
ONT.1571.	ONT.2405;ONT.10457	ONT.2405;ONT.10457
ONT.8884.	ENSRNOG00000001520;ENSRNOG00000005438	Dlx1;Pcsk2
ONT.361.1	ONT.1905;ONT.7960;ONT.12901;ONT.420	ONT.1905;ONT.7960;ONT.12901;ONT.420
ONT.4343.	ONT.263;ONT.18	ONT.263;ONT.18
ONT.1222.	ENSRNOG00000039726;ENSRNOG00000022598;ENSRNOG0000000	LOC307727;Trerf1;Igsf9b
ONT.7708.	ONT.2611	ONT.2611
ONT.1145	ENSRNOG00000052415	Gstt2
ONT.1630.	ONT.10401	ONT.10401
ONT.1013	ENSRNOG00000020829;ONT.420	Them4;ONT.420
ONT.6092.	ENSRNOG00000047628;ENSRNOG00000000436;ENSRNOG0000000	Khsrp;Egfl8;Aatk

ONT.11020	ENSRNOG00000056476;ENSRNOG00000042660	Slc22a13;LOC685081
ONT.10703	ENSRNOG00000000233;ENSRNOG00000055371;ENSRNOG00000056786;ENSRNOG00000027037;ENSRNOG00000024535;ENSRNOG000	Grm6;Sptbn4;Piezo1;Alox12;Zzef1;Slit3
ONT.7876.	ENSRNOG00000047657;ENSRNOG00000027032	C4a;Rhbdd3
ONT.6092.	ENSRNOG00000047628;ENSRNOG00000004392	Khsrp;Aatk
ONT.13374	ENSRNOG00000020829;ENSRNOG00000053553	Them4;AABR07059168.1
ONT.5585.	ONT.5372	ONT.5372
ONT.6840.	ONT.12901	ONT.12901
ONT.686.3	ONT.160;ONT.6214;ONT.5132;ENSRNOG00000000308;ONT.9797;ONT.5946;ONT.5448;ONT.1036;ENSRNOG00000036699;ONT.9605;ONT.7220;ONT.5807;ONT.5671;ONT.4753;ONT.3990;ONT.3598;ENSRNOG00000001073;ENSRNOG00000005408;ENSRNOG00000014935;ONT.9269;ONT.8192;ONT.7133;ONT.6392;ONT.4832;ONT.420;ONT.3492;ONT.3381;ONT.258	ONT.160;ONT.6214;ONT.5132;Zbtb24;ONT.9797;ONT.5946;ONT.5448;ONT.1036;Faap100;ONT.9605;ONT.7220;ONT.5807;ONT.5671;ONT.4753;ONT.3990;ONT.3598;RGD1563482;LOC100911674;Twnk;ONT.9269;ONT.8192;ONT.7133;ONT.6392;ONT.4832;ONT.420;ONT.3492;ONT.3381;ONT.258
ONT.96.1	ENSRNOG00000019376	Zfp329
ONT.9965.	ENSRNOG00000056011;ENSRNOG00000000956	LOC314140;Rasl11a
ONT.12754	ENSRNOG00000019355;ENSRNOG00000019317	Lman1l;Cplx3
ONT.11841	ENSRNOG00000008635	Mks1
ONT.10405	ONT.5416;ONT.3910;ONT.3735;ONT.13452;ONT.8255;ONT.6650;ONT.6331;ONT.5693;ONT.5464;ONT.5460;ONT.516;ONT.5076;ONT.366;ONT.3169;ONT.2665;ONT.2600;O	ONT.5416;ONT.3910;ONT.3735;ONT.13452;ONT.8255;ONT.6650;ONT.6331;ONT.5693;ONT.5464;ONT.5460;ONT.516;ONT.5076;ONT.366;ONT.3169;ONT.2665;ONT.2600;O
ONT.6676.	ENSRNOG00000009348;ENSRNOG00000006049;ENSRNOG00000001104;ENSRNOG00000024931;ENSRNOG00000016242	Nos3;Rfx1;Foxk1;Ccdc88b;Fzd1
ONT.7823.	ENSRNOG00000024027;ENSRNOG00000010944;ENSRNOG00000020673;ENSRNOG00000021871;ENSRNOG00000026942	Lemd3;Hyou1;Pbxip1;Gcn1;RGD1311595
ONT.11725	ENSRNOG00000045524	Slc39a3

ONT.11200	ENSRNOG00000004160;ENSRNOG00000007683;ENSRNOG00000001588;ENSRNOG00000043215;ENSRNOG00000016902;ENSRNOG00000014192;ENSRNOG00000001452;ENSRNOG00000020193;ENSRNOG00000028679;ENSRNOG00000004773;ENSRNOG00000012647;ENS	Prps2;Prdm13;Hoxd13;Rtbdn;Ptf1a;Naa30;Fzd9;Runx2;Hoxc13;Yaf2;Nkx2-4;Basp1
ONT.13731	ENSRNOG00000019965;ENSRNOG00000050669;ENSRNOG00000004019;ENSRNOG00000013583;ENSRNOG00000010128;ENSRNOG00000001870;ENSRNOG00000018748;ENSRNOG00000011060	Tgfb1i1;LOC100911515;Phlda1;Tbc1d8;Slc27a2;Lztr1;Slc16a11;Unc119
ONT.3179.:	ENSRNOG00000004091;ENSRNOG00000002659	Cwc25;Ciita
ONT.131.1	ENSRNOG00000015517;ENSRNOG00000017072;ENSRNOG00000037514;ONT.4909;ENSRNOG00000008694;ENSRNOG00000003256;ENSRNOG00000036802;ENSRNOG00000004940;ONT.7082;ENSRNOG00000039086;ENSRNOG00000004442;ENSRNOG00000011774	Zfp444;Slc16a14;Qser1;ONT.4909;Miox;Ccng1;Snhg11;Rnf215;ONT.7082;Ccdc153;Dglucy;Fblim1
ONT.2879.:	ENSRNOG00000049876	Prkar1a
ONT.4323.	ENSRNOG00000006975;ENSRNOG00000008822;ENSRNOG00000016271;ENSRNOG00000011158;ENSRNOG00000003936;ENSRNOG00000016110;ENSRNOG00000046607;ENSRNOG00000023497;ENSRNO	Wasl;Fkbp1a;Itm2b;Ppp2r2a;Pwwp2a;Kcnk12;Cited4;Foxe1;Rprd2
ONT.686.1	ONT.5448;ONT.1036;ONT.9605;ONT.7220;ONT.5807;ONT.4753;ONT.3990;ONT.9269;ONT.8192;ONT.7133;ONT.6392;ONT.4832;ONT.420;ONT.3492;ONT.3381;ONT.258	ONT.5448;ONT.1036;ONT.9605;ONT.7220;ONT.5807;ONT.4753;ONT.3990;ONT.9269;ONT.8192;ONT.7133;ONT.6392;ONT.4832;ONT.420;ONT.3492;ONT.3381;ONT.258
ONT.583.2	ENSRNOG00000021285;ENSRNOG00000019968	Celsr1;Trim8
ONT.8140.	ENSRNOG00000006467	Eif2b2
ONT.484.1	ONT.12966	ONT.12966
ONT.8660.	ENSRNOG00000015295;ENSRNOG00000019653;ENSRNOG00000006570;ENSRNOG00000053991;ENSRNOG00000010841;ENSRNOG00000001892;ENSRNOG00000049661	Ctdsp1;Csrnp2;Plekhg3;P3h1;Col8a2;Tbx1;Inafm1
ONT.3526.:	ONT.7937;ONT.6746	ONT.7937;ONT.6746
ONT.2511.	ONT.5472;ENSRNOG00000019959	ONT.5472;Kcnc3

ONT.9581.[illegible]	ENSRNOG00000003657;ENSRNOG00000018809;ENSRNOG00000032446;ENSRNOG00000029079;ENSRNOG00000013140;ENSRNOG00000001126;ONT.4129;ENSRNOG00000001706;ENSRNOG00000018019	Pkmyt1;Psmd5;Recql4;Hspb7;Pdzd2;Fbxw8;ONT.4129;Kalrn;Hspa12a
ONT.5110.	ENSRNOG00000034241;ENSRNOG00000028984;ENSRNOG0000005	Set;RGD1563307;AABR07031963.1
ONT.1191[illegible]	ENSRNOG00000004200	Sybu
ONT.9411.[illegible]	ENSRNOG00000007851;ENSRNOG00000037275;ENSRNOG00000016010;ENSRNOG00000052142;ENSRNOG00000002032;ENSRNOG00000014940;ENSRNOG00000012564;ENSRNOG00000027654;ENSRNOG00000025808;ENSRNOG00000020229;ONT.9141;ENSRNOG00000007115;ENSRNOG00000024543;ENSRNOG00000031896;ENSRNOG00000013529;ENSRNOG00000052415;ENSRNOG00000014461;ENSRNOG00000024266;ENSRNOG00000008336;ENSRNOG00000014909;ENSRNOG00000007310;ENSRNOG00000057245;ENSRNOG00000027867;ENSRNOG00000039297;ENSRNOG00000002194;ENSRNOG0000004	Psma3l;Tlcd2;Mul1;Ahsa2;Ifngr2;Sfrp5;RGD1564541;Appbp2;Aars2;Homer3;ONT.9141;Crebl2;Mfsd4b;Stt3a;Stk4;Gstt2;Galns;Haus5;Tnfrsf11b;Sh2d5;Klrb1a;Rbbp8nl;Rexo4;Mrpl52;Coq2;Cenpw;Hsd3b7;ONT.3512
ONT.6043.[illegible]	ENSRNOG00000051678	AABR07012475.1
ONT.5511.[illegible]	ONT.19	ONT.19
ONT.4476.[illegible]	ENSRNOG00000005464;ENSRNOG00000039928;ONT.2605;ENSRNOG00000004650;ENSRNOG000000	Lgalsl;AABR07020786.1;ONT.2605;Begain;Barx1
ONT.7962.	ENSRNOG00000056670;ENSRNOG00000040249	AABR07000902.1;AC115420.1
ONT.1374.	ONT.733;ONT.2665;ONT.13452;ONT.12350;ONT.1024	ONT.733;ONT.2665;ONT.13452;ONT.12350;ONT.1024
ONT.1055[illegible]	ENSRNOG00000047521;ENSRNOG00000003084	Ccdc166;Parp1
ONT.6216.	ONT.7361;ENSRNOG00000033099;ENSRNOG00000038369	ONT.7361;Dcc;AABR07038477.1
ONT.4476.[illegible]	ENSRNOG00000005464;ENSRNOG00000039928;ENSRNOG0000001	Lgalsl;AABR07020786.1;Barx1
ONT.1383.[illegible]	ENSRNOG00000030156	LOC102547344
ONT.1655.[illegible]	ENSRNOG00000056330;ENSRNOG00000017329;ENSRNOG0000002	Cnbd1;Tmem129;Pdyn
ONT.4538.	ONT.12901;ONT.69	ONT.12901;ONT.69
ONT.6347.	ONT.13272	ONT.13272
ONT.5878.	ONT.5662	ONT.5662

ONT.3640.	ENSRNOG00000058371;ONT.5057;ONT.6989;ONT.4170;ONT.6442;ONT.13045;ONT.10952	AABR07013154.2;ONT.5057;ONT.6989;ONT.4170;ONT.6442;ONT.13045;ONT.10952
ONT.1003.	ENSRNOG00000018217;ENSRNOG00000007405	Syt5;Krba1
ONT.13385	ONT.8916;ONT.6288;ONT.55;ONT.13525	ONT.8916;ONT.6288;ONT.55;ONT.13525
ONT.3648.	ONT.4491;ONT.2600	ONT.4491;ONT.2600
ONT.175.1	ONT.2059;ENSRNOG00000058371;ONT.8463;ONT.4491;ONT.10457	ONT.2059;AABR07013154.2;ONT.8463;ONT.4491;ONT.10457
ONT.5347.	ENSRNOG00000056956;ENSRNOG00000016767;ENSRNOG00000020630;ENSRNOG00000013713;ENSRNOG00000003132;ENSRNOG00000049104;ENSRNOG00000015459;ENSRNOG00000014801;ENSRNOG00000059997;ENSRNOG00000062295;ENSRNOG00000001781;ENSRNOG00000006467;ENSRNOG00000005618;ENSRNOG00000000503;ENSRNOG00000022502;ENSRNOG00000052273;ENSRNOG00000054751;ENSRNOG00000030572;ENSRNOG00000021365;ENSRNOG00000010839;ENSRNOG00000012734;ENSRNOG00000001527;ENSRNOG00000003865;ENSRNOG00000051592;ENSRNOG00000050828;ENSRNOG00000016214;ENSRNOG00000021056;ENSRNOG00000011962;	Thap1;Ggps1;Il9r;LOC499240;Mip;LOC100911256;Heatr3;Exog;Brip1;Gm9918;Lmln;Eif2b2;Fmc1;Ppard;Nsrp1;RGD1305713;Lmbrd2;Smc5;Ybey;Krt222;Dcun1d1;Cd80;Tmigd1;AABR07044925.1;Vkorc1;Agl;Kcnj14;Gin1;H2afv
ONT.671.1	ENSRNOG00000020134;ENSRNOG00000052687	Upf1;Megf8
ONT.14337	ONT.11043;ONT.7479;ONT.7055;ONT.6598;ONT.615;ONT.4421	ONT.11043;ONT.7479;ONT.7055;ONT.6598;ONT.615;ONT.4421
ONT.6102.	ENSRNOG00000004218;ENSRNOG00000014909;ENSRNOG00000009227;ENSRNOG00000001483;ENSRNOG00000001527;ENSRNOG00000000082;ONT.9340;ONT.12603;ENSRNOG00000025184;ENSRNOG00000009506;ENSRNOG00000007197;ENSRNOG00000005749;ENSRNOG00000060436;ENSRNOG00000010227;ENSRNOG00000006399	Klhl28;Sh2d5;Aplnr;Rcc1l;Cd80;Hltf;ONT.9340;ONT.12603;Prss35;Mre11a;Nr1h4;Foxred2;Vti1b;Gpatch2l;Synj2bp
ONT.821.7	ONT.4795;ONT.4013;ONT.2091;ONT.7427;ONT.5818;ONT.5410;ONT.84;ONT.6246;ONT.5032;ONT.1090;ONT.11899	ONT.4795;ONT.4013;ONT.2091;ONT.7427;ONT.5818;ONT.5410;ONT.84;ONT.6246;ONT.5032;ONT.1090;ONT.11899

ONT.10715	ENSRNOG00000017354;ONT.9684;ENSRNOG00000017871;ENSRNOG00000056087;ENSRNOG00000049045;ENSRNOG00000010771;ENSRNOG00000052687;ONT.13673;ENSRNOG00000046981;ENSRNOG00000029912;ENSRNOG00000003657;ENSRNOG00000055371;ENSR	Zyx;ONT.9684;Sidt2;Erich2;AABR07045032.1;Pkd1;Megf8;ONT.13673;Gp1bb;Sept5;Pkmyt1;Sptbn4;Col17a1
ONT.1978.[illegible]	ENSRNOG00000052486;ONT.3077;ONT.1389	Kcna6;ONT.3077;ONT.1389
ONT.4343.	ENSRNOG00000061484	Adamts2
ONT.1413[illegible]	ENSRNOG00000032530;ENSRNOG00000028198;ENSRNOG00000008169;ENSRNOG00000016870;ENSRNOG00000017602;ENSRNOG00000013328;ENSRNOG00000001080;ONT.7962;ENSRNOG00000054008;ENSRNOG00000009906;ENSRNOG00000010905;ENSRNOG00000013973;ENSRNOG00000004666;ENSRNOG00000060674;ENSRNOG00000047532;ENSRNOG00000046538;ENSRNOG00000043302;ENSRNOG00000042797;ENSRNOG0000004	AABR07072821.1;Sh2b3;Slc24a2;Pcif1;Il34;Rbpms;Arl6ip4;ONT.7962;Scamp5;Slfnl1;Dlx5;Lcn2;Prr5l;RGD1561557;LOC687631;AC108323.2;LOC690478;LOC690460;AABR07044583.2;AABR07044583.1
ONT.6102.[illegible]	ONT.13445	ONT.13445
ONT.263.1	ENSRNOG00000014493;ENSRNOG00000004091;ENSRNOG00000018752;ENSRNOG00000019196;ENSRNOG00000018476;ENSRNOG00000012763;ENSRNOG00000062125;ENSRNOG00000006237;ENSRNOG00000008118;ONT.8149;ENSRNOG00000023346;ENSRNOG00000025324;ENSRNOG00000052687;ENSRNOG00000014903;ENSRNOG00000009172;ENSRNOG00000014461;ENSRNOG00000009815;ENSRNOG00000004263;ENSRNOG00000024410;ENSRNOG00000024266;ENSRNOG00000013529;ENSRNOG00000019414;ENSRNOG00000019376;ENSRNOG00000010345;ONT.1525;ENSRNOG00000027088;ENSRNOG00000004660;ENSRNOG00000007637;ENSRNOG00000036686;ENS	Golga1;Cwc25;Clcf1;Xpnpep3;LOC100911225;Cwf19l1;Aox3;Gpr37l1;Sync;ONT.8149;Cplane2;Spire1;Megf8;Zfyve27;Cpvl;Galns;Pnma2;Ints7;Blvrb;Haus5;Stk4;Tmem79;Zfp329;Ssmem1;ONT.1525;Ube2k;Fzd6;Acer2;Anapc11;Itprip
ONT.8054.[illegible]	ONT.197	ONT.197

ONT.8775.	ENSRNOG00000003185;ENSRNOG00000014137;ENSRNOG00000010777;ENSRNOG00000006813;ENSRNOG00000017428;ENSRNOG00000014658;ENSRNOG00000003738;ENSRNOG00000003669;ENSRNOG00000002364;ONT.5434;ENSRNOG00000010188;ENSRNOG00000023020;ENSRNOG00000026866;ENSRNOG00000012785;ENSRNOG00000030212;ENSRNOG00000052637	Acbd3;Fbln1;Tox;Sumf1;Map1b;Zfp423;Ush2a;Myocd;Rnf112;ONT.5434;Satb2;Fdx2;Syn3;Armc10;Cerkl;Samd1;Tfap2a
ONT.8859.	ENSRNOG00000011970;ENSRNOG00000014522;ENSRNOG00000039305;ENSRNOG00000052704;ENSRNOG00000004409;ENSRNOG00000007062;ENSRNOG00000060105;ENSRNOG00000016870;ENSRNO	Tmem82;Mlycd;AABR07037250.1;Atp6v0a2;Sash3;Rin3;Astn2;Pcif1;Jph3
ONT.12463	ENSRNOG00000013603	Dffa
ONT.3512.	ONT.5416;ONT.3735;ONT.11826;ONT.6650;ONT.2362;ONT.6560;ONT.615;ONT.5693;ONT.5076;ONT.1597;ONT.12069;ONT.8699;ONT.6705;ONT.516;ONT.4374;ONT.2790;ONT.1442;ONT.13956;ONT.13497;ONT.8286;ONT.12640;ONT.12384	ONT.5416;ONT.3735;ONT.11826;ONT.6650;ONT.2362;ONT.6560;ONT.615;ONT.5693;ONT.5076;ONT.1597;ONT.12069;ONT.8699;ONT.6705;ONT.516;ONT.4374;ONT.2790;ONT.1442;ONT.13956;ONT.13497;ONT.8286;ONT.12640;ONT.12384
ONT.4275.	ENSRNOG00000002154	Mepe
ONT.9299.	ENSRNOG00000057487;ENSRNOG00000046323;ENSRNOG00000013623;ENSRNOG00000025520;ENSRNOG00000025142	Krtap12-2;LOC100361739;Amer2;Lbx1;Mex3b
ONT.11545	ENSRNOG00000013529;ENSRNOG00000000515;ENSRNOG00000018999;ENSRNOG00000039297	Stk4;Mapk13;Cdc14b;Mrpl52
ONT.7591.	ENSRNOG00000007610;ENSRNOG00000008644;ONT.11507;ENSRNOG00000022166;ENSRNOG00000051472;ENSRNOG00000016526;ENSRNOG00000002292;ENSRNOG00000059604;ENSRNOG00000013928;ENSRNOG00000014237;ENSRNOG00000005985;ENSRNOG0000000	Gdf11;Nkx2-1;ONT.11507;Ammecr1;Hoxd11;Dsg2;Hnrnpd;AABR07012274.1;Dsp;Zfp503;Kcnma1;Hoxb3;ONT.8706
ONT.10587	ONT.10125;ONT.11542	ONT.10125;ONT.11542
ONT.8028.	ENSRNOG00000000795	RT1-N3
ONT.3659.	ENSRNOG00000018988;ENSRNOG00000017119;ENSRNOG00000036660;ENSRNOG00000002831	Ing5;Ciao2a;Fn3krp;Wfikkn2
ONT.1374.	ONT.2665;ONT.13452;ONT.12350;ONT.1024	ONT.2665;ONT.13452;ONT.12350;ONT.1024
ONT.1841.	ONT.11388	ONT.11388

ONT.3640.	ENSRNOG00000058371;ONT.5057;ONT.6989;ONT.4170;ONT.6442;ONT.13045;ONT.10952	AABR07013154.2;ONT.5057;ONT.6989;ONT.4170;ONT.6442;ONT.13045;ONT.10952
ONT.4194.	ONT.8673	ONT.8673
ONT.1177	ENSRNOG00000007509;ENSRNOG00000050735;ENSRNOG00000019572;ENSRNOG00000002267	Slc1a6;Cd99;Lsm4;Chic2
ONT.6686.	ENSRNOG00000005342;ENSRNOG00000013847	Rassf5;Nova2
ONT.3035.	ENSRNOG00000042133;ONT.1054	AABR07006275.1;ONT.10546
ONT.5932.	ONT.7634;ONT.13272	ONT.7634;ONT.13272
ONT.1433	ENSRNOG00000061121;ENSRNOG00000029591;ENSRNOG0000000	Wdfy3;Pkdrej;Otub2
ONT.7708.	ONT.2611	ONT.2611
ONT.5570.	ENSRNOG00000003970;ENSRNOG00000025819	Tnpo2;Olr202
ONT.1087	ENSRNOG00000054086;ENSRNOG00000013089	Sp5;Kif13b
ONT.3457.	ONT.6219;ONT.2059;ENSRNOG00000030714;ENSRNOG00000058371	ONT.6219;ONT.2059;Bsn;AABR07013154.2
ONT.4394.	ONT.887;ONT.8314;ONT.6288;ONT.55	ONT.887;ONT.8314;ONT.6288;ONT.55
ONT.115.1	ENSRNOG00000042471;ENSRNOG00000061132;ENSRNOG00000020307;ENSRNOG00000037911;ENSRNOG00000023143;ENSRNOG00000015008;ENSRNOG00000053337;ENSRNOG00000013004	RGD1560324;LOC103694210;RGD1309139;LOC680227;Nlrp1a;Gcm2;Ly49s6;Akr1d1
ONT.8894.	ENSRNOG00000051458;ENSRNOG00000007478;ENSRNOG00000002085;ENSRNOG00000016910;ENSRNOG00000038212;ENSRNOG00000020304;ONT.7464;ENSRNOG00000058568;ENSRNOG00000009766;ONT.264;ONT.2284;ENSRNOG00000009008;ENSRNOG00000006934;ENSRNOG00000000704;ENSRNOG00000010350;ENSRNOG00000008713;ENSRNOG00000005153;ENSRNOG00000039278;ENSRNOG00000008908;ENSRNOG00000015691;ENSRNOG00000018700;ENSRNOG00000004857;ENSRNOG00000005724;ENSRNOG00000024952;ENSRNOG00000004414;ENSRNOG00000001527;ENSRNOG00000004395;	Hic2;Cry2;Thegl;Ralgps1;Socs6;Pdcd11;ONT.7464;Dhrs9;Gpr180;ONT.264;ONT.2284;Rab39a;Acvr1b;Cmklr1;Rcan2;Slc41a2;Tedc1;Slc25a51;Slc35a1;Inka2;Mobp;L2hgdh;LOC100910771;Atp8b1;F11r;Cd80;Fbxw11;ONT.4576
ONT.671.2	ENSRNOG00000016145;ENSRNOG00000009897;ENSRNOG00000030247;ENSRNOG00000020134;ENSRNOG00000052687	Strn4;Clcnkb;Ankhd1;Upf1;Megf8

ONT.6092.	ENSRNOG00000047628;ENSRNOG00000057817;ENSRNOG00000055809;ENSRNOG00000000436;ENSRNOG00000004392	Khsrp;Epb41l1;LOC100911769;Egfl8;Aatk
ONT.198.2	ONT.2189	ONT.2189
ONT.8755.	ENSRNOG00000004532	Fam69b
ONT.7349.	ENSRNOG00000019968	Trim8
ONT.3972.	ONT.6288;ENSRNOG00000060998	ONT.6288;AABR07029467.2
ONT.12642	ONT.6469	ONT.6469
ONT.5585.	ONT.5372	ONT.5372
ONT.2774.	ENSRNOG00000019196;ENSRNOG00000014493;ENSRNOG00000062125;ENSRNOG00000012763;ENSRNOG00000004091;ENSRNOG00000018752;ENSRNOG00000018476;ENSRNOG00000023346;ENSRNOG00000006237;ENSRNOG00000008118;ENSRNOG00000019414;ONT.8149;ONT.1525;ENSRNOG00000025324;ENSRNOG00000004263;ENSRNOG00000052687;ENSRNOG00000014461;ENSRNOG00000036686;ENSRNOG00000007637;ENSRNOG00000009172;ENSRNOG00000027434;ENSRNOG00000009815;ENSRNOG00000024410;ENSRNOG00000024266;ENSRNOG00000013529;ENSRNOG00000019376;ENSRNOG00000058790;ENSRNOG00000014903;ENSRNOG00000004660;ENS	Xpnpep3;Golga1;Aox3;Cwf19l1;Cwc25;Clcf1;LOC100911225;Cplane2;Gpr37l1;Sync;Tmem79;ONT.8149;ONT.1525;Spire1;Ints7;Megf8;Galns;Anapc11;Acer2;Cpvl;Fitm2;Pnma2;Blvrb;Haus5;Stk4;Zfp329;Fam120b;Zfyve27;Fzd6;Ccl9
ONT.4682.	ENSRNOG00000004555;ENSRNOG00000004672	Sec14l3;Sec14l2
ONT.8911.	ENSRNOG00000004544;ENSRNOG00000016309;ENSRNOG00000032148;ENSRNOG00000029260;ENSRNOG00000000704;ENSRNOG00000033266;ENSRNOG00000003879;ENSRNOG00000018515;ONT.13359;ENSRNOG00000028362;ENSRNOG00000024114;ENSRNOG00000025601;ENSRNOG00000019677	Ube2e3;Rgp1;AABR07018050.1;Pitpnm2;Cmklr1;Prss30;Rnf167;Det1;ONT.13359;Unc80;Zdbf2;Topaz1;Arid3b
ONT.1287	ENSRNOG00000042502	Smim17
ONT.3728.	ENSRNOG00000005965;ENSRNOG00000008235;ENSRNOG00000022745;ENSRNOG00000018988;ENSRNOG00000036798	Irak4;Mylk2;RGD1306502;Ing5;Dusp3
ONT.9465.	ENSRNOG00000028176	Elmod2
ONT.9189.	ENSRNOG00000002826;ONT.9392	Hsd17b7;ONT.9392

ONT.9465.4	ENSRNOG00000006467;ENSRNOG00000014801;ENSRNOG00000049104;ENSRNOG00000021365;ENSRNOG00000014214;ENSRNOG00000054751;ENSRNOG00000012734;ENSRNOG00000016767;ENSRNOG00000013713;ENSRNOG00000005618;ENSRNOG00000052275;ENSRNOG00000016214;ENSRNOG00000003132;ENSRNOG00000013521;ENSRNOG00000015459;ENSRNOG00000003745;ENSRNOG00000020658;ENSRNOG00000011962;ENSRNOG00000038166;ENSRNOG00000021056;ENSRNOG00000001757;ENSRNOG00000059997;ENSRNO	Eif2b2;Exog;LOC100911256;Ybey;Rpl27a;Lmbrd2;Dcun1d1;Ggps1;LOC499240;Fmc1;H2afv;Agl;Mip;Dhfr;Heatr3;Atf3;Aarsd1;Gin1;Ptgr2;Kcnj14;Tm4sf19;Brip1;Ppard;Ccl28
ONT.5407.	ENSRNOG00000014265;ONT.9842;ONT.7978;ONT.519;ONT.40;ONT.11043;ENSRNOG00000039859;ONT.9523;ONT.9302;ONT.8892;ONT.8101;ONT.7462;ONT.7065;ONT.69;ONT.6849;ONT.6786;ONT.6314;ONT.6218;ONT.5827;ONT.5412;ONT.417;ONT.3865;ONT.3445;ONT.1688;ONT.13505;ONT.12721;ONT.1	Tnfrsf19;ONT.9842;ONT.7978;ONT.519;ONT.40;ONT.11043;Dnmt1;ONT.9523;ONT.9302;ONT.8892;ONT.8101;ONT.7462;ONT.7065;ONT.69;ONT.6849;ONT.6786;ONT.6314;ONT.6218;ONT.5827;ONT.5412;ONT.417;ONT.3865;ONT.3445;ONT.1688;ONT.13505;ONT.12721;ONT.12384;ONT.12111
ONT.6891.	ONT.8149	ONT.8149
ONT.10546	ENSRNOG00000049104;ENSRNOG00000059997;ENSRNOG00000006467;ENSRNOG00000012734;ENSRNOG00000003132;ENSRNOG00000054751;ENSRNOG00000016767;ENSRNOG00000014801;ENSRNOG00000021056;ENSRNOG00000016214;ENSRNOG00000038166;ENSRNOG00000011879;ENSRNOG00000021365;ENSRNOG00000000503;ENSRNOG00000052273;ENSRNOG00000013713;ENSRNOG00000001527;ENSRNOG00000022502;ENSRNOG00000051592;ENSRNOG00000020630;ENSRNOG00000003865;ENSRNOG00000011962;ENSRNOG00000052275;ENSRNOG00000020658;ENSRNOG00000050828;ENSRNOG00000059640;ENSRNOG00000014231;ENSRNOG00000010839;	LOC100911256;Brip1;Eif2b2;Dcun1d1;Mip;Lmbrd2;Ggps1;Exog;Kcnj14;Agl;Ptgr2;Nfat5;Ybey;Ppard;RGD1305713;LOC499240;Cd80;Nsrp1;AABR07044925.1;Il9r;Tmigd1;Gin1;H2afv;Aarsd1;Vkorc1;Ccl28;Pnoc;Krt222;Heatr3;Fmc1

ONT.9465.:	ENSRNOG00000016214;ENSRNOG00000003132;ENSRNOG00000013521;ENSRNOG00000015459;ENSRNOG00000003745;ENSRNOG00000010839;ENSRNOG00000001757;ENSRNOG00000020658;ENSRNOG00000011962;ENSRNOG00000038166;ENSRNOG00000011879;ENSRNOG00000052273;ENSRNOG00000021056;ENSRNOG00000059997;ENSRNOG00000020630;ENSRNOG00000000503;ENSRNOG0000005	Agl;Mip;Dhfr;Heatr3;Atf3;Krt222;Tm4sf19;Aarsd1;Gin1;Ptgr2;Nfat5;RGD1305713;Kcnj14;Brip1;Il9r;Ppard;Ccl28
ONT.11779	ENSRNOG00000007509	Slc1a6
ONT.8012.	ENSRNOG00000000582	Ddo
ONT.6709.	ENSRNOG00000005533	LOC686013
ONT.3801.	ENSRNOG00000060998	AABR07029467.2
ONT.10217	ONT.1731	ONT.1731
ONT.2286.	ENSRNOG00000017857;ONT.1314;ENSRNOG00000006093;ENSRNOG00000021273	LOC108348250;ONT.1314;LRRTM1;Crls1
ONT.4476.	ENSRNOG00000060528	Cacna1g
ONT.7406.	ONT.12669	ONT.12669
ONT.3457.	ENSRNOG00000024120;ONT.7628;ONT.1772;ENSRNOG0000000919	Rxfp1;ONT.7628;ONT.1772;Lctl
ONT.11587	ENSRNOG00000061763;ENSRNOG00000045736	AABR07012039.1;AABR07069238.1
ONT.4912.	ENSRNOG00000047356;ENSRNOG00000009371;ENSRNOG00000010267;ENSRNOG00000007485;ENSRNOG00000020279;ONT.2182;ENSRNOG00000032759	Abl1;LOC100909750;Klhdc10;Arfgef2;Syt11;ONT.2182;Cdx2
ONT.8028.:	ENSRNOG00000042905;ENSRNOG00000050998;ENSRNOG00000061639;ENSRNOG00000050183;ENSRNOG00000031090;ENSRNOG00000031607;ENSRNOG00000047706	RT1-T24-4;AABR07044397.1;AABR07044404.1;RT1-CE1;RT1-CE7;RT1-CE3;LOC103690108
ONT.7962.	ONT.8149;ENSRNOG00000033196;ENSRNOG00000040249;ENSRNOG00000059889;ENSRNOG00000056670;ENSRNOG00000033028	ONT.8149;AABR07004891.1;AC115420.1;AC120291.4;AABR07000902.1;LOC499584
ONT.87.1	ENSRNOG00000033196;ENSRNOG00000040249;ENSRNOG00000058178;ENSRNOG00000056670;ENSRNOG00000051678	AABR07004891.1;AC115420.1;AC113773.2;AABR07000902.1;AABR07012475.1
ONT.7101.	ONT.7555	ONT.7555
