# Supplementary material for: Effects of Maternal High-Fructose Diet on Long Non-Coding RNAs and Anxiety-like Behaviors in Offspring
Source: Int J Mol Sci. 2023 Feb 24;24(5):4460. doi: 10.3390/ijms24054460 (PMC10003385; doi:10.3390/ijms24054460)
Supplement: Supplementary file 1 [file ijms-24-04460-s001.zip › Table S4.pdf]

Table S4: The target genes of DElncRNAs.

Con vs F13%				
#lncRNA_ID	Pvalue	log2FC	Gene_ID	Symbol
ONT.11765.1	0.006445971	-0.972428178	ONT.4464	ONT.4464
ONT.119.1	0.004499037	-0.977651038	ENSRNOG000	Ppard;Lmln;Mip;Tmigd1;Gna15;Fmc1;Eif2b2;Nfat5;Dcun1d1;Polr2e;Gpx4;LOC499240;Sbno2;Rpl27a;Pnoc;Stk11;Atp5f1d;Exog;Midn;Heatr3;Cirbp;RGD1562114;Efna2;Agl;Ggps1;Mdp1;Il9r;Aarsd1;Kcnj14;Ybey;Nsrp1;Cbarp;Smc5;Ptgr2;LOC100911256;Vkorc1;AABR07044925.1;Lmbrd2;Ccl28;Brip1;ONT.11763;ONT.121
ONT.13539.1	0.000268271	1.166809638	ENSRNOG000	AABR07002868.1;ONT.1097
ONT.14125.1	0.040219845	-0.644675525	ENSRNOG000	Slc18b1;Rps12
ONT.252.2	0.029552328	0.77416135	ENSRNOG000	Tarm1;Oscar;Cacng8;Cacng6;ONT.972;ONT.973;ONT.974
ONT.4463.11	0.006981091	-0.684037284	ENSRNOG000	Ankar;Osgepl1;Ormdl1
ONT.7138.4	0.005166759	1.167076573	ENSRNOG000	Nes;Bcan;Hapln2;Naxe;Crabp2;Iqgap3;AABR07072748.1;ONT.7602
ONT.897.1	0.034007634	-0.586571594	ENSRNOG000	Rpl36a;Gla;Hnrnph2;Armcx3;Armcx6;Armcx1;LOC501618

Con vs F40%				
#lncRNA_ID	Pvalue	log2FC	Gene_ID	Symbol
ONT.11141.1	0.0137758	-0.773269862	ENSRNOG000	AABR07064224.1;Foxg1;ONT.11508
ONT.11141.2	0.027684626	-0.689685084	ENSRNOG000	AABR07064224.1;Foxg1;ONT.11508
ONT.119.1	0.025194148	-0.666099521	ENSRNOG000	Tarm1;Oscar;Cacng8;Cacng6;ONT.972;ONT.973;ONT.974
ONT.12048.6	0.011098438	-0.638670794	ENSRNOG000	Tubgcp6;Mapk11;Trabd;Mov10l1;Mapk12;Hdac10;Selenoo
ONT.1569.4	0.029043746	0.610950678	ENSRNOG000	Rtn3;RGD1560108
ONT.252.2	0.027732556	0.892528369	ENSRNOG000	AABR07002868.1;ONT.1097
ONT.258.1	0.000490034	0.837815034	ENSRNOG000	Tyrobp;Hcst;Aplp1;Kirrel2;Nphs1;Hspb6;Lin37;Psenen;Igflr1;Cox6b1;Upk1a;U2af1l4;Proser3;Nfkbid;Zbtb32;Kmt2b;ONT.1109
ONT.5511.1	0.047857674	-1.01329447	ENSRNOG000	Cbr4;ONT.5300
ONT.5511.2	0.04757914	-0.734908179	ENSRNOG000	Cbr4;ONT.19;ONT.5300
ONT.5545.1	0.014029193	0.723262578	ENSRNOG000	Cldn23
ONT.5636.1	0.02743975	-0.779773942	ENSRNOG000	Fbxl21;Lect2;Tgfbi;ONT.1149
ONT.5939.1	0.003205036	-1.270945382	ONT.5723	ONT.5723
ONT.7138.4	0.047749449	0.682542952	ENSRNOG000	Nes;Bcan;Hapln2;Naxe;Crabp2;Iqgap3;AABR07072748.1;ONT.7602
ONT.7803.1	0.04304278	0.631262657	ONT.7351	ONT.7351
