# Supplementary material for: Effects of Maternal High-Fructose Diet on Long Non-Coding RNAs and Anxiety-like Behaviors in Offspring
Source: Int J Mol Sci. 2023 Feb 24;24(5):4460. doi: 10.3390/ijms24054460 (PMC10003385; doi:10.3390/ijms24054460)
Supplement: Supplementary file 1 [file ijms-24-04460-s001.zip › Table S5.pdf]

Table S5: The full KEGG pathway enrichment results of cis co-expression clusters in F13%.

Term	Count	GeneID	Pvalue	group
positive regulation of transcript	16	ENSRNOG00000000438;ENSRNOG00000002332;ENSRNOG00000003443;ENSRNOG00000004642;ENSRNOG00000006876;ENSRNOG00000008282;ENSRNOG00000008785;ENSRNOG00000009145;ENSRNOG00000015822;ENSRNOG00000016299;ENSRNOG00000018532;ENSRNOG00000020060;ENSRNOG00000020369;ENSRNOG00000027557;ENSRNOG00000032539;ENSRNO	1.50E-15	BP
protein ubiquitination	38	ENSRNOG00000000036;ENSRNOG00000000327;ENSRNOG00000000438;ENSRNOG00000001283;ENSRNOG00000001844;ENSRNOG00000001878;ENSRNOG00000002214;ENSRNOG00000003258;ENSRNOG00000003334;ENSRNOG00000004193;ENSRNOG00000004218;ENSRNOG00000005215;ENSRNOG00000005342;ENSRNOG00000005371;ENSRNOG00000006224;ENSRNOG00000006335;ENSRNOG00000006515;ENSRNOG00000007981;ENSRNOG00000008421;ENSRNOG00000010453;ENSRNOG00000010959;ENSRNOG00000015418;ENSRNOG00000016422;ENSRNOG00000017365;ENSRNOG00000018867;ENSRNOG00000019390;ENSRNOG00000020088;ENSRNOG00000020105;ENSRNOG00000020302;ENSRNOG00000020880;ENSRNOG00000022772;ENSRNOG00000025868;ENSRNOG00000029441;ENSRNOG00000031100;ENSRNOG00000032419;ENSRNOG00000033372;ENSRNO	2.00E-08	BP
response to drug	8	ENSRNOG00000011648;ENSRNOG00000014475;ENSRNOG00000014656;ENSRNOG00000020129;ENSRNOG00000020369;ENSRNOG00000029096;ENSRNOG00000029811;ENSRNOG00000062101	7.40E-08	BP
angiogenesis	9	ENSRNOG00000004089;ENSRNOG00000008785;ENSRNOG00000010841;ENSRNOG00000011648;ENSRNOG00000016299;ENSRNOG00000039668;ENSRNOG00000048812;ENSRNOG00000055597;ENSRNO	2.00E-06	BP
protein autophosphorylation	2	ENSRNOG00000003742;ENSRNOG00000062101	2.50E-06	BP

nucleus	91	ENSRNOG00000000327;ENSRNOG00000000438;ENSRNOG00000001283;ENSRNOG00000002105;ENSRNOG00000002163;ENSRNOG00000002214;ENSRNOG00000002332;ENSRNOG00000002506;ENSRNOG00000003258;ENSRNOG00000003284;ENSRNOG00000003443;ENSRNOG00000003742;ENSRNOG00000004459;ENSRNOG00000004474;ENSRNOG00000004642;ENSRNOG00000005271;ENSRNOG00000005342;ENSRNOG00000005506;ENSRNOG00000005513;ENSRNOG00000005890;ENSRNOG00000006001;ENSRNOG00000006118;ENSRNOG00000006122;ENSRNOG00000006515;ENSRNOG00000006876;ENSRNOG00000007922;ENSRNOG00000008282;ENSRNOG00000008785;ENSRNOG00000009052;ENSRNOG00000009145;ENSRNOG00000010267;ENSRNOG00000010453;ENSRNOG00000010760;ENSRNOG00000011648;ENSRNOG00000011859;ENSRNOG00000012364;ENSRNOG00000012727;ENSRNOG00000014997;ENSRNOG00000015768;ENSRNOG00000015822;ENSRNOG00000016299;ENSRNOG00000016478;	2.80E-22	CC
nucleoplasm	19	ENSRNOG00000002105;ENSRNOG00000002214;ENSRNOG00000003258;ENSRNOG00000003284;ENSRNOG00000003742;ENSRNOG00000005506;ENSRNOG00000005513;ENSRNOG00000006876;ENSRNOG00000008785;ENSRNOG00000009145;ENSRNOG00000010453;ENSRNOG00000016299;ENSRNOG00000017342;ENSRNOG00000020060;ENSRNOG00000020274;ENSRNOG00000031927;ENSRNOG00000053787;ENSRNOG00000054251;ENS	1.20E-17	CC
Cul3-RING ubiquitin ligase co	30	ENSRNOG00000000036;ENSRNOG00000001844;ENSRNOG00000001878;ENSRNOG00000002214;ENSRNOG00000003334;ENSRNOG00000004193;ENSRNOG00000004218;ENSRNOG00000005215;ENSRNOG00000005371;ENSRNOG00000006224;ENSRNOG00000006335;ENSRNOG00000006515;ENSRNOG00000007981;ENSRNOG00000008421;ENSRNOG00000010453;ENSRNOG00000010959;ENSRNOG00000015418;ENSRNOG00000016422;ENSRNOG00000017365;ENSRNOG00000018867;ENSRNOG00000019390;ENSRNOG00000020088;ENSRNOG00000020105;ENSRNOG00000020302;ENSRNOG00000020880;ENSRNOG00000025868;ENSRNOG00000029441;ENSRNOG00000031100;	1.80E-15	CC

perinuclear region of cytoplasm	14	ENSRNOG00000002332;ENSRNOG00000003284;ENSRNOG00000005299;ENSRNOG00000005890;ENSRNOG00000006122;ENSRNOG00000010453;ENSRNOG00000012727;ENSRNOG00000012748;ENSRNOG00000014997;ENSRNOG00000018524;ENSRNOG00000022331;ENSRNOG00000030212;ENSRNOG00000050570;ENSRNOG00000058316	1.10E-14	CC
neuronal cell body	11	ENSRNOG00000005299;ENSRNOG00000010938;ENSRNOG00000011572;ENSRNOG00000011648;ENSRNOG00000011859;ENSRNOG00000014475;ENSRNOG00000015418;ENSRNOG00000016478;ENSRNOG00000020302;ENSRNOG00000031100;ENSRNOG00000033372	1.00E-13	CC
ATP binding	16	ENSRNOG00000001214;ENSRNOG00000002105;ENSRNOG00000002506;ENSRNOG00000003472;ENSRNOG00000003742;ENSRNOG00000005299;ENSRNOG00000005530;ENSRNOG00000008625;ENSRNOG00000027911;ENSRNOG00000029096;ENSRNOG00000038720;ENSRNOG00000040266;ENSRNOG00000042353;ENSRNOG00000050277;ENSRNOG00000050570;ENSRNO	9.90E-19	MF
zinc ion binding	19	ENSRNOG00000000438;ENSRNOG00000004089;ENSRNOG00000004642;ENSRNOG00000008543;ENSRNOG00000011989;ENSRNOG00000012364;ENSRNOG00000017342;ENSRNOG00000018532;ENSRNOG00000021896;ENSRNOG00000022417;ENSRNOG00000022772;ENSRNOG00000040208;ENSRNOG00000043311;ENSRNOG00000047028;ENSRNOG00000047940;ENSRNOG00000047957;ENSRNOG00000054605;ENSRNOG00000062101;ONT	5.50E-14	MF
protein homodimerization activ	8	ENSRNOG00000007310;ENSRNOG00000010453;ENSRNOG00000026870;ENSRNOG00000029441;ENSRNOG00000029811;ENSRNOG00000050570;ENSRNOG00000058714;ENSRNOG00000061739	9.00E-14	MF
identical protein binding	20	ENSRNOG00000000438;ENSRNOG00000001214;ENSRNOG00000001926;ENSRNOG00000004193;ENSRNOG00000007310;ENSRNOG00000010263;ENSRNOG00000010453;ENSRNOG00000014997;ENSRNOG00000016496;ENSRNOG00000018570;ENSRNOG00000026870;ENSRNOG00000029441;ENSRNOG00000029811;ENSRNOG00000047957;ENSRNOG00000050123;ENSRNOG00000050570;ENSRNOG00000054495;ENSRNOG00000055138;ENSRNOG00000058714;ENSRNOG000	5.00E-12	MF

metal ion binding	56	ENSRNOG00000000017;ENSRNOG00000000438;ENSRNOG00000001214;ENSRNOG00000002105;ENSRNOG00000002163;ENSRNOG00000003443;ENSRNOG00000003472;ENSRNOG00000004089;ENSRNOG00000004642;ENSRNOG00000005342;ENSRNOG00000006118;ENSRNOG00000008543;ENSRNOG00000008625;ENSRNOG00000008785;ENSRNOG00000009145;ENSRNOG00000010091;ENSRNOG00000010730;ENSRNOG00000011989;ENSRNOG00000012364;ENSRNOG00000015822;ENSRNOG00000016299;ENSRNOG00000017342;ENSRNOG00000018532;ENSRNOG00000018864;ENSRNOG00000019479;ENSRNOG00000020129;ENSRNOG00000021896;ENSRNOG00000022417;ENSRNOG00000022772;ENSRNOG00000023356;ENSRNOG00000025651;ENSRNOG00000026176;ENSRNOG00000027557;ENSRNOG00000029237;ENSRNOG00000031665;ENSRNOG00000033694;ENSRNOG00000038202;ENSRNOG00000039057;ENSRNOG00000040208;ENSRNOG00000042496;[illegible]	5.50E-12	MF

Term	Count	GeneID	Pvalue	group
positive regulation of transcript	10	ENSRNOG00000002389;ENSRNOG00000002671;ENSRNOG00000003359;ENSRNOG00000004814;ENSRNOG00000006069;ENSRNOG00000006355;ENSRNOG00000018803;ENSRNOG00000022249;ENSRNOG00000025923;ENSRNOG0000005	7.90E-14	BP

translation	121	ENSRNOG00000001611;ENSRNOG00000002116;ENSRNOG00000002178;ENSRNOG00000003149;ENSRNOG00000003158;ENSRNOG00000004107;ENSRNOG00000004196;ENSRNOG00000004214;ENSRNOG00000004426;ENSRNOG00000004814;ENSRNOG00000005975;ENSRNOG00000006027;ENSRNOG00000006069;ENSRNOG00000006727;ENSRNOG00000006898;ENSRNOG00000006939;ENSRNOG00000008546;ENSRNOG00000008555;ENSRNOG00000009078;ENSRNOG00000009297;ENSRNOG00000009439;ENSRNOG00000010363;ENSRNOG00000010746;ENSRNOG00000010855;ENSRNOG00000010912;ENSRNOG00000011955;ENSRNOG00000012650;ENSRNOG00000012738;ENSRNOG00000013508;ENSRNOG00000014641;ENSRNOG00000015989;ENSRNOG00000016387;ENSRNOG00000016580;ENSRNOG00000017127;ENSRNOG00000017158;ENSRNOG00000018320;ENSRNOG00000018471;ENSRNOG00000018630;ENSRNOG00000018774;ENSRNOG00000018795;ENSRNOG00000019106;ENSRNOG00000019578;	6.60E-08	BP
response to drug	6	ENSRNOG00000002693;ENSRNOG00000006589;ENSRNOG00000009439;ENSRNOG00000018795;ENSRNOG00000020676;ENSRNOG000	3.80E-07	BP
protein autophosphorylation	8	ENSRNOG00000002671;ENSRNOG00000006027;ENSRNOG00000006069;ENSRNOG00000018414;ENSRNOG00000018839;ENSRNOG00000020835;ENSRNOG00000048315;	8.80E-07	BP
brain development	28	ENSRNOG00000001227;ENSRNOG00000001551;ENSRNOG00000002693;ENSRNOG00000003359;ENSRNOG00000004426;ENSRNOG00000006030;ENSRNOG00000006589;ENSRNOG00000010872;ENSRNOG00000014963;ENSRNOG00000018414;ENSRNOG00000018803;ENSRNOG00000018839;ENSRNOG00000019780;ENSRNOG00000019895;ENSRNOG00000020058;ENSRNOG00000026116;ENSRNOG00000034161;ENSRNOG00000034246;ENSRNOG00000037793;ENSRNOG00000038951;ENSRNOG00000042978;ENSRNOG00000046889;ENSRNOG00000048174;ENSRNOG00000050090;ENSRNOG00000050206;ENSRNOG00000051650;ENSRNOG000	1.40E-06	BP

nucleus	144	ENSRNOG00000000490;ENSRNOG00000000622;ENSRNOG00000001212;ENSRNOG00000001227;ENSRNOG00000002320;ENSRNOG00000002389;ENSRNOG00000002671;ENSRNOG00000002693;ENSRNOG00000002721;ENSRNOG00000003359;ENSRNOG00000003542;ENSRNOG00000003626;ENSRNOG00000003712;ENSRNOG00000004107;ENSRNOG00000004426;ENSRNOG00000004814;ENSRNOG00000004996;ENSRNOG00000005062;ENSRNOG00000005686;ENSRNOG00000005828;ENSRNOG00000005975;ENSRNOG00000006355;ENSRNOG00000006494;ENSRNOG00000006532;ENSRNOG00000006559;ENSRNOG00000006589;ENSRNOG00000006644;ENSRNOG00000008461;ENSRNOG00000008604;ENSRNOG00000009117;ENSRNOG00000009288;ENSRNOG00000009297;ENSRNOG00000009439;ENSRNOG00000009823;ENSRNOG00000010170;ENSRNOG00000010363;ENSRNOG00000010428;ENSRNOG00000010872;ENSRNOG00000011015;ENSRNOG00000011728;ENSRNOG00000012266;ENSRNOG00000012985;	7.10E-20	CC
nucleoplasm	50	ENSRNOG00000000622;ENSRNOG00000001227;ENSRNOG00000002389;ENSRNOG00000002721;ENSRNOG00000003359;ENSRNOG00000003626;ENSRNOG00000003712;ENSRNOG00000004814;ENSRNOG00000004996;ENSRNOG00000006355;ENSRNOG00000006559;ENSRNOG00000006589;ENSRNOG00000008604;ENSRNOG00000010170;ENSRNOG00000011015;ENSRNOG00000011728;ENSRNOG00000013039;ENSRNOG00000013508;ENSRNOG00000017445;ENSRNOG00000018137;ENSRNOG00000018408;ENSRNOG00000018803;ENSRNOG00000018998;ENSRNOG00000019097;ENSRNOG00000019249;ENSRNOG00000019293;ENSRNOG00000019798;ENSRNOG00000021248;ENSRNOG00000022249;ENSRNOG00000022664;ENSRNOG00000025923;ENSRNOG00000026649;ENSRNOG00000027906;ENSRNOG00000029512;ENSRNOG00000030225;ENSRNOG00000031127;ENSRNOG00000034182;ENSRNOG00000039249;ENSRNOG00000048470;ENSRNOG00000048562;ENSRNOG00000048736;ENSRNOG00000049229;	7.20E-19	CC

cytosol	111	ENSRNOG00000000490;ENSRNOG00000001212;ENSRNOG00000001611;ENSRNOG00000002116;ENSRNOG00000002178;ENSRNOG00000002693;ENSRNOG00000003158;ENSRNOG00000003359;ENSRNOG00000003597;ENSRNOG00000004107;ENSRNOG00000004214;ENSRNOG00000004426;ENSRNOG00000004992;ENSRNOG00000005828;ENSRNOG00000005975;ENSRNOG00000006027;ENSRNOG00000006130;ENSRNOG00000008604;ENSRNOG00000009288;ENSRNOG00000009439;ENSRNOG00000009870;ENSRNOG00000010746;ENSRNOG00000011014;ENSRNOG00000011955;ENSRNOG00000012004;ENSRNOG00000012266;ENSRNOG00000013508;ENSRNOG00000014641;ENSRNOG00000014829;ENSRNOG00000015277;ENSRNOG00000015844;ENSRNOG00000016387;ENSRNOG00000016580;ENSRNOG00000017127;ENSRNOG00000018091;ENSRNOG00000018320;ENSRNOG00000018471;ENSRNOG00000018630;ENSRNOG00000018774;ENSRNOG00000018795;ENSRNOG00000018839;ENSRNOG00000019106;	9.80E-18	CC
neuronal cell body	19	ENSRNOG00000007281;ENSRNOG00000009779;ENSRNOG00000010765;ENSRNOG00000011076;ENSRNOG00000015406;ENSRNOG00000018110;ENSRNOG00000018993;ENSRNOG00000019400;ENSRNOG00000028545;ENSRNOG00000036701;ENSRNOG00000046366;ENSRNOG00000047014;ENSRNOG00000051480;ENSRNOG00000053288;ENSRNOG00000053452;ENSRNO	3.10E-15	CC

extracellular vesicular exosome	112	ENSRNOG00000000490;ENSRNOG00000000622;ENSRNOG00000001611;ENSRNOG00000001640;ENSRNOG00000001806;ENSRNOG00000002116;ENSRNOG00000002376;ENSRNOG00000002671;ENSRNOG00000002693;ENSRNOG00000002721;ENSRNOG00000003597;ENSRNOG00000003626;ENSRNOG00000004107;ENSRNOG00000004132;ENSRNOG00000004214;ENSRNOG00000004426;ENSRNOG00000004814;ENSRNOG00000004992;ENSRNOG00000005355;ENSRNOG00000005828;ENSRNOG00000005975;ENSRNOG00000006028;ENSRNOG00000006130;ENSRNOG00000006532;ENSRNOG00000006589;ENSRNOG00000009439;ENSRNOG00000009856;ENSRNOG00000010170;ENSRNOG00000010872;ENSRNOG00000011825;ENSRNOG00000011955;ENSRNOG00000012953;ENSRNOG00000013505;ENSRNOG00000013508;ENSRNOG00000014568;ENSRNOG00000014816;ENSRNOG00000014963;ENSRNOG00000015844;ENSRNOG00000016387;ENSRNOG00000016660;ENSRNOG00000017127;ENSRNOG00000017209;	3.00E-14	CC
structural constituent of riboso	110	ENSRNOG00000000490;ENSRNOG00000001611;ENSRNOG00000002116;ENSRNOG00000002178;ENSRNOG00000003158;ENSRNOG00000004107;ENSRNOG00000004196;ENSRNOG00000004214;ENSRNOG00000004426;ENSRNOG00000005975;ENSRNOG00000006898;ENSRNOG00000006939;ENSRNOG00000008546;ENSRNOG00000008555;ENSRNOG00000009078;ENSRNOG00000010363;ENSRNOG00000010746;ENSRNOG00000010855;ENSRNOG00000010912;ENSRNOG00000011955;ENSRNOG00000012650;ENSRNOG00000013508;ENSRNOG00000014641;ENSRNOG00000015989;ENSRNOG00000016387;ENSRNOG00000016580;ENSRNOG00000017127;ENSRNOG00000018320;ENSRNOG00000018471;ENSRNOG00000018774;ENSRNOG00000018795;ENSRNOG00000019106;ENSRNOG00000019578;ENSRNOG00000019734;ENSRNOG00000019970;ENSRNOG00000019974;ENSRNOG00000020354;ENSRNOG00000020982;ENSRNOG00000022234;ENSRNOG00000022609;ENSRNOG00000023344;ENSRNOG00000023385;	7.20E-18	MF

ATP binding	22	ENSRNOG00000002671;ENSRNOG00000002693;ENSRNOG00000003149;ENSRNOG00000005686;ENSRNOG00000006027;ENSRNOG00000006069;ENSRNOG00000010170;ENSRNOG00000010872;ENSRNOG00000017445;ENSRNOG00000018414;ENSRNOG00000018839;ENSRNOG00000019097;ENSRNOG00000020835;ENSRNOG00000022753;ENSRNOG00000025286;ENSRNOG00000030225;ENSRNOG00000043201;ENSRNOG00000048315;ENSRNOG00000051624;ENSRNOG000	3.50E-17	MF
zinc ion binding	17	ENSRNOG00000004132;ENSRNOG00000004996;ENSRNOG00000006559;ENSRNOG00000006644;ENSRNOG00000012266;ENSRNOG00000016631;ENSRNOG00000019293;ENSRNOG00000019682;ENSRNOG00000021891;ENSRNOG00000022249;ENSRNOG00000022664;ENSRNOG00000025923;ENSRNOG00000026649;ENSRNOG00000040205;ENSRNOG00000048562;ENSRNOG00000054513;ENSRNOG0000005	1.50E-12	MF
protein homodimerization activ	11	ENSRNOG00000001227;ENSRNOG00000013039;ENSRNOG00000018414;ENSRNOG00000018471;ENSRNOG00000018839;ENSRNOG00000019798;ENSRNOG00000019891;ENSRNOG00000020279;ENSRNOG00000037897;ENSRNOG0000004	1.60E-12	MF
identical protein binding	19	ENSRNOG00000001227;ENSRNOG00000002693;ENSRNOG00000006069;ENSRNOG00000013039;ENSRNOG00000018414;ENSRNOG00000018471;ENSRNOG00000018630;ENSRNOG00000018839;ENSRNOG00000019798;ENSRNOG00000019891;ENSRNOG00000020279;ENSRNOG00000026649;ENSRNOG00000030963;ENSRNOG00000037897;ENSRNOG00000048199;ENSRNOG00000050450;ENSRNOG0000005	1.70E-11	MF

Term	Count	GneID	Pvalue	group
positive regulation of transcript	6	ENSRNOG00000001892;ENSRNOG00000002979;ENSRNOG00000006587;ENSRNOG00000008277;ENSRNOG00000014629;ENSRNOG000	1.30E-15	BP
protein ubiquitination	11	ENSRNOG00000000611;ENSRNOG00000001470;ENSRNOG00000005758;ENSRNOG00000012333;ENSRNOG00000012524;ENSRNOG00000015385;ENSRNOG00000019529;ENSRNOG00000042274;ENSRNOG00000043106;ENSRNOG00000046497;ENSRNOG00000049219	7.40E-07	BP
brain development	10	ENSRNOG00000001068;ENSRNOG00000001139;ENSRNOG00000002979;ENSRNOG00000004426;ENSRNOG00000009266;ENSRNOG00000034246;ENSRNOG00000047211;ENSRNOG00000048172;ENSRNOG00000050784;ENSRNOG0000005	7.50E-07	BP

response to drug	3	ENSRNOG00000008277;ENSRNOG00000047211;ENSRNOG0000005	8.30E-07	BP
neuron migration	2	ENSRNOG00000042274;ENSRNOG00000047211	9.30E-07	BP
nucleus	61	ENSRNOG00000001068;ENSRNOG00000001139;ENSRNOG00000001309;ENSRNOG00000001470;ENSRNOG00000001632;ENSRNOG00000001892;ENSRNOG00000002979;ENSRNOG00000003833;ENSRNOG00000004206;ENSRNOG00000004426;ENSRNOG00000004474;ENSRNOG00000005258;ENSRNOG00000005486;ENSRNOG00000006587;ENSRNOG00000006929;ENSRNOG00000008277;ENSRNOG00000008960;ENSRNOG00000009103;ENSRNOG00000009266;ENSRNOG00000010189;ENSRNOG00000010363;ENSRNOG00000010685;ENSRNOG00000010873;ENSRNOG00000012524;ENSRNOG00000014463;ENSRNOG00000014629;ENSRNOG00000014765;ENSRNOG00000015385;ENSRNOG00000015753;ENSRNOG00000016313;ENSRNOG00000016580;ENSRNOG00000019027;ENSRNOG00000019178;ENSRNOG00000019310;ENSRNOG00000019507;ENSRNOG00000019565;ENSRNOG00000019721;ENSRNOG00000020683;ENSRNOG00000021224;ENSRNOG00000021984;ENSRNOG00000022934;ENSRNOG00000026905;	2.30E-21	CC
nucleoplasm	22	ENSRNOG00000001139;ENSRNOG00000001470;ENSRNOG00000003833;ENSRNOG00000005258;ENSRNOG00000010189;ENSRNOG00000010685;ENSRNOG00000014463;ENSRNOG00000019027;ENSRNOG00000019178;ENSRNOG00000019310;ENSRNOG00000019507;ENSRNOG00000019565;ENSRNOG00000020683;ENSRNOG00000021224;ENSRNOG00000022934;ENSRNOG00000026905;ENSRNOG00000029028;ENSRNOG00000033790;ENSRNOG00000049219;ENSRNOG00000052445;ENSRNOG00000058461;	9.40E-20	CC
neuronal cell body	7	ENSRNOG00000004206;ENSRNOG00000004426;ENSRNOG00000034246;ENSRNOG00000042274;ENSRNOG00000047211;ENSRNOG00000048172;ENSRNOG00000055401	6.10E-17	CC
perinuclear region of cytoplasm	8	ENSRNOG00000001470;ENSRNOG00000001813;ENSRNOG00000008277;ENSRNOG00000014765;ENSRNOG00000015385;ENSRNOG00000019178;ENSRNOG00000047088;ENSRNOG00000048172	1.40E-14	CC
Golgi membrane	8	ENSRNOG00000001068;ENSRNOG00000001813;ENSRNOG00000005331;ENSRNOG00000014765;ENSRNOG00000015051;ENSRNOG00000047088;ENSRNOG00000048172;ENSRNOG00000061832	5.20E-14	CC

ATP binding	10	ENSRNOG00000000611;ENSRNOG00000001309;ENSRNOG00000001470;ENSRNOG00000004160;ENSRNOG00000006962;ENSRNOG00000015385;ENSRNOG00000029028;ENSRNOG00000033790;ONT.1411	1.00E-18	MF
zinc ion binding	5	ENSRNOG00000001470;ENSRNOG00000014629;ENSRNOG00000046497;ENSRNOG00000049219;ENSRNOG00000052445	1.50E-13	MF
protein homodimerization activ	9	ENSRNOG00000001139;ENSRNOG00000001813;ENSRNOG00000001892;ENSRNOG00000004160;ENSRNOG00000005331;ENSRNOG00000010685;ENSRNOG00000019565;ENSRNOG00000046585;ENSRNO	1.50E-13	MF
identical protein binding	13	ENSRNOG00000001139;ENSRNOG00000001813;ENSRNOG00000001892;ENSRNOG00000004160;ENSRNOG00000005331;ENSRNOG00000010685;ENSRNOG00000019565;ENSRNOG00000033835;ENSRNOG00000046227;ENSRNOG00000046497;ENSRNOG00000046585;ENSRNOG00000052025;ENSRNOG000	1.70E-12	MF
metal ion binding	23	ENSRNOG00000001309;ENSRNOG00000001470;ENSRNOG00000001729;ENSRNOG00000003329;ENSRNOG00000003833;ENSRNOG00000004160;ENSRNOG00000004206;ENSRNOG00000004426;ENSRNOG00000005486;ENSRNOG00000006962;ENSRNOG00000012524;ENSRNOG00000014629;ENSRNOG00000015385;ENSRNOG00000019721;ENSRNOG00000026905;ENSRNOG00000029028;ENSRNOG00000034246;ENSRNOG00000043225;ENSRNOG00000046497;ENSRNOG00000046527;ENSRNOG00000049219;ENSRNOG00000050473;ENSRNO	1.90E-11	MF

#Kegg_pathway-yellow	ko_id	Cluster_fre	GeneID	P-value	rich_factor
Ribosome	ko03010	17 out of 54	ENSRNOG00000004426;ENSRNOG00000008555;ENSRNOG00000010164;ENSRNOG00000010189;ENSRNOG00000014179;ENSRNOG00000016580;ENSRNOG00000016961;ENSRNOG00000022934;ENSRNOG00000024845;ENSRNOG00000027503;ENSRNOG00000028939;ENSRNOG00000034246;ENSRNOG00000042886;ENSRNOG00000049921;ENSRNOG00000050473;ENSRNO	1.14233E-09	5.956195151
Wnt signaling pathway	ko04310	5 out of 54	ENSRNOG00000001068;ENSRNOG00000009745;ENSRNOG00000016313;ENSRNOG00000047211;ENSRNOG00000048172	0.002863805	5.101797704
Ubiquitin mediated proteolysi	ko04120	5 out of 54	ENSRNOG00000000611;ENSRNOG00000004426;ENSRNOG00000032690;ENSRNOG00000034246;ONT	0.00316413	4.985185185
Axon guidance	ko04360	5 out of 54	ENSRNOG00000001068;ENSRNOG00000001470;ENSRNOG00000009745;ENSRNOG00000047211;ENSRNOG00000048172	0.004495266	4.591617934
VEGF signaling pathway	ko04370	3 out of 54	ENSRNOG00000001068;ENSRNOG00000009745;ENSRNOG0000004	0.005065598	8.581056466
Endocytosis	ko04144	6 out of 54	ENSRNOG00000001086;ENSRNOG00000015753;ENSRNOG00000033835;ENSRNOG00000046585;ENSRNOG00000047088;ONT.14104	0.005546695	3.673294347
Bacterial invasion of epithelia	ko05100	3 out of 54	ENSRNOG00000001068;ENSRNOG00000033835;ENSRNOG0000005	0.009655073	6.797979798
Mitophagy - animal	ko04137	3 out of 54	ENSRNOG00000004426;ENSRNOG00000015385;ENSRNOG0000003	0.010714691	6.543055556
Yersinia infection	ko05135	4 out of 54	ENSRNOG00000001068;ENSRNOG00000001470;ENSRNOG00000048172;ENSRNOG00000059705	0.01637997	4.081438163
B cell receptor signaling pathv	ko04662	3 out of 54	ENSRNOG00000001068;ENSRNOG00000009745;ENSRNOG0000004	0.024383774	4.80224261
MAPK signaling pathway	ko04010	5 out of 54	ENSRNOG00000001068;ENSRNOG00000008960;ENSRNOG00000009745;ENSRNOG00000029028;ENSRNOG00000048172	0.031717739	2.814217443
Kaposi sarcoma-associated he	ko05167	4 out of 54	ENSRNOG00000001068;ENSRNOG00000004426;ENSRNOG00000009745;ENSRNOG00000034246	0.037936381	3.143810477
Cholesterol metabolism	ko04979	2 out of 54	ENSRNOG00000005331;ENSRNOG00000014765	0.038162555	6.462277092
Natural killer cell mediated cy	ko04650	3 out of 54	ENSRNOG00000001068;ENSRNOG00000009745;ENSRNOG0000004	0.040475052	3.935672515
Regulation of actin cytoskelet	ko04810	4 out of 54	ENSRNOG00000001068;ENSRNOG00000001470;ENSRNOG00000008960;ENSRNOG00000048172	0.042889268	3.021324355
Human immunodeficiency vir	ko05170	4 out of 54	ENSRNOG00000001068;ENSRNOG00000001470;ENSRNOG00000009745;ENSRNOG00000048172	0.054498917	2.791703704
Ras signaling pathway	ko04014	4 out of 54	ENSRNOG00000001068;ENSRNOG00000008960;ENSRNOG00000046497;ENSRNOG00000048172	0.05714183	2.747739866
Long-term potentiation	ko04720	2 out of 54	ENSRNOG00000009745;ENSRNOG00000036827	0.060799747	4.985185185
Adherens junction	ko04520	2 out of 54	ENSRNOG00000001068;ENSRNOG00000048172	0.080094989	4.25564589
Pancreatic cancer	ko05212	2 out of 54	ENSRNOG00000001068;ENSRNOG00000048172	0.081780315	4.204373048
Chemokine signaling pathway	ko04062	3 out of 54	ENSRNOG00000001068;ENSRNOG00000048172;ENSRNOG0000005	0.089353346	2.829429429

Fc epsilon RI signaling pathwa	ko04664	2 out of 54	ENSRNOG00000001068;ENSRNOG00000048172	0.102817137	3.673294347
Selenocompound metabolism	ko00450	1 out of 54	ONT.1417	0.10354939	9.183235867
Colorectal cancer	ko05210	2 out of 54	ENSRNOG00000001068;ENSRNOG00000048172	0.110132689	3.524878414
Choline metabolism in cancer	ko05231	2 out of 54	ENSRNOG00000001068;ENSRNOG00000048172	0.113841585	3.455078841
Rap1 signaling pathway	ko04015	3 out of 54	ENSRNOG00000001068;ENSRNOG00000008960;ENSRNOG0000004	0.140645526	2.305922663
Fc gamma R-mediated phagoc	ko04666	2 out of 54	ENSRNOG00000001068;ENSRNOG00000001470	0.158539976	2.814217443
Osteoclast differentiation	ko04380	2 out of 54	ENSRNOG00000001068;ENSRNOG00000009745	0.166627803	2.726273148
Pentose phosphate pathway	ko00030	1 out of 54	ENSRNOG00000004160	0.168257396	5.452546296
AMPK signaling pathway	ko04152	2 out of 54	ENSRNOG00000001309;ENSRNOG00000047088	0.16866139	2.705139248
Viral myocarditis	ko05416	2 out of 54	ENSRNOG00000001068;ENSRNOG00000048172	0.16866139	2.705139248
Glyoxylate and dicarboxylate	ko00630	1 out of 54	ENSRNOG00000009536	0.17304058	5.287317621
Human cytomegalovirus infec	ko05163	3 out of 54	ENSRNOG00000001068;ENSRNOG00000009745;ENSRNOG0000004	0.173581358	2.085436034
Sphingolipid signaling pathwa	ko04071	2 out of 54	ENSRNOG00000001068;ENSRNOG00000048172	0.178893344	2.604201216
Amyotrophic lateral sclerosis	ko05014	4 out of 54	ENSRNOG00000001068;ENSRNOG00000005331;ENSRNOG00000009745;ENSRNOG00000015385	0.202604064	1.719029374
Other types of O-glycan biosy	ko00514	1 out of 54	ENSRNOG00000001729	0.223906071	3.965488215
Breast cancer	ko05224	2 out of 54	ENSRNOG00000008960;ENSRNOG00000047211	0.224886176	2.23694207
Fluid shear stress and atherosc	ko05418	2 out of 54	ENSRNOG00000001068;ENSRNOG00000048172	0.224886176	2.23694207
Oxytocin signaling pathway	ko04921	2 out of 54	ENSRNOG00000001309;ENSRNOG00000009745	0.22700377	2.222694032
Gastric cancer	ko05226	2 out of 54	ENSRNOG00000008960;ENSRNOG00000047211	0.22700377	2.222694032
Homologous recombination	ko03440	1 out of 54	ONT.14100	0.228374942	3.877366255
Parkinson disease	ko05012	3 out of 54	ENSRNOG00000004426;ENSRNOG00000015385;ENSRNOG0000003	0.229573514	1.811226451
Carbon metabolism	ko01200	2 out of 54	ENSRNOG00000004160;ENSRNOG00000009536	0.235489421	2.16747182
Basal transcription factors	ko03022	1 out of 54	ENSRNOG00000019178	0.241630562	3.635030864
Amino sugar and nucleotide su	ko00520	1 out of 54	ENSRNOG00000013816	0.250343208	3.48962963
Aminoacyl-tRNA biosynthesis	ko00970	1 out of 54	ENSRNOG00000024460	0.250343208	3.48962963
Tight junction	ko04530	2 out of 54	ENSRNOG00000001068;ONT.1410	0.258914638	2.028854436
Cysteine and methionine meta	ko00270	1 out of 54	ENSRNOG00000013409	0.267474794	3.231138546
Ferroptosis	ko04216	1 out of 54	ENSRNOG00000013409	0.275895898	3.115740741
Necroptosis	ko04217	2 out of 54	ENSRNOG00000001813;ENSRNOG00000046585	0.27598862	1.938683128
Protein processing in endoplas	ko04141	2 out of 54	ENSRNOG00000000611;ENSRNOG00000008277	0.290921729	1.866112101
Endocrine and other factor-reg	ko04961	1 out of 54	ENSRNOG00000033835	0.29245405	2.908024691
Basal cell carcinoma	ko05217	1 out of 54	ENSRNOG00000047211	0.32054115	2.604201216
Amphetamine addiction	ko05031	1 out of 54	ENSRNOG00000009745	0.328363027	2.528717123
Focal adhesion	ko04510	2 out of 54	ENSRNOG00000001068;ENSRNOG00000048172	0.329136282	1.702258356
Glutathione metabolism	ko00480	1 out of 54	ENSRNOG00000013409	0.343742574	2.390157281
Adipocytokine signaling pathw	ko04920	1 out of 54	ENSRNOG00000001309	0.343742574	2.390157281
Proteoglycans in cancer	ko05205	2 out of 54	ENSRNOG00000001068;ENSRNOG00000047211	0.348066575	1.630668051
Melanoma	ko05218	1 out of 54	ENSRNOG00000008960	0.355049886	2.295808967
Renin secretion	ko04924	1 out of 54	ENSRNOG00000009745	0.358776326	2.265993266
p53 signaling pathway	ko04115	1 out of 54	ENSRNOG00000003329	0.362481631	2.23694207
cAMP signaling pathway	ko04024	2 out of 54	ENSRNOG00000001068;ENSRNOG00000048172	0.362677108	1.579017932
Synaptic vesicle cycle	ko04721	1 out of 54	ENSRNOG00000033835	0.36616592	2.208626348
Renal cell carcinoma	ko05211	1 out of 54	ENSRNOG00000001068	0.377093851	2.127822945

Longevity regulating pathway	ko04211	1 out of 54	ENSRNOG00000001309	0.4018783	1.960466084
Toll-like receptor signaling pa	ko04620	1 out of 54	ENSRNOG00000001068	0.412201501	1.896537842
Pathways in cancer	ko05200	4 out of 54	ENSRNOG00000001068;ENSRNOG00000008960;ENSRNOG00000047211;ENSRNOG00000048172	0.412947172	1.22658335
Salmonella infection	ko05132	2 out of 54	ENSRNOG00000001068;ENSRNOG00000059705	0.443578087	1.337022847
Melanogenesis	ko04916	1 out of 54	ENSRNOG00000047211	0.451773874	1.677706553
AGE-RAGE signaling pathwa	ko04933	1 out of 54	ENSRNOG00000001068	0.451773874	1.677706553
PD-L1 expression and PD-1 cl	ko05235	1 out of 54	ENSRNOG00000009745	0.458109999	1.646051712
Th1 and Th2 cell differentiatio	ko04658	1 out of 54	ENSRNOG00000009745	0.464374236	1.615569273
Glucagon signaling pathway	ko04922	1 out of 54	ENSRNOG00000009745	0.467479646	1.600747537
Pancreatic secretion	ko04972	1 out of 54	ENSRNOG00000001068	0.467479646	1.600747537
Epstein-Barr virus infection	ko05169	2 out of 54	ENSRNOG00000001068;ONT.1410	0.472516547	1.264358561
TNF signaling pathway	ko04668	1 out of 54	ENSRNOG00000001813	0.479725543	1.544083907
Leukocyte transendothelial mi	ko04670	1 out of 54	ENSRNOG00000001068	0.479725543	1.544083907
Biosynthesis of amino acids	ko01230	1 out of 54	ENSRNOG00000004160	0.494644793	1.478656623
C-type lectin receptor signalin	ko04625	1 out of 54	ENSRNOG00000009745	0.503393933	1.441995715
Glutamatergic synapse	ko04724	1 out of 54	ENSRNOG00000009745	0.503393933	1.441995715
Oocyte meiosis	ko04114	1 out of 54	ENSRNOG00000009745	0.50914392	1.41854863
Neurotrophin signaling pathwa	ko04722	1 out of 54	ENSRNOG00000001068	0.51482855	1.395851852
T cell receptor signaling pathv	ko04660	1 out of 54	ENSRNOG00000009745	0.517646583	1.384773663
Th17 cell differentiation	ko04659	1 out of 54	ENSRNOG00000009745	0.526004643	1.352569624
Prion disease	ko05020	2 out of 54	ENSRNOG00000001068;ENSRNOG00000009745	0.531228803	1.132996633
Purine metabolism	ko00230	1 out of 54	ENSRNOG00000004160	0.552851182	1.255262457
Dopaminergic synapse	ko04728	1 out of 54	ENSRNOG00000009745	0.555452285	1.246296296
Signaling pathways regulating	ko04550	1 out of 54	ENSRNOG00000047211	0.568236672	1.203320562
Insulin signaling pathway	ko04910	1 out of 54	ENSRNOG00000029028	0.573248769	1.186948854
HIF-1 signaling pathway	ko04066	1 out of 54	ENSRNOG00000029028	0.585530518	1.147904483
Adrenergic signaling in cardio	ko04261	1 out of 54	ENSRNOG00000036827	0.597465163	1.111347016
Cushing syndrome	ko04934	1 out of 54	ENSRNOG00000047211	0.597465163	1.111347016
Spliceosome	ko03040	1 out of 54	ENSRNOG00000033790	0.599811292	1.104313174
Hippo signaling pathway	ko04390	1 out of 54	ENSRNOG00000047211	0.604463357	1.090509259
Alcoholism	ko05034	1 out of 54	ENSRNOG00000001309	0.609062334	1.077046182
Autophagy - animal	ko04140	1 out of 54	ENSRNOG00000001309	0.6113421	1.070438537
mTOR signaling pathway	ko04150	1 out of 54	ENSRNOG00000047211	0.615862556	1.057463524
MicroRNAs in cancer	ko05206	1 out of 54	ENSRNOG00000047211	0.631281467	1.014427218
Influenza A	ko05164	1 out of 54	ENSRNOG00000020683	0.635574171	1.002767135
cGMP-PKG signaling pathwa	ko04022	1 out of 54	ENSRNOG00000009745	0.639817812	0.991372054
Non-alcoholic fatty liver disea	ko04932	1 out of 54	ENSRNOG00000001068	0.644012942	0.980233042
Phospholipase D signaling pat	ko04072	1 out of 54	ENSRNOG00000033835	0.648160105	0.969341564
Cellular senescence	ko04218	1 out of 54	ENSRNOG00000009745	0.650215868	0.963986086
Hepatocellular carcinoma	ko05225	1 out of 54	ENSRNOG00000047211	0.656312677	0.948268921
PI3K-Akt signaling pathway	ko04151	2 out of 54	ENSRNOG00000001068;ENSRNOG00000008960	0.656786506	0.901713083
NOD-like receptor signaling p	ko04621	1 out of 54	ENSRNOG00000001813	0.658321674	0.943143143
Viral carcinogenesis	ko05203	1 out of 54	ENSRNOG00000001068	0.696165113	0.851129178
Phagosome	ko04145	1 out of 54	ENSRNOG00000001068	0.706693244	0.826926453
Alzheimer disease	ko05010	2 out of 54	ENSRNOG00000009745;ENSRNOG00000047211	0.709659508	0.817243473
Tuberculosis	ko05152	1 out of 54	ENSRNOG00000009745	0.711822195	0.815334026
Calcium signaling pathway	ko04020	1 out of 54	ENSRNOG00000009745	0.769645286	0.692386831
Human T-cell leukemia virus	ko05166	1 out of 54	ENSRNOG00000009745	0.786680797	0.658420685
Human papillomavirus infecti	ko05165	1 out of 54	ENSRNOG00000047211	0.872035524	0.497098238

#Kegg_pathway-blue	ko_id	Cluster_fre	GeneID	P-value	rich_factor
Leukocyte transendothelial migration	ko04670	19 out of 14	ENSRNOG00000001419;ENSRNOG00000001691;ENSRNOG00000001926;ENSRNOG00000005342;ENSRNOG00000007922;ENSRNOG00000010085;ENSRNOG00000010263;ENSRNOG00000011424;ENSRNOG00000016695;ENSRNOG00000018524;ENSRNOG00000020485;ENSRNOG00000023531;ENSRNOG00000027691;ENSRNOG00000030386;ENSRNOG00000030889;ENSRNOG00000031521;ENSRNOG00000054495;ENSRNOG00000055138;ENS	4.49E-13	11.3159292
Tight junction	ko04530	18 out of 14	ENSRNOG00000001419;ENSRNOG00000001691;ENSRNOG00000001926;ENSRNOG00000007922;ENSRNOG00000010085;ENSRNOG00000010263;ENSRNOG00000011424;ENSRNOG00000018524;ENSRNOG00000023531;ENSRNOG00000027691;ENSRNOG00000028216;ENSRNOG00000030386;ENSRNOG00000030889;ENSRNOG00000031521;ENSRNOG00000054080;ENSRNOG00000054495;ENSRNOG0000005	6.48E-11	7.043023256
Renin-angiotensin system	ko04614	10 out of 14	ENSRNOG00000007467;ENSRNOG00000029237;ENSRNOG00000031665;ENSRNOG00000032857;ENSRNOG00000033414;ENSRNOG00000046297;ENSRNOG00000048630;ENSRNOG00000049279;ENSRNOG00000062101;ONT.2260	1.26E-10	17.71052632
Cell adhesion molecules	ko04514	16 out of 14	ENSRNOG00000001419;ENSRNOG00000001691;ENSRNOG00000001926;ENSRNOG00000007922;ENSRNOG00000010085;ENSRNOG00000010263;ENSRNOG00000011424;ENSRNOG00000020129;ENSRNOG00000023531;ENSRNOG00000027691;ENSRNOG00000030386;ENSRNOG00000030889;ENSRNOG00000031521;ENSRNOG00000054495;ENSRNOG00000055138;ENSRNO	1.11E-08	5.884153005
Hepatitis C	ko05160	15 out of 14	ENSRNOG00000001419;ENSRNOG00000001691;ENSRNOG00000001926;ENSRNOG00000007922;ENSRNOG00000010085;ENSRNOG00000010263;ENSRNOG00000011424;ENSRNOG00000023531;ENSRNOG00000027691;ENSRNOG00000030386;ENSRNOG00000030889;ENSRNOG00000031521;ENSRNOG00000054495;ENSRNOG00000055138;	2.29E-08	6.04491018
Endocrine and other factor-regulated calciun	ko04961	7 out of 14(	ENSRNOG00000001092;ENSRNOG00000029237;ENSRNOG00000032857;ENSRNOG00000033414;ENSRNOG00000046297;ENSRNOG00000048630;ENSRNOG00000049279	2.75E-05	7.851666667
Protein digestion and absorption	ko04974	7 out of 14(	ENSRNOG00000006119;ENSRNOG00000010296;ENSRNOG00000012344;ENSRNOG00000014311;ENSRNOG00000016057;ENSRNOG00000031665;ENSRNOG00000039668	0.000881115	4.529807692
Renin secretion	ko04924	5 out of 14(	ENSRNOG00000007467;ENSRNOG00000011648;ENSRNOG00000038202;ENSRNOG00000062101;ONT	0.005658455	4.37012987

Malaria	ko05144	4 out of 14(	ENSRNOG00000007310;ENSRNOG00000007811;ENSRNOG00000057410;ENSRNOG00000061739	0.020108962	3.845714286
Gastric acid secretion	ko04971	4 out of 14(	ENSRNOG00000014347;ENSRNOG00000018524;ENSRNOG00000029811;ENSRNOG00000038202	0.025197017	3.589333333
Parkinson disease	ko05012	9 out of 14(	ENSRNOG00000004680;ENSRNOG00000005299;ENSRNOG00000011572;ENSRNOG00000014656;ENSRNOG00000017466;ENSRNOG00000018101;ENSRNOG00000018168;ENSRNOG00000020299;ENSRNO	0.028205298	2.095847751
Bile secretion	ko04976	4 out of 14(	ENSRNOG00000010378;ENSRNOG00000011648;ENSRNOG00000014347;ENSRNOG00000031249	0.032242775	3.32345679
Prion disease	ko05020	9 out of 14(	ENSRNOG00000004680;ENSRNOG00000005299;ENSRNOG00000011572;ENSRNOG00000014656;ENSRNOG00000017466;ENSRNOG00000018101;ENSRNOG00000018168;ENSRNOG00000020299;ENSRNO	0.039922398	1.966558442
Sphingolipid metabolism	ko00600	3 out of 14(	ENSRNOG00000007637;ENSRNOG00000036866;ENSRNOG0000004	0.045971513	3.738888889
Alzheimer disease	ko05010	11 out of 14	ENSRNOG00000001953;ENSRNOG00000004680;ENSRNOG00000005299;ENSRNOG00000011572;ENSRNOG00000014656;ENSRNOG00000016847;ENSRNOG00000017466;ENSRNOG00000018101;ENSRNOG00000018168;ENSRNOG00000020299;ENSRNOG00000038202	0.052879979	1.733723653
Hypertrophic cardiomyopathy	ko05410	4 out of 14(	ENSRNOG00000007467;ENSRNOG00000008346;ENSRNOG00000062101;ONT.2260	0.054449422	2.804166667
Huntington disease	ko05016	9 out of 14(	ENSRNOG00000004680;ENSRNOG00000005299;ENSRNOG00000011572;ENSRNOG00000014656;ENSRNOG00000017466;ENSRNOG00000018101;ENSRNOG00000018168;ENSRNOG00000020299;ENSRNO	0.069384926	1.765889213
Thyroid hormone synthesis	ko04918	3 out of 14(	ENSRNOG00000016275;ENSRNOG00000018822;ENSRNOG0000004	0.103455917	2.656578947
Alanine, aspartate and glutamate metabolism	ko00250	2 out of 14(	ENSRNOG00000008625;ENSRNOG00000023814	0.11874115	3.365
Linoleic acid metabolism	ko00591	2 out of 14(	ENSRNOG00000016838;ENSRNOG00000021405	0.11874115	3.365
Cell cycle	ko04110	4 out of 14(	ENSRNOG00000001283;ENSRNOG00000002105;ENSRNOG00000010760;ENSRNOG00000059500	0.122906316	2.103125
Endocytosis	ko04144	7 out of 14(	ENSRNOG00000003284;ENSRNOG00000004680;ENSRNOG00000005299;ENSRNOG00000007806;ENSRNOG00000014997;ENSRNOG00000017466;ENSRNOG00000019902	0.131813103	1.652982456
Natural killer cell mediated cytotoxicity	ko04650	4 out of 14(	ENSRNOG00000020485;ENSRNOG00000055196;ENSRNOG00000060246;ENSRNOG00000061739	0.135914938	2.02406015
Arachidonic acid metabolism	ko00590	3 out of 14(	ENSRNOG00000016838;ENSRNOG00000021405;ENSRNOG0000004	0.142655794	2.294318182
Amyotrophic lateral sclerosis	ko05014	9 out of 14(	ENSRNOG00000004680;ENSRNOG00000005299;ENSRNOG00000011572;ENSRNOG00000014656;ENSRNOG00000017466;ENSRNOG00000018101;ENSRNOG00000018168;ENSRNOG00000020299;ENSRNO	0.150013341	1.491871921
Dopaminergic synapse	ko04728	4 out of 14(	ENSRNOG00000004680;ENSRNOG00000005299;ENSRNOG00000017466;ENSRNOG00000038202	0.155018321	1.922857143

Complement and coagulation cascades	ko04610	3 out of 14(	ENSRNOG00000014118;ENSRNOG00000057855;ENSRNOG0000006	0.156687157	2.194565217
Basal transcription factors	ko03022	2 out of 14(	ENSRNOG00000018027;ENSRNOG00000020274	0.159380799	2.804166667
RNA degradation	ko03018	3 out of 14(	ENSRNOG00000001214;ENSRNOG00000013078;ENSRNOG0000006	0.167481755	2.125263158
Ether lipid metabolism	ko00565	2 out of 14(	ENSRNOG00000004089;ENSRNOG00000016838	0.169911323	2.692
Ras signaling pathway	ko04014	6 out of 14(	ENSRNOG00000005271;ENSRNOG00000005342;ENSRNOG00000016838;ENSRNOG00000020369;ENSRNOG00000022631;ENSRNOG000	0.176777048	1.58976378
Small cell lung cancer	ko05222	3 out of 14(	ENSRNOG00000011300;ENSRNOG00000043311;ENSRNOG0000004	0.189688725	1.999009901
Salmonella infection	ko05132	6 out of 14(	ENSRNOG00000011572;ENSRNOG00000018101;ENSRNOG00000018168;ENSRNOG00000020299;ENSRNOG00000042353;ENSRNOG000	0.192403839	1.547126437
Spliceosome	ko03040	4 out of 14(	ENSRNOG00000005513;ENSRNOG00000010587;ENSRNOG00000024170;ENSRNOG00000040045	0.208225688	1.703797468
Proteoglycans in cancer	ko05205	5 out of 14(	ENSRNOG00000007650;ENSRNOG00000016695;ENSRNOG00000018524;ENSRNOG00000020369;ENSRNOG00000020485	0.212977934	1.572429907
Mineral absorption	ko04978	2 out of 14(	ENSRNOG00000000017;ENSRNOG00000014475	0.245985107	2.103125
Rap1 signaling pathway	ko04015	5 out of 14(	ENSRNOG00000005271;ENSRNOG00000005342;ENSRNOG00000020485;ENSRNOG00000022631;ENSRNOG00000038202	0.248456365	1.482378855
Wnt signaling pathway	ko04310	4 out of 14(	ENSRNOG00000012364;ENSRNOG00000014180;ENSRNOG00000022417;ENSRNOG00000022772	0.249494	1.574269006
Ubiquitin mediated proteolysis	ko04120	4 out of 14(	ENSRNOG00000001283;ENSRNOG00000006335;ENSRNOG00000014029;ENSRNOG00000043311	0.262543331	1.538285714
Pentose and glucuronate interconversions	ko00040	1 out of 14(	ENSRNOG00000001092	0.270002226	3.204761905
Chagas disease	ko05142	3 out of 14(	ENSRNOG00000007467;ENSRNOG00000062101;ONT.2260	0.280145587	1.628225806
Proximal tubule bicarbonate reclamation	ko04964	1 out of 14(	ENSRNOG00000011648	0.280873376	3.059090909
Central carbon metabolism in cancer	ko05230	2 out of 14(	ENSRNOG00000001214;ENSRNOG00000018824	0.290175042	1.869444444
Graft-versus-host disease	ko05332	2 out of 14(	ENSRNOG00000055196;ENSRNOG00000060246	0.290175042	1.869444444
alpha-Linolenic acid metabolism	ko00592	1 out of 14(	ENSRNOG00000016838	0.302135775	2.804166667
Salivary secretion	ko04970	2 out of 14(	ENSRNOG00000014347;ENSRNOG00000038202	0.328612619	1.703797468
Maturity onset diabetes of the young	ko04950	1 out of 14(	ENSRNOG00000020420	0.332864509	2.492592593
Lysosome	ko04142	3 out of 14(	ENSRNOG00000007650;ENSRNOG00000014997;ENSRNOG0000001	0.337160043	1.463043478
Phototransduction	ko04744	1 out of 14(	ENSRNOG00000038202	0.342805857	2.403571429
Estrogen signaling pathway	ko04915	3 out of 14(	ENSRNOG00000016695;ENSRNOG00000038202;ENSRNOG0000004	0.357537286	1.411888112
Signaling pathways regulating pluripotency (	ko04550	3 out of 14(	ENSRNOG00000004768;ENSRNOG00000016299;ENSRNOG0000001	0.365668609	1.392413793
Galactose metabolism	ko00052	1 out of 14(	ENSRNOG00000001214	0.371755922	2.170967742
Antifolate resistance	ko01523	1 out of 14(	ENSRNOG00000019902	0.371755922	2.170967742
Insulin signaling pathway	ko04910	3 out of 14(	ENSRNOG00000020420;ENSRNOG00000038202;ENSRNOG0000004	0.373783874	1.373469388
Pentose phosphate pathway	ko00030	1 out of 14(	ENSRNOG00000001214	0.381121716	2.103125
ECM-receptor interaction	ko04512	2 out of 14(	ENSRNOG00000008346;ENSRNOG00000011300	0.387763454	1.495555556
SNARE interactions in vesicular transport	ko04130	1 out of 14(	ENSRNOG00000012748	0.399439416	1.979411765
Fluid shear stress and atherosclerosis	ko05418	3 out of 14(	ENSRNOG00000014205;ENSRNOG00000016695;ENSRNOG0000003	0.410023134	1.294230769
GnRH signaling pathway	ko04912	2 out of 14(	ENSRNOG00000016695;ENSRNOG00000038202	0.424226214	1.387628866

Apoptosis - multiple species	ko04215	1 out of 14(	ENSRNOG00000043311	0.425911372	1.818918919
Fructose and mannose metabolism	ko00051	1 out of 14(	ENSRNOG00000001214	0.434475294	1.771052632
TGF-beta signaling pathway	ko04350	2 out of 14(	ENSRNOG00000010760;ENSRNOG00000050123	0.44456004	1.332673267
Glycolysis / Gluconeogenesis	ko00010	2 out of 14(	ENSRNOG00000001214;ENSRNOG00000020420	0.449581997	1.319607843
Regulation of actin cytoskeleton	ko04810	4 out of 14(	ENSRNOG00000008346;ENSRNOG00000018524;ENSRNOG00000020485;ENSRNOG00000022631	0.450548639	1.165367965
Pyruvate metabolism	ko00620	1 out of 14(	ENSRNOG00000020420	0.45941338	1.641463415
MicroRNAs in cancer	ko05206	3 out of 14(	ENSRNOG00000000924;ENSRNOG00000006122;ENSRNOG0000001	0.472725535	1.173837209
Antigen processing and presentation	ko04612	2 out of 14(	ENSRNOG00000055196;ENSRNOG00000060246	0.479168938	1.246296296
Bladder cancer	ko05219	1 out of 14(	ENSRNOG00000016695	0.483259274	1.529545455
B cell receptor signaling pathway	ko04662	2 out of 14(	ENSRNOG00000004273;ENSRNOG00000020485	0.484006319	1.234862385
Glucagon signaling pathway	ko04922	2 out of 14(	ENSRNOG00000001214;ENSRNOG00000038202	0.484006319	1.234862385
Pancreatic secretion	ko04972	2 out of 14(	ENSRNOG00000014347;ENSRNOG00000016838	0.484006319	1.234862385
Parathyroid hormone synthesis, secretion an(	ko04928	2 out of 14(	ENSRNOG00000001092;ENSRNOG00000047028	0.493598168	1.212612613
TNF signaling pathway	ko04668	2 out of 14(	ENSRNOG00000042353;ENSRNOG00000047957	0.503077844	1.191150442
Cellular senescence	ko04218	3 out of 14(	ENSRNOG00000005342;ENSRNOG00000010760;ENSRNOG0000003	0.506675701	1.115469613
Inflammatory mediator regulation of TRP ch	ko04750	2 out of 14(	ENSRNOG00000021405;ENSRNOG00000038202	0.507775058	1.180701754
Type II diabetes mellitus	ko04930	1 out of 14(	ENSRNOG00000020420	0.520703433	1.373469388
Fat digestion and absorption	ko04975	1 out of 14(	ENSRNOG00000016838	0.520703433	1.373469388
Toxoplasmosis	ko05145	2 out of 14(	ENSRNOG00000011300;ENSRNOG00000043311	0.521693823	1.15042735
Biosynthesis of amino acids	ko01230	2 out of 14(	ENSRNOG00000001214;ENSRNOG00000020420	0.526275193	1.140677966
C-type lectin receptor signaling pathway	ko04625	2 out of 14(	ENSRNOG00000038202;ENSRNOG00000054251	0.539842483	1.112396694
Oocyte meiosis	ko04114	2 out of 14(	ENSRNOG00000001283;ENSRNOG00000038202	0.548738685	1.094308943
Ferroptosis	ko04216	1 out of 14(	ENSRNOG00000010210	0.568640778	1.201785714
AMPK signaling pathway	ko04152	2 out of 14(	ENSRNOG00000001214;ENSRNOG00000011603	0.574704865	1.043410853
Pyrimidine metabolism	ko00240	1 out of 14(	ENSRNOG00000050277	0.587698546	1.140677966
Focal adhesion	ko04510	3 out of 14(	ENSRNOG00000008346;ENSRNOG00000011300;ENSRNOG0000002	0.591299893	0.984878049
Sphingolipid signaling pathway	ko04071	2 out of 14(	ENSRNOG00000007637;ENSRNOG00000047368	0.595507879	1.004477612
Glycerolipid metabolism	ko00561	1 out of 14(	ENSRNOG00000029096	0.611814435	1.068253968
Pathways in cancer	ko05200	8 out of 14(	ENSRNOG00000005342;ENSRNOG00000011300;ENSRNOG00000016695;ENSRNOG00000020369;ENSRNOG00000022631;ENSRNOG00000038202;ENSRNOG00000043311;ENSRNOG00000047957	0.615856213	0.946221441
Vascular smooth muscle contraction	ko04270	2 out of 14(	ENSRNOG00000016838;ENSRNOG00000038202	0.642323805	0.921917808
Steroid hormone biosynthesis	ko00140	1 out of 14(	ENSRNOG00000021405	0.645386706	0.975362319
Amphetamine addiction	ko05031	1 out of 14(	ENSRNOG00000038202	0.645386706	0.975362319
Long-term potentiation	ko04720	1 out of 14(	ENSRNOG00000038202	0.65069472	0.961428571
Glutathione metabolism	ko00480	1 out of 14(	ENSRNOG00000048812	0.666150042	0.921917808
Retinol metabolism	ko00830	1 out of 14(	ENSRNOG00000021405	0.666150042	0.921917808
Non-small cell lung cancer	ko05223	1 out of 14(	ENSRNOG00000005342	0.666150042	0.921917808
Apelin signaling pathway	ko04371	2 out of 14(	ENSRNOG00000014205;ENSRNOG00000038202	0.667621931	0.879738562
Pertussis	ko05133	1 out of 14(	ENSRNOG00000038202	0.676074419	0.897333333
Melanoma	ko05218	1 out of 14(	ENSRNOG00000022631	0.680926199	0.885526316
Glioma	ko05214	1 out of 14(	ENSRNOG00000038202	0.690414346	0.862820513

Carbon metabolism	ko01200	2 out of 14(	ENSRNOG00000001214;ENSRNOG00000020420	0.69475914	0.836024845
Prolactin signaling pathway	ko04917	1 out of 14(	ENSRNOG00000008282	0.69505283	0.851898734
Alcoholism	ko05034	2 out of 14(	ENSRNOG00000038202;ENSRNOG00000055597	0.698020623	0.830864198
mTOR signaling pathway	ko04150	2 out of 14(	ENSRNOG00000011603;ENSRNOG00000018824	0.707633676	0.815757576
Chemical carcinogenesis	ko05204	1 out of 14(	ENSRNOG00000021405	0.708558291	0.820731707
Arrhythmogenic right ventricular cardiomyo	ko05412	1 out of 14(	ENSRNOG00000008346	0.708558291	0.820731707
Drug metabolism - other enzymes	ko00983	1 out of 14(	ENSRNOG00000050277	0.712926796	0.810843373
Longevity regulating pathway	ko04211	1 out of 14(	ENSRNOG00000001092	0.737805549	0.756179775
Platinum drug resistance	ko01524	1 out of 14(	ENSRNOG00000014475	0.741738606	0.747777778
PPAR signaling pathway	ko03320	1 out of 14(	ENSRNOG00000029096	0.74561308	0.73956044
Non-alcoholic fatty liver disease	ko04932	2 out of 14(	ENSRNOG00000014656;ENSRNOG00000020420	0.746398229	0.756179775
Toll-like receptor signaling pathway	ko04620	1 out of 14(	ENSRNOG00000007437	0.749429838	0.731521739
Necroptosis	ko04217	2 out of 14(	ENSRNOG00000042353;ENSRNOG00000047957	0.751957808	0.747777778
Thermogenesis	ko04714	3 out of 14(	ENSRNOG00000004784;ENSRNOG00000014656;ENSRNOG0000004	0.752084768	0.770610687
Fc epsilon RI signaling pathway	ko04664	1 out of 14(	ENSRNOG00000020485	0.760542288	0.708421053
Progesterone-mediated oocyte maturation	ko04914	1 out of 14(	ENSRNOG00000001283	0.760542288	0.708421053
Circadian entrainment	ko04713	1 out of 14(	ENSRNOG00000038202	0.764136593	0.701041667
Aldosterone synthesis and secretion	ko04925	1 out of 14(	ENSRNOG00000038202	0.764136593	0.701041667
Protein processing in endoplasmic reticulum	ko04141	2 out of 14(	ENSRNOG00000000438;ENSRNOG00000005958	0.770602983	0.719786096
Cardiac muscle contraction	ko04260	1 out of 14(	ENSRNOG00000014656	0.774601237	0.67979798
Endocrine resistance	ko01522	1 out of 14(	ENSRNOG00000016695	0.777985966	0.673
Glycerophospholipid metabolism	ko00564	1 out of 14(	ENSRNOG00000016838	0.781320225	0.666336634
IL-17 signaling pathway	ko04657	1 out of 14(	ENSRNOG00000047957	0.781320225	0.666336634
Melanogenesis	ko04916	1 out of 14(	ENSRNOG00000038202	0.791027603	0.647115385
AGE-RAGE signaling pathway in diabetic c	ko04933	1 out of 14(	ENSRNOG00000016695	0.791027603	0.647115385
RNA transport	ko03013	2 out of 14(	ENSRNOG00000007701;ENSRNOG00000023356	0.792786276	0.686734694
Phosphatidylinositol signaling system	ko04070	1 out of 14(	ENSRNOG00000038202	0.797260249	0.63490566
Viral carcinogenesis	ko05203	2 out of 14(	ENSRNOG00000047957;ENSRNOG00000055597	0.81306904	0.656585366
Tuberculosis	ko05152	2 out of 14(	ENSRNOG00000038202;ENSRNOG00000054251	0.831574042	0.628971963
PI3K-Akt signaling pathway	ko04151	4 out of 14(	ENSRNOG00000008346;ENSRNOG00000011300;ENSRNOG00000020369;ENSRNOG00000022631	0.83291694	0.695607235
cAMP signaling pathway	ko04024	2 out of 14(	ENSRNOG00000020485;ENSRNOG00000038202	0.844816065	0.609049774
Fc gamma R-mediated phagocytosis	ko04666	1 out of 14(	ENSRNOG00000020485	0.84566636	0.542741935
Neurotrophin signaling pathway	ko04722	1 out of 14(	ENSRNOG00000038202	0.847990162	0.5384
T cell receptor signaling pathway	ko04660	1 out of 14(	ENSRNOG00000020485	0.850279221	0.534126984
Dilated cardiomyopathy	ko05414	1 out of 14(	ENSRNOG00000008346	0.850279221	0.534126984
Serotonergic synapse	ko04726	1 out of 14(	ENSRNOG00000021405	0.852534052	0.52992126
Platelet activation	ko04611	1 out of 14(	ENSRNOG00000012748	0.854755163	0.52578125
Thyroid hormone signaling pathway	ko04919	1 out of 14(	ENSRNOG00000001214	0.859098224	0.517692308
Amoebiasis	ko05146	1 out of 14(	ENSRNOG00000011300	0.859098224	0.517692308
Relaxin signaling pathway	ko04926	1 out of 14(	ENSRNOG00000016695	0.867401254	0.502238806
FoxO signaling pathway	ko04068	1 out of 14(	ENSRNOG00000014205	0.871368717	0.494852941
Purine metabolism	ko00230	1 out of 14(	ENSRNOG00000020420	0.877099965	0.484172662
Staphylococcus aureus infection	ko05150	1 out of 14(	ENSRNOG00000047393	0.880779201	0.477304965
NF-kappa B signaling pathway	ko04064	1 out of 14(	ENSRNOG00000047957	0.886093978	0.467361111
Human immunodeficiency virus 1 infection	ko05170	2 out of 14(	ENSRNOG00000038202;ENSRNOG00000047957	0.890218147	0.5384
Human cytomegalovirus infection	ko05163	2 out of 14(	ENSRNOG00000038202;ENSRNOG00000047957	0.891540572	0.53625498
Apoptosis	ko04210	1 out of 14(	ENSRNOG00000016496	0.894434152	0.451677852
Systemic lupus erythematosus	ko05322	1 out of 14(	ENSRNOG00000061379	0.894434152	0.451677852
HIF-1 signaling pathway	ko04066	1 out of 14(	ENSRNOG00000001214	0.8991442	0.442763158
Breast cancer	ko05224	1 out of 14(	ENSRNOG00000022631	0.905101195	0.431410256
Adrenergic signaling in cardiomyocytes	ko04261	1 out of 14(	ENSRNOG00000038202	0.906535022	0.42866242
Oxytocin signaling pathway	ko04921	1 out of 14(	ENSRNOG00000038202	0.906535022	0.42866242
Gastric cancer	ko05226	1 out of 14(	ENSRNOG00000022631	0.906535022	0.42866242

Human T-cell leukemia virus 1 infection	ko05166	2 out of 14(	ENSRNOG00000001283;ENSRNOG00000006876	0.908574862	0.507924528
Oxidative phosphorylation	ko00190	1 out of 14(	ENSRNOG00000014656	0.910708709	0.420625
Hippo signaling pathway	ko04390	1 out of 14(	ENSRNOG00000050123	0.910708709	0.420625
Autophagy - animal	ko04140	1 out of 14(	ENSRNOG00000012748	0.914697293	0.412883436
Yersinia infection	ko05135	1 out of 14(	ENSRNOG00000020485	0.924489439	0.393567251
cGMP-PKG signaling pathway	ko04022	1 out of 14(	ENSRNOG00000038202	0.930033958	0.382386364
Hepatocellular carcinoma	ko05225	1 out of 14(	ENSRNOG00000020369	0.938076267	0.36576087
Chemokine signaling pathway	ko04062	1 out of 14(	ENSRNOG00000020485	0.939014708	0.363783784
NOD-like receptor signaling pathway	ko04621	1 out of 14(	ENSRNOG00000047957	0.939014708	0.363783784
MAPK signaling pathway	ko04010	2 out of 14(	ENSRNOG00000020369;ENSRNOG00000022631	0.947923442	0.434193548
Phagosome	ko04145	1 out of 14(	ENSRNOG00000054251	0.959023201	0.318957346
Kaposi sarcoma-associated herpesvirus infec	ko05167	1 out of 14(	ENSRNOG00000038202	0.965379749	0.303153153
Human papillomavirus infection	ko05165	2 out of 14(	ENSRNOG00000008346;ENSRNOG00000011300	0.969297299	0.383475783
Transcriptional misregulation in cancer	ko05202	1 out of 14(	ENSRNOG00000002163	0.975679423	0.274693878
Calcium signaling pathway	ko04020	1 out of 14(	ENSRNOG00000038202	0.978161474	0.267063492
Cytokine-cytokine receptor interaction	ko04060	1 out of 14(	ENSRNOG00000050123	0.983950855	0.247426471
Epstein-Barr virus infection	ko05169	1 out of 14(	ENSRNOG00000047957	0.984910937	0.24384058
Herpes simplex virus 1 infection	ko05168	2 out of 14(	ENSRNOG00000005513;ENSRNOG00000047957	0.984977262	0.332345679
Olfactory transduction	ko04740	1 out of 14(	ENSRNOG00000038202	1	0.048592058
#Kegg_pathway	ko_id	Cluster_fre(	GeneID	P-value	rich_factor

#Kegg_pathway-red	ko_id	Cluster_fre	GeneID	P-value	rich_factor
Oxidative phosphorylation	ko00190	47 out of 27	ENSRNOG00000000064;ENSRNOG00000001170;ENSRNOG00000001551;ENSRNOG00000002721;ENSRNOG00000002840;ENSRNOG00000003626;ENSRNOG00000004526;ENSRNOG00000005698;ENSRNOG00000006939;ENSRNOG00000007235;ENSRNOG00000007392;ENSRNOG00000008569;ENSRNOG00000010807;ENSRNOG00000011825;ENSRNOG00000012383;ENSRNOG00000012550;ENSRNOG00000014078;ENSRNOG00000014568;ENSRNOG00000016660;ENSRNOG00000016952;ENSRNOG00000017571;ENSRNOG00000017817;ENSRNOG00000020602;ENSRNOG00000021177;ENSRNOG00000023387;ENSRNOG00000024309;ENSRNOG00000026616;ENSRNOG00000026646;ENSRNOG00000027049;ENSRNOG00000028717;ENSRNOG00000028884;ENSRNOG00000029339;ENSRNOG00000030237;ENSRNOG00000032602;ENSRNOG00000034161;ENSRNOG00000034182;ENSRNOG00000038951;ENSRNOG00000042903;ENSRNOG00000048174;ENSRNOG00000048320;ENSRNOG00000049394;ENSRNOG00000049912;	0	9.95580036
Ribosome	ko03010	112 out of 2	ENSRNOG00000000490;ENSRNOG00000000926;ENSRNOG00000000957;ENSRNOG00000001611;ENSRNOG00000002116;ENSRNOG00000002178;ENSRNOG00000003158;ENSRNOG00000004107;ENSRNOG00000004196;ENSRNOG00000004214;ENSRNOG00000004426;ENSRNOG00000005975;ENSRNOG00000006898;ENSRNOG00000008546;ENSRNOG00000008555;ENSRNOG00000010746;ENSRNOG00000011494;ENSRNOG00000012650;ENSRNOG00000013508;ENSRNOG00000013845;ENSRNOG00000014272;ENSRNOG00000014641;ENSRNOG00000015989;ENSRNOG00000016387;ENSRNOG00000016580;ENSRNOG00000016896;ENSRNOG00000016961;ENSRNOG00000017127;ENSRNOG00000017552;ENSRNOG00000018471;ENSRNOG00000018774;ENSRNOG00000018795;ENSRNOG00000019106;ENSRNOG00000019578;ENSRNOG00000019734;ENSRNOG00000019970;ENSRNOG00000019974;ENSRNOG00000020354;ENSRNOG00000020982;ENSRNOG00000022234;ENSRNOG00000022609;ENSRNOG00000023385;	0	7.622316605

Parkinson disease	ko05012	59 out of 27	ENSRNOG00000001170;ENSRNOG00000001551;ENSRNOG00000002721;ENSRNOG00000002840;ENSRNOG00000003597;ENSRNOG00000003626;ENSRNOG00000004426;ENSRNOG00000004526;ENSRNOG00000005698;ENSRNOG00000006069;ENSRNOG00000006939;ENSRNOG00000007235;ENSRNOG00000008569;ENSRNOG00000010807;ENSRNOG00000011825;ENSRNOG00000012383;ENSRNOG00000012550;ENSRNOG00000013505;ENSRNOG00000014078;ENSRNOG00000014568;ENSRNOG00000016660;ENSRNOG00000016952;ENSRNOG00000017209;ENSRNOG00000017445;ENSRNOG00000017558;ENSRNOG00000017571;ENSRNOG00000017817;ENSRNOG00000018371;ENSRNOG00000019974;ENSRNOG00000020602;ENSRNOG00000021177;ENSRNOG00000021438;ENSRNOG00000023387;ENSRNOG00000024309;ENSRNOG00000026616;ENSRNOG00000026646;ENSRNOG00000028717;ENSRNOG00000028750;ENSRNOG00000029339;ENSRNOG00000030237;ENSRNOG00000032602;ENSRNOG00000032967;	0	6.919145652
Amyotrophic lateral sclerosis	ko05014	60 out of 27	ENSRNOG00000001170;ENSRNOG00000001551;ENSRNOG00000002721;ENSRNOG00000002840;ENSRNOG00000003597;ENSRNOG00000003626;ENSRNOG00000004526;ENSRNOG00000004992;ENSRNOG00000005698;ENSRNOG00000006069;ENSRNOG00000006939;ENSRNOG00000007235;ENSRNOG00000007504;ENSRNOG00000007756;ENSRNOG00000008569;ENSRNOG00000010807;ENSRNOG00000011825;ENSRNOG00000012383;ENSRNOG00000012550;ENSRNOG00000014078;ENSRNOG00000014568;ENSRNOG00000016660;ENSRNOG00000016952;ENSRNOG00000017209;ENSRNOG00000017445;ENSRNOG00000017558;ENSRNOG00000017571;ENSRNOG00000017817;ENSRNOG00000018371;ENSRNOG00000020602;ENSRNOG00000021177;ENSRNOG00000021438;ENSRNOG00000021954;ENSRNOG00000023387;ENSRNOG00000024309;ENSRNOG00000026616;ENSRNOG00000026646;ENSRNOG00000028356;ENSRNOG00000028717;ENSRNOG00000028750;ENSRNOG00000029339;ENSRNOG00000030237;	0	5.008682709

Alzheimer disease	ko05010	62 out of 2[illegible]	ENSRNOG00000001170;ENSRNOG00000001551;ENSRNOG00000002721;ENSRNOG00000002840;ENSRNOG00000003597;ENSRNOG00000003626;ENSRNOG00000004526;ENSRNOG00000005355;ENSRNOG00000005698;ENSRNOG00000006069;ENSRNOG00000006939;ENSRNOG00000007235;ENSRNOG00000007756;ENSRNOG00000008569;ENSRNOG00000010807;ENSRNOG00000011825;ENSRNOG00000012383;ENSRNOG00000012550;ENSRNOG00000013505;ENSRNOG00000014078;ENSRNOG00000014568;ENSRNOG00000016660;ENSRNOG00000016952;ENSRNOG00000017209;ENSRNOG00000017445;ENSRNOG00000017558;ENSRNOG00000017571;ENSRNOG00000017817;ENSRNOG00000018371;ENSRNOG00000018630;ENSRNOG00000020602;ENSRNOG00000021177;ENSRNOG00000021438;ENSRNOG00000023387;ENSRNOG00000024309;ENSRNOG00000026616;ENSRNOG00000026646;ENSRNOG00000028717;ENSRNOG00000028750;ENSRNOG00000029339;ENSRNOG00000030237;ENSRNOG00000030963;[illegible]	0	4.921099186
Non-alcoholic fatty liver disease	ko04932	38 out of 2[illegible]	ENSRNOG00000001170;ENSRNOG00000002721;ENSRNOG00000004526;ENSRNOG00000005698;ENSRNOG00000006069;ENSRNOG00000006939;ENSRNOG00000008569;ENSRNOG00000010807;ENSRNOG00000011825;ENSRNOG00000012383;ENSRNOG00000012550;ENSRNOG00000014078;ENSRNOG00000014568;ENSRNOG00000016660;ENSRNOG00000016952;ENSRNOG00000017571;ENSRNOG00000017817;ENSRNOG00000020602;ENSRNOG00000021177;ENSRNOG00000023387;ENSRNOG00000024309;ENSRNOG00000026616;ENSRNOG00000026646;ENSRNOG00000028717;ENSRNOG00000029339;ENSRNOG00000030237;ENSRNOG00000032602;ENSRNOG00000034161;ENSRNOG00000034182;ENSRNOG00000042903;ENSRNOG00000048174;ENSRNOG00000048320;ENSRNOG00000049394;ENSRNOG00000050514;ENSRNOG00000059061;	2.80E-13	7.235389217

Thermogenesis	ko04714	47 out of 27	ENSRNOG00000000064;ENSRNOG00000001170;ENSRNOG00000001551;ENSRNOG00000002721;ENSRNOG00000002840;ENSRNOG00000003626;ENSRNOG00000004526;ENSRNOG00000005698;ENSRNOG00000006939;ENSRNOG00000007235;ENSRNOG00000008223;ENSRNOG00000008569;ENSRNOG00000010807;ENSRNOG00000011825;ENSRNOG00000012383;ENSRNOG00000012550;ENSRNOG00000014078;ENSRNOG00000014568;ENSRNOG00000016660;ENSRNOG00000016952;ENSRNOG00000017571;ENSRNOG00000017817;ENSRNOG00000020602;ENSRNOG00000021177;ENSRNOG00000023387;ENSRNOG00000024309;ENSRNOG00000026616;ENSRNOG00000026646;ENSRNOG00000027049;ENSRNOG00000028717;ENSRNOG00000028884;ENSRNOG00000029339;ENSRNOG00000030237;ENSRNOG00000032602;ENSRNOG00000034161;ENSRNOG00000034182;ENSRNOG00000038951;ENSRNOG00000042903;ENSRNOG00000048174;ENSRNOG00000048320;ENSRNOG00000049394;ENSRNOG00000049912;	2.98E-13	6.079878082
Prion disease	ko05020	55 out of 27	ENSRNOG00000001170;ENSRNOG00000001551;ENSRNOG00000002721;ENSRNOG00000002840;ENSRNOG00000003597;ENSRNOG00000003626;ENSRNOG00000004526;ENSRNOG00000005698;ENSRNOG00000006069;ENSRNOG00000006939;ENSRNOG00000007235;ENSRNOG00000008569;ENSRNOG00000010807;ENSRNOG00000011825;ENSRNOG00000012383;ENSRNOG00000012550;ENSRNOG00000013505;ENSRNOG00000014078;ENSRNOG00000014568;ENSRNOG00000016660;ENSRNOG00000016952;ENSRNOG00000017209;ENSRNOG00000017445;ENSRNOG00000017558;ENSRNOG00000017571;ENSRNOG00000017817;ENSRNOG00000018371;ENSRNOG00000020602;ENSRNOG00000021177;ENSRNOG00000021438;ENSRNOG00000023387;ENSRNOG00000024309;ENSRNOG00000026616;ENSRNOG00000026646;ENSRNOG00000028717;ENSRNOG00000028750;ENSRNOG00000029339;ENSRNOG00000030237;ENSRNOG00000032602;ENSRNOG00000032967;ENSRNOG00000034161;ENSRNOG00000034182;	1.34E-12	6.052158273

Huntington disease	ko05016	59 out of 27	ENSRNOG00000001170;ENSRNOG00000001551;ENSRNOG00000002721;ENSRNOG00000002840;ENSRNOG00000003597;ENSRNOG00000003626;ENSRNOG00000004526;ENSRNOG00000005698;ENSRNOG00000006939;ENSRNOG00000007235;ENSRNOG00000007504;ENSRNOG00000007756;ENSRNOG00000008569;ENSRNOG00000010807;ENSRNOG00000011825;ENSRNOG00000012383;ENSRNOG00000012550;ENSRNOG00000013505;ENSRNOG00000014078;ENSRNOG00000014568;ENSRNOG00000016163;ENSRNOG00000016660;ENSRNOG00000016952;ENSRNOG00000017209;ENSRNOG00000017445;ENSRNOG00000017558;ENSRNOG00000017571;ENSRNOG00000017817;ENSRNOG00000018371;ENSRNOG00000019293;ENSRNOG00000020602;ENSRNOG00000021177;ENSRNOG00000021438;ENSRNOG00000023387;ENSRNOG00000024309;ENSRNOG00000026616;ENSRNOG00000026646;ENSRNOG00000028717;ENSRNOG00000028750;ENSRNOG00000029339;ENSRNOG00000030237;ENSRNOG00000032602;	1.48E-12	5.829834092
Retrograde endocannabinoid signaling	ko04723	22 out of 27	ENSRNOG00000002721;ENSRNOG00000005698;ENSRNOG00000006939;ENSRNOG00000008223;ENSRNOG00000008569;ENSRNOG00000011825;ENSRNOG00000012383;ENSRNOG00000014078;ENSRNOG00000014568;ENSRNOG00000017571;ENSRNOG00000020602;ENSRNOG00000023387;ENSRNOG00000026616;ENSRNOG00000026646;ENSRNOG00000028717;ENSRNOG00000029339;ENSRNOG00000034182;ENSRNOG00000048320;ENSRNOG00000049394;ENSRNOG000	5.33E-09	4.360385376
Cardiac muscle contraction	ko04260	16 out of 27	ENSRNOG00000001170;ENSRNOG00000004526;ENSRNOG00000010807;ENSRNOG00000012550;ENSRNOG00000016660;ENSRNOG00000016952;ENSRNOG00000017817;ENSRNOG00000021177;ENSRNOG00000024309;ENSRNOG00000030237;ENSRNOG00000032602;ENSRNOG00000034161;ENSRNOG00000042903;ENSRNOG00000048174;ENSRNOG00000059061;ONT.4361	2.68E-08	5.477508902
Gap junction	ko04540	13 out of 27	ENSRNOG00000003597;ENSRNOG00000010170;ENSRNOG00000017209;ENSRNOG00000017445;ENSRNOG00000017558;ENSRNOG00000018371;ENSRNOG00000021438;ENSRNOG00000028750;ENSRNOG00000032967;ENSRNOG00000046151;ENSRNOG00000047505;ENSRNOG00000048169;ENSRNOG000	1.07E-06	5.183495556

Phagosome	ko04145	18 out of 27	ENSRNOG00000003597;ENSRNOG00000006130;ENSRNOG00000007392;ENSRNOG00000010170;ENSRNOG00000017209;ENSRNOG00000017445;ENSRNOG00000017558;ENSRNOG00000018371;ENSRNOG00000018566;ENSRNOG00000021438;ENSRNOG00000028750;ENSRNOG00000032967;ENSRNOG00000039954;ENSRNOG00000046151;ENSRNOG00000047505;ENSRNOG00000048169;ENSRNOG0000005	5.02E-05	2.891268028
Mitophagy - animal	ko04137	8 out of 278	ENSRNOG00000004426;ENSRNOG00000006069;ENSRNOG00000019425;ENSRNOG00000019974;ENSRNOG00000034246;ENSRNOG00000043114;ENSRNOG00000057823;	0.002392807	3.389208633
Apoptosis	ko04210	10 out of 27	ENSRNOG00000003597;ENSRNOG00000006069;ENSRNOG00000018566;ENSRNOG00000021438;ENSRNOG00000028750;ENSRNOG00000032967;ENSRNOG00000039954;ENSRNOG00000048169;ENSRNOG00000053468;ONT.13366	0.0127074	2.274636667
Ubiquitin mediated proteolysis	ko04120	10 out of 27	ENSRNOG00000004426;ENSRNOG00000004814;ENSRNOG00000005828;ENSRNOG00000016930;ENSRNOG00000019798;ENSRNOG00000019974;ENSRNOG00000033199;ENSRNOG00000034246;ENSRNOG00000057823;ONT.872	0.034388201	1.936690647
Autophagy - animal	ko04140	9 out of 278	ENSRNOG00000004992;ENSRNOG00000006027;ENSRNOG00000006069;ENSRNOG00000007756;ENSRNOG00000018566;ENSRNOG00000019425;ENSRNOG00000039954;ENSRNOG00000059880;ONT.1286	0.052027844	1.87134219
Autophagy - other	ko04136	3 out of 278	ENSRNOG00000007756;ENSRNOG00000019425;ONT.12865	0.08327773	2.905035971
Valine, leucine and isoleucine biosynthesis	ko00290	1 out of 278	ONT.3510	0.139102257	6.778417266
Glycolysis / Gluconeogenesis	ko00010	5 out of 278	ENSRNOG00000018630;ENSRNOG00000021009;ENSRNOG00000030963;ENSRNOG00000033057;ENSRNOG00000050630	0.182868513	1.661376781
Protein processing in endoplasmic reticulum	ko04141	8 out of 278	ENSRNOG00000001050;ENSRNOG00000005828;ENSRNOG00000006027;ENSRNOG00000006069;ENSRNOG00000008604;ENSRNOG00000019798;ENSRNOG00000036689;ENSRNOG00000048315	0.188002203	1.449928827
Synaptic vesicle cycle	ko04721	4 out of 278	ENSRNOG00000003905;ENSRNOG00000007392;ENSRNOG00000014816;ENSRNOG00000016163	0.204269852	1.716055004
Protein export	ko03060	2 out of 278	ENSRNOG00000017036;ENSRNOG00000018075	0.210415582	2.337385264
Cysteine and methionine metabolism	ko00270	3 out of 278	ENSRNOG00000010625;ENSRNOG00000026649;ONT.3510	0.212974624	1.882893685
Lysosome	ko04142	6 out of 278	ENSRNOG00000001465;ENSRNOG00000012953;ENSRNOG00000018566;ENSRNOG00000019249;ENSRNOG00000039954;ENSRNOG000	0.22280272	1.473568971
Sulfur relay system	ko04122	1 out of 278	ENSRNOG00000026636	0.236361919	3.76578737

Tight junction	ko04530	7 out of 278	ENSRNOG00000003597;ENSRNOG00000021438;ENSRNOG00000028750;ENSRNOG00000032967;ENSRNOG00000048169;ENSRNOG00000053468;ONT.872	0.245444602	1.379329095
RNA polymerase	ko03020	2 out of 278	ENSRNOG00000019293;ENSRNOG00000048562	0.254347924	2.054065838
Biosynthesis of amino acids	ko01230	5 out of 278	ENSRNOG00000018630;ENSRNOG00000030963;ENSRNOG00000033057;ENSRNOG00000050630;ONT	0.268941905	1.436105353
Propanoate metabolism	ko00640	2 out of 278	ENSRNOG00000005686;ONT.3883	0.287448674	1.882893685
HIF-1 signaling pathway	ko04066	6 out of 278	ENSRNOG00000004814;ENSRNOG00000018630;ENSRNOG00000030963;ENSRNOG00000033057;ENSRNOG00000033199;ENSRNOG000	0.292367869	1.337845513
Carbon metabolism	ko01200	6 out of 278	ENSRNOG00000005686;ENSRNOG00000018630;ENSRNOG00000030963;ENSRNOG00000033057;ENSRNOG00000050630;ONT.3510	0.339229048	1.263059118
Salmonella infection	ko05132	9 out of 278	ENSRNOG00000005828;ENSRNOG00000006130;ENSRNOG00000014317;ENSRNOG00000018630;ENSRNOG00000030963;ENSRNOG00000033057;ENSRNOG00000050630;ENSRNOG00000059373;ONT.1382	0.364482867	1.168692632
Vasopressin-regulated water reabsorption	ko04962	2 out of 278	ENSRNOG00000003905;ENSRNOG00000006130	0.374351879	1.540549379
Other types of O-glycan biosynthesis	ko00514	2 out of 278	ENSRNOG00000003359;ENSRNOG00000024533	0.374351879	1.540549379
Proteasome	ko03050	2 out of 278	ENSRNOG00000048470;ENSRNOG00000049229	0.466556351	1.278946654
Pantothenate and CoA biosynthesis	ko00770	1 out of 278	ENSRNOG00000025286	0.467204255	1.613908873
Glycosaminoglycan degradation	ko00531	1 out of 278	ENSRNOG00000001465	0.467204255	1.613908873
Cytosolic DNA-sensing pathway	ko04623	2 out of 278	ENSRNOG00000019293;ENSRNOG00000048562	0.49548745	1.210431655
PPAR signaling pathway	ko03320	3 out of 278	ENSRNOG00000007771;ENSRNOG00000046889;ENSRNOG0000005	0.506009933	1.117321527
Mannose type O-glycan biosynthesis	ko00515	1 out of 278	ENSRNOG00000013742	0.513091462	1.412170264
Pyrimidine metabolism	ko00240	2 out of 278	ENSRNOG00000002671;ENSRNOG00000002693	0.523405412	1.148884282
Hepatitis C	ko05160	5 out of 278	ENSRNOG00000001050;ENSRNOG00000006027;ENSRNOG00000006069;ENSRNOG00000048315;ENSRNOG00000051650	0.550604062	1.014733124
Collecting duct acid secretion	ko04966	1 out of 278	ENSRNOG00000007392	0.555039606	1.255262457
Phenylalanine metabolism	ko00360	1 out of 278	ENSRNOG00000006589	0.555039606	1.255262457
Legionellosis	ko05134	2 out of 278	ENSRNOG00000004992;ENSRNOG00000009439	0.55898636	1.075939249
Citrate cycle (TCA cycle)	ko00020	1 out of 278	ENSRNOG00000005686	0.617101991	1.059127698
Kaposi sarcoma-associated herpesvirus infecti	ko05167	6 out of 278	ENSRNOG00000004426;ENSRNOG00000019974;ENSRNOG00000034246;ENSRNOG00000048315;ENSRNOG00000057823;ONT.12865	0.64419801	0.916002333
Bacterial invasion of epithelial cells	ko05100	2 out of 278	ENSRNOG00000014317;ONT.1382	0.668285126	0.880313931
Renin-angiotensin system	ko04614	1 out of 278	ENSRNOG00000003858	0.680294103	0.891897009
Spliceosome	ko03040	4 out of 278	ENSRNOG00000016507;ENSRNOG00000031127;ENSRNOG00000032232;ONT.3852	0.690470995	0.858027502
Glutamatergic synapse	ko04724	3 out of 278	ENSRNOG00000014816;ENSRNOG00000016163;ENSRNOG0000005	0.697955702	0.840299661
Renal cell carcinoma	ko05211	2 out of 278	ENSRNOG00000004814;ENSRNOG00000033199	0.701657963	0.826636252
Drug metabolism - other enzymes	ko00983	2 out of 278	ENSRNOG00000002671;ENSRNOG00000002693	0.707988986	0.816676779
Tyrosine metabolism	ko00350	1 out of 278	ENSRNOG00000006589	0.716534144	0.806954436

Neurotrophin signaling pathway	ko04722	3 out of 27ε	ENSRNOG00000018839;ENSRNOG00000019907;ENSRNOG0000002	0.718760492	0.813410072
mTOR signaling pathway	ko04150	4 out of 27ε	ENSRNOG00000007392;ENSRNOG00000019908;ENSRNOG00000027028;ONT.8028	0.722569251	0.821626335
Cell cycle	ko04110	3 out of 27ε	ENSRNOG00000005828;ENSRNOG00000021248;ENSRNOG0000005	0.733625013	0.794345773
Homologous recombination	ko03440	1 out of 27ε	ONT.3569	0.74100076	0.753157474
Various types of N-glycan biosynthesis	ko00513	1 out of 27ε	ONT.3501	0.74100076	0.753157474
Glycine, serine and threonine metabolism	ko00260	1 out of 27ε	ONT.3510	0.748679313	0.736784485
Measles	ko05162	4 out of 27ε	ENSRNOG00000001050;ENSRNOG00000006027;ENSRNOG00000006069;ENSRNOG00000048315	0.752146312	0.788188054
GABAergic synapse	ko04727	2 out of 27ε	ENSRNOG00000003905;ONT.1286	0.754693488	0.744881018
Basal transcription factors	ko03022	1 out of 27ε	ENSRNOG00000058393	0.763362542	0.706085132
Aminoacyl-tRNA biosynthesis	ko00970	1 out of 27ε	ENSRNOG00000003149	0.77719082	0.677841727
Rheumatoid arthritis	ko05323	3 out of 27ε	ENSRNOG00000007392;ENSRNOG00000018566;ENSRNOG0000003	0.77873394	0.736784485
Cholesterol metabolism	ko04979	1 out of 27ε	ENSRNOG00000013505	0.802478099	0.627631228
Sphingolipid metabolism	ko00600	1 out of 27ε	ENSRNOG00000012953	0.802478099	0.627631228
Fanconi anemia pathway	ko03460	1 out of 27ε	ONT.3569	0.808339658	0.616219751
Valine, leucine and isoleucine degradation	ko00280	1 out of 27ε	ONT.3883	0.808339658	0.616219751
Ferroptosis	ko04216	1 out of 27ε	ENSRNOG00000013505	0.81402788	0.605215827
N-Glycan biosynthesis	ko00510	1 out of 27ε	ONT.3501	0.81402788	0.605215827
Arginine and proline metabolism	ko00330	1 out of 27ε	ENSRNOG00000010872	0.81402788	0.605215827
Regulation of lipolysis in adipocytes	ko04923	1 out of 27ε	ENSRNOG00000003442	0.824904593	0.584346316
Notch signaling pathway	ko04330	1 out of 27ε	ENSRNOG00000005355	0.824904593	0.584346316
Phosphatidylinositol signaling system	ko04070	2 out of 27ε	ENSRNOG00000009948;ENSRNOG00000011420	0.82508318	0.639473327
Antigen processing and presentation	ko04612	2 out of 27ε	ENSRNOG00000018566;ENSRNOG00000039954	0.832961866	0.627631228
Spinocerebellar ataxia	ko05017	3 out of 27ε	ENSRNOG00000007756;ENSRNOG00000009288;ENSRNOG0000001	0.834474243	0.664550712
Apelin signaling pathway	ko04371	3 out of 27ε	ENSRNOG00000019425;ONT.12865;ONT.8028	0.834474243	0.664550712
Oocyte meiosis	ko04114	2 out of 27ε	ENSRNOG00000005828;ENSRNOG00000051650	0.882540718	0.551090835
Fc gamma R-mediated phagocytosis	ko04666	2 out of 27ε	ENSRNOG00000014317;ONT.1382	0.885309165	0.546646554
MicroRNAs in cancer	ko05206	3 out of 27ε	ENSRNOG00000010625;ENSRNOG00000021248;ENSRNOG0000002	0.887430811	0.591141041
Acute myeloid leukemia	ko05221	1 out of 27ε	ENSRNOG00000018414	0.888623002	0.464275155
Adipocytokine signaling pathway	ko04920	1 out of 27ε	ENSRNOG00000019907	0.888623002	0.464275155
Gastric acid secretion	ko04971	1 out of 27ε	ENSRNOG00000017679	0.895148632	0.451894484
Osteoclast differentiation	ko04380	2 out of 27ε	ENSRNOG00000004763;ENSRNOG00000018414	0.895790838	0.529563849
Renin secretion	ko04924	1 out of 27ε	ENSRNOG00000003442	0.901293218	0.440156965
Cellular senescence	ko04218	3 out of 27ε	ENSRNOG00000013505;ENSRNOG00000020835;ONT.1073	0.906797296	0.561747287
Inositol phosphate metabolism	ko00562	1 out of 27ε	ENSRNOG00000011420	0.907078933	0.429013751
FoxO signaling pathway	ko04068	2 out of 27ε	ENSRNOG00000019425;ONT.1286	0.914138221	0.498413034
NOD-like receptor signaling pathway	ko04621	3 out of 27ε	ENSRNOG00000013505;ENSRNOG00000019425;ONT.12865	0.914398069	0.5496014
Pancreatic cancer	ko05212	1 out of 27ε	ONT.3569	0.917656093	0.40833839
Purine metabolism	ko00230	2 out of 27ε	ENSRNOG00000002671;ENSRNOG00000002693	0.920204135	0.487655918
Viral protein interaction with cytokine and cy	ko04061	1 out of 27ε	ENSRNOG00000018414	0.922485749	0.398730427
Endocytosis	ko04144	5 out of 27ε	ENSRNOG00000006130;ENSRNOG00000014317;ENSRNOG00000052887;ONT.13829;ONT.872	0.92758942	0.594598006
Longevity regulating pathway	ko04211	1 out of 27ε	ENSRNOG00000007756	0.931314575	0.380809959
Transcriptional misregulation in cancer	ko05202	4 out of 27ε	ENSRNOG00000006532;ENSRNOG00000018414;ENSRNOG00000048736;ENSRNOG00000058393	0.934728029	0.553340185
Morphine addiction	ko05032	1 out of 27ε	ENSRNOG00000003442	0.940954576	0.36055411
Progesterone-mediated oocyte maturation	ko04914	1 out of 27ε	ENSRNOG00000021248	0.942714292	0.356758803

Viral carcinogenesis	ko05203	3 out of 278	ENSRNOG00000017828;ENSRNOG00000048315;ENSRNOG0000005	0.944603289	0.495981751
Fluid shear stress and atherosclerosis	ko05418	2 out of 278	ENSRNOG00000018566;ENSRNOG00000039954	0.947633253	0.434513927
TGF-beta signaling pathway	ko04350	1 out of 278	ENSRNOG00000005828	0.952227663	0.335565211
Leishmaniasis	ko05140	1 out of 278	ENSRNOG00000009439	0.953652479	0.332275356
Proteoglycans in cancer	ko05205	3 out of 278	ENSRNOG00000011076;ENSRNOG00000018566;ENSRNOG0000003	0.954679719	0.475122706
Alcoholism	ko05034	2 out of 278	ENSRNOG00000006532;ENSRNOG00000018839	0.954968105	0.418420819
Protein digestion and absorption	ko04974	1 out of 278	ENSRNOG00000014816	0.956376324	0.325885445
PD-L1 expression and PD-1 checkpoint pathw	ko05235	1 out of 278	ENSRNOG00000019907	0.958940631	0.319736663
Hepatitis B	ko05161	2 out of 278	ENSRNOG00000017828;ENSRNOG00000051650	0.959302193	0.40833839
Th1 and Th2 cell differentiation	ko04658	1 out of 278	ENSRNOG00000019907	0.961354711	0.313815614
Glucagon signaling pathway	ko04922	1 out of 278	ENSRNOG00000003712	0.962508178	0.310936572
B cell receptor signaling pathway	ko04662	1 out of 278	ENSRNOG00000019907	0.962508178	0.310936572
Insulin resistance	ko04931	1 out of 278	ENSRNOG00000003359	0.965766768	0.302607914
cGMP-PKG signaling pathway	ko04022	2 out of 278	ENSRNOG00000003442;ENSRNOG00000013505	0.9684613	0.385137345
Necroptosis	ko04217	2 out of 278	ENSRNOG00000013505;ENSRNOG00000048315	0.971540427	0.376578737
Hematopoietic cell lineage	ko04640	1 out of 278	ENSRNOG00000018414	0.973141506	0.282434053
C-type lectin receptor signaling pathway	ko04625	1 out of 278	ENSRNOG00000017828	0.9739442	0.280099887
T cell receptor signaling pathway	ko04660	1 out of 278	ENSRNOG00000019907	0.977612965	0.268984812
Platelet activation	ko04611	1 out of 278	ENSRNOG00000021161	0.978931997	0.264781924
Viral myocarditis	ko05416	1 out of 278	ENSRNOG00000017158	0.979562178	0.262729351
Th17 cell differentiation	ko04659	1 out of 278	ENSRNOG00000019907	0.979562178	0.262729351
Amoebiasis	ko05146	1 out of 278	ENSRNOG00000006130	0.980173575	0.260708356
RNA transport	ko03013	2 out of 278	ENSRNOG00000009439;ENSRNOG00000017158	0.981204363	0.345837616
Ras signaling pathway	ko04014	3 out of 278	ENSRNOG00000006130;ENSRNOG00000018414;ENSRNOG0000001	0.982009992	0.400300232
Sphingolipid signaling pathway	ko04071	1 out of 278	ENSRNOG00000003442	0.982442238	0.252926017
Vascular smooth muscle contraction	ko04270	1 out of 278	ENSRNOG00000020676	0.987809967	0.232137578
Insulin signaling pathway	ko04910	1 out of 278	ONT.8028	0.9881753	0.23055841
Systemic lupus erythematosus	ko05322	1 out of 278	ENSRNOG00000006532	0.988873559	0.227463667
Epstein-Barr virus infection	ko05169	3 out of 278	ENSRNOG00000019907;ENSRNOG00000048315;ONT.872	0.989381005	0.368392243
cAMP signaling pathway	ko04024	2 out of 278	ENSRNOG00000003442;ENSRNOG00000014039	0.990280563	0.306715713
Breast cancer	ko05224	1 out of 278	ONT.3569	0.991009326	0.217256964
Rap1 signaling pathway	ko04015	2 out of 278	ENSRNOG00000008223;ENSRNOG00000018414	0.991718569	0.29860869
Hippo signaling pathway	ko04390	1 out of 278	ENSRNOG00000051650	0.992040852	0.21182554
Regulation of actin cytoskeleton	ko04810	2 out of 278	ENSRNOG00000014317;ONT.1382	0.992559712	0.293437977
Human papillomavirus infection	ko05165	4 out of 278	ENSRNOG00000006494;ENSRNOG00000007392;ENSRNOG00000020213;ENSRNOG00000048315	0.993418992	0.386234602
Wnt signaling pathway	ko04310	1 out of 278	ENSRNOG00000005828	0.994308833	0.198199335
Influenza A	ko05164	1 out of 278	ENSRNOG00000048315	0.994806693	0.194782105
MAPK signaling pathway	ko04010	3 out of 278	ENSRNOG00000018414;ENSRNOG00000018839;ENSRNOG0000002	0.995401265	0.327987932
Human immunodeficiency virus 1 infection	ko05170	2 out of 278	ENSRNOG00000004814;ENSRNOG00000033199	0.99554337	0.271136691
Calcium signaling pathway	ko04020	2 out of 278	ENSRNOG00000013505;ENSRNOG00000017679	0.995778885	0.268984812
Cell adhesion molecules	ko04514	1 out of 278	ENSRNOG00000030515	0.996054581	0.185202658
Herpes simplex virus 1 infection	ko05168	4 out of 278	ENSRNOG00000001050;ENSRNOG00000006027;ENSRNOG00000006069;ENSRNOG00000048315	0.998165418	0.334736655
Tuberculosis	ko05152	1 out of 278	ENSRNOG00000006130	0.998472116	0.158374235
Neuroactive ligand-receptor interaction	ko04080	3 out of 278	ENSRNOG00000003442;ENSRNOG00000008223;ENSRNOG0000001	0.998642538	0.284011897
PI3K-Akt signaling pathway	ko04151	3 out of 278	ENSRNOG00000018414;ENSRNOG00000018839;ENSRNOG0000005	0.999362555	0.262729351

Human T-cell leukemia virus 1 infection	ko05166	1 out of 27٤	ENSRNOG00000013505	0.99968143	0.127894665
Cytokine-cytokine receptor interaction	ko04060	1 out of 27٤	ENSRNOG00000018414	0.999743286	0.124603259
Pathways in cancer	ko05200	4 out of 27٤	ENSRNOG00000004814;ENSRNOG00000018414;ENSRNOG00000033199;ONT.3569	0.999971922	0.238257197
Olfactory transduction	ko04740	1 out of 27٤	ENSRNOG00000042978	1	0.02447082
