# Supplementary material for: Effects of Maternal High-Fructose Diet on Long Non-Coding RNAs and Anxiety-like Behaviors in Offspring
Source: Int J Mol Sci. 2023 Feb 24;24(5):4460. doi: 10.3390/ijms24054460 (PMC10003385; doi:10.3390/ijms24054460)
Supplement: Supplementary file 1 [file ijms-24-04460-s001.zip › Table S6.pdf]

**Table S6:** The full KEGG pathway enrichment results of trans co-expression clusters in F13%.

| Term | Count | GeneID | Pvalue | group |
|---|---|---|---|---|
| positive regulation of transcription from R | 4 | ENSRNOG00000002671;ENSRNOG00000004814;ENSRNOG00000006355;ENSRNOG00000025923 | 1.70E-14 | BP |
| response to drug | 3 | ENSRNOG00000006589;ENSRNOG00000018795;ENSRNOG0000002 | 1.10E-07 | BP |
| cellular metabolic process | 149 | ENSRNOG00000000622;ENSRNOG00000001170;ENSRNOG00000001212;ENSRNOG00000001806;ENSRNOG00000002671;ENSRNOG00000003626;ENSRNOG00000004107;ENSRNOG00000004214;ENSRNOG00000004426;ENSRNOG00000004526;ENSRNOG00000004608;ENSRNOG00000004814;ENSRNOG00000005355;ENSRNOG00000005686;ENSRNOG00000006030;ENSRNOG00000006130;ENSRNOG00000006355;ENSRNOG00000006532;ENSRNOG00000006559;ENSRNOG00000006589;ENSRNOG00000006727;ENSRNOG00000006898;ENSRNOG00000006939;ENSRNOG00000008223;ENSRNOG00000008546;ENSRNOG00000008555;ENSRNOG00000008604;ENSRNOG00000009078;ENSRNOG00000009117;ENSRNOG00000009297;ENSRNOG00000009823;ENSRNOG00000010170;ENSRNOG00000010428;ENSRNOG00000010502;ENSRNOG00000010746;ENSRNOG00000010855;ENSRNOG00000011015;ENSRNOG00000011955;ENSRNOG00000012383;ENSRNOG00000012550;ENSRNOG00000013039;ENSRNOG00000013508; | 5.90E-07 | BP |
| negative regulation of cell proliferation | 2 | ENSRNOG00000006030;ENSRNOG00000050827 | 1.10E-06 | BP |
| Notch signaling pathway | 6 | ENSRNOG00000005355;ENSRNOG00000018471;ENSRNOG00000037897;ENSRNOG00000042939;ENSRNOG00000048199;ONT.153 | 1.70E-06 | BP |

| | | | | |
|---|---|---|---|---|
| nucleus | 81 | ENSRNOG00000000490;ENSRNOG00000000622;ENSRNOG00000001212;ENSRNOG00000002671;ENSRNOG00000002721;ENSRNOG00000003626;ENSRNOG00000004107;ENSRNOG00000004426;ENSRNOG00000004814;ENSRNOG00000005686;ENSRNOG00000006355;ENSRNOG00000006494;ENSRNOG00000006532;ENSRNOG00000006559;ENSRNOG00000006589;ENSRNOG00000008461;ENSRNOG00000008604;ENSRNOG00000009117;ENSRNOG00000009297;ENSRNOG00000009823;ENSRNOG00000010170;ENSRNOG00000010428;ENSRNOG00000011015;ENSRNOG00000013039;ENSRNOG00000013508;ENSRNOG00000015844;ENSRNOG00000016046;ENSRNOG00000016387;ENSRNOG00000016631;ENSRNOG00000017209;ENSRNOG00000017445;ENSRNOG00000018137;ENSRNOG00000018371;ENSRNOG00000018471;ENSRNOG00000018774;ENSRNOG00000019249;ENSRNOG00000019293;ENSRNOG00000019682;ENSRNOG00000019798;ENSRNOG00000019895;ENSRNOG00000020213;ENSRNOG00000020354; | 8.20E-21 | CC |
| nucleoplasm | 28 | ENSRNOG00000000622;ENSRNOG00000002721;ENSRNOG00000003626;ENSRNOG00000004814;ENSRNOG00000006355;ENSRNOG00000006559;ENSRNOG00000006589;ENSRNOG00000008604;ENSRNOG00000010170;ENSRNOG00000011015;ENSRNOG00000013039;ENSRNOG00000013508;ENSRNOG00000017445;ENSRNOG00000018137;ENSRNOG00000019249;ENSRNOG00000019293;ENSRNOG00000019798;ENSRNOG00000025923;ENSRNOG00000026649;ENSRNOG00000027906;ENSRNOG00000029512;ENSRNOG00000031127;ENSRNOG00000034182;ENSRNOG00000048470;ENSRNOG00000048562;ENSRNOG00000049229;ENSRNOG000 | 1.00E-18 | CC |
| neuronal cell body | 10 | ENSRNOG00000004426;ENSRNOG00000006030;ENSRNOG00000006130;ENSRNOG00000017209;ENSRNOG00000018371;ENSRNOG00000018839;ENSRNOG00000019895;ENSRNOG00000034246;ENSRNOG00000047505;ENSRNOG0000005 | 1.40E-15 | CC |

| | | | | |
|---|---|---|---|---|
| extracellular vesicular exosome | 77 | ENSRNOG00000000490;ENSRNOG00000000622;ENSRNOG00000001806;ENSRNOG00000002376;ENSRNOG00000002671;ENSRNOG00000002721;ENSRNOG00000003597;ENSRNOG00000003626;ENSRNOG00000004107;ENSRNOG00000004132;ENSRNOG00000004214;ENSRNOG00000004426;ENSRNOG00000004814;ENSRNOG00000005355;ENSRNOG00000006028;ENSRNOG00000006130;ENSRNOG00000006532;ENSRNOG00000006589;ENSRNOG00000010170;ENSRNOG00000011825;ENSRNOG00000011955;ENSRNOG00000013508;ENSRNOG00000014816;ENSRNOG00000015844;ENSRNOG00000016387;ENSRNOG00000016660;ENSRNOG00000017127;ENSRNOG00000017209;ENSRNOG00000017445;ENSRNOG00000018091;ENSRNOG00000018320;ENSRNOG00000018371;ENSRNOG00000018471;ENSRNOG00000018774;ENSRNOG00000019106;ENSRNOG00000019578;ENSRNOG00000019798;ENSRNOG00000019895;ENSRNOG00000019908;ENSRNOG00000020296;ENSRNOG00000021161;ENSRNOG00000021175; | 5.80E-14 | CC |
| perinuclear region of cytoplasm | 5 | ENSRNOG00000002671;ENSRNOG00000006130;ENSRNOG00000011076;ENSRNOG00000013039;ENSRNOG00000051650 | 1.40E-13 | CC |
| ATP binding | 10 | ENSRNOG00000002671;ENSRNOG00000005686;ENSRNOG00000010170;ENSRNOG00000017445;ENSRNOG00000018839;ENSRNOG00000025286;ENSRNOG00000043201;ENSRNOG00000051624;ENSRNOG00000061216;ONT.2092 | 8.10E-18 | MF |
| zinc ion binding | 9 | ENSRNOG00000004132;ENSRNOG00000006559;ENSRNOG00000016631;ENSRNOG00000019293;ENSRNOG00000019682;ENSRNOG00000025923;ENSRNOG00000026649;ENSRNOG00000040205;ENSRNO | 4.50E-13 | MF |
| protein homodimerization activity | 6 | ENSRNOG00000013039;ENSRNOG00000018471;ENSRNOG00000018839;ENSRNOG00000019798;ENSRNOG00000037897;ENSRNOG000 | 6.90E-13 | MF |

| | | | | |
|---|---|---|---|---|
| metal ion binding | 33 | ENSRNOG00000001212;ENSRNOG00000002671;ENSRNOG00000004132;ENSRNOG00000004426;ENSRNOG00000005686;ENSRNOG00000006355;ENSRNOG00000006559;ENSRNOG00000009888;ENSRNOG00000010091;ENSRNOG00000011015;ENSRNOG00000012367;ENSRNOG00000014829;ENSRNOG00000016631;ENSRNOG00000016660;ENSRNOG00000018958;ENSRNOG00000019293;ENSRNOG00000019682;ENSRNOG00000020058;ENSRNOG00000023385;ENSRNOG00000025923;ENSRNOG00000026116;ENSRNOG00000026649;ENSRNOG00000033803;ENSRNOG00000034180;ENSRNOG00000034246;ENSRNOG00000040205;ENSRNOG00000045963;ENSRNOG00000048562;ENSRNOG00000048958;ENSRNOG00000050473;ENSRNOG0000005 | 2.00E-11 | MF |
| identical protein binding | 7 | ENSRNOG00000013039;ENSRNOG00000018471;ENSRNOG00000018839;ENSRNOG00000019798;ENSRNOG00000026649;ENSRNOG00000037897;ENSRNOG00000048199 | 3.40E-11 | MF |

| Term | Count | GeneID | Pvalue | group |
|---|---|---|---|---|
| positive regulation of transcription from R | 6 | ENSRNOG00000001892;ENSRNOG00000002979;ENSRNOG00000006587;ENSRNOG00000008277;ENSRNOG00000014629;ENSRNOG000 | 1.10E-15 | BP |
| response to drug | 2 | ENSRNOG00000008277;ENSRNOG00000047211 | 3.10E-08 | BP |
| protein ubiquitination | 11 | ENSRNOG00000000611;ENSRNOG00000001470;ENSRNOG00000005758;ENSRNOG00000012333;ENSRNOG00000012524;ENSRNOG00000015385;ENSRNOG00000019529;ENSRNOG00000042274;ENSRNOG00000043106;ENSRNOG00000046497;ENSRNOG00000049219 | 6.90E-07 | BP |
| neuron migration | 2 | ENSRNOG00000042274;ENSRNOG00000047211 | 8.90E-07 | BP |
| protein autophosphorylation | 3 | ENSRNOG00000001309;ENSRNOG00000015385;ENSRNOG0000002 | 1.40E-06 | BP |

| | | | | |
|---|---|---|---|---|
| nucleus | 55 | ENSRNOG00000001068;ENSRNOG00000001139;ENSRNOG00000001309;ENSRNOG00000001470;ENSRNOG00000001632;ENSRNOG00000001892;ENSRNOG00000002979;ENSRNOG00000003833;ENSRNOG00000004206;ENSRNOG00000004426;ENSRNOG00000004474;ENSRNOG00000005258;ENSRNOG00000005486;ENSRNOG00000006587;ENSRNOG00000006929;ENSRNOG00000008277;ENSRNOG00000009103;ENSRNOG00000009266;ENSRNOG00000010189;ENSRNOG00000010363;ENSRNOG00000010685;ENSRNOG00000010873;ENSRNOG00000012524;ENSRNOG00000014463;ENSRNOG00000014629;ENSRNOG00000014765;ENSRNOG00000015385;ENSRNOG00000015753;ENSRNOG00000016313;ENSRNOG00000016580;ENSRNOG00000019027;ENSRNOG00000019178;ENSRNOG00000019310;ENSRNOG00000019565;ENSRNOG00000019721;ENSRNOG00000021224;ENSRNOG00000022934;ENSRNOG00000026905;ENSRNOG00000027722;ENSRNOG00000029028;ENSRNOG00000034246;ENSRNOG00000036675; | 2.50E-21 | CC |
| nucleoplasm | 19 | ENSRNOG00000001139;ENSRNOG00000001470;ENSRNOG00000003833;ENSRNOG00000005258;ENSRNOG00000010189;ENSRNOG00000010685;ENSRNOG00000014463;ENSRNOG00000019027;ENSRNOG00000019178;ENSRNOG00000019310;ENSRNOG00000019565;ENSRNOG00000021224;ENSRNOG00000022934;ENSRNOG00000026905;ENSRNOG00000029028;ENSRNOG00000049219;ENSRNOG00000052445;ENSRNOG00000058461;ONT | 1.20E-19 | CC |
| neuronal cell body | 6 | ENSRNOG00000004206;ENSRNOG00000004426;ENSRNOG00000034246;ENSRNOG00000042274;ENSRNOG00000047211;ENSRNOG000 | 1.10E-16 | CC |
| perinuclear region of cytoplasm | 8 | ENSRNOG00000001470;ENSRNOG00000001813;ENSRNOG00000008277;ENSRNOG00000014765;ENSRNOG00000015385;ENSRNOG00000019178;ENSRNOG00000047088;ENSRNOG00000048172 | 1.20E-14 | CC |
| Golgi membrane | 7 | ENSRNOG00000001068;ENSRNOG00000001813;ENSRNOG00000005331;ENSRNOG00000014765;ENSRNOG00000015051;ENSRNOG00000047088;ENSRNOG00000048172 | 1.10E-13 | CC |
| ATP binding | 9 | ENSRNOG00000000611;ENSRNOG00000001309;ENSRNOG00000001470;ENSRNOG00000004160;ENSRNOG00000006962;ENSRNOG00000015385;ENSRNOG00000029028;ONT.14113;ONT.1417 | 1.50E-18 | MF |

| | | | | |
|---|---|---|---|---|
| protein homodimerization activity | 9 | ENSRNOG00000001139;ENSRNOG00000001813;ENSRNOG00000001892;ENSRNOG00000004160;ENSRNOG00000005331;ENSRNOG00000010685;ENSRNOG00000019565;ENSRNOG00000046585;ENSRNO | 1.30E-13 | MF |
| zinc ion binding | 5 | ENSRNOG00000001470;ENSRNOG00000014629;ENSRNOG00000046497;ENSRNOG00000049219;ENSRNOG00000052445 | 1.40E-13 | MF |
| identical protein binding | 13 | ENSRNOG00000001139;ENSRNOG00000001813;ENSRNOG00000001892;ENSRNOG00000004160;ENSRNOG00000005331;ENSRNOG00000010685;ENSRNOG00000019565;ENSRNOG00000033835;ENSRNOG00000046227;ENSRNOG00000046497;ENSRNOG00000046585;ENSRNOG00000052025;ENSRNOG000 | 1.50E-12 | MF |
| metal ion binding | 21 | ENSRNOG00000001309;ENSRNOG00000001470;ENSRNOG00000003833;ENSRNOG00000004160;ENSRNOG00000004206;ENSRNOG00000004426;ENSRNOG00000005486;ENSRNOG00000006962;ENSRNOG00000012524;ENSRNOG00000014629;ENSRNOG00000015385;ENSRNOG00000019721;ENSRNOG00000026905;ENSRNOG00000029028;ENSRNOG00000034246;ENSRNOG00000043225;ENSRNOG00000046497;ENSRNOG00000046527;ENSRNOG00000049219;ENSRNOG000 | 1.80E-11 | MF |

| #Kegg_pathway-blue | ko_id | Cluster_fre | GeneID | P-value | rich_factor |
|---|---|---|---|---|---|
| Pentose phosphate pathway | ko00030 | 1 out of 48 | ENSRNOG00000004160 | 0.151011424 | 6.134114583 |
| Purine metabolism | ko00230 | 1 out of 48 | ENSRNOG00000004160 | 0.510907675 | 1.412170264 |
| Cysteine and methionine metabolism | ko00270 | 1 out of 48 | ENSRNOG00000013409 | 0.241630562 | 3.635030864 |
| Selenocompound metabolism | ko00450 | 1 out of 48 | ONT.1417 | 0.092566678 | 10.33114035 |
| Glutathione metabolism | ko00480 | 1 out of 48 | ENSRNOG00000013409 | 0.312216875 | 2.688926941 |
| Amino sugar and nucleotide sugar meta | ko00520 | 1 out of 48 | ENSRNOG00000013816 | 0.225890647 | 3.925833333 |
| Glyoxylate and dicarboxylate metaboli | ko00630 | 1 out of 48 | ENSRNOG00000009536 | 0.155351302 | 5.948232323 |
| Aminoacyl-tRNA biosynthesis | ko00970 | 1 out of 48 | ENSRNOG00000024460 | 0.225890647 | 3.925833333 |
| Carbon metabolism | ko01200 | 2 out of 48 | ENSRNOG00000004160;ENSRNOG00000009536 | 0.197732602 | 2.438405797 |
| Biosynthesis of amino acids | ko01230 | 1 out of 48 | ENSRNOG00000004160 | 0.45472545 | 1.663488701 |
| Ribosome | ko03010 | 17 out of 48 | ENSRNOG00000004426;ENSRNOG00000008555;ENSRNOG00000010164;ENSRNOG00000010189;ENSRNOG00000014179;ENSRNOG00000016580;ENSRNOG00000016961;ENSRNOG00000022934;ENSRNOG00000024845;ENSRNOG00000027503;ENSRNOG00000028939;ENSRNOG00000034246;ENSRNOG00000042886;ENSRNOG00000049921;ENSRNOG00000050473;ENSRNO | 1.38E-10 | 6.700719545 |
| Basal transcription factors | ko03022 | 1 out of 48 | ENSRNOG00000019178 | 0.217901183 | 4.089409722 |
| Homologous recombination | ko03440 | 1 out of 48 | ONT.14100 | 0.205765381 | 4.362037037 |
| MAPK signaling pathway | ko04010 | 4 out of 48 | ENSRNOG00000001068;ENSRNOG00000009745;ENSRNOG00000029028;ENSRNOG00000048172 | 0.072251713 | 2.532795699 |
| Ras signaling pathway | ko04014 | 3 out of 48 | ENSRNOG00000001068;ENSRNOG00000046497;ENSRNOG0000004 | 0.138826721 | 2.318405512 |
| Rap1 signaling pathway | ko04015 | 2 out of 48 | ENSRNOG00000001068;ENSRNOG00000048172 | 0.322382387 | 1.729441997 |
| Calcium signaling pathway | ko04020 | 1 out of 48 | ENSRNOG00000009745 | 0.728715115 | 0.778935185 |
| cGMP-PKG signaling pathway | ko04022 | 1 out of 48 | ENSRNOG00000009745 | 0.596424496 | 1.115293561 |
| cAMP signaling pathway | ko04024 | 2 out of 48 | ENSRNOG00000001068;ENSRNOG00000048172 | 0.311062921 | 1.776395173 |
| Chemokine signaling pathway | ko04062 | 3 out of 48 | ENSRNOG00000001068;ENSRNOG00000048172;ENSRNOG0000005 | 0.067731596 | 3.183108108 |
| HIF-1 signaling pathway | ko04066 | 1 out of 48 | ENSRNOG00000029028 | 0.542803386 | 1.291392544 |
| Sphingolipid signaling pathway | ko04071 | 2 out of 48 | ENSRNOG00000001068;ENSRNOG00000048172 | 0.14870983 | 2.929726368 |
| Phospholipase D signaling pathway | ko04072 | 1 out of 48 | ENSRNOG00000033835 | 0.604741342 | 1.090509259 |
| Oocyte meiosis | ko04114 | 1 out of 48 | ENSRNOG00000009745 | 0.468649581 | 1.595867209 |
| Ubiquitin mediated proteolysis | ko04120 | 5 out of 48 | ENSRNOG00000000611;ENSRNOG00000004426;ENSRNOG00000032690;ENSRNOG00000034246;ONT | 0.001873319 | 5.608333333 |
| Mitophagy - animal | ko04137 | 3 out of 48 | ENSRNOG00000004426;ENSRNOG00000015385;ENSRNOG0000003 | 0.007747598 | 7.3609375 |
| Autophagy - animal | ko04140 | 1 out of 48 | ENSRNOG00000001309 | 0.568193869 | 1.204243354 |
| Protein processing in endoplasmic retic | ko04141 | 2 out of 48 | ENSRNOG00000000611;ENSRNOG00000008277 | 0.246581135 | 2.099376114 |
| Endocytosis | ko04144 | 6 out of 48 | ENSRNOG00000001086;ENSRNOG00000015753;ENSRNOG00000033835;ENSRNOG00000046585;ENSRNOG00000047088;ONT.14104 | 0.003064277 | 4.13245614 |
| Phagosome | ko04145 | 1 out of 48 | ENSRNOG00000001068 | 0.663750496 | 0.930292259 |
| mTOR signaling pathway | ko04150 | 1 out of 48 | ENSRNOG00000047211 | 0.572659596 | 1.189646465 |
| PI3K-Akt signaling pathway | ko04151 | 1 out of 48 | ENSRNOG00000001068 | 0.867123557 | 0.507213609 |
| AMPK signaling pathway | ko04152 | 2 out of 48 | ENSRNOG00000001309;ENSRNOG00000047088 | 0.139939231 | 3.043281654 |
| Longevity regulating pathway | ko04211 | 1 out of 48 | ENSRNOG00000001309 | 0.366634404 | 2.205524345 |
| Ferroptosis | ko04216 | 1 out of 48 | ENSRNOG00000013409 | 0.249382571 | 3.505208333 |
| Necroptosis | ko04217 | 2 out of 48 | ENSRNOG00000001813;ENSRNOG00000046585 | 0.233340058 | 2.181018519 |
| Cellular senescence | ko04218 | 1 out of 48 | ENSRNOG00000009745 | 0.60679419 | 1.084484346 |

| | | | | | |
|---|---|---|---|---|---|
| Wnt signaling pathway | ko04310 | 5 out of 48 | ENSRNOG00000001068;ENSRNOG00000009745;ENSRNOG00000016313;ENSRNOG00000047211;ENSRNOG00000048172 | 0.001692007 | 5.739522417 |
| Axon guidance | ko04360 | 5 out of 48 | ENSRNOG00000001068;ENSRNOG00000001470;ENSRNOG00000009745;ENSRNOG00000047211;ENSRNOG00000048172 | 0.002682102 | 5.165570175 |
| VEGF signaling pathway | ko04370 | 3 out of 48 | ENSRNOG00000001068;ENSRNOG00000009745;ENSRNOG0000004 | 0.00363063 | 9.653688525 |
| Osteoclast differentiation | ko04380 | 2 out of 48 | ENSRNOG00000001068;ENSRNOG00000009745 | 0.138199483 | 3.067057292 |
| Hippo signaling pathway | ko04390 | 1 out of 48 | ENSRNOG00000047211 | 0.561409477 | 1.226822917 |
| Focal adhesion | ko04510 | 2 out of 48 | ENSRNOG00000001068;ENSRNOG00000048172 | 0.280743454 | 1.91504065 |
| Adherens junction | ko04520 | 2 out of 48 | ENSRNOG00000001068;ENSRNOG00000048172 | 0.065252481 | 4.787601626 |
| Tight junction | ko04530 | 2 out of 48 | ENSRNOG00000001068;ONT.1410 | 0.218274595 | 2.28246124 |
| Signaling pathways regulating pluripot | ko04550 | 1 out of 48 | ENSRNOG00000047211 | 0.525890678 | 1.353735632 |
| Toll-like receptor signaling pathway | ko04620 | 1 out of 48 | ENSRNOG00000001068 | 0.376357542 | 2.133605072 |
| NOD-like receptor signaling pathway | ko04621 | 1 out of 48 | ENSRNOG00000001813 | 0.614901689 | 1.061036036 |
| C-type lectin receptor signaling pathwa | ko04625 | 1 out of 48 | ENSRNOG00000009745 | 0.463122215 | 1.622245179 |
| Natural killer cell mediated cytotoxicity | ko04650 | 3 out of 48 | ENSRNOG00000001068;ENSRNOG00000009745;ENSRNOG0000004 | 0.029982974 | 4.427631579 |
| Th1 and Th2 cell differentiation | ko04658 | 1 out of 48 | ENSRNOG00000009745 | 0.425796967 | 1.817515432 |
| Th17 cell differentiation | ko04659 | 1 out of 48 | ENSRNOG00000009745 | 0.484899477 | 1.521640827 |
| T cell receptor signaling pathway | ko04660 | 1 out of 48 | ENSRNOG00000009745 | 0.476836303 | 1.55787037 |
| B cell receptor signaling pathway | ko04662 | 3 out of 48 | ENSRNOG00000001068;ENSRNOG00000009745;ENSRNOG0000004 | 0.017867806 | 5.402522936 |
| Fc epsilon RI signaling pathway | ko04664 | 2 out of 48 | ENSRNOG00000001068;ENSRNOG00000048172 | 0.084195387 | 4.13245614 |
| Fc gamma R-mediated phagocytosis | ko04666 | 2 out of 48 | ENSRNOG00000001068;ENSRNOG00000001470 | 0.131291492 | 3.165994624 |
| TNF signaling pathway | ko04668 | 1 out of 48 | ENSRNOG00000001813 | 0.440444232 | 1.737094395 |
| Leukocyte transendothelial migration | ko04670 | 1 out of 48 | ENSRNOG00000001068 | 0.440444232 | 1.737094395 |
| Long-term potentiation | ko04720 | 1 out of 48 | ENSRNOG00000009745 | 0.30151574 | 2.804166667 |
| Synaptic vesicle cycle | ko04721 | 1 out of 48 | ENSRNOG00000033835 | 0.333139757 | 2.484704641 |
| Neurotrophin signaling pathway | ko04722 | 1 out of 48 | ENSRNOG00000001068 | 0.474121214 | 1.570333333 |
| Glutamatergic synapse | ko04724 | 1 out of 48 | ENSRNOG00000009745 | 0.463122215 | 1.622245179 |
| Dopaminergic synapse | ko04728 | 1 out of 48 | ENSRNOG00000009745 | 0.513436645 | 1.402083333 |
| Regulation of actin cytoskeleton | ko04810 | 3 out of 48 | ENSRNOG00000001068;ENSRNOG00000001470;ENSRNOG0000004 | 0.112855629 | 2.549242424 |
| Insulin signaling pathway | ko04910 | 1 out of 48 | ENSRNOG00000029028 | 0.530784414 | 1.33531746 |
| Melanogenesis | ko04916 | 1 out of 48 | ENSRNOG00000047211 | 0.413809443 | 1.887419872 |
| Adipocytokine signaling pathway | ko04920 | 1 out of 48 | ENSRNOG00000001309 | 0.312216875 | 2.688926941 |
| Oxytocin signaling pathway | ko04921 | 2 out of 48 | ENSRNOG00000001309;ENSRNOG00000009745 | 0.190327681 | 2.500530786 |
| Glucagon signaling pathway | ko04922 | 1 out of 48 | ENSRNOG00000009745 | 0.428756141 | 1.800840979 |
| Renin secretion | ko04924 | 1 out of 48 | ENSRNOG00000009745 | 0.326235669 | 2.549242424 |
| Non-alcoholic fatty liver disease | ko04932 | 1 out of 48 | ENSRNOG00000001068 | 0.600604116 | 1.102762172 |
| AGE-RAGE signaling pathway in diab | ko04933 | 1 out of 48 | ENSRNOG00000001068 | 0.413809443 | 1.887419872 |
| Cushing syndrome | ko04934 | 1 out of 48 | ENSRNOG00000047211 | 0.554520745 | 1.250265393 |
| Endocrine and other factor-regulated ca | ko04961 | 1 out of 48 | ENSRNOG00000033835 | 0.26465453 | 3.271527778 |
| Pancreatic secretion | ko04972 | 1 out of 48 | ENSRNOG00000001068 | 0.428756141 | 1.800840979 |
| Cholesterol metabolism | ko04979 | 2 out of 48 | ENSRNOG00000005331;ENSRNOG00000014765 | 0.030741861 | 7.270061728 |
| Alzheimer disease | ko05010 | 2 out of 48 | ENSRNOG00000009745;ENSRNOG00000047211 | 0.646831972 | 0.919398907 |
| Parkinson disease | ko05012 | 3 out of 48 | ENSRNOG00000004426;ENSRNOG00000015385;ENSRNOG0000003 | 0.181788405 | 2.037629758 |
| Amyotrophic lateral sclerosis | ko05014 | 4 out of 48 | ENSRNOG00000001068;ENSRNOG00000005331;ENSRNOG00000009745;ENSRNOG00000015385 | 0.151051074 | 1.933908046 |
| Prion disease | ko05020 | 2 out of 48 | ENSRNOG00000001068;ENSRNOG00000009745 | 0.468572242 | 1.274621212 |
| Amphetamine addiction | ko05031 | 1 out of 48 | ENSRNOG00000009745 | 0.297912597 | 2.844806763 |

| | | | | | |
|---|---|---|---|---|---|
| Alcoholism | ko05034 | 1 out of 48 | ENSRNOG00000001309 | 0.565943902 | 1.211676955 |
| Bacterial invasion of epithelial cells | ko05100 | 3 out of 48 | ENSRNOG00000001068;ENSRNO G00000033835;ENSRNOG0000005 | 0.006971717 | 7.647727273 |
| Salmonella infection | ko05132 | 2 out of 48 | ENSRNOG00000001068;ENSRNO G00000059705 | 0.385542884 | 1.504150702 |
| Yersinia infection | ko05135 | 4 out of 48 | ENSRNOG00000001068;ENSRNO G00000001470;ENSRNOG0000004 8172;ENSRNOG00000059705 | 0.010955468 | 4.591617934 |
| Tuberculosis | ko05152 | 1 out of 48 | ENSRNOG00000009745 | 0.668980466 | 0.917250779 |
| Human cytomegalovirus infection | ko05163 | 3 out of 48 | ENSRNOG00000001068;ENSRNO G00000009745;ENSRNOG0000004 | 0.135327331 | 2.346115538 |
| Human papillomavirus infection | ko05165 | 1 out of 48 | ENSRNOG00000047211 | 0.83909838 | 0.559235518 |
| Human T-cell leukemia virus 1 infectio | ko05166 | 1 out of 48 | ENSRNOG00000009745 | 0.746618058 | 0.74072327 |
| Kaposi sarcoma-associated herpesvirus | ko05167 | 4 out of 48 | ENSRNOG00000001068;ENSRNO G00000004426;ENSRNOG0000000 9745;ENSRNOG00000034246 | 0.026004156 | 3.536786787 |
| Epstein-Barr virus infection | ko05169 | 2 out of 48 | ENSRNOG00000001068;ONT.1410 | 0.412673178 | 1.422403382 |
| Human immunodeficiency virus 1 infec | ko05170 | 4 out of 48 | ENSRNOG00000001068;ENSRNO G00000001470;ENSRNOG0000000 9745;ENSRNOG00000048172 | 0.037856362 | 3.140666667 |
| Pathways in cancer | ko05200 | 3 out of 48 | ENSRNOG00000001068;ENSRNO G00000047211;ENSRNOG0000004 | 0.560749083 | 1.034929701 |
| Viral carcinogenesis | ko05203 | 1 out of 48 | ENSRNOG00000001068 | 0.653046685 | 0.957520325 |
| Proteoglycans in cancer | ko05205 | 2 out of 48 | ENSRNOG00000001068;ENSRNO G00000047211 | 0.297816547 | 1.834501558 |
| MicroRNAs in cancer | ko05206 | 1 out of 48 | ENSRNOG00000047211 | 0.587936371 | 1.14123062 |
| Colorectal cancer | ko05210 | 2 out of 48 | ENSRNOG00000001068;ENSRNO G00000048172 | 0.090327478 | 3.965488215 |
| Renal cell carcinoma | ko05211 | 1 out of 48 | ENSRNOG00000001068 | 0.343366189 | 2.393800813 |
| Pancreatic cancer | ko05212 | 2 out of 48 | ENSRNOG00000001068;ENSRNO G00000048172 | 0.066651983 | 4.729919679 |
| Basal cell carcinoma | ko05217 | 1 out of 48 | ENSRNOG00000047211 | 0.290651628 | 2.929726368 |
| Breast cancer | ko05224 | 1 out of 48 | ENSRNOG00000047211 | 0.552201044 | 1.258279915 |
| Hepatocellular carcinoma | ko05225 | 1 out of 48 | ENSRNOG00000047211 | 0.612890294 | 1.066802536 |
| Gastric cancer | ko05226 | 1 out of 48 | ENSRNOG00000047211 | 0.554520745 | 1.250265393 |
| Choline metabolism in cancer | ko05231 | 2 out of 48 | ENSRNOG00000001068;ENSRNO G00000048172 | 0.093442402 | 3.886963696 |
| PD-L1 expression and PD-1 checkpoin | ko05235 | 1 out of 48 | ENSRNOG00000009745 | 0.41983352 | 1.851808176 |
| Viral myocarditis | ko05416 | 2 out of 48 | ENSRNOG00000001068;ENSRNO G00000048172 | 0.139939231 | 3.043281654 |
| Fluid shear stress and atherosclerosis | ko05418 | 2 out of 48 | ENSRNOG00000001068;ENSRNO G00000048172 | 0.188482791 | 2.516559829 |

| #Kegg_pathway-red | ko_id | Cluster_fre | GeneID | P-value | rich_factor |
|---|---|---|---|---|---|
| Citrate cycle (TCA cycle) | ko00020 | 1 out of 16 | ENSRNOG00000005686 | 0 | 7.246525604 |
| Oxidative phosphorylation | ko00190 | 31 out of 16 | ENSRNOG00000001170;ENSRNOG00000002721;ENSRNOG00000003626;ENSRNOG00000004526;ENSRNOG00000006939;ENSRNOG00000008569;ENSRNOG00000011825;ENSRNOG00000012383;ENSRNOG00000012550;ENSRNOG00000014078;ENSRNOG00000016660;ENSRNOG00000016952;ENSRNOG00000017817;ENSRNOG00000023387;ENSRNOG00000024309;ENSRNOG00000026616;ENSRNOG00000026646;ENSRNOG00000027049;ENSRNOG00000028717;ENSRNOG00000028884;ENSRNOG00000030237;ENSRNOG00000034161;ENSRNOG00000034182;ENSRNOG00000042903;ENSRNOG00000048174;ENSRNOG00000048320;ENSRNOG00000049394;ENSRNOG00000049912; | 0 | 7.05999157 |
| Purine metabolism | ko00230 | 1 out of 16 | ENSRNOG00000002671 | 1.79E-13 | 7.506971556 |
| Pyrimidine metabolism | ko00240 | 1 out of 16 | ENSRNOG00000002671 | 2.65E-13 | 8.473230345 |
| Cysteine and methionine meta | ko00270 | 2 out of 16 | ENSRNOG00000010625;ENSRNOG00000026649 | 3.71E-13 | 8.600547689 |
| Valine, leucine and isoleucine | ko00280 | 1 out of 16 | ONT.3883 | 4.06E-13 | 11.19946319 |
| Tyrosine metabolism | ko00350 | 1 out of 16 | ENSRNOG00000006589 | 5.27E-13 | 8.767974081 |
| Phenylalanine metabolism | ko00360 | 1 out of 16 | ENSRNOG00000006589 | 6.68E-13 | 5.550236347 |
| Other types of O-glycan biosy | ko00514 | 1 out of 16 | ENSRNOG00000024533 | 8.05E-13 | 5.837317538 |
| Propanoate metabolism | ko00640 | 2 out of 16 | ENSRNOG00000005686;ONT.3883 | 1.94E-09 | 8.840562974 |
| Pantothenate and CoA biosyn | ko00770 | 1 out of 16 | ENSRNOG00000025286 | 3.77E-08 | 5.408531554 |
| Drug metabolism - other enzy | ko00983 | 1 out of 16 | ENSRNOG00000002671 | 1.21E-07 | 7.006506786 |
| Carbon metabolism | ko01200 | 1 out of 16 | ENSRNOG00000005686 | 1.65E-05 | 3.835315326 |
| Ribosome | ko03010 | 73 out of 16 | ENSRNOG00000000490;ENSRNOG00000000926;ENSRNOG00000000957;ENSRNOG00000004107;ENSRNOG00000004214;ENSRNOG00000004426;ENSRNOG00000006898;ENSRNOG00000008546;ENSRNOG00000008555;ENSRNOG00000010746;ENSRNOG00000011494;ENSRNOG00000013508;ENSRNOG00000013845;ENSRNOG00000014272;ENSRNOG00000015989;ENSRNOG00000016387;ENSRNOG00000016961;ENSRNOG00000017127;ENSRNOG00000017552;ENSRNOG00000018471;ENSRNOG00000018774;ENSRNOG00000018795;ENSRNOG00000019106;ENSRNOG00000019578;ENSRNOG00000019970;ENSRNOG00000019974;ENSRNOG00000020354;ENSRNOG00000020982;ENSRNOG00000022609;ENSRNOG00000023385;ENSRNOG00000024845;ENSRNOG00000025388;ENSRNOG00000026260;ENSRNOG00000028021;ENSRNOG00000028690;ENSRNOG00000029410;ENSRNOG00000029512;ENSRNOG00000029627;ENSRNOG00000031022;ENSRNOG00000032605;ENSRNOG00000032803;ENSRNOG00000032847; | 0.110934395 | 3.503253393 |
| RNA transport | ko03013 | 1 out of 16 | ENSRNOG00000017158 | 0.128092492 | 3.21131561 |

| | | | | | |
|---|---|---|---|---|---|
| RNA polymerase | ko03020 | 2 out of 16[illegible] | ENSRNOG00000019293;ENSRNOG00000048562 | 0.307100875 | 2.752556237 |
| Spliceosome | ko03040 | 2 out of 16[illegible] | ENSRNOG00000031127;ONT.3852 | 0.014753116 | 2.715609174 |
| Proteasome | ko03050 | 2 out of 16[illegible] | ENSRNOG00000048470;ENSRNOG00000049229 | 0.176361261 | 2.627440045 |
| Protein export | ko03060 | 1 out of 16[illegible] | ENSRNOG00000018075 | 0.029670615 | 2.352475389 |
| MAPK signaling pathway | ko04010 | 1 out of 16[illegible] | ENSRNOG00000018839 | 0.032170364 | 2.312147239 |
| Ras signaling pathway | ko04014 | 2 out of 16[illegible] | ENSRNOG00000006130;ENSRNOG00000018839 | 0.156526109 | 2.195076493 |
| Rap1 signaling pathway | ko04015 | 1 out of 16[illegible] | ENSRNOG00000008223 | 0.23332286 | 2.18127098 |
| cAMP signaling pathway | ko04024 | 1 out of 16[illegible] | ENSRNOG00000014039 | 0.160691446 | 2.167638037 |
| HIF-1 signaling pathway | ko04066 | 2 out of 16[illegible] | ENSRNOG00000004814;ENSRNOG00000033199 | 0.239740227 | 2.140877073 |
| Neuroactive ligand-receptor i | ko04080 | 1 out of 16[illegible] | ENSRNOG00000008223 | 0.376148516 | 2.140877073 |
| Cell cycle | ko04110 | 1 out of 16[illegible] | ENSRNOG00000051650 | 0.252598889 | 2.064417178 |
| Oocyte meiosis | ko04114 | 1 out of 16[illegible] | ENSRNOG00000051650 | 0.397609077 | 1.993230379 |
| Ubiquitin mediated proteolysi | ko04120 | 7 out of 16[illegible] | ENSRNOG00000004426;ENSRNOG00000004814;ENSRNOG00000019798;ENSRNOG00000019974;ENSRNOG00000033199;ENSRNOG00000034246;ONT.872 | 0.428431738 | 1.806365031 |
| Mitophagy - animal | ko04137 | 3 out of 16[illegible] | ENSRNOG00000004426;ENSRNOG00000019974;ENSRNOG0000003 | 0.485452569 | 1.5211495 |
| Protein processing in endopla | ko04141 | 3 out of 16[illegible] | ENSRNOG00000008604;ENSRNOG00000019798;ENSRNOG0000003 | 0.385978614 | 1.501394311 |
| Lysosome | ko04142 | 1 out of 16[illegible] | ENSRNOG00000019249 | 0.348902225 | 1.433149115 |
| Endocytosis | ko04144 | 4 out of 16[illegible] | ENSRNOG00000006130;ENSRNOG00000014317;ONT.13829;ONT.87 | 0.41642994 | 1.409845878 |
| Phagosome | ko04145 | 14 out of 1[illegible] | ENSRNOG00000003597;ENSRNOG00000006130;ENSRNOG00000010170;ENSRNOG00000017209;ENSRNOG00000017445;ENSRNOG00000017558;ENSRNOG00000018371;ENSRNOG00000021438;ENSRNOG00000028750;ENSRNOG00000032967;ENSRNOG00000046151;ENSRNOG00000047505;ENSRNOG00000048169;ENSRNOG00000053468 | 0.520287985 | 1.376278119 |
| mTOR signaling pathway | ko04150 | 2 out of 16[illegible] | ENSRNOG00000019908;ENSRNOG00000027028 | 0.536816291 | 1.313720022 |
| PI3K-Akt signaling pathway | ko04151 | 2 out of 16[illegible] | ENSRNOG00000018839;ENSRNOG00000051650 | 0.618104614 | 1.050976018 |
| Apoptosis | ko04210 | 7 out of 16[illegible] | ENSRNOG00000003597;ENSRNOG00000021438;ENSRNOG00000028750;ENSRNOG00000032967;ENSRNOG00000048169;ENSRNOG00000053468;ONT.13366 | 0.63769843 | 0.996615189 |
| Cardiac muscle contraction | ko04260 | 12 out of 1[illegible] | ENSRNOG00000001170;ENSRNOG00000004526;ENSRNOG00000012550;ENSRNOG00000016660;ENSRNOG00000016952;ENSRNOG00000017817;ENSRNOG00000024309;ENSRNOG00000030237;ENSRNOG00000034161;ENSRNOG00000042903;ENSRNOG00000048174;ONT | 0.644005046 | 0.979723406 |
| Notch signaling pathway | ko04330 | 1 out of 16[illegible] | ENSRNOG00000005355 | 0.636268258 | 0.932317435 |
| Hippo signaling pathway | ko04390 | 1 out of 16[illegible] | ENSRNOG00000051650 | 0.632125993 | 0.92733178 |
| Tight junction | ko04530 | 7 out of 16[illegible] | ENSRNOG00000003597;ENSRNOG00000021438;ENSRNOG00000028750;ENSRNOG00000032967;ENSRNOG00000048169;ENSRNOG00000053468;ONT.872 | 0.731768858 | 0.811279733 |

| | | | | | |
|---|---|---|---|---|---|
| Gap junction | ko04540 | 13 out of 16 | ENSRNOG00000003597;ENSRNOG00000010170;ENSRNOG00000017209;ENSRNOG00000017445;ENSRNOG00000017558;ENSRNOG00000018371;ENSRNOG00000021438;ENSRNOG00000028750;ENSRNOG00000032967;ENSRNOG00000046151;ENSRNOG00000047505;ENSRNOG00000048169;ENSRNOG000 | 0.743594311 | 0.781130824 |
| Platelet activation | ko04611 | 1 out of 16 | ENSRNOG00000021161 | 0.743464755 | 0.76057475 |
| Renin-angiotensin system | ko04614 | 1 out of 16 | ENSRNOG00000003858 | 0.762601955 | 0.731692164 |
| Cytosolic DNA-sensing pathw | ko04623 | 2 out of 16 | ENSRNOG00000019293;ENSRNOG00000048562 | 0.774666892 | 0.713625691 |
| C-type lectin receptor signalin | ko04625 | 1 out of 16 | ENSRNOG00000017828 | 0.783363365 | 0.700650679 |
| Fc gamma R-mediated phago | ko04666 | 2 out of 16 | ENSRNOG00000014317;ONT.1382 | 0.786196494 | 0.696429891 |
| Thermogenesis | ko04714 | 32 out of 16 | ENSRNOG00000001170;ENSRNOG00000002721;ENSRNOG00000003626;ENSRNOG00000004526;ENSRNOG00000006939;ENSRNOG00000008223;ENSRNOG00000008569;ENSRNOG00000011825;ENSRNOG00000012383;ENSRNOG00000012550;ENSRNOG00000014078;ENSRNOG00000016660;ENSRNOG00000016952;ENSRNOG00000017817;ENSRNOG00000023387;ENSRNOG00000024309;ENSRNOG00000026616;ENSRNOG00000026646;ENSRNOG00000027049;ENSRNOG00000028717;ENSRNOG00000028884;ENSRNOG00000030237;ENSRNOG00000034161;ENSRNOG00000034182;ENSRNOG00000042903;ENSRNOG00000048174;ENSRNOG00000048320;ENSRNOG00000049394;ENSRNOG00000049912;ENSRNO | 0.766566095 | 0.696429891 |
| Synaptic vesicle cycle | ko04721 | 3 out of 16 | ENSRNOG00000003905;ENSRNOG00000014816;ENSRNOG0000001 | 0.802523992 | 0.672135825 |
| Neurotrophin signaling pathw | ko04722 | 1 out of 16 | ENSRNOG00000018839 | 0.834472451 | 0.664410126 |
| Retrograde endocannabinoid s | ko04723 | 16 out of 16 | ENSRNOG00000002721;ENSRNOG00000006939;ENSRNOG00000008223;ENSRNOG00000008569;ENSRNOG00000011825;ENSRNOG00000012383;ENSRNOG00000014078;ENSRNOG00000023387;ENSRNOG00000026616;ENSRNOG00000026646;ENSRNOG00000028717;ENSRNOG00000034182;ENSRNOG00000048320;ENSRNOG00000049394;ENSRNOG00000050514;ONT.7763 | 0.797248055 | 0.635205286 |
| Glutamatergic synapse | ko04724 | 3 out of 16 | ENSRNOG00000014816;ENSRNOG00000016163;ENSRNOG0000005 | 0.874027365 | 0.563938351 |
| GABAergic synapse | ko04727 | 1 out of 16 | ENSRNOG00000003905 | 0.838785603 | 0.555804625 |
| Regulation of actin cytoskelet | ko04810 | 2 out of 16 | ENSRNOG00000014317;ONT.1382 | 0.912735125 | 0.50046477 |

| | | | | | |
|---|---|---|---|---|---|
| Non-alcoholic fatty liver disea | ko04932 | 27 out of 16 | ENSRNOG00000001170;ENSRNOG00000002721;ENSRNOG00000004526;ENSRNOG00000006939;ENSRNOG00000008569;ENSRNOG00000011825;ENSRNOG00000012383;ENSRNOG00000012550;ENSRNOG00000014078;ENSRNOG00000016660;ENSRNOG00000016952;ENSRNOG00000017817;ENSRNOG00000023387;ENSRNOG00000024309;ENSRNOG00000026616;ENSRNOG00000026646;ENSRNOG00000028717;ENSRNOG00000030237;ENSRNOG00000034161;ENSRNOG00000034182;ENSRNOG00000042903;ENSRNOG00000048174;ENSRNOG00000048320;ENSRNOG00000049394;ENSRNOG00000050514;ONT | 0.880602082 | 0.477716372 |
| Vasopressin-regulated water r | ko04962 | 2 out of 163 | ENSRNOG00000003905;ENSRNOG00000006130 | 0.884750529 | 0.469948626 |
| Protein digestion and absorpti | ko04974 | 1 out of 163 | ENSRNOG00000014816 | 0.933673654 | 0.462429448 |
| Alzheimer disease | ko05010 | 41 out of 16 | ENSRNOG00000001170;ENSRNOG00000002721;ENSRNOG00000003597;ENSRNOG00000003626;ENSRNOG00000004526;ENSRNOG00000005355;ENSRNOG00000006939;ENSRNOG00000008569;ENSRNOG00000011825;ENSRNOG00000012383;ENSRNOG00000012550;ENSRNOG00000014078;ENSRNOG00000016660;ENSRNOG00000016952;ENSRNOG00000017209;ENSRNOG00000017445;ENSRNOG00000017558;ENSRNOG00000017817;ENSRNOG00000018371;ENSRNOG00000021438;ENSRNOG00000023387;ENSRNOG00000024309;ENSRNOG00000026616;ENSRNOG00000026646;ENSRNOG00000028717;ENSRNOG00000028750;ENSRNOG00000030237;ENSRNOG00000032967;ENSRNOG00000034161;ENSRNOG00000034182;ENSRNOG00000042903;ENSRNOG00000046151;ENSRNOG00000047505;ENSRNOG00000048169;ENSRNOG00000048174;ENSRNOG00000048320;ENSRNOG00000049394;ENSRNOG0000004 | 0.888755694 | 0.462429448 |

| | | | | | |
|---|---|---|---|---|---|
| Parkinson disease | ko05012 | 43 out of 1( | ENSRNOG00000001170;ENSRNOG00000002721;ENSRNOG00000003597;ENSRNOG00000003626;ENSRNOG00000004426;ENSRNOG00000004526;ENSRNOG00000006939;ENSRNOG00000008569;ENSRNOG00000011825;ENSRNOG00000012383;ENSRNOG00000012550;ENSRNOG00000014078;ENSRNOG00000016660;ENSRNOG00000016952;ENSRNOG00000017209;ENSRNOG00000017445;ENSRNOG00000017558;ENSRNOG00000017817;ENSRNOG00000018371;ENSRNOG00000019974;ENSRNOG00000021438;ENSRNOG00000023387;ENSRNOG00000024309;ENSRNOG00000026616;ENSRNOG00000026646;ENSRNOG00000028717;ENSRNOG00000028750;ENSRNOG00000030237;ENSRNOG00000032967;ENSRNOG00000034161;ENSRNOG00000034182;ENSRNOG00000034246;ENSRNOG00000042903;ENSRNOG00000046151;ENSRNOG00000047505;ENSRNOG00000048169;ENSRNOG00000048174;ENSRNOG00000048320;ENSRNOG00000049394;ENS | 0.937433331 | 0.455147094 |
| Amyotrophic lateral sclerosis | ko05014 | 41 out of 1( | ENSRNOG00000001170;ENSRNOG00000002721;ENSRNOG00000003597;ENSRNOG00000003626;ENSRNOG00000004526;ENSRNOG00000006939;ENSRNOG00000008569;ENSRNOG00000011825;ENSRNOG00000012383;ENSRNOG00000012550;ENSRNOG00000014078;ENSRNOG00000016660;ENSRNOG00000016952;ENSRNOG00000017209;ENSRNOG00000017445;ENSRNOG00000017558;ENSRNOG00000017817;ENSRNOG00000018371;ENSRNOG00000021438;ENSRNOG00000021954;ENSRNOG00000023387;ENSRNOG00000024309;ENSRNOG00000026616;ENSRNOG00000026646;ENSRNOG00000028717;ENSRNOG00000028750;ENSRNOG00000030237;ENSRNOG00000032967;ENSRNOG00000034161;ENSRNOG00000034182;ENSRNOG00000042903;ENSRNOG00000046151;ENSRNOG00000047505;ENSRNOG00000048169;ENSRNOG00000048174;ENSRNOG00000048320;ENSRNOG00000049394;ENSRNOG0000004 | 0.894505499 | 0.451591258 |

| | | | | | |
|---|---|---|---|---|---|
| Huntington disease | ko05016 | 43 out of 16 | ENSRNOG00000001170;ENSRNOG00000002721;ENSRNOG00000003597;ENSRNOG00000003626;ENSRNOG00000004526;ENSRNOG00000006939;ENSRNOG00000008569;ENSRNOG00000011825;ENSRNOG00000012383;ENSRNOG00000012550;ENSRNOG00000014078;ENSRNOG00000016163;ENSRNOG00000016660;ENSRNOG00000016952;ENSRNOG00000017209;ENSRNOG00000017445;ENSRNOG00000017558;ENSRNOG00000017817;ENSRNOG00000018371;ENSRNOG00000019293;ENSRNOG00000021438;ENSRNOG00000023387;ENSRNOG00000024309;ENSRNOG00000026616;ENSRNOG00000026646;ENSRNOG00000028717;ENSRNOG00000028750;ENSRNOG00000030237;ENSRNOG00000032967;ENSRNOG00000034161;ENSRNOG00000034182;ENSRNOG00000042903;ENSRNOG00000046151;ENSRNOG00000047505;ENSRNOG00000048169;ENSRNOG00000048174;ENSRNOG00000048320;ENSRNOG00000048562;ENSRNOG00000049394;ENS | 0.894505499 | 0.451591258 |
| Prion disease | ko05020 | 40 out of 16 | ENSRNOG00000001170;ENSRNOG00000002721;ENSRNOG00000003597;ENSRNOG00000003626;ENSRNOG00000004526;ENSRNOG00000006939;ENSRNOG00000008569;ENSRNOG00000011825;ENSRNOG00000012383;ENSRNOG00000012550;ENSRNOG00000014078;ENSRNOG00000016660;ENSRNOG00000016952;ENSRNOG00000017209;ENSRNOG00000017445;ENSRNOG00000017558;ENSRNOG00000017817;ENSRNOG00000018371;ENSRNOG00000021438;ENSRNOG00000023387;ENSRNOG00000024309;ENSRNOG00000026616;ENSRNOG00000026646;ENSRNOG00000028717;ENSRNOG00000028750;ENSRNOG00000030237;ENSRNOG00000032967;ENSRNOG00000034161;ENSRNOG00000034182;ENSRNOG00000042903;ENSRNOG00000046151;ENSRNOG00000047505;ENSRNOG00000048169;ENSRNOG00000048174;ENSRNOG00000048320;ENSRNOG00000049394;ENSRNO | 0.896355683 | 0.44809055 |
| Alcoholism | ko05034 | 2 out of 16 | ENSRNOG00000006532;ENSRNOG00000018839 | 0.898173613 | 0.4446437 |
| Bacterial invasion of epithelia | ko05100 | 2 out of 16 | ENSRNOG00000014317;ONT.1382 | 0.911621016 | 0.418867253 |
| Salmonella infection | ko05132 | 3 out of 16 | ENSRNOG00000006130;ENSRNOG00000014317;ONT.13829 | 0.913172694 | 0.41585382 |
| Amoebiasis | ko05146 | 1 out of 16 | ENSRNOG00000006130 | 0.92727503 | 0.387944168 |
| Tuberculosis | ko05152 | 1 out of 16 | ENSRNOG00000006130 | 0.940170315 | 0.361273006 |
| Hepatitis C | ko05160 | 1 out of 16 | ENSRNOG00000051650 | 0.941223245 | 0.359029074 |
| Hepatitis B | ko05161 | 2 out of 16 | ENSRNOG00000017828;ENSRNOG00000051650 | 0.947165051 | 0.346129826 |
| Human papillomavirus infecti | ko05165 | 2 out of 16 | ENSRNOG00000006494;ENSRNOG00000020213 | 0.985573678 | 0.329365704 |

| | | | | | |
|---|---|---|---|---|---|
| Kaposi sarcoma-associated he | ko05167 | 3 out of 16: | ENSRNOG00000004426;ENSRNOG00000019974;ENSRNOG0000003 | 0.991804581 | 0.298727033 |
| Epstein-Barr virus infection | ko05169 | 1 out of 16: | ONT.872 | 0.968465486 | 0.29491674 |
| Human immunodeficiency vir | ko05170 | 2 out of 16: | ENSRNOG00000004814;ENSRNOG00000033199 | 0.977127693 | 0.270110659 |
| Pathways in cancer | ko05200 | 2 out of 16: | ENSRNOG00000004814;ENSRNOG00000033199 | 0.977127693 | 0.270110659 |
| Transcriptional misregulation | ko05202 | 1 out of 16: | ENSRNOG00000006532 | 0.979816571 | 0.261555118 |
| Viral carcinogenesis | ko05203 | 2 out of 16: | ENSRNOG00000017828;ENSRNOG00000051650 | 0.981869658 | 0.254641766 |
| Proteoglycans in cancer | ko05205 | 1 out of 16: | ENSRNOG00000011076 | 0.986864248 | 0.235933392 |
| MicroRNAs in cancer | ko05206 | 2 out of 16: | ENSRNOG00000010625;ENSRNOG00000026649 | 0.992470301 | 0.209433627 |
| Renal cell carcinoma | ko05211 | 2 out of 16: | ENSRNOG00000004814;ENSRNOG00000033199 | 0.999585078 | 0.203176383 |
| Systemic lupus erythematosus | ko05322 | 1 out of 16: | ENSRNOG00000006532 | 0.995919063 | 0.186463487 |
| Viral myocarditis | ko05416 | 1 out of 16: | ENSRNOG00000017158 | 0.998288033 | 0.161462796 |
