# Supplementary material for: Effects of Maternal High-Fructose Diet on Long Non-Coding RNAs and Anxiety-like Behaviors in Offspring
Source: Int J Mol Sci. 2023 Feb 24;24(5):4460. doi: 10.3390/ijms24054460 (PMC10003385; doi:10.3390/ijms24054460)
Supplement: Supplementary file 1 [file ijms-24-04460-s001.zip › Table S7.pdf]

Table S7: The full KEGG pathway enrichment results of cis co-expression clusters in F40%.

Term	Count	GeneID	Pvalue	group
response to drug	1	ENSRNOG00000018294	3.70E-08	BP
brain development	2	ENSRNOG00000018294;ENSRNOG00000048174	2.20E-06	BP
protein ubiquitination	1	ENSRNOG00000018294	3.90E-06	BP
hippocampus development	1	ENSRNOG00000048174	9.00E-06	BP
primary metabolic process	30	ENSRNOG00000002116;ENSRNOG00000006228;ENSRNOG00000009078;ENSRNOG00000009823;ENSRNOG00000011955;ENSRNOG00000018294;ENSRNOG00000019974;ENSRNOG00000021153;ENSRNOG00000023385;ENSRNOG00000027049;ENSRNOG00000029410;ENSRNOG00000030237;ENSRNOG00000031022;ENSRNOG00000031127;ENSRNOG00000031315;ENSRNOG00000033803;ENSRNOG00000033916;ENSRNOG00000034180;ENSRNOG00000037607;ENSRNOG00000038074;ENSRNOG00000048174;ENSRNOG00000048958;ENSRNOG00000049848;ENSRNOG00000049912;ENSRNOG00000049921;ENSRNOG00000060449;ONT.14316;O	1.00E-05	BP
nucleus	9	ENSRNOG00000009823;ENSRNOG00000018294;ENSRNOG00000023385;ENSRNOG00000027049;ENSRNOG00000029410;ENSRNOG00000031022;ENSRNOG00000031127;ENSRNOG00000031315;ENSRNO	2.00E-21	CC
nucleoplasm	1	ENSRNOG00000031127	1.30E-19	CC
perinuclear region of cytoplasm	1	ENSRNOG00000018294	2.70E-14	CC
extracellular vesicular exosome	10	ENSRNOG00000002116;ENSRNOG00000011825;ENSRNOG00000011955;ENSRNOG00000018294;ENSRNOG00000021153;ENSRNOG00000023385;ENSRNOG00000027049;ENSRNOG00000031127;ENSRNOG00000037607;ENSRNOG0000003	1.20E-12	CC
cell junction	6	ENSRNOG00000002116;ENSRNOG00000011955;ENSRNOG00000018294;ENSRNOG00000023385;ENSRNOG00000037607;ENSRNOG000	2.30E-09	CC
ATP binding	1	ENSRNOG00000018294	3.00E-18	MF
metal ion binding	9	ENSRNOG00000002116;ENSRNOG00000006228;ENSRNOG00000023385;ENSRNOG00000033803;ENSRNOG00000034180;ENSRNOG00000037607;ENSRNOG00000038074;ENSRNOG00000048958;ONT.6961	1.00E-11	MF

binding	24	ENSRNOG00000002116;ENSRNOG00000006228;ENSRNOG00000009078;ENSRNOG00000009823;ENSRNOG00000011955;ENSRNOG00000012163;ENSRNOG00000018294;ENSRNOG00000021153;ENSRNOG00000023385;ENSRNOG00000029410;ENSRNOG00000031022;ENSRNOG00000031127;ENSRNOG00000031315;ENSRNOG00000033803;ENSRNOG00000033916;ENSRNOG00000034180;ENSRNOG00000037607;ENSRNOG00000038074;ENSRNOG00000048174;ENSRNOG00000048958;ENSRNOG00000049848;ENSRNOG00000049921;ENSRNO	4.50E-08	MF
catalytic activity	11	ENSRNOG00000006228;ENSRNOG00000009823;ENSRNOG00000018294;ENSRNOG00000021153;ENSRNOG00000027049;ENSRNOG00000029410;ENSRNOG00000030237;ENSRNOG00000048174;ENSRNOG00000049848;ENSRNOG0000004	1.70E-07	MF
protein domain specific binding	1	ENSRNOG00000018294	4.70E-06	MF

Term	Count	GeneID	Pvalue	group
positive regulation of transcription frc	3	ENSRNOG00000008658;ENSRNOG00000012073;ENSRNOG0000001	1.00E-15	BP
response to drug	1	ENSRNOG00000004327	3.30E-08	BP
neuron migration	1	ENSRNOG00000042753	1.80E-06	BP
intracellular signal transduction	12	ENSRNOG00000001931;ENSRNOG00000003033;ENSRNOG00000004143;ENSRNOG00000009288;ENSRNOG00000012278;ENSRNOG00000019183;ENSRNOG00000020873;ENSRNOG00000020899;ENSRNOG00000042753;ENSRNOG00000046962;ENSRNOG00000048389;ENS	3.10E-06	BP
in utero embryonic development	1	ENSRNOG00000003033	3.70E-06	BP
nucleus	16	ENSRNOG00000000566;ENSRNOG00000001931;ENSRNOG00000008658;ENSRNOG00000009288;ENSRNOG00000012073;ENSRNOG00000012278;ENSRNOG00000014882;ENSRNOG00000015329;ENSRNOG00000019317;ENSRNOG00000020737;ENSRNOG00000021168;ENSRNOG00000042753;ENSRNOG00000048226;ENSRNOG00000048389;ENSRNOG00000050978;ENSRNO	1.90E-20	CC
nucleoplasm	2	ENSRNOG00000000566;ENSRNOG00000015329	1.80E-19	CC
neuronal cell body	2	ENSRNOG00000004327;ENSRNOG00000021056	5.10E-16	CC
perinuclear region of cytoplasm	1	ENSRNOG00000058372	1.30E-14	CC
extracellular vesicular exosome	9	ENSRNOG00000000566;ENSRNOG00000002947;ENSRNOG00000003098;ENSRNOG00000003217;ENSRNOG00000004327;ENSRNOG00000009889;ENSRNOG00000012073;ENSRNOG00000020873;ENSRNO	1.70E-12	CC
ATP binding	1	ENSRNOG00000003105	2.60E-18	MF
zinc ion binding	1	ONT.66	1.80E-13	MF
protein homodimerization activity	1	ENSRNOG00000001324	2.10E-13	MF
identical protein binding	3	ENSRNOG00000000566;ENSRNOG00000001324;ENSRNOG0000000	1.00E-11	MF

metal ion binding	7	ENSRNOG00000003033;ENSRNOG00000004143;ENSRNOG00000009889;ENSRNOG00000012236;ENSRNOG00000019183;ENSRNOG00000052051;ONT.66	2.90E-11	MF

Term	Count	GeneID	Pvalue	group
positive regulation of transcription frc	13	ENSRNOG00000000438;ENSRNOG00000002332;ENSRNOG00000003443;ENSRNOG00000004642;ENSRNOG00000008282;ENSRNOG00000008785;ENSRNOG00000009145;ENSRNOG00000015822;ENSRNOG00000016299;ENSRNOG00000018532;ENSRNOG00000020060;ENSRNOG00000020369;ENSRNOG000	1.90E-15	BP
protein ubiquitination	34	ENSRNOG00000000036;ENSRNOG00000000438;ENSRNOG00000001844;ENSRNOG00000001878;ENSRNOG00000002214;ENSRNOG00000003334;ENSRNOG00000004193;ENSRNOG00000004218;ENSRNOG00000005215;ENSRNOG00000005342;ENSRNOG00000005371;ENSRNOG00000006224;ENSRNOG00000006335;ENSRNOG00000006515;ENSRNOG00000007981;ENSRNOG00000008421;ENSRNOG00000010453;ENSRNOG00000010959;ENSRNOG00000015418;ENSRNOG00000016422;ENSRNOG00000017365;ENSRNOG00000018867;ENSRNOG00000019390;ENSRNOG00000020088;ENSRNOG00000020105;ENSRNOG00000020302;ENSRNOG00000020880;ENSRNOG00000022772;ENSRNOG00000025868;ENSRNOG00000029441;ENSRNOG00000031100;ENSRNOG00000032419;ENS	2.90E-08	BP
response to drug	5	ENSRNOG00000011648;ENSRNOG00000020129;ENSRNOG00000020369;ENSRNOG00000029096;ENSRNOG00000029811	1.00E-06	BP
intracellular signal transduction	13	ENSRNOG00000000438;ENSRNOG00000000704;ENSRNOG00000001092;ENSRNOG00000005271;ENSRNOG00000005342;ENSRNOG00000005506;ENSRNOG00000016299;ENSRNOG00000018524;ENSRNOG00000020299;ENSRNOG00000020369;ENSRNOG00000042353;ENSRNOG00000047957;ENSRNOG000	1.40E-06	BP
protein autophosphorylation	1	ENSRNOG00000003742	3.70E-06	BP

nucleus	66	ENSRNOG00000000438;ENSRNOG00000002163;ENSRNOG00000002214;ENSRNOG00000002332;ENSRNOG00000002506;ENSRNOG00000003443;ENSRNOG00000003742;ENSRNOG00000004474;ENSRNOG00000004642;ENSRNOG00000005271;ENSRNOG00000005342;ENSRNOG00000005506;ENSRNOG00000005513;ENSRNOG00000006118;ENSRNOG00000006122;ENSRNOG00000006515;ENSRNOG00000007922;ENSRNOG00000008282;ENSRNOG00000008785;ENSRNOG00000009052;ENSRNOG00000009145;ENSRNOG00000010267;ENSRNOG00000010453;ENSRNOG00000010760;ENSRNOG00000011648;ENSRNOG00000011859;ENSRNOG00000012364;ENSRNOG00000014997;ENSRNOG00000015768;ENSRNOG00000015822;ENSRNOG00000016299;ENSRNOG00000016478;ENSRNOG00000017156;ENSRNOG00000018524;ENSRNOG00000018532;ENSRNOG00000019479;ENSRNOG00000019902;ENSRNOG00000020060;ENSRNOG00000020274;ENSRNOG00000022772;ENSRNOG00000023356;ENSRNOG00000025651;	4.70E-22	CC
nucleoplasm	11	ENSRNOG00000002214;ENSRNOG00000003742;ENSRNOG00000005506;ENSRNOG00000005513;ENSRNOG00000008785;ENSRNOG00000009145;ENSRNOG00000010453;ENSRNOG00000016299;ENSRNOG00000020060;ENSRNOG00000020274;ENSRNOG00000031927	1.10E-17	CC
Cul3-RING ubiquitin ligase complex	30	ENSRNOG00000000036;ENSRNOG00000001844;ENSRNOG00000001878;ENSRNOG00000002214;ENSRNOG00000003334;ENSRNOG00000004193;ENSRNOG00000004218;ENSRNOG00000005215;ENSRNOG00000005371;ENSRNOG00000006224;ENSRNOG00000006335;ENSRNOG00000006515;ENSRNOG00000007981;ENSRNOG00000008421;ENSRNOG00000010453;ENSRNOG00000010959;ENSRNOG00000015418;ENSRNOG00000016422;ENSRNOG00000017365;ENSRNOG00000018867;ENSRNOG00000019390;ENSRNOG00000020088;ENSRNOG00000020105;ENSRNOG00000020302;ENSRNOG00000020880;ENSRNOG00000025868;ENSRNOG00000029441;ENSRNOG00000031100;	1.10E-15	CC
perinuclear region of cytoplasm	7	ENSRNOG00000002332;ENSRNOG00000005299;ENSRNOG00000006122;ENSRNOG00000010453;ENSRNOG00000014997;ENSRNOG00000018524;ENSRNOG00000030212	4.10E-14	CC

neuronal cell body	9	ENSRNOG00000005299;ENSRNOG00000011572;ENSRNOG00000011648;ENSRNOG00000011859;ENSRNOG00000015418;ENSRNOG00000016478;ENSRNOG00000020302;ENSRNOG00000031100;ENSRNO	5.60E-14 CC
ATP binding	13	ENSRNOG00000001214;ENSRNOG00000002506;ENSRNOG00000003742;ENSRNOG00000005299;ENSRNOG00000005530;ENSRNOG00000008625;ENSRNOG00000027911;ENSRNOG00000029096;ENSRNOG00000038720;ENSRNOG00000040266;ENSRNOG00000042353;ENSRNOG00000050277;ENSRNOG000	4.90E-19 MF
protein homodimerization activity	7	ENSRNOG00000007310;ENSRNOG00000010453;ENSRNOG00000026870;ENSRNOG00000029441;ENSRNOG00000029811;ENSRNOG00000058714;ENSRNOG00000061739	6.90E-14 MF
zinc ion binding	13	ENSRNOG00000000438;ENSRNOG00000004089;ENSRNOG00000004642;ENSRNOG00000012364;ENSRNOG00000018532;ENSRNOG00000021896;ENSRNOG00000022417;ENSRNOG00000022772;ENSRNOG00000040208;ENSRNOG00000047028;ENSRNOG00000047940;ENSRNOG00000047957;ENSRNOG000	1.00E-13 MF
identical protein binding	17	ENSRNOG00000000438;ENSRNOG00000001214;ENSRNOG00000001926;ENSRNOG00000004193;ENSRNOG00000007310;ENSRNOG00000010263;ENSRNOG00000010453;ENSRNOG00000014997;ENSRNOG00000026870;ENSRNOG00000029441;ENSRNOG00000029811;ENSRNOG00000047957;ENSRNOG00000050123;ENSRNOG00000054495;ENSRNOG00000055138;ENSRNOG00000058714;ENSRNOG0000006	2.50E-12 MF

metal ion binding	42	ENSRNOG00000000017;ENSRNOG00000000438;ENSRNOG00000001214;ENSRNOG00000002163;ENSRNOG00000003443;ENSRNOG00000004089;ENSRNOG00000004642;ENSRNOG00000005342;ENSRNOG00000006118;ENSRNOG00000008625;ENSRNOG00000008785;ENSRNOG00000009145;ENSRNOG00000011976;ENSRNOG00000012364;ENSRNOG00000015822;ENSRNOG00000016299;ENSRNOG00000018532;ENSRNOG00000018864;ENSRNOG00000019479;ENSRNOG00000020129;ENSRNOG00000021896;ENSRNOG00000022417;ENSRNOG00000022772;ENSRNOG00000023356;ENSRNOG00000025651;ENSRNOG00000027557;ENSRNOG00000029237;ENSRNOG00000033694;ENSRNOG00000038202;ENSRNOG00000039057;ENSRNOG00000040208;ENSRNOG00000042496;ENSRNOG00000046242;ENSRNOG00000046297;ENSRNOG00000047028;ENSRNOG00000047940;ENSRNOG00000047957;ENSRNOG00000048630;ENSRNOG00000049279;ENS	4.40E-12	MF

#Kegg_pathway-yellow	ko_id	Cluster_fre	GeneID	P-value	rich_factor
Tight junction	ko04530	18 out of 96	ENSRNOG00000001419;ENSRNOG00000001691;ENSRNOG00000001926;ENSRNOG00000007922;ENSRNOG00000010085;ENSRNOG00000010263;ENSRNOG00000011424;ENSRNOG00000018524;ENSRNOG00000023531;ENSRNOG00000027691;ENSRNOG00000028216;ENSRNOG00000030386;ENSRNOG00000030889;ENSRNOG00000031521;ENSRNOG00000054080;ENSRNOG00000054495;ENSRNOG0000005	7.13E-14	10.27107558
Leukocyte transendothelial migration	ko04670	18 out of 96	ENSRNOG00000001419;ENSRNOG00000001691;ENSRNOG00000001926;ENSRNOG00000005342;ENSRNOG00000007922;ENSRNOG00000010085;ENSRNOG00000010263;ENSRNOG00000011424;ENSRNOG00000016695;ENSRNOG00000018524;ENSRNOG00000023531;ENSRNOG00000027691;ENSRNOG00000030386;ENSRNOG00000030889;ENSRNOG00000031521;ENSRNOG00000054495;ENSRNOG0000005	1.18E-13	15.63384956
Cell adhesion molecules	ko04514	16 out of 96	ENSRNOG00000001419;ENSRNOG00000001691;ENSRNOG00000001926;ENSRNOG00000007922;ENSRNOG00000010085;ENSRNOG00000010263;ENSRNOG00000011424;ENSRNOG00000020129;ENSRNOG00000023531;ENSRNOG00000027691;ENSRNOG00000030386;ENSRNOG00000030889;ENSRNOG00000031521;ENSRNOG00000054495;ENSRNOG00000055138;ENSRNO	3.61E-11	8.581056466
Hepatitis C	ko05160	15 out of 96	ENSRNOG00000001419;ENSRNOG00000001691;ENSRNOG00000001926;ENSRNOG00000007922;ENSRNOG00000010085;ENSRNOG00000010263;ENSRNOG00000011424;ENSRNOG00000023531;ENSRNOG00000027691;ENSRNOG00000030386;ENSRNOG00000030889;ENSRNOG00000031521;ENSRNOG00000054495;ENSRNOG00000055138;	1.07E-10	8.815494012
Renin-angiotensin system	ko04614	6 out of 96	ENSRNOG00000029237;ENSRNOG00000032857;ENSRNOG00000033414;ENSRNOG00000046297;ENSRNOG00000048630;ENSRNOG000	2.03E-06	15.49671053
Endocrine and other factor-regulated calc	ko04961	7 out of 96	ENSRNOG00000001092;ENSRNOG00000029237;ENSRNOG00000032857;ENSRNOG00000033414;ENSRNOG00000046297;ENSRNOG00000048630;ENSRNOG00000049279	2.27E-06	11.45034722
Malaria	ko05144	4 out of 96	ENSRNOG00000007310;ENSRNOG00000007811;ENSRNOG00000057410;ENSRNOG00000061739	0.005554178	5.608333333
Gastric acid secretion	ko04971	4 out of 96	ENSRNOG00000014347;ENSRNOG00000018524;ENSRNOG00000029811;ENSRNOG00000038202	0.007084472	5.234444444

Parkinson disease	ko05012	8 out of 96	ENSRNOG00000004680;ENSRNOG00000005299;ENSRNOG00000011572;ENSRNOG00000017466;ENSRNOG00000018101;ENSRNOG00000018168;ENSRNOG00000020299;ENSRNOG00000038202	0.009145485	2.716839677
Prion disease	ko05020	7 out of 96	ENSRNOG00000004680;ENSRNOG00000005299;ENSRNOG00000011572;ENSRNOG00000017466;ENSRNOG00000018101;ENSRNOG00000018168;ENSRNOG00000020299	0.037433621	2.230587121
Bile secretion	ko04976	3 out of 96	ENSRNOG00000010378;ENSRNOG00000011648;ENSRNOG0000001	0.049472629	3.635030864
Dopaminergic synapse	ko04728	4 out of 96	ENSRNOG00000004680;ENSRNOG00000005299;ENSRNOG00000017466;ENSRNOG00000038202	0.054449422	2.804166667
Huntington disease	ko05016	7 out of 96	ENSRNOG00000004680;ENSRNOG00000005299;ENSRNOG00000011572;ENSRNOG00000017466;ENSRNOG00000018101;ENSRNOG00000018168;ENSRNOG00000020299	0.060354374	2.00297619
Alanine, aspartate and glutamate metabol	ko00250	2 out of 96	ENSRNOG00000008625;ENSRNOG00000023814	0.062413487	4.907291667
Alzheimer disease	ko05010	8 out of 96	ENSRNOG00000004680;ENSRNOG00000005299;ENSRNOG00000011572;ENSRNOG00000017466;ENSRNOG00000018101;ENSRNOG00000018168;ENSRNOG00000020299;ENSRNOG00000038202	0.069151535	1.838797814
Endocytosis	ko04144	6 out of 96	ENSRNOG00000004680;ENSRNOG00000005299;ENSRNOG00000007806;ENSRNOG00000014997;ENSRNOG00000017466;ENSRNOG000	0.070486694	2.06622807
Spliceosome	ko03040	4 out of 96	ENSRNOG00000005513;ENSRNOG00000010587;ENSRNOG00000024170;ENSRNOG00000040045	0.077541612	2.484704641
Basal transcription factors	ko03022	2 out of 96	ENSRNOG00000018027;ENSRNOG00000020274	0.08571589	4.089409722
Wnt signaling pathway	ko04310	4 out of 96	ENSRNOG00000012364;ENSRNOG00000014180;ENSRNOG00000022417;ENSRNOG00000022772	0.096825879	2.295808967
Ras signaling pathway	ko04014	5 out of 96	ENSRNOG00000005271;ENSRNOG00000005342;ENSRNOG00000020369;ENSRNOG00000022631;ENSRNOG00000038202	0.117117936	1.932004593
Amyotrophic lateral sclerosis	ko05014	7 out of 96	ENSRNOG00000004680;ENSRNOG00000005299;ENSRNOG00000011572;ENSRNOG00000017466;ENSRNOG00000018101;ENSRNOG00000018168;ENSRNOG00000020299	0.119715652	1.69216954
Salmonella infection	ko05132	5 out of 96	ENSRNOG00000011572;ENSRNOG00000018101;ENSRNOG00000018168;ENSRNOG00000020299;ENSRNOG00000042353	0.12730378	1.880188378
Natural killer cell mediated cytotoxicity	ko04650	3 out of 96	ENSRNOG00000055196;ENSRNOG00000060246;ENSRNOG0000006	0.153865539	2.213815789
Graft-versus-host disease	ko05332	2 out of 96	ENSRNOG00000055196;ENSRNOG00000060246	0.166701784	2.726273148
Estrogen signaling pathway	ko04915	3 out of 96	ENSRNOG00000016695;ENSRNOG00000038202;ENSRNOG0000004	0.178692792	2.059003497
Signaling pathways regulating pluripoten	ko04550	3 out of 96	ENSRNOG00000004768;ENSRNOG00000016299;ENSRNOG0000001	0.18378726	2.030603448
Renin secretion	ko04924	2 out of 96	ENSRNOG00000011648;ENSRNOG00000038202	0.184928284	2.549242424
Prolactin signaling pathway	ko04917	2 out of 96	ENSRNOG00000008282;ENSRNOG00000057557	0.192299402	2.484704641

Salivary secretion	ko04970	2 out of 96	ENSRNOG00000014347;ENSRNOG00000038202	0.192299402	2.484704641
Pentose and glucuronate interconversions	ko00040	1 out of 96	ENSRNOG00000001092	0.19369612	4.673611111
Rap1 signaling pathway	ko04015	4 out of 96	ENSRNOG00000005271;ENSRNOG00000005342;ENSRNOG00000022631;ENSRNOG00000038202	0.200793502	1.729441997
Proximal tubule bicarbonate reclamation	ko04964	1 out of 96	ENSRNOG00000011648	0.201929837	4.461174242
Fluid shear stress and atherosclerosis	ko05418	3 out of 96	ENSRNOG00000014205;ENSRNOG00000016695;ENSRNOG0000003	0.212459058	1.887419872
Complement and coagulation cascades	ko04610	2 out of 96	ENSRNOG00000014118;ENSRNOG00000057855	0.240963628	2.133605072
Maturity onset diabetes of the young	ko04950	1 out of 96	ENSRNOG00000020420	0.241866747	3.635030864
Phototransduction	ko04744	1 out of 96	ENSRNOG00000038202	0.249613505	3.505208333
GnRH signaling pathway	ko04912	2 out of 96	ENSRNOG00000016695;ENSRNOG00000038202	0.259875792	2.02362543
Galactose metabolism	ko00052	1 out of 96	ENSRNOG00000001214	0.272386857	3.165994624
Antifolate resistance	ko01523	1 out of 96	ENSRNOG00000019902	0.272386857	3.165994624
TGF-beta signaling pathway	ko04350	2 out of 96	ENSRNOG00000010760;ENSRNOG00000050123	0.275023989	1.943481848
Small cell lung cancer	ko05222	2 out of 96	ENSRNOG00000011300;ENSRNOG00000047957	0.275023989	1.943481848
Glycolysis / Gluconeogenesis	ko00010	2 out of 96	ENSRNOG00000001214;ENSRNOG00000020420	0.278810248	1.924428105
Pentose phosphate pathway	ko00030	1 out of 96	ENSRNOG00000001214	0.279824921	3.067057292
Cellular senescence	ko04218	3 out of 96	ENSRNOG00000005342;ENSRNOG00000010760;ENSRNOG0000003	0.280576445	1.626726519
Antigen processing and presentation	ko04612	2 out of 96	ENSRNOG00000055196;ENSRNOG00000060246	0.301492558	1.817515432
Glucagon signaling pathway	ko04922	2 out of 96	ENSRNOG00000001214;ENSRNOG00000038202	0.305263809	1.800840979
Parathyroid hormone synthesis, secretion	ko04928	2 out of 96	ENSRNOG00000001092;ENSRNOG00000047028	0.312795499	1.768393393
TNF signaling pathway	ko04668	2 out of 96	ENSRNOG00000042353;ENSRNOG00000047957	0.320310688	1.737094395
Fructose and mannose metabolism	ko00051	1 out of 96	ENSRNOG00000001214	0.322899125	2.582785088
Biosynthesis of amino acids	ko01230	2 out of 96	ENSRNOG00000001214;ENSRNOG00000020420	0.339010906	1.663488701
Pyruvate metabolism	ko00620	1 out of 96	ENSRNOG00000020420	0.34347002	2.393800813
Pathways in cancer	ko05200	7 out of 96	ENSRNOG00000005342;ENSRNOG00000011300;ENSRNOG00000016695;ENSRNOG00000020369;ENSRNOG00000022631;ENSRNOG00000038202;ENSRNOG00000047957	0.359904954	1.207417985
Bladder cancer	ko05219	1 out of 96	ENSRNOG00000016695	0.363422268	2.230587121
Proteoglycans in cancer	ko05205	3 out of 96	ENSRNOG00000016695;ENSRNOG00000018524;ENSRNOG0000002	0.37280083	1.375876168
AMPK signaling pathway	ko04152	2 out of 96	ENSRNOG00000001214;ENSRNOG00000011603	0.379569758	1.521640827
Type II diabetes mellitus	ko04930	1 out of 96	ENSRNOG00000020420	0.395351012	2.00297619
Ether lipid metabolism	ko00565	1 out of 96	ENSRNOG00000004089	0.401543939	1.962916667
Insulin signaling pathway	ko04910	2 out of 96	ENSRNOG00000020420;ENSRNOG00000038202	0.443572566	1.33531746
Pyrimidine metabolism	ko00240	1 out of 96	ENSRNOG00000050277	0.454529668	1.663488701
Apelin signaling pathway	ko04371	2 out of 96	ENSRNOG00000014205;ENSRNOG00000038202	0.464112163	1.28295207
Glycerolipid metabolism	ko00561	1 out of 96	ENSRNOG00000029096	0.476562529	1.55787037
Mineral absorption	ko04978	1 out of 96	ENSRNOG00000000017	0.481931692	1.533528646
Carbon metabolism	ko01200	2 out of 96	ENSRNOG00000001214;ENSRNOG00000020420	0.490805596	1.219202899
Amphetamine addiction	ko05031	1 out of 96	ENSRNOG00000038202	0.507970782	1.422403382
Long-term potentiation	ko04720	1 out of 96	ENSRNOG00000038202	0.513021012	1.402083333
Central carbon metabolism in cancer	ko05230	1 out of 96	ENSRNOG00000001214	0.522968084	1.363136574
MicroRNAs in cancer	ko05206	2 out of 96	ENSRNOG00000006122;ENSRNOG00000018524	0.526139717	1.14123062
Non-small cell lung cancer	ko05223	1 out of 96	ENSRNOG00000005342	0.527865952	1.34446347

Ubiquitin mediated proteolysis	ko04120	2 out of 96	ENSRNOG00000006335;ENSRNOG00000014029	0.535490696	1.121666667
Pertussis	ko05133	1 out of 96	ENSRNOG00000038202	0.537512879	1.308611111
Thyroid hormone synthesis	ko04918	1 out of 96	ENSRNOG00000016275	0.542262934	1.291392544
Melanoma	ko05218	1 out of 96	ENSRNOG00000022631	0.542262934	1.291392544
Necroptosis	ko04217	2 out of 96	ENSRNOG00000042353;ENSRNOG00000047957	0.550797317	1.090509259
Glioma	ko05214	1 out of 96	ENSRNOG00000038202	0.551618679	1.258279915
PI3K-Akt signaling pathway	ko04151	4 out of 96	ENSRNOG00000011300;ENSRNOG00000020369;ENSRNOG00000022631;ENSRNOG00000057557	0.56019733	1.014427218
Drug metabolism - other enzymes	ko00983	1 out of 96	ENSRNOG00000050277	0.574188249	1.18247992
Longevity regulating pathway	ko04211	1 out of 96	ENSRNOG00000001092	0.599791913	1.102762172
ECM-receptor interaction	ko04512	1 out of 96	ENSRNOG00000011300	0.603908486	1.090509259
PPAR signaling pathway	ko03320	1 out of 96	ENSRNOG00000029096	0.607983153	1.078525641
Toll-like receptor signaling pathway	ko04620	1 out of 96	ENSRNOG00000007437	0.612016334	1.066802536
RNA degradation	ko03018	1 out of 96	ENSRNOG00000001214	0.62387113	1.033114035
Circadian entrainment	ko04713	1 out of 96	ENSRNOG00000038202	0.627742511	1.022352431
Aldosterone synthesis and secretion	ko04925	1 out of 96	ENSRNOG00000038202	0.627742511	1.022352431
Endocrine resistance	ko01522	1 out of 96	ENSRNOG00000016695	0.64283763	0.981458333
IL-17 signaling pathway	ko04657	1 out of 96	ENSRNOG00000047957	0.646515766	0.971740924
Melanogenesis	ko04916	1 out of 96	ENSRNOG00000038202	0.657326758	0.943709936
AGE-RAGE signaling pathway in diabeti	ko04933	1 out of 96	ENSRNOG00000016695	0.657326758	0.943709936
Protein digestion and absorption	ko04974	1 out of 96	ENSRNOG00000016057	0.657326758	0.943709936
Phosphatidylinositol signaling system	ko04070	1 out of 96	ENSRNOG00000038202	0.664351638	0.925904088
Pancreatic secretion	ko04972	1 out of 96	ENSRNOG00000014347	0.674622589	0.900420489
Regulation of actin cytoskeleton	ko04810	2 out of 96	ENSRNOG00000018524;ENSRNOG00000022631	0.686618003	0.849747475
Inflammatory mediator regulation of TRP	ko04750	1 out of 96	ENSRNOG00000038202	0.691054081	0.860928363
Toxoplasmosis	ko05145	1 out of 96	ENSRNOG00000011300	0.70051597	0.838853276
C-type lectin receptor signaling pathway	ko04625	1 out of 96	ENSRNOG00000038202	0.712687097	0.81112259
Oocyte meiosis	ko04114	1 out of 96	ENSRNOG00000038202	0.718587788	0.797933604
Neurotrophin signaling pathway	ko04722	1 out of 96	ENSRNOG00000038202	0.72436853	0.785166667
Human immunodeficiency virus 1 infecti	ko05170	2 out of 96	ENSRNOG00000038202;ENSRNOG00000047957	0.727964225	0.785166667
Human cytomegalovirus infection	ko05163	2 out of 96	ENSRNOG00000038202;ENSRNOG00000047957	0.730008647	0.782038513
Cell cycle	ko04110	1 out of 96	ENSRNOG00000010760	0.732820004	0.766764323
Thyroid hormone signaling pathway	ko04919	1 out of 96	ENSRNOG00000001214	0.738311327	0.754967949
Amoebiasis	ko05146	1 out of 96	ENSRNOG00000011300	0.738311327	0.754967949
Relaxin signaling pathway	ko04926	1 out of 96	ENSRNOG00000016695	0.748961089	0.732431592
FoxO signaling pathway	ko04068	1 out of 96	ENSRNOG00000014205	0.754123981	0.721660539
Lysosome	ko04142	1 out of 96	ENSRNOG00000014997	0.759181777	0.711201691
Purine metabolism	ko00230	1 out of 96	ENSRNOG00000020420	0.761671927	0.706085132
Staphylococcus aureus infection	ko05150	1 out of 96	ENSRNOG00000047393	0.766576033	0.69606974
Cytokine-cytokine receptor interaction	ko04060	2 out of 96	ENSRNOG00000050123;ENSRNOG00000057557	0.770044956	0.721660539
NF-kappa B signaling pathway	ko04064	1 out of 96	ENSRNOG00000047957	0.773745544	0.681568287
Vascular smooth muscle contraction	ko04270	1 out of 96	ENSRNOG00000038202	0.778403706	0.672231735
HIF-1 signaling pathway	ko04066	1 out of 96	ENSRNOG00000001214	0.791816266	0.645696272
Breast cancer	ko05224	1 out of 96	ENSRNOG00000022631	0.800308372	0.629139957
Adrenergic signaling in cardiomyocytes	ko04261	1 out of 96	ENSRNOG00000038202	0.802377269	0.625132696
Oxytocin signaling pathway	ko04921	1 out of 96	ENSRNOG00000038202	0.802377269	0.625132696
Gastric cancer	ko05226	1 out of 96	ENSRNOG00000022631	0.802377269	0.625132696
Hippo signaling pathway	ko04390	1 out of 96	ENSRNOG00000050123	0.808457535	0.613411458
Alcoholism	ko05034	1 out of 96	ENSRNOG00000038202	0.812407819	0.605838477
JAK-STAT signaling pathway	ko04630	1 out of 96	ENSRNOG00000057557	0.816277465	0.598450203
mTOR signaling pathway	ko04150	1 out of 96	ENSRNOG00000011603	0.818182559	0.594823232
MAPK signaling pathway	ko04010	2 out of 96	ENSRNOG00000020369;ENSRNOG00000022631	0.829591798	0.633198925
cGMP-PKG signaling pathway	ko04022	1 out of 96	ENSRNOG00000038202	0.837890938	0.55764678
Non-alcoholic fatty liver disease	ko04932	1 out of 96	ENSRNOG00000020420	0.841239956	0.551381086
Hepatocellular carcinoma	ko05225	1 out of 96	ENSRNOG00000020369	0.850881637	0.533401268
NOD-like receptor signaling pathway	ko04621	1 out of 96	ENSRNOG00000047957	0.852431254	0.530518018
Protein processing in endoplasmic reticul	ko04141	1 out of 96	ENSRNOG00000000438	0.855482839	0.524844029
RNA transport	ko03013	1 out of 96	ENSRNOG00000023356	0.868460064	0.500744048

Focal adhesion	ko04510	1 out of 96	ENSRNOG00000011300	0.880283023	0.478760163
Viral carcinogenesis	ko05203	1 out of 96	ENSRNOG00000047957	0.880283023	0.478760163
Tuberculosis	ko05152	1 out of 96	ENSRNOG00000038202	0.891053398	0.458625389
cAMP signaling pathway	ko04024	1 out of 96	ENSRNOG00000038202	0.89876235	0.444098793
Kaposi sarcoma-associated herpesvirus ir	ko05167	1 out of 96	ENSRNOG00000038202	0.899818628	0.442098348
Transcriptional misregulation in cancer	ko05202	1 out of 96	ENSRNOG00000002163	0.921316707	0.400595238
Herpes simplex virus 1 infection	ko05168	2 out of 96	ENSRNOG00000005513;ENSRNOG00000047957	0.922785543	0.484670782
Calcium signaling pathway	ko04020	1 out of 96	ENSRNOG00000038202	0.926902483	0.389467593
Thermogenesis	ko04714	1 out of 96	ENSRNOG00000004784	0.934207793	0.374602417
Epstein-Barr virus infection	ko05169	1 out of 96	ENSRNOG00000047957	0.943236308	0.355600845
Human papillomavirus infection	ko05165	1 out of 96	ENSRNOG00000011300	0.974356999	0.279617759
Neuroactive ligand-receptor interaction	ko04080	1 out of 96	ENSRNOG00000057557	0.976198018	0.274150372
Olfactory transduction	ko04740	1 out of 96	ENSRNOG00000038202	0.999999784	0.070863418

#Kegg_pathway-red	ko_id	Cluster_fre	kegg	P-value	rich_factor
Melanoma	ko05218	7 out of 31	ENSRNOG00000008658;ENSRNOG00000012278;ENSRNOG00000012912;ENSRNOG00000013867;ENSRNOG00000020899;ENSRNOG00000048389;ENSRNOG00000061530	3.78E-09	27.99405772
Breast cancer	ko05224	6 out of 31	ENSRNOG00000012278;ENSRNOG00000012912;ENSRNOG00000013867;ENSRNOG00000020899;ENSRNOG00000048389;ENSRNOG000	9.78E-06	11.6898263
Gastric cancer	ko05226	6 out of 31	ENSRNOG00000012278;ENSRNOG00000012912;ENSRNOG00000013867;ENSRNOG00000020899;ENSRNOG00000048389;ENSRNOG000	1.01E-05	11.61536881
Ras signaling pathway	ko04014	7 out of 31	ENSRNOG00000006108;ENSRNOG00000012278;ENSRNOG00000012912;ENSRNOG00000013867;ENSRNOG00000020899;ENSRNOG00000048389;ENSRNOG00000061530	1.44E-05	8.376174752
Rap1 signaling pathway	ko04015	6 out of 31	ENSRNOG00000012278;ENSRNOG00000012912;ENSRNOG00000013867;ENSRNOG00000020899;ENSRNOG00000048389;ENSRNOG000	8.14E-05	8.033537019
Regulation of actin cytoskeleton	ko04810	6 out of 31	ENSRNOG00000012278;ENSRNOG00000012912;ENSRNOG00000013867;ENSRNOG00000020899;ENSRNOG00000048389;ENSRNOG000	8.96E-05	7.894428152
PI3K-Akt signaling pathway	ko04151	7 out of 31	ENSRNOG00000006108;ENSRNOG00000012278;ENSRNOG00000012912;ENSRNOG00000013867;ENSRNOG00000020899;ENSRNOG00000048389;ENSRNOG00000061530	0.000208756	5.497541052
Pathways in cancer	ko05200	8 out of 31	ENSRNOG00000006108;ENSRNOG00000008658;ENSRNOG00000012278;ENSRNOG00000012912;ENSRNOG00000013867;ENSRNOG00000020899;ENSRNOG00000048389;ENSRNOG00000061530	0.000384548	4.273258121
MAPK signaling pathway	ko04010	6 out of 31	ENSRNOG00000012278;ENSRNOG00000012912;ENSRNOG00000013867;ENSRNOG00000020899;ENSRNOG00000048389;ENSRNOG000	0.00044378	5.882622268
Serotonergic synapse	ko04726	4 out of 31	ENSRNOG00000004327;ENSRNOG00000006108;ENSRNOG00000007778;ENSRNOG00000019183	0.000747243	9.572771146
Phototransduction	ko04744	2 out of 31	ENSRNOG00000046962;ENSRNOG00000052051	0.003754811	21.70967742
Cholinergic synapse	ko04725	3 out of 31	ENSRNOG00000006108;ENSRNOG00000013829;ENSRNOG0000002	0.006511835	7.793217535
Purine metabolism	ko00230	3 out of 31	ENSRNOG00000009889;ENSRNOG00000012236;ENSRNOG0000004	0.010443384	6.559758645
Amino sugar and nucleotide sugar metabolism	ko00520	2 out of 31	ENSRNOG00000009889;ENSRNOG00000017605	0.011634099	12.15741935
Tryptophan metabolism	ko00380	2 out of 31	ENSRNOG00000001324;ENSRNOG00000004327	0.012542135	11.6898263
Ferroptosis	ko04216	2 out of 31	ENSRNOG00000013604;ENSRNOG00000019183	0.014448183	10.85483871
Arachidonic acid metabolism	ko00590	2 out of 31	ENSRNOG00000007778;ENSRNOG00000019183	0.033657223	6.907624633
Dopaminergic synapse	ko04728	2 out of 31	ENSRNOG00000004327;ENSRNOG00000006108	0.077054311	4.341935484
Phenylalanine metabolism	ko00360	1 out of 31	ENSRNOG00000004327	0.085249849	11.25686977
Galactose metabolism	ko00052	1 out of 31	ENSRNOG00000009889	0.097265472	9.804370447
Starch and sucrose metabolism	ko00500	1 out of 31	ENSRNOG00000009889	0.097265472	9.804370447
Folate biosynthesis	ko00790	1 out of 31	ENSRNOG00000000566	0.097265472	9.804370447

Alcoholism	ko05034	2 out of 31	ENSRNOG00000004327;ENSRNOG00000006108	0.098838119	3.752289924
Pentose phosphate pathway	ko00030	1 out of 31	ENSRNOG00000009889	0.100245428	9.497983871
Fructose and mannose metabolism	ko00051	1 out of 31	ENSRNOG00000017605	0.11792626	7.998302207
Linoleic acid metabolism	ko00591	1 out of 31	ENSRNOG00000019183	0.123744796	7.598387097
Tyrosine metabolism	ko00350	1 out of 31	ENSRNOG00000004327	0.129526181	7.23655914
Glycine, serine and threonine metabolism	ko00260	1 out of 31	ENSRNOG00000000925	0.140978413	6.607293128
Cocaine addiction	ko05030	1 out of 31	ENSRNOG00000004327	0.152284774	6.078709677
Cholesterol metabolism	ko04979	1 out of 31	ENSRNOG00000010549	0.163447061	5.628434886
Transcriptional misregulation in cancer	ko05202	2 out of 31	ENSRNOG00000003098;ENSRNOG00000008658	0.192315452	2.481105991
Amphetamine addiction	ko05031	1 out of 31	ENSRNOG00000004327	0.204052538	4.404862085
Glutathione metabolism	ko00480	1 out of 31	ENSRNOG00000013604	0.214554361	4.163499779
Adipocytokine signaling pathway	ko04920	1 out of 31	ENSRNOG00000004143	0.214554361	4.163499779
Inositol phosphate metabolism	ko00562	1 out of 31	ENSRNOG00000003033	0.230056084	3.847284606
Synaptic vesicle cycle	ko04721	1 out of 31	ENSRNOG00000019317	0.230056084	3.847284606
Mitophagy - animal	ko04137	1 out of 31	ENSRNOG00000008658	0.232610752	3.799193548
Longevity regulating pathway	ko04211	1 out of 31	ENSRNOG00000004143	0.255236631	3.415005437
GABAergic synapse	ko04727	1 out of 31	ENSRNOG00000006108	0.260176212	3.339950372
Morphine addiction	ko05032	1 out of 31	ENSRNOG00000006108	0.267526186	3.233356211
Progesterone-mediated oocyte maturation	ko04914	1 out of 31	ENSRNOG00000020737	0.269960436	3.199320883
Circadian entrainment	ko04713	1 out of 31	ENSRNOG00000006108	0.272386857	3.165994624
Glycolysis / Gluconeogenesis	ko00010	1 out of 31	ENSRNOG00000009889	0.286782316	2.979759646
Melanogenesis	ko04916	1 out of 31	ENSRNOG00000008658	0.291519261	2.922456576
AGE-RAGE signaling pathway in diabetic con	ko04933	1 out of 31	ENSRNOG00000003033	0.291519261	2.922456576
Phosphatidylinositol signaling system	ko04070	1 out of 31	ENSRNOG00000003033	0.296225752	2.867315886
Biosynthesis of amino acids	ko01230	1 out of 31	ENSRNOG00000000925	0.323835713	2.57572444
Glutamatergic synapse	ko04724	1 out of 31	ENSRNOG00000006108	0.330572676	2.511863503
Neuroactive ligand-receptor interaction	ko04080	2 out of 31	ENSRNOG00000010549;ENSRNOG00000013829	0.330671813	1.697963597
Cell cycle	ko04110	1 out of 31	ENSRNOG00000020737	0.346040606	2.374495968
Osteoclast differentiation	ko04380	1 out of 31	ENSRNOG00000008658	0.346040606	2.374495968
AMPK signaling pathway	ko04152	1 out of 31	ENSRNOG00000004143	0.348221878	2.356089022
Thyroid hormone signaling pathway	ko04919	1 out of 31	ENSRNOG00000003033	0.350396108	2.337965261
Relaxin signaling pathway	ko04926	1 out of 31	ENSRNOG00000006108	0.359023051	2.268175253
Apelin signaling pathway	ko04371	1 out of 31	ENSRNOG00000006108	0.398509625	1.986506431
Spinocerebellar ataxia	ko05017	1 out of 31	ENSRNOG00000009288	0.398509625	1.986506431
Oxytocin signaling pathway	ko04921	1 out of 31	ENSRNOG00000021056	0.40651733	1.935894802
Hippo signaling pathway	ko04390	1 out of 31	ENSRNOG00000013867	0.412455305	1.899596774
Carbon metabolism	ko01200	1 out of 31	ENSRNOG00000000925	0.414421823	1.887798036
Retrograde endocannabinoid signaling	ko04723	1 out of 31	ENSRNOG00000006108	0.433739902	1.77740049
MicroRNAs in cancer	ko05206	1 out of 31	ENSRNOG00000020737	0.435637434	1.767066767
Influenza A	ko05164	1 out of 31	ENSRNOG00000015329	0.439414054	1.746755654
Non-alcoholic fatty liver disease	ko04932	1 out of 31	ENSRNOG00000004143	0.446894058	1.707502718
Necroptosis	ko04217	1 out of 31	ENSRNOG00000019183	0.450597749	1.688530466
Cellular senescence	ko04218	1 out of 31	ENSRNOG00000020737	0.452440583	1.679201568
Chemokine signaling pathway	ko04062	1 out of 31	ENSRNOG00000006108	0.45975228	1.642894507
Kaposi sarcoma-associated herpesvirus infectic	ko05167	1 out of 31	ENSRNOG00000006108	0.523055091	1.369078756
Human immunodeficiency virus 1 infection	ko05170	1 out of 31	ENSRNOG00000006108	0.566125788	1.215741935
Human cytomegalovirus infection	ko05163	1 out of 31	ENSRNOG00000006108	0.567592218	1.210898342
Calcium signaling pathway	ko04020	1 out of 31	ENSRNOG00000003033	0.569053852	1.20609319
Human T-cell leukemia virus 1 infection	ko05166	1 out of 31	ENSRNOG00000010549	0.587625404	1.146926354
Olfactory transduction	ko04740	1 out of 31	ONT.66	0.992825781	0.219448003

#Kegg_pathway-blue	ko_id	Cluster_fre	GeneID	P-value	rich_factor
Ribosome	ko03010	23 out of 35	ENSRNOG00000000957;ENSRNOG00000002116;ENSRNOG00000011494;ENSRNOG00000013845;ENSRNOG00000019974;ENSRNOG00000023385;ENSRNOG00000024845;ENSRNOG00000029410;ENSRNOG00000031022;ENSRNOG00000031315;ENSRNOG00000032803;ENSRNOG00000033916;ENSRNOG00000034180;ENSRNOG00000037607;ENSRNOG00000038074;ENSRNOG00000048958;ENSRNOG00000049848;ENSRNOG00000049921;ENSRNOG00000060449;ONT.14316;O	1.21E-21	12.43293173
Oxidative phosphorylation	ko00190	8 out of 35	ENSRNOG00000011825;ENSRNOG00000026616;ENSRNOG00000027049;ENSRNOG00000030237;ENSRNOG00000048174;ENSRNOG00000049912;ONT.11638;ONT.7763	9.25E-08	13.46
Parkinson disease	ko05012	9 out of 35	ENSRNOG00000011825;ENSRNOG00000018294;ENSRNOG00000019974;ENSRNOG00000026616;ENSRNOG00000030237;ENSRNOG00000048174;ENSRNOG00000049912;ONT.11638;ONT.7763	7.43E-07	8.383391003
Thermogenesis	ko04714	8 out of 35	ENSRNOG00000011825;ENSRNOG00000026616;ENSRNOG00000027049;ENSRNOG00000030237;ENSRNOG00000048174;ENSRNOG00000049912;ONT.11638;ONT.7763	3.94E-06	8.219847328
Prion disease	ko05020	8 out of 35	ENSRNOG00000011825;ENSRNOG00000018294;ENSRNOG00000026616;ENSRNOG00000030237;ENSRNOG00000048174;ENSRNOG00000049912;ONT.11638;ONT.7763	1.30E-05	6.992207792
Non-alcoholic fatty liver disease	ko04932	6 out of 35	ENSRNOG00000011825;ENSRNOG00000026616;ENSRNOG00000030237;ENSRNOG00000048174;ONT.11638;ONT.7763	4.30E-05	9.074157303
Amyotrophic lateral sclerosis	ko05014	8 out of 35	ENSRNOG00000011825;ENSRNOG00000018294;ENSRNOG00000026616;ENSRNOG00000030237;ENSRNOG00000048174;ENSRNOG00000049912;ONT.11638;ONT.7763	9.36E-05	5.304433498
Huntington disease	ko05016	7 out of 35	ENSRNOG00000011825;ENSRNOG00000026616;ENSRNOG00000030237;ENSRNOG00000048174;ENSRNOG00000049912;ONT.11638;O	0.000222077	5.493877551
Alzheimer disease	ko05010	7 out of 35	ENSRNOG00000011825;ENSRNOG00000026616;ENSRNOG00000030237;ENSRNOG00000048174;ENSRNOG00000049912;ONT.11638;O	0.000833582	4.413114754
Retrograde endocannabinoid signaling	ko04723	4 out of 35	ENSRNOG00000011825;ENSRNOG00000026616;ONT.11638;ONT.77	0.00353623	6.297076023
Thyroid hormone synthesis	ko04918	2 out of 35	ENSRNOG00000006228;ENSRNOG00000018294	0.032181634	7.084210526
Cardiac muscle contraction	ko04260	2 out of 35	ENSRNOG00000030237;ENSRNOG00000048174	0.051961546	5.438383838
Protein export	ko03060	1 out of 35	ENSRNOG00000018294	0.102451546	9.282758621
Protein processing in endoplasmic reticulum	ko04141	2 out of 35	ENSRNOG00000006228;ENSRNOG00000018294	0.152652614	2.879144385
Mitophagy - animal	ko04137	1 out of 35	ENSRNOG00000019974	0.258431507	3.365
Antigen processing and presentation	ko04612	1 out of 35	ENSRNOG00000018294	0.332515965	2.492592593
Spliceosome	ko03040	1 out of 35	ENSRNOG00000031127	0.447322583	1.703797468
Ubiquitin mediated proteolysis	ko04120	1 out of 35	ENSRNOG00000019974	0.481795471	1.538285714

Kaposi sarcoma-associated herpesvirus infection	ko05167	1 out of 35	ENSRNOG00000019974	0.566586908	1.212612613
Salmonella infection	ko05132	1 out of 35	ENSRNOG00000012163	0.626570086	1.031417625
