# Supplementary material for: Effects of Maternal High-Fructose Diet on Long Non-Coding RNAs and Anxiety-like Behaviors in Offspring
Source: Int J Mol Sci. 2023 Feb 24;24(5):4460. doi: 10.3390/ijms24054460 (PMC10003385; doi:10.3390/ijms24054460)
Supplement: Supplementary file 1 [file ijms-24-04460-s001.zip › Table S8.pdf]

Table S8: The full KEGG pathway enrichment results of DElncRNA target genes in Con/F13% and Con/F40%.

Con vs F40%

#Kegg_pathway	ko_id	Cluster_fre	GeneID	P-value	rich_factor
Adrenergic signaling in cardiomyocyt	ko04261	5 out of 22	ENSRNOG00000024411	2.52E-05	13.63925883
Cardiac muscle contraction	ko04260	4 out of 22	ENSRNOG00000006984;ENSRNOG00000031233;ENSRNOG00000057848;ENSRNOG00000057852;ONT.972	7.25E-05	17.30394858
Osteoclast differentiation	ko04380	4 out of 22	ENSRNOG00000006984;ENSRNOG00000031233	0.000196649	13.38352273
MAPK signaling pathway	ko04010	5 out of 22	ENSRNOG00000006984;ENSRNOG00000031233	0.000619691	6.907624633
Arrhythmogenic right ventricular carc	ko05412	3 out of 22	ENSRNOG00000024497	0.000868378	15.66851441
Hypertrophic cardiomyopathy	ko05410	3 out of 22	ENSRNOG00000006984;ENSRNOG00000031233	0.001371677	13.38352273
Leukocyte transendothelial migration	ko04670	3 out of 22	ENSRNOG00000006984;ENSRNOG00000024309;ENSRNOG00000031233;ENSRNOG00000031317	0.002190746	11.37007241
Thermogenesis	ko04714	4 out of 22	ENSRNOG00000006984;ENSRNOG00000031233	0.002878765	6.53851492
Dilated cardiomyopathy	ko05414	3 out of 22	ENSRNOG00000006984;ENSRNOG00000020929;ENSRNOG0000003	0.00298639	10.1969697
FoxO signaling pathway	ko04068	3 out of 22	ENSRNOG00000006984;ENSRNOG00000031233	0.00370564	9.447192513
Prion disease	ko05020	4 out of 22	ENSRNOG00000024309;ENSRNOG00000031317	0.005140028	5.561983471
Oxytocin signaling pathway	ko04921	3 out of 22	ENSRNOG00000006984;ENSRNOG00000031233	0.005539545	8.183555298
Cellular senescence	ko04218	3 out of 22	ENSRNOG00000006984;ENSRNOG00000031233;ENSRNOG0000004	0.00820724	7.098442993
VEGF signaling pathway	ko04370	2 out of 22	ENSRNOG00000057848;ENSRNOG00000057852;ONT.972	0.008763228	14.04172876
RIG-I-like receptor signaling pathway	ko04622	2 out of 22	ENSRNOG00000006984;ENSRNOG00000031233	0.009325934	13.5959596
Pertussis	ko05133	2 out of 22	ENSRNOG00000024309;ENSRNOG00000031317	0.013029296	11.42060606
Amyotrophic lateral sclerosis	ko05014	4 out of 22	ENSRNOG00000006984;ENSRNOG00000031233	0.013408614	4.219435737
Prolactin signaling pathway	ko04917	2 out of 22	ENSRNOG00000031915	0.014384928	10.84234753
Alzheimer disease	ko05010	4 out of 22	ENSRNOG00000024411	0.015894856	4.011922504
Toll-like receptor signaling pathway	ko04620	2 out of 22	ONT.5723	0.019190487	9.31027668

Term	Count	GeneID	Pvalue	group
response to drug	1	ENSRNOG00000018681	3.10E-08	BP
intracellular signal transduction	7	ENSRNOG00000006984;ENSRNOG00000020845;ENSRNOG00000020849;ENSRNOG00000020873;ENSRNOG00000025111;ENSRNOG00000027894;ENSRNOG00000031233	1.60E-06	BP
neuron migration	1	ENSRNOG00000047891	1.70E-06	BP
positive regulation of GTPase activity	1	ENSRNOG00000027894	3.20E-06	BP
protein ubiquitination	1	ENSRNOG00000012168	3.80E-06	BP
angiogenesis	1	ENSRNOG00000012216	9.20E-06	BP
protein transport	1	ENSRNOG00000006984	9.90E-06	BP
brain development	4	ENSRNOG00000018681;ENSRNOG00000018798;ENSRNOG00000020851;ENSRNOG00000047891	1.00E-05	BP
negative regulation of cell proliferatic	1	ENSRNOG00000006984	1.60E-05	BP
regulation of growth	2	ENSRNOG00000006984;ENSRNOG00000022101	2.20E-05	BP
negative regulation of neuron differen	1	ENSRNOG00000047891	2.20E-05	BP
response to light stimulus	1	ENSRNOG00000012168	2.30E-05	BP
hippocampus development	1	ENSRNOG00000018798	2.50E-05	BP
aging	1	ENSRNOG00000047891	3.80E-05	BP
negative regulation of transcription fr	2	ENSRNOG00000031915;ENSRNOG00000047891	4.60E-05	BP
glomerulus development	1	ENSRNOG00000020873	4.70E-05	BP
single-organism organelle organizatio	6	ENSRNOG00000006028;ENSRNOG00000018681;ENSRNOG00000020873;ENSRNOG00000031093;ENSRNOG00000031915;ENSRNOG000	6.50E-05	BP
memory	1	ENSRNOG00000055525	7.30E-05	BP
cytoplasmic microtubule organization	1	ENSRNOG00000006028	1.10E-04	BP
peptidyl-serine phosphorylation	1	ENSRNOG00000031233	0.00012	BP

Fc epsilon RI signaling pathway	ko04664	2 out of 22	ENSRNOG00000011424	0.020383558	9.016267943
Progesterone-mediated oocyte matura	ko04914	2 out of 22	ENSRNOG00000006984;ENSRNOG00000024309;ENSRNOG00000031233;ENSRNOG00000031317	0.020383558	9.016267943
GnRH signaling pathway	ko04912	2 out of 22	ENSRNOG00000006984;ENSRNOG00000031233	0.021195955	8.830365511
Endocrine resistance	ko01522	2 out of 22	ENSRNOG00000006984;ENSRNOG00000031233;ENSRNOG00000057848;ENSRNOG00000057852;ONT	0.022439748	8.565454545
IL-17 signaling pathway	ko04657	2 out of 22	ENSRNOG00000006984;ENSRNOG00000031233	0.022860998	8.480648065
Leishmaniasis	ko05140	2 out of 22	ENSRNOG00000006984;ENSRNOG00000011424;ENSRNOG0000003	0.023285549	8.397504456
AGE-RAGE signaling pathway in dia	ko04933	2 out of 22	ENSRNOG00000024309;ENSRNOG00000031317	0.024144484	8.236013986
PD-L1 expression and PD-1 checkpoi	ko05235	2 out of 22	ENSRNOG00000020941;ENSRNOG00000021202;ENSRNOG00000024309;ENSRNOG00000031317	0.025016424	8.080617496
Th1 and Th2 cell differentiation	ko04658	2 out of 22	ENSRNOG00000055519	0.02590124	7.930976431
TNF signaling pathway	ko04668	2 out of 22	ENSRNOG00000006984;ENSRNOG00000031233	0.028168781	7.58004827
Inflammatory mediator regulation of	ko04750	2 out of 22	ENSRNOG00000006984;ENSRNOG00000031233	0.028631678	7.513556619
Toxoplasmosis	ko05145	2 out of 22	ENSRNOG00000006984;ENSRNOG00000031233	0.030038865	7.320901321
C-type lectin receptor signaling pathw	ko04625	2 out of 22	ENSRNOG00000006984;ENSRNOG00000031233	0.031957694	7.078888054
Growth hormone synthesis, secretion	ko04935	2 out of 22	ENSRNOG00000006984;ENSRNOG00000031233	0.032935081	6.963784183
Oocyte meiosis	ko04114	2 out of 22	ENSRNOG00000057848;ENSRNOG00000057852;ONT.972	0.032935081	6.963784183

nucleus	14	ENSRNOG00000006984;ENSRNOG00000012168;ENSRNOG00000019201;ENSRNOG00000020922;ENSRNOG00000022101;ENSRNOG00000025111;ENSRNOG00000031233;ENSRNOG00000031915;ENSRNOG00000042465;ENSRNOG00000047891;ENSRNOG00000055519;ENSRNOG00000055525;ONT.11508;O	5.20E-22	CC
nucleoplasm	5	ENSRNOG00000019201;ENSRNOG00000022101;ENSRNOG00000031233;ENSRNOG00000031915;ENSRNOG00000055525	3.00E-20	CC
perinuclear region of cytoplasm	1	ENSRNOG00000020851	1.90E-14	CC
Golgi membrane	1	ENSRNOG00000021202	1.90E-13	CC
extracellular vesicular exosome	7	ENSRNOG00000006028;ENSRNOG00000012216;ENSRNOG00000019201;ENSRNOG00000020873;ENSRNOG00000021202;ENSRNOG00000022101;ENSRNOG00000055716	1.00E-12	CC
ATP binding	3	ENSRNOG00000019201;ENSRNOG00000031233;ENSRNOG0000003	7.70E-19	MF
zinc ion binding	2	ENSRNOG00000020941;ENSRNOG00000021202	9.20E-14	MF
protein homodimerization activity	2	ENSRNOG00000019201;ENSRNOG00000020922	1.20E-13	MF
identical protein binding	5	ENSRNOG00000012189;ENSRNOG00000019201;ENSRNOG00000020845;ENSRNOG00000020851;ENSRNOG00000020922	9.20E-12	MF
metal ion binding	7	ENSRNOG00000018798;ENSRNOG00000019201;ENSRNOG00000020851;ENSRNOG00000031233;ENSRNOG00000042465;ENSRNOG00000055519;ENSRNOG00000055525	1.20E-11	MF

Chagas disease	ko05142	2 out of 22	ENSRNOG00000006984;ENSRNOG00000031233	0.033428215	6.907624633
Neurotrophin signaling pathway	ko04722	2 out of 22	ENSRNOG00000027894	0.033924288	6.852363636
T cell receptor signaling pathway	ko04660	2 out of 22	ENSRNOG00000006984;ENSRNOG00000031233	0.034423287	6.797979798
Platelet activation	ko04611	2 out of 22	ENSRNOG00000006984;ENSRNOG00000024309;ENSRNOG00000031233;ENSRNOG00000031317	0.035429998	6.691761364
Th17 cell differentiation	ko04659	2 out of 22	ENSRNOG00000006984;ENSRNOG00000031233	0.035937681	6.639887245
Natural killer cell mediated cytotoxici	ko04650	2 out of 22	ENSRNOG00000006984;ENSRNOG00000031233	0.037996916	6.440191388
Relaxin signaling pathway	ko04926	2 out of 22	ENSRNOG00000006984;ENSRNOG00000031233	0.038518778	6.392130258
Sphingolipid signaling pathway	ko04071	2 out of 22	ENSRNOG00000006984;ENSRNOG00000031233	0.038518778	6.392130258
Fatty acid biosynthesis	ko00061	1 out of 22	ENSRNOG00000006984;ENSRNOG00000031233	0.041241912	23.79292929
Dopaminergic synapse	ko04728	2 out of 22	ENSRNOG00000024309;ENSRNOG00000057848;ENSRNOG00000057852;ONT.972	0.041708059	6.118181818
Signaling pathways regulating pluripc	ko04550	2 out of 22	ENSRNOG00000011424	0.044440313	5.907210031
Fluid shear stress and atherosclerosis	ko05418	2 out of 22	ENSRNOG00000011424	0.050680614	5.490675991
Oxidative phosphorylation	ko00190	2 out of 22	ENSRNOG00000024309	0.053025232	5.353409091
Hepatitis B	ko05161	2 out of 22	ENSRNOG00000006984;ENSRNOG00000031233	0.056614732	5.159912377
Retrograde endocannabinoid signalin	ko04723	2 out of 22	ENSRNOG00000006984;ENSRNOG00000031233	0.059670686	5.009037746
Yersinia infection	ko05135	2 out of 22	ENSRNOG00000006984;ENSRNOG00000031233	0.059670686	5.009037746
NOD-like receptor signaling pathway	ko04621	2 out of 22	ENSRNOG00000006984;ENSRNOG00000031233	0.068525944	4.62997543
Proteoglycans in cancer	ko05205	2 out of 22	ENSRNOG00000006984;ENSRNOG00000031233	0.088145321	4.002548853
Tuberculosis	ko05152	2 out of 22	ENSRNOG00000057848;ENSRNOG00000057852;ONT.972	0.088145321	4.002548853
Kaposi sarcoma-associated herpesvir	ko05167	2 out of 22	ENSRNOG00000006984;ENSRNOG00000031233	0.09383083	3.858312858
Rap1 signaling pathway	ko04015	2 out of 22	ENSRNOG00000006984;ENSRNOG00000031233	0.097439161	3.773327994
Human immunodeficiency virus 1 inf	ko05170	2 out of 22	ENSRNOG00000006984;ENSRNOG00000031233	0.11454018	3.426181818
Human cytomegalovirus infection	ko05163	2 out of 22	ENSRNOG00000020845;ENSRNOG00000020849	0.115301241	3.412531691
Salmonella infection	ko05132	2 out of 22	ENSRNOG00000006984;ENSRNOG00000031233	0.122986028	3.281783351
Notch signaling pathway	ko04330	1 out of 22	ENSRNOG00000006984;ENSRNOG00000031233	0.127155939	7.384012539
Epstein-Barr virus infection	ko05169	2 out of 22	ENSRNOG00000020941	0.134750583	3.10342556

Fatty acid metabolism	ko01212	1 out of 22	ENSRNOG00000006984;ENSRNOG00000020845;ENSRNOG00000031233;ENSRNOG00000055716	0.143433146	6.488980716
Parkinson disease	ko05012	2 out of 22	ENSRNOG00000006984;ENSRNOG00000031233	0.145155694	2.963825102
Lysine degradation	ko00310	1 out of 22	ENSRNOG00000006984;ENSRNOG00000031233	0.149462068	6.20685112
Huntington disease	ko05016	2 out of 22	ENSRNOG00000006984;ENSRNOG00000031233	0.190036072	2.497217069
Spliceosome	ko03040	1 out of 22	ENSRNOG00000006984;ENSRNOG00000031233	0.310970295	2.710586881
Alcoholism	ko05034	1 out of 22	ENSRNOG00000055525	0.317493257	2.64365881
Hepatitis C	ko05160	1 out of 22	ENSRNOG00000006984;ENSRNOG00000031233	0.325564091	2.564507349
MicroRNAs in cancer	ko05206	1 out of 22	ENSRNOG00000006984;ENSRNOG00000031233	0.333543774	2.489957717
Tight junction	ko04530	1 out of 22	ENSRNOG00000031915	0.333543774	2.489957717
Non-alcoholic fatty liver disease	ko04932	1 out of 22	ENSRNOG00000006984;ENSRNOG00000031233	0.343000456	2.406026558
Cell adhesion molecules	ko04514	1 out of 22	ENSRNOG00000006984;ENSRNOG00000031233	0.350783046	2.340288127
Viral carcinogenesis	ko05203	1 out of 22	ENSRNOG00000057848;ENSRNOG00000057852;ONT.972	0.383993149	2.089135255
Regulation of actin cytoskeleton	ko04810	1 out of 22	ENSRNOG00000006984;ENSRNOG00000031233	0.421151872	1.85399449
Herpes simplex virus 1 infection	ko05168	1 out of 22	ENSRNOG00000006984;ENSRNOG00000031233	0.620039588	1.057463524

Con vs F13%

#Kegg_pathway	ko_id	Cluster_frequency	GeneID	P-value	rich_factor
Oxytocin signaling pathway	ko04921	4 out of 26 15.3846153	ENSRNOG00000021056;ENSRNOG00000057848;ENSRNOG00000057852;ONT.972	0.000833409	9.232729054
Arrhythmogenic right ventricular carc	ko05412	3 out of 26 11.5384615	ENSRNOG00000057848;ENSRNOG00000057852;ONT.972	0.001429805	13.25797373
Hypertrophic cardiomyopathy	ko05410	3 out of 26 11.5384615	ENSRNOG00000057848;ENSRNOG00000057852;ONT.972	0.002248561	11.32451923
Cardiac muscle contraction	ko04260	3 out of 26 11.5384615	ENSRNOG00000057848;ENSRNOG00000057852;ONT.972	0.002454912	10.98135198
Dilated cardiomyopathy	ko05414	3 out of 26 11.5384615	ENSRNOG00000057848;ENSRNOG00000057852;ONT.972	0.004849641	8.628205128
Adrenergic signaling in cardiomyocyt	ko04261	3 out of 26 11.5384615	ENSRNOG00000057848;ENSRNOG00000057852;ONT.972	0.008909078	6.924546791
MAPK signaling pathway	ko04010	4 out of 26 15.3846153	ENSRNOG00000016203;ENSRNOG00000057848;ENSRNOG00000057852;ONT.972	0.009695871	4.675930521
Longevity regulating pathway	ko04211	2 out of 26 7.69230769	ENSRNOG00000014287;ENSRNOG00000051592	0.02475508	8.143474503
Ubiquinone and other terpenoid-quinc	ko00130	1 out of 26 3.84615384	ENSRNOG00000050828	0.035307618	27.87573964
Glycosphingolipid biosynthesis - glob	ko00603	1 out of 26 3.84615384	ENSRNOG00000011513	0.040632026	24.15897436
AMPK signaling pathway	ko04152	2 out of 26 7.69230769	ENSRNOG00000014287;ENSRNOG00000051592	0.04880931	5.61836613

Term	Count	GeneID	Pvalue	group
positive reg	3	ENSRNOG00000011879;ENSRNOG00000025730;ENSRNOG00000051592	4.80E-16	BP
response to	1	ENSRNOG00000018681	3.10E-08	BP
intracellulaı	4	ENSRNOG00000000503;ENSRNOG00000011879;ENSRNOG00000027894;ENSRNOG00000051592	1.00E-06	BP
protein ubic	2	ENSRNOG00000012734;ENSRNOG00000051592	1.70E-06	BP
positive reg	2	ENSRNOG00000006467;ENSRNOG00000027894	3.30E-06	BP
nucleus	15	ENSRNOG00000001781;ENSRNOG00000003132;ENSRNOG00000003991;ENSRNOG00000004001;ENSRNOG00000005618;ENSRNOG00000006467;ENSRNOG00000011661;ENSRNOG00000011879;ENSRNOG00000014625;ENSRNOG00000014801;ENSRNOG00000015434;ENSRNOG00000015999;ENSRNOG00000016411;ENSRNOG00000018681;ENSRNOG00000019201;ENSRNOG00000020658;ENSRNOG00000022101;ENSRNOG00000025730;ENSRNOG00000027894;ENSRNOG00000038166;ENSRNOG00000042961;ENSRNOG00000049104;ENSRNOG00000050828;ENSRNOG0000005	4.50E-22	CC
nucleoplasn	8	ENSRNOG00000011661;ENSRNOG00000011879;ENSRNOG00000015999;ENSRNOG00000019201;ENSRNOG00000022101;ENSRNOG00000022502;ENSRNOG00000030572;ENSRNOG00000051592	1.60E-18	CC
neuronal ce	1	ENSRNOG00000021056	2E-14	CC
extracellula	5	ENSRNOG00000019201;ENSRNOG00000019840;ENSRNOG00000022101;ENSRNOG00000038166;ENSRNOG00000055716	1.1E-12	CC
axon	3	ENSRNOG00000006467;ENSRNOG00000049104;ENSRNOG0000005	1.1E-10	CC
cell junctioı	5	ENSRNOG00000001781;ENSRNOG00000003132;ENSRNOG00000027894;ENSRNOG00000030572;ENSRNOG00000057848	5.80E-10	CC

FoxO signaling pathway	ko04068	2 out of 26	7.69230769	ENSRNOG00000014287;ENSRNOG00000051592	0.053641794	5.32918552	mitochondr	8	ENSRNOG00000004001;ENSRNOG00000005618;ENSRNOG00000014625;ENSRNOG00000014801;ENSRNOG00000019201;ENSRNOG00000025730;ENSRNOG00000038166;ENSRNOG00000051592	2.30E-09	CC
Terpenoid backbone biosynthesis	ko00900	1 out of 26	3.84615384	ENSRNOG00000016767	0.074558177	12.94230769	endoplasmi	4	ENSRNOG00000003132;ENSRNOG00000022101;ENSRNOG00000042961;ENSRNOG00000050828	2.30E-08	CC
MicroRNAs in cancer	ko05206	2 out of 26	7.69230769	ENSRNOG00000016203;ENSRNOG00000051592	0.080938717	4.213774597	endoplasmi	1	ENSRNOG00000050828	5.80E-08	CC
Galactose metabolism	ko00052	1 out of 26	3.84615384	ENSRNOG00000011513	0.082221845	11.6898263	intracellular	33	ENSRNOG00000000503;ENSRNOG00000003991;ENSRNOG00000011661;ENSRNOG00000011879;ENSRNOG00000015434;ENSRNOG00000015999;ENSRNOG00000019201;ENSRNOG00000020658;ENSRNOG00000022101;ENSRNOG00000022502;ENSRNOG00000030572;ENSRNOG00000049104;ENSRNOG00000051592;ENSRNOG00000055519;	8.30E-08	CC
Starch and sucrose metabolism	ko00500	1 out of 26	3.84615384	ENSRNOG00000016214	0.082221845	11.6898263	postsynapti	1	ENSRNOG00000057848	5.30E-07	CC
RNA polymerase	ko03020	1 out of 26	3.84615384	ENSRNOG00000013545	0.087297016	10.98135198	centrosome	1	ENSRNOG00000049104	5.80E-07	CC
Nicotinate and nicotinamide metaboli	ko00760	1 out of 26	3.84615384	ENSRNOG00000051592	0.099867159	9.536437247	cytosol	5	ENSRNOG00000015434;ENSRNOG00000016411;ENSRNOG00000019201;ENSRNOG00000022101;ENSRNOG00000051592	0.0000012	CC
Homologous recombination	ko03440	1 out of 26	3.84615384	ENSRNOG00000059997	0.117186029	8.052991453	protein com	12	ENSRNOG00000004001;ENSRNOG00000005378;ENSRNOG00000006467;ENSRNOG00000012734;ENSRNOG00000014625;ENSRNOG00000014801;ENSRNOG00000018681;ENSRNOG00000030572;ENSRNOG00000042961;ENSRNOG00000049104;ENSRNOG00000051592;ENS	0.0000024	CC
Sphingolipid metabolism	ko00600	1 out of 26	3.84615384	ENSRNOG00000011513	0.138982799	6.710826211	proteinaceo	2	ENSRNOG00000018798;ENSRNOG00000018870	0.0000024	CC
Fanconi anemia pathway	ko03460	1 out of 26	3.84615384	ENSRNOG00000059997	0.141372471	6.588811189	nuclear mer	2	ENSRNOG00000051592;ENSRNOG00000059997	3.20E-06	CC
Ferroptosis	ko04216	1 out of 26	3.84615384	ENSRNOG00000013604	0.143755765	6.471153846	plasma mer	8	ENSRNOG00000003132;ENSRNOG00000005378;ENSRNOG00000016371;ENSRNOG00000021056;ENSRNOG00000027894;ENSRNOG00000049104;ENSRNOG00000055716;ENSRNOG00000057848	5.10E-06	CC
Cytosolic DNA-sensing pathway	ko04623	1 out of 26	3.84615384	ENSRNOG00000013545	0.143755765	6.471153846	dendrite	3	ENSRNOG00000018798;ENSRNOG00000021056;ENSRNOG0000004	7.60E-06	CC
Ribosome	ko03010	3 out of 26	11.5384615	ENSRNOG00000011494;ENSRNOG00000014214;ENSRNOG0000001	0.155890384	2.183039852	postsynapti	1	ENSRNOG00000057848	8.80E-06	CC
Glycerolipid metabolism	ko00561	1 out of 26	3.84615384	ENSRNOG00000011513	0.16026161	5.752136752	apical plasn	1	ENSRNOG00000003132	1.20E-05	CC
Longevity regulating pathway - multi	ko04213	1 out of 26	3.84615384	ENSRNOG00000051592	0.164921089	5.575147929	ATP bindin	4	ENSRNOG00000006467;ENSRNOG00000020658;ENSRNOG00000030572;ENSRNOG00000059997	6.70E-19	MF

Cytokine-cytokine receptor interactio	ko04060	2 out of 26	7.69230769	ENSRNOG00000020630;ENSRNOG00000059640	0.172089444	2.66459276
Amphetamine addiction	ko05031	1 out of 26	3.84615384	ENSRNOG00000051592	0.174165581	5.251950948
Glutathione metabolism	ko00480	1 out of 26	3.84615384	ENSRNOG00000013604	0.183311627	4.964172813
Adipocytokine signaling pathway	ko04920	1 out of 26	3.84615384	ENSRNOG00000014287	0.183311627	4.964172813
Acute myeloid leukemia	ko05221	1 out of 26	3.84615384	ENSRNOG00000000503	0.183311627	4.964172813
Viral protein interaction with cytokin	ko04061	1 out of 26	3.84615384	ENSRNOG00000059640	0.210169122	4.263348416
Intestinal immune network for IgA pr	ko04672	1 out of 26	3.84615384	ENSRNOG00000059640	0.221107273	4.026495726
PPAR signaling pathway	ko03320	1 out of 26	3.84615384	ENSRNOG00000000503	0.223277356	3.982248521
Huntington disease	ko05016	2 out of 26	7.69230769	ENSRNOG00000013545;ENSRNOG00000014625	0.244113229	2.113029827
Glucagon signaling pathway	ko04922	1 out of 26	3.84615384	ENSRNOG00000051592	0.26135897	3.324629499
Cholinergic synapse	ko04725	1 out of 26	3.84615384	ENSRNOG00000021056	0.277701835	3.097304405
Hematopoietic cell lineage	ko04640	1 out of 26	3.84615384	ENSRNOG00000020630	0.283740309	3.019871795
PI3K-Akt signaling pathway	ko04151	2 out of 26	7.69230769	ENSRNOG00000014287;ENSRNOG00000016203	0.289702836	1.87278871
Chagas disease	ko05142	1 out of 26	3.84615384	ENSRNOG00000005378	0.291716141	2.922456576
Osteoclast differentiation	ko04380	1 out of 26	3.84615384	ENSRNOG00000055716	0.299606538	2.831129808
Amoebiasis	ko05146	1 out of 26	3.84615384	ENSRNOG00000005378	0.303519974	2.787573964
Herpes simplex virus 1 infection	ko05168	2 out of 26	7.69230769	ENSRNOG00000006467;ENSRNOG00000055519	0.308348805	1.789553656
Relaxin signaling pathway	ko04926	1 out of 26	3.84615384	ENSRNOG00000005378	0.311283865	2.704362801
Lysosome	ko04142	1 out of 26	3.84615384	ENSRNOG00000011513	0.318964501	2.625975474
Oxidative phosphorylation	ko00190	1 out of 26	3.84615384	ENSRNOG00000014625	0.359757738	2.264903846
Autophagy - animal	ko04140	1 out of 26	3.84615384	ENSRNOG00000014287	0.365135	2.223218499
JAK-STAT signaling pathway	ko04630	1 out of 26	3.84615384	ENSRNOG00000020630	0.366917751	2.209662289
mTOR signaling pathway	ko04150	1 out of 26	3.84615384	ENSRNOG00000014287	0.368695688	2.196270396
Wnt signaling pathway	ko04310	1 out of 26	3.84615384	ENSRNOG00000000503	0.379262909	2.119208277
Tight junction	ko04530	1 out of 26	3.84615384	ENSRNOG00000014287	0.381007495	2.106887299
Cellular senescence	ko04218	1 out of 26	3.84615384	ENSRNOG00000051592	0.396498051	2.002124947
Chemokine signaling pathway	ko04062	1 out of 26	3.84615384	ENSRNOG00000059640	0.403262462	1.958835759
Axon guidance	ko04360	1 out of 26	3.84615384	ENSRNOG00000016203	0.411615499	1.907287449
RNA transport	ko03013	1 out of 26	3.84615384	ENSRNOG00000006467	0.421490788	1.848901099
Rap1 signaling pathway	ko04015	1 out of 26	3.84615384	ENSRNOG00000016203	0.470026723	1.596407997
Regulation of actin cytoskeleton	ko04810	1 out of 26	3.84615384	ENSRNOG00000027894	0.475996577	1.568764569
Calcium signaling pathway	ko04020	1 out of 26	3.84615384	ENSRNOG00000005378	0.506292529	1.438034188
Ras signaling pathway	ko04014	1 out of 26	3.84615384	ENSRNOG00000016203	0.509088362	1.426711084
Thermogenesis	ko04714	1 out of 26	3.84615384	ENSRNOG00000014625	0.520120219	1.383147387
Parkinson disease	ko05012	1 out of 26	3.84615384	ENSRNOG00000014625	0.55562011	1.253926005
Prion disease	ko05020	1 out of 26	3.84615384	ENSRNOG00000014625	0.579072935	1.176573427
Amyotrophic lateral sclerosis	ko05014	1 out of 26	3.84615384	ENSRNOG00000014625	0.682338158	0.892572944
Alzheimer disease	ko05010	1 out of 26	3.84615384	ENSRNOG00000014625	0.701050857	0.848675914
Pathways in cancer	ko05200	1 out of 26	3.84615384	ENSRNOG00000000503	0.802454631	0.636879816

zinc ion bin	2	ENSRNOG00000000503;ENSRNOG00000038166	1.40E-13	MF
protein hom	1	ENSRNOG00000019201	1.70E-13	MF
identical pr	2	ENSRNOG00000019201;ENSRNOG00000051592	4.70E-12	MF
metal ion bi	14	ENSRNOG00000000503;ENSRNOG00000001781;ENSRNOG00000004001;ENSRNOG00000005378;ENSRNOG00000014801;ENSRNOG00000018798;ENSRNOG00000019201;ENSRNOG00000019840;ENSRNOG00000020658;ENSRNOG00000038166;ENSRNOG00000049104;ENSRNOG00000051592;ENSRNOG00000055519;ENSRNOG00000059997	5.80E-12	MF
