# Supplementary material for: Effects of Maternal High-Fructose Diet on Long Non-Coding RNAs and Anxiety-like Behaviors in Offspring
Source: Int J Mol Sci. 2023 Feb 24;24(5):4460. doi: 10.3390/ijms24054460 (PMC10003385; doi:10.3390/ijms24054460)
Supplement: Supplementary file 1 [file ijms-24-04460-s001.zip › Table S9.pdf]

Table S9: Full-length non-chimeric sequence data statistics results.

Sample ID	Number of clean reads (except rRNA)	Number of full-length reads	Full-length percentage
Con1	2,748,210	2348008	85.44%
Con2	2,293,820	1909878	83.26%
Con3	2,782,864	2409906	86.60%
Con4	2,849,151	2429253	85.26%
Con5	2,979,383	2587449	86.85%
Con6	4,489,867	3965535	88.32%
Con7	2,563,717	2236522	87.24%
Con8	2,688,024	2355555	87.63%
F13-1	2,584,376	2244115	86.83%
F13-2	2,420,978	2017438	83.33%
F13-3	1,989,869	1608858	80.85%
F13-4	2,821,820	2457464	87.09%
F13-5	2,984,296	2656094	89.00%
F13-6	2,494,795	2116993	84.86%
F13-7	2,405,348	2092146	86.98%
F13-8	2,683,020	2335542	87.05%
F40-1	2,911,574	2517434	86.46%
F40-2	2,581,382	2140123	82.91%
F40-3	2,473,451	2107634	85.21%
F40-4	2,747,333	2240158	81.54%
F40-5	4,308,537	3845442	89.25%
F40-6	3,575,450	3159672	88.37%
F40-7	2,521,622	2213607	87.79%
F40-8	2,811,906	2427625	86.33%
