# Supplementary figures and images for: Effects of Maternal High-Fructose Diet on Long Non-Coding RNAs and Anxiety-like Behaviors in Offspring
Source: Int J Mol Sci. 2023 Feb 24;24(5):4460. doi: 10.3390/ijms24054460 (PMC10003385; doi:10.3390/ijms24054460)

Red

Blue

Yellow

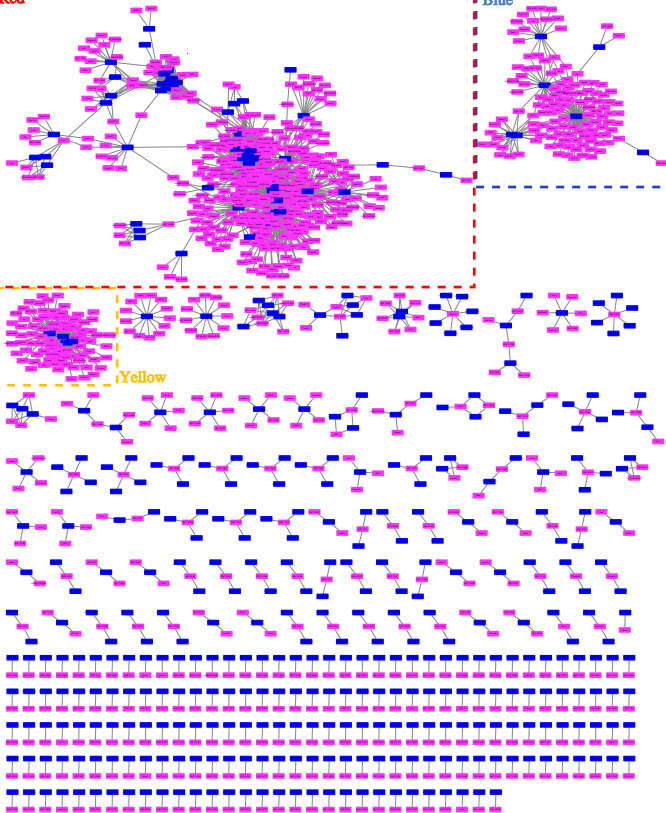

Supplement: Supplementary file 1 [file ijms-24-04460-s001.zip › Figure S1.pdf]

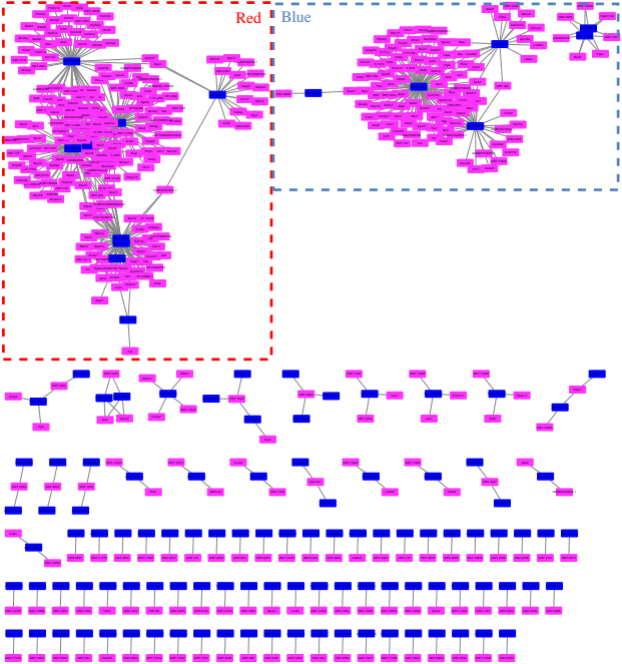

Supplement: Supplementary file 1 [file ijms-24-04460-s001.zip › Figure S2.pdf]

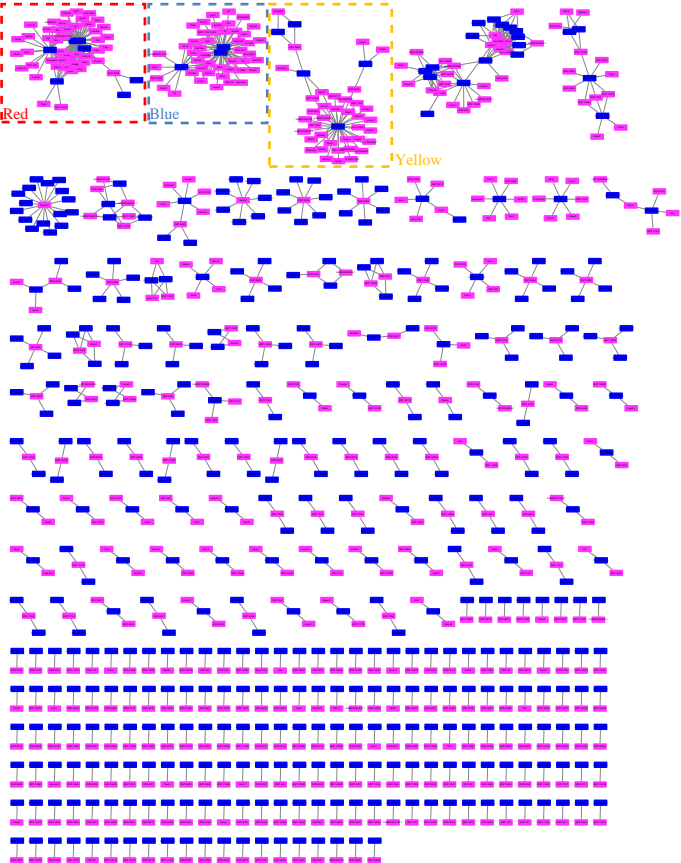

Supplement: Supplementary file 1 [file ijms-24-04460-s001.zip › Figure S3.pdf]

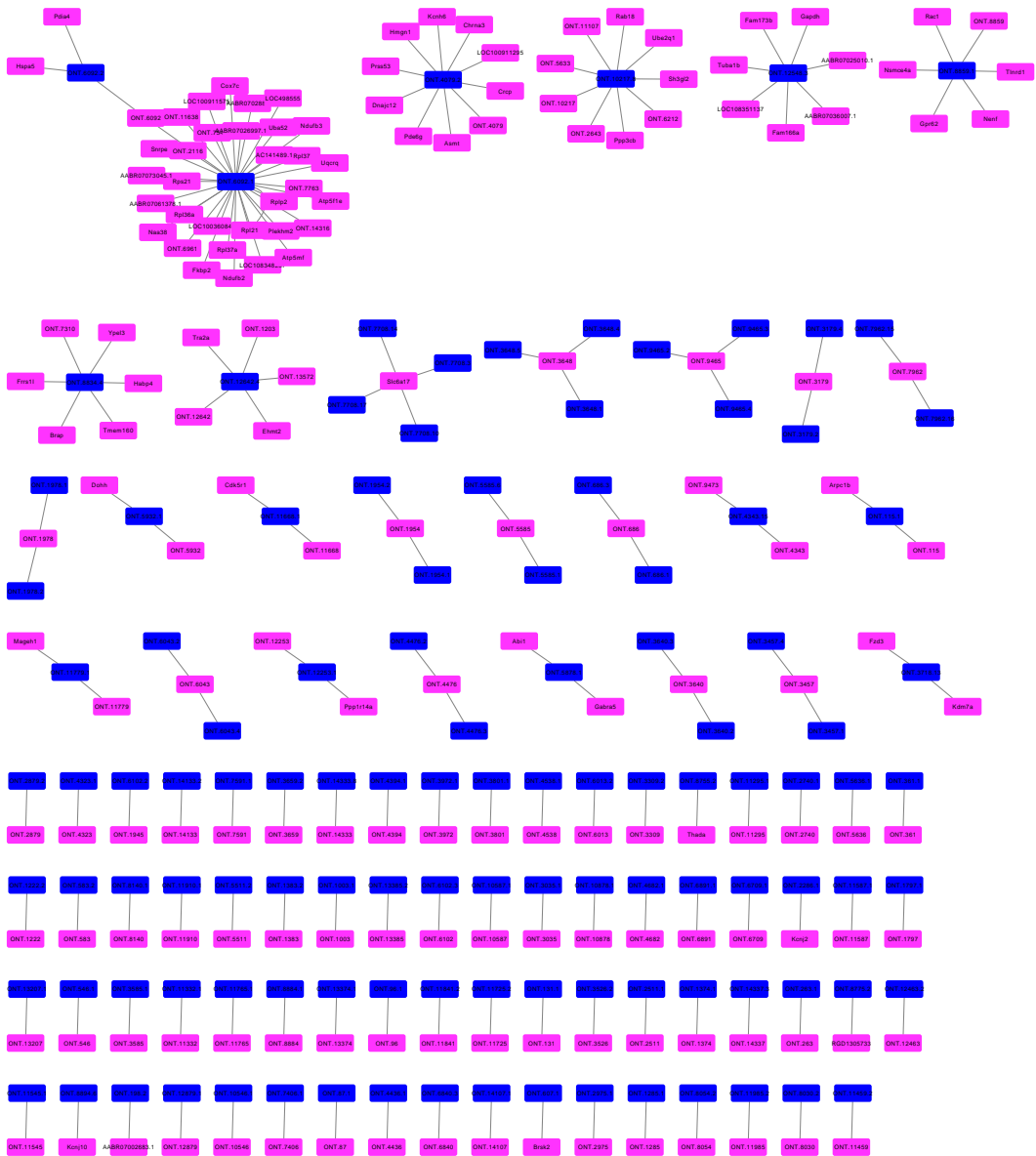

Supplement: Supplementary file 1 [file ijms-24-04460-s001.zip › Figure S4.pdf]
